# Identification of Novel Small-Molecule Janus Kinase 1 and 2 Inhibitors Through Structure-Based Virtual Screening, *In Vitro* Assays, and Molecular Dynamics Simulations

**DOI:** 10.1021/acsomega.6c02611

**Published:** 2026-07-25

**Authors:** Ahmet Avci, Hayrünnisa Taşci, Begüm Nurpelin Sağlık Özkan, Birsen Tozkoparan, Nesrin Gökhan Kelekçi

**Affiliations:** † Department of Pharmaceutical Chemistry, Faculty of Pharmacy, 37515 Hacettepe University, Ankara 06100, Turkey; ‡ Department of Pharmaceutical Chemistry, Faculty of Pharmacy, 52944 Anadolu University, Eskişehir 26470, Turkey

## Abstract

Janus kinases (JAKs) are central mediators of cytokine-driven signal transducer and activator of transcription (STAT) signaling, and dysregulation of the JAK–STAT axis is implicated in inflammatory, autoimmune, and neoplastic diseases. To identify new small-molecule inhibitors of Janus kinase 1 (JAK1) and Janus kinase 2 (JAK2), we implemented an integrated virtual screening workflow combining structure-based docking and ligand-based prioritization using the InterBioScreen (IBS) compound library. A drug-likeness filter reduced 521,627 IBS molecules to 116,064 candidates, which were then docked into the ATP-binding sites of JAK1 (PDB ID: 4EI4) and JAK2 (PDB ID: 6VGL) using Glide SP; compounds were prioritized using docking score thresholds of <− 8.5 kcal/mol (JAK1) and <− 9.0 kcal/mol (JAK2), yielding 407 JAK1- and 298 JAK2-focused hits. Subsequent analysis shortlisted 42 candidates for enzymatic evaluation. *In vitro* kinase assays identified multiple nanomolar inhibitors, including compound **1–3** (IC_50_ = 0.032 μM) among the most potent for JAK1 and compound **2–8** (IC_50_ = 0.026 μM) for JAK2. Finally, 100 ns molecular dynamics (MD) simulations supported stable binding for representative complexes, with persistent hinge-region interactions for compound **1–3** in JAK1 (e.g., Glu957/Leu959) and compound **2–8** in JAK2 (e.g., Glu930/Leu932), consistent with a stable interaction network in the active site. Collectively, these results define validated IBS-derived hit scaffolds for further optimization and selectivity profiling toward JAK-targeted therapeutics.

## Graphical abstract

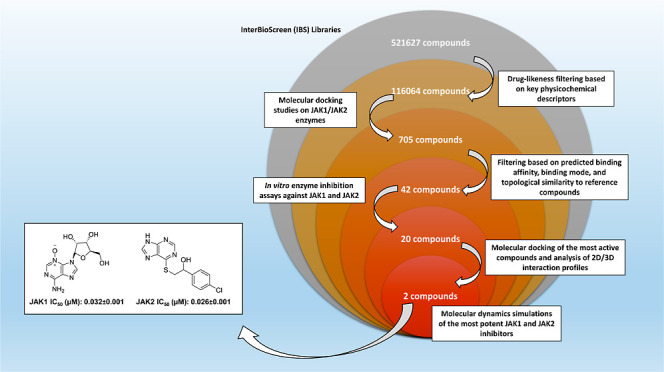

## 1 Introduction

Janus kinases (JAK1, JAK2, JAK3, and TYK2) are multidomain tyrosine kinases that associate with cytokine receptors and trigger the JAK–STAT (Janus kinase–signal transducer and activator of transcription) signaling cascade.^1^ The JAK–STAT pathway is a conserved mechanism across diverse organisms, responsible for transducing signals that regulate development and maintain homeostasis. In mammals, the JAK–STAT pathway constitutes a principal signaling route for cytokines and growth factors and contributes to the regulation of gene expression programs that control cell proliferation, migration, and apoptosis.

Disruption of the JAK–STAT signaling mechanism activated by cytokines, interleukins, and growth factors has been shown to contribute to the pathogenesis of autoimmune, inflammatory, and neoplastic diseases (Figure 1). Accordingly, this pathway represents a major therapeutic target in both immunology and oncology.^2–10^ Findings from recent studies further support the view that JAK–STAT signaling constitutes a critical target for the treatment of numerous chronic disorders. Among the Janus kinase family, JAK1 has been reported as a therapeutic target for pruritic dermatitis,^11^ allergic rhinitis,^12^ asthma,^13^ and inflammatory bowel disease,^14^ whereas combined targeting of JAK1 and JAK2 has been implicated in the treatment of rheumatoid arthritis and psoriasis. Moreover, the therapeutic potential of selective JAK3 inhibition has been investigated for rheumatoid arthritis.^15^ Dual inhibition of JAK1/TYK2 has been proposed as a promising strategy for inflammatory diseases,^16^ and selective TYK2 inhibition has been emphasized as potentially beneficial in autoimmune disorders.^17^

**Figure 1:**
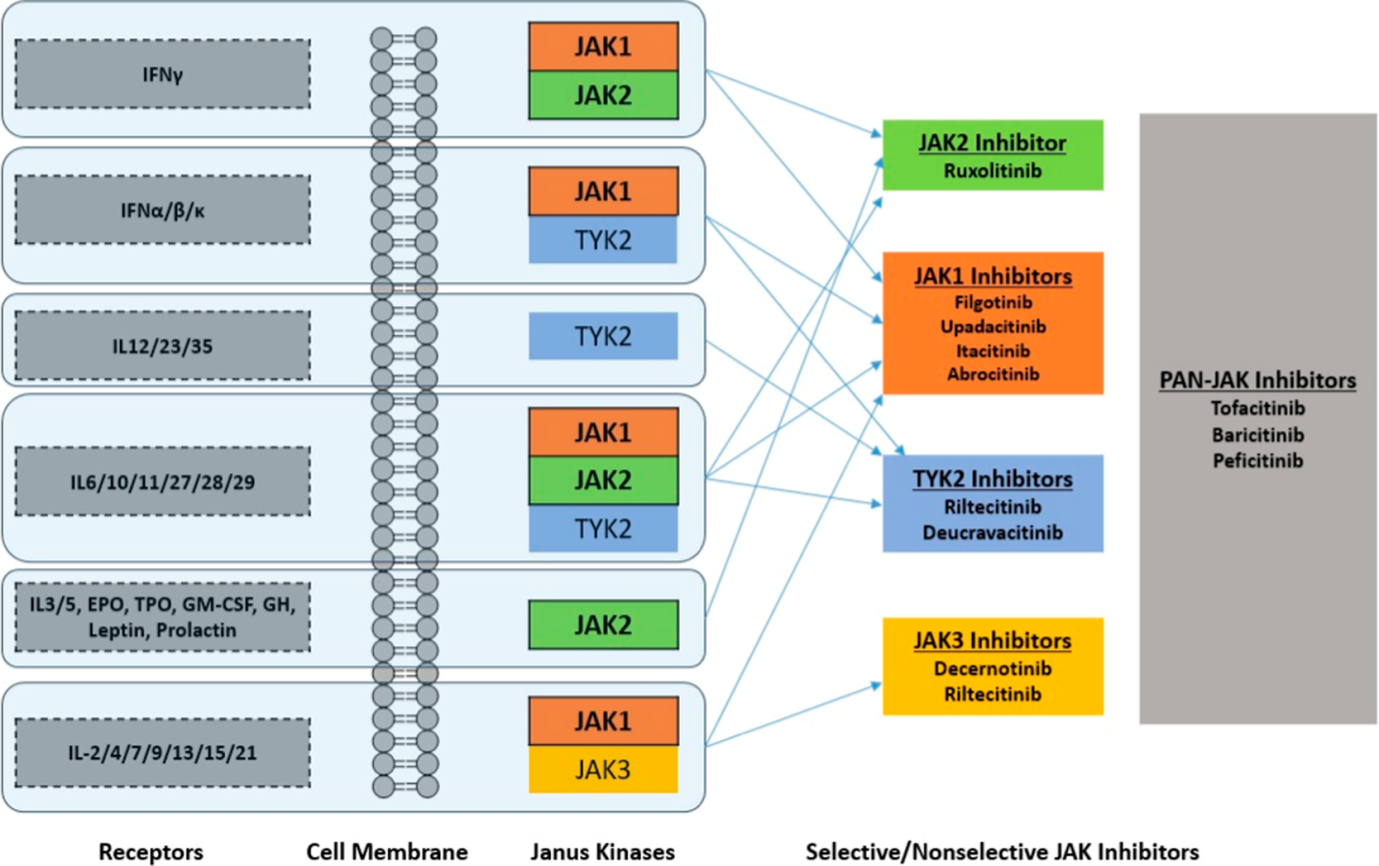
Different cytokines and their receptors interacting with the different isoforms of JAK.^20^ This figure was created by the authors based on the scientific background discussed in the cited literature, no previously published graphical material was reproduced or adapted.

In solid tumors, identification of the role of the JAK/STAT3 signaling pathway in regulating cell proliferation and angiogenesis^18^ has strengthened the mechanistic link between cancer and JAK–STAT signaling, and thereby underscored its potential as a therapeutic target in oncology. Furthermore, the occurrence of JAK2 mutations in myeloproliferative neoplasms^19^ has brought attention to JAK inhibition as an effective and clinically relevant therapeutic approach for the management these disorders.^1^

Accumulating evidence supports the concept that inhibition of JAK enzymes can confer immunomodulatory, anti-inflammatory, and anticancer effects. Among the small molecules designed to inhibit JAKs, several candidates are currently under evaluation in ongoing clinical phase trials, whereas others have received approval from the U.S. Food and Drug Administration (FDA) and entered clinical use.

The first generation of clinically approved JAK inhibitors include ruxolitinib (**1**) (JAK1/2), tofacitinib (**2**) (JAK1/3), baricitinib (**3**) (JAK1/2), and peficitinib (**4**) (pan-JAK), filgotinib **(5)** (JAK1), and upadacitinib **(6)** (JAK1), itacitinib **(7)**, and abrocitinib **(8)** (Figure 2). Despite their proven therapeutic efficacy, these agents are associated with class-related safety concerns most notably an increased risk of infections—as well as limitations related to kinase selectivity.^21–38^ Consequently, the substantial therapeutic benefit provided by JAK inhibitors is tempered by adverse effects and selectivity challenges. There is therefore a growing need to discover more effective and selective molecules with improved safety profiles.

**Figure 2:**
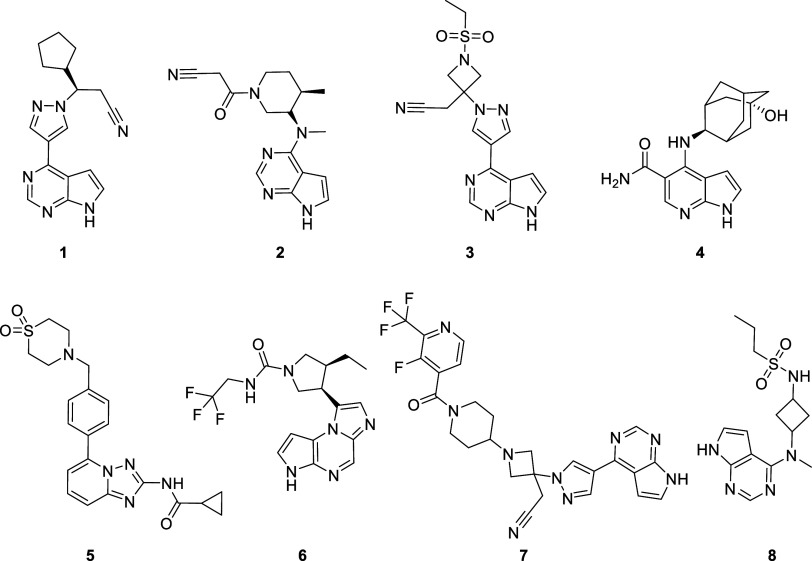
Structures of ruxolitinib (1), tofacitinib (2), baricitinib (3), peficitinib (4), filgotinib (5), and upadacitinib (6), itacitinib (7), abrocitinib (8).

Ruxolitinib competitively inhibits the ATP-binding catalytic domain of JAK1/JAK2 protein kinases,^39^ resulting in disruption of cytokine and growth factor signaling pathways and reduction of pro-inflammatory cytokines and chemokines that are elevated in myelofibrosis and other inflammatory conditions.

Tofacitinib exerts its effect by exhibiting more selective inhibitory activity on JAK1/JAK3 isoenzymes, restricting the transmission of intracellular growth factor and cytokine-mediated signals via the JAK-STAT pathway. In this way, it reduces inflammation by blocking the phosphorylation and intracellular activation of transcription activators.^40^ Tofacitinib is approved for rheumatoid arthritis (RA), psoriatic arthritis, ankylosing spondylitis, and polyarticular juvenile idiopathic arthritis.

Baricitinib, classified as a disease-modifying antirheumatic drug (DMARD), functions by inhibiting JAK1/JAK2 proteins and preventing the phosphorylation and activation of STATs. Additionally, baricitinib regulates the signaling pathways of various interleukins, interferons, and growth factors.^41^ In COVID-19 patients experiencing rapidly escalating oxygen requirements and systemic inflammation, the National Institutes of Health recommends dexamethasone and suggests considering baricitinib as a secondary immunomodulatory drug.

Peficitinib is a next-generation JAK inhibitor that irreversibly inhibits the activities of JAK1, JAK2, JAK3, and TYK2 enzymes, showing moderate selectivity for JAK3. It is approved for the treatment of RA patients who have not responded adequately to DMARDs, including methotrexate. It is currently used in Japan and Korea.^42^

Filgotinib and upadacitinib are second-generation JAK inhibitors approved for rheumatoid arthritis. Upadacitinib is also approved for the treatment of psoriatic arthritis, ankylosing spondylitis, nonradiographic axial spondyloarthritis, atopic dermatitis, Crohn’s disease, and ulcerative colitis.^20^

Abrocitinib, a reversible inhibitor of JAK1, acts by disrupting the STAT pathway by blocking the enzyme’s ATP-binding site, similar to other JAK inhibitors. It is indicated for the treatment of atopic dermatitis.^43^

Driven by advances in technology and the continuous expansion of scientific knowledge, rational drug design strategies have gained increasing importance in the discovery of small organic molecules as enzyme inhibitors. Among these approaches, virtual screening has emerged as a widely adopted and practical tool, enabling the rapid prioritization of compounds with high predicted binding affinity from large chemical libraries. Importantly, virtual screening approaches are widely used in drug discovery and have yielded successful outcomes in identifying biologically active compounds; however, the relationship between predicted binding affinity and experimental activity is not always straightforward, and therefore requires experimental validation.^39–45^

In light of these considerations, a structure-based virtual screening workflow was employed to identify new and potent JAK inhibitors with a particular focus on JAK1 and JAK2. The IBS (InterBioScreen) compound library, comprising 521,627 compounds of both natural and synthetic origin, was selected for this purpose. The virtual screening study is summarized in Scheme 1, and details are presented under the Materials and Methods heading.

**Scheme 1:**
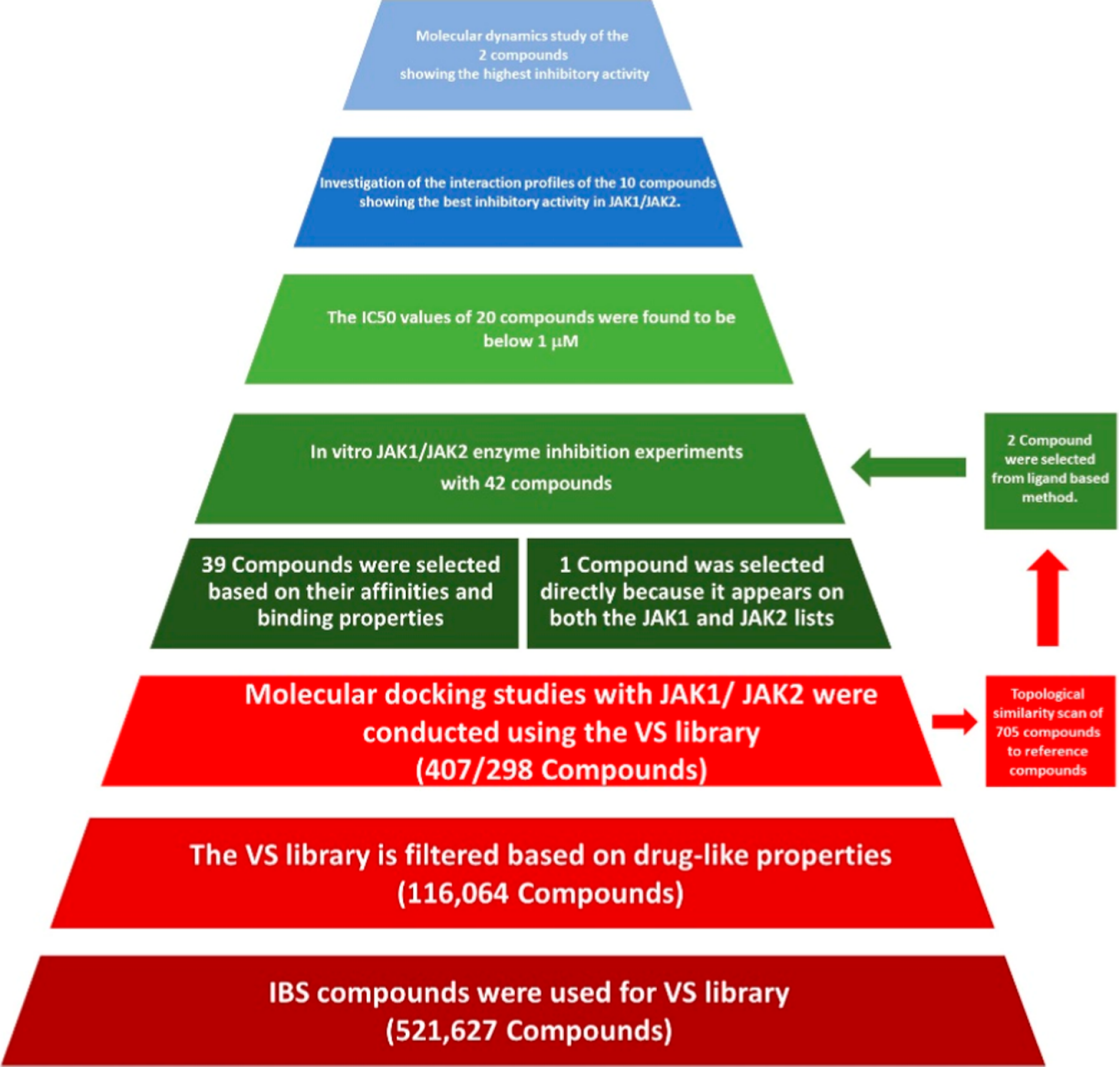
Stages of Virtual Screening Study

## 2 Materials and Methods

### 2.1 Molecular Modeling and Virtual Screening

#### 2.1.1 Library Preparation

The IBS natural and synthetic compound libraries were downloaded (https://www.ibscreen.com, accessed on March 2022) and were prepared using LigPrep.^46^ As part of the preparation workflow, salts and counterions were eliminated, multiple plausible tautomeric forms were taken into account, and all generated structures were subsequently energy-minimized using the conjugate gradients algorithm with the OPLS3e force field (2019-4, Schrödinger, LLC, New York, NY). The final prepared compounds were subsequently used for the calculation of molecular descriptors, three-dimensional topological similarity screening, molecular docking studies, and molecular dynamics simulations. Lipinski’s rule of five, along with key molecular descriptors such as molecular weight, LogP, hydrogen bond donor and acceptor numbers, and total polar surface area (tPSA), were evaluated using QikProp.^47^

#### 2.1.2 Protein Preparation

For docking studies on Janus Kinase 1 and 2 proteins, JAK1 (PDB ID: 4EI4)^48^ and JAK2 (PDB ID: 6VGL)^49^ were selected from the Protein Data Bank (www.rcsb.org). Protein structures were prepared using the Protein Preparation Wizard implemented in the Schrödinger software suite.^50^ During this preparation process, missing hydrogen atoms were added, bond orders were assigned, and bond geometries were refined using the OPLS 2005 force field. The protonation states of ionizable amino acid residues were automatically determined under the specified physiological conditions. Crystallographic water molecules located at distances greater than 5 Å from the cocrystallized ligands were removed to avoid interference with ligand binding.

#### 2.1.3 Molecular Docking

Molecular docking studies were conducted using the Glide module of the Schrödinger software suite.^51^ The docking grid was defined by centering the search space on the centroid coordinates of the cocrystallized inhibitors located in the active sites of JAK1 and JAK2, namely (1*R*,3*R*)-3-(2-methylimidazo4,5-dpyrrolo2,3-bpyridin-1­(8H)-yl)­cyclohexanol (7.2043, 58.5498, 0.3554) and ruxolitinib (57.1304, −4.1443, 24.4102), respectively. Docking was carried out in the virtual screening mode, generating five poses for each ligand. The resulting binding energies are reported in kcal/mol. Docking calculations were performed using a rigid receptor model within the Glide virtual screening workflow. No explicit receptor flexibility was considered during docking; however, molecular dynamics simulations were subsequently performed to evaluate the dynamic stability of the ligand–protein complexes. Water molecules located more than 5 Å away from the ligand within the active site were removed. Following the redocking procedure performed for validation of the docking parameters, the RMSD values were found to be 0.6944 Å for JAK1 and 0.7425 Å for JAK2, and their two-dimensional images are shown in the Supporting Information. The resulting ligand–protein interactions were examined in detail through comprehensive analysis of both two- and three-dimensional interaction diagrams.

#### 2.1.4 Ligand-Based Methods

Ligand-based three-dimensional shape similarity analysis was conducted using the shape screening workflow of the phase module implemented in the Schrödinger software suite.^52^ The screening procedure was executed in simple run mode, with the lower threshold for library filtering set to 0.5.

### 2.2 Purchased Compounds

All 42 compounds used in this study were purchased from IBS (InterBioScreen, Sutomore, Montenegro). Certificates of compounds provided by IBS accompanied each compound and verified their stated purity. The purity of all compounds was determined by the manufacturer using NMR spectroscopy. Copy of the certificate is provided in the Supporting Information. All compounds were stored and handled according to the manufacturer’s recommendations prior to use in biological activity assays.

### 2.3 *In Vitro* JAK1 and JAK2 Inhibitory Screening Assays

*In vitro* JAK1 and JAK2 inhibitory screening assays were performed in accordance with the protocols provided in the JAK1 (Janus Kinase 1) and JAK2 (Janus Kinase 2) Assay Kits. The experimental procedures were conducted as described by the manufacturer^53,54^ and detailed descriptions of the assay protocols are provided in the Supporting Information. The IC_50_ values were calculated by nonlinear regression analysis using a four-parameter logistic (4PL) equation to fit the dose–response data. The inhibition percentages were plotted against the log concentrations of the inhibitors, and the IC_50_ values were derived as the concentration resulting in a 50% reduction in enzyme activity. All experiments were performed in triplicate, and the results are expressed as mean ± standard deviation (SD). Statistical analysis and curve fitting were performed using GraphPad Prism software, ensuring that all reported values exhibited a coefficient of determination (*R* ^2^) > 0.98.

### 2.4 Molecular Dynamics Studies

Molecular dynamics (MD) simulations, which are considered an important computational tool for evaluating the time-dependent stability of a ligand within the active site of a drug–receptor complex, were performed in this study for compounds **1–3** (JAK-1 enzyme) and **2–8** (JAK-2 enzyme).^55^ To assess the stability of the complexes obtained from docking, 100 ns MD simulations were carried out.

The dynamic simulations were performed using the Desmond application^56^ with a three-point water model (TIP3P), followed by energy minimization of the complex,^57^ employing the Schrödinger Suite standard force field with transferable intermolecular potentials (OPLS3e). System neutralization was achieved by adding Na^+^ and Cl^–^ ions to obtain a final salt concentration of 0.15 M, thereby mimicking the physiological concentration of monovalent ions.^58^ Constant temperature (310.55 K) and pressure (1.01325 bar) were maintained using the *NPT* ensemble (constant number of particles, pressure, and temperature). The RESPA integrator^59^ was used to integrate the equations of motion. NH thermostats^60^ were employed to keep the simulation temperature constant, and the MTK method^61^ was applied to control the pressure. Long-range electrostatic interactions were calculated using the particle mesh Ewald (PME) method.^62^ The cutoff for van der Waals and short-range electrostatic interactions was set to 9.0 Å. The protein–ligand complexes were solvated in an orthorhombic box with a minimum distance of 10 Å between the solute and the box boundaries to ensure periodic boundary conditions (PBC). The system for the JAK1 complex consisted of approximately 12,450 TIP3P water molecules, while the JAK2 complex contained 11,800 water molecules. The final dimensions of the equilibrated simulation boxes were approximately 90 Å × 90 Å × 90 Å.

System equilibration was performed using the default protocol provided in Desmond, which consists of a series of restrained minimization and molecular dynamics simulations designed to gradually relax the system. Following completion of system setup, the MD simulation was run using the settings described above. The radius of gyration (Rg), root-mean-square fluctuation (RMSF), and root-mean-square deviation (RMSD) values were calculated using the Desmond application.^56^

## 3 Results and Discussion

### 3.1 Druglikeness Filtering

The IBS database (521,627 compounds) was subjected to a virtual screening workflow, beginning with drug-likeness filtering. At this stage of the workflow, compounds with unfavorable ADMET (absorption, distribution, metabolism, excretion and toxicity) profiles were eliminated by calculating key physicochemical descriptors associated with orally drug-like chemical space using QikProp.^63,64^ These descriptors included molecular weight (130–725 Da), number of rotatable bonds (0–15), hydrogen bond donor and acceptor counts (0–6 and 2–20, respectively), logP (−2 to −6), and topological polar surface area (7–200 Å^2^). Only compounds within the software-defined ideal ranges were retained, resulting in approximately 116,064 molecules being selected for subsequent screening steps. These thresholds were selected in accordance with widely accepted drug-likeness criteria (Lipinski and Veber rules) to retain compounds within orally bioavailable chemical space.

### 3.2 Molecular Docking

At this stage of the study, binding-energy cutoff values were defined to prioritize compounds with potential JAK1 and JAK2 inhibitory activity. Based on redocking results, lower binding-energy thresholds were set at −8.5 kcal/mol for JAK1 (redocking score: −9.5 kcal/mol) and −9.0 kcal/mol for JAK2 (redocking score: −10.8 kcal/mol), respectively. The selected thresholds were chosen to retain compounds with binding energies reasonably close to those of cocrystallized reference ligands, while maintaining a manageable number of candidates. According to these criteria, 407 compounds were identified as putative JAK1 inhibitors, while 298 compounds were selected as candidates for JAK2 inhibition. The results of the docking results of 705 compounds are given in the Supporting Information.

The selection of compounds for subsequent *in vitro* evaluation was further refined through detailed analysis of their binding interaction profiles, with particular emphasis on key amino acid residues identified from the crystallographic binding modes of the cocrystallized reference ligands.

In this context, the interaction patterns of ruxolitinib in the JAK2 crystal structure and that of (1*R*,3*R*)-3-(2-methylimidazo­[4,5-d]­pyrrolo­[2,3-b]­pyridin-1­(8H)-yl)­cyclohexanol in the JAK1 structure were used as reference benchmarks, as these residues are known to play critical roles in ligand recognition, orientation, and stabilization within the ATP-binding site. For JAK1, interactions involving Met956 (π–cation interaction), Leu959, Arg1007, and Asn1008 were considered essential, as these residues stabilize the reference ligand through a combination of hydrophobic, hydrogen-bonding, and electrostatic interactions, thereby contributing to both binding affinity and proper ligand orientation. For JAK2, interactions with Met929 and Leu932, including π–cation and hydrophobic contacts observed in the ruxolitinib-bound crystal structure, were defined as priority interactions due to their central role in anchoring ATP-competitive inhibitors within the hinge region. In addition, hydrogen-bond interactions formed within the hinge (core) region, which are characteristic of ATP-competitive kinase inhibitors, were also evaluated to ensure biologically relevant binding modes.

Based on the combined selection criteria, the 705 compounds that satisfied the docking threshold were subjected to detailed binding mode analysis. At this stage, prioritization was guided by the ability of the compounds to establish key interactions with the predefined amino acid residues, particularly those involved in hinge-region hydrogen bonding and interactions observed for the cocrystallized reference ligands. Compounds that failed to reproduce these critical binding features were systematically excluded, resulting in a refined subset of candidates.

To further reduce redundancy and ensure broader chemical representation, the final selection also incorporated structural diversity considerations. Accordingly, compounds were chosen to reflect a range of distinct chemical scaffolds rather than focusing on a single chemotype. Through this integrated filtering and diversification strategy, a total of 39 compounds were ultimately selected for *in vitro* evaluation. Moreover, based on its favorable docking energies, STOCK2S-34232 (compound **1–10**/**2–8**), identified among the selected candidate lists for both JAK1 and JAK2 was also included in the activity assays irrespective of its detailed binding interaction features.

### 3.3 Ligand-Based Screening

Molecules identified through structure-based virtual screening may either reproduce key binding features observed for established reference ligands within the macromolecular active site or reveal alternative and potentially novel binding modes. In this context, candidate molecules that are structurally similar to known inhibitors are often expected to exhibit comparable binding behavior and, therefore, a higher likelihood of success in subsequent in vitro activity assays. In the present study, a ligand-based filtering strategy was intentionally not applied prior to the structure-based molecular docking stage. This decision was motivated by the aim of identifying novel chemical scaffolds with potential affinity toward JAK1 and JAK2 enzymes, rather than biasing the virtual screening workflow toward previously reported inhibitor chemotypes. Nevertheless, given the recognized value of ligand-based similarity analyses in the identification of biologically active compounds, such approaches were not entirely excluded from the study design.

Accordingly, following molecular docking and energy-based filtering, the selected candidate molecules were subjected to a postdocking ligand-based similarity assessment. Specifically, the top-ranked compounds were evaluated for their three-dimensional shape similarity to the reference JAK inhibitors ruxolitinib and (1*R*,3*R*)-3-(2-methylimidazo­[4,5-d]­pyrrolo­[2,3-b]­pyridin-1­(8H)-yl)­cyclohexanol. This analysis was performed using the shape-based screening module implemented in the Schrödinger software suite, with the aim of identifying candidates that may share comparable binding behavior with the reference ligands. As a result of the independent virtual screening workflows performed for JAK1 and JAK2, the chemical structures of the selected compounds together with their predicted binding energies within the respective enzyme active sites are summarized in Tables 1 and 2. Based on the integrated evaluation of all *in silico* results, including docking performance and shape-based similarity analysis, two compounds, compound **1–5** (STOCK3S-84679) and compound **2–16** (STOCK1N-71510) and, exhibiting similarity scores greater than 0.5 were prioritized for subsequent biological activity studies, regardless of their detailed binding interaction profiles. A similarity threshold of 0.5 was selected to avoid excessive bias toward known inhibitor chemotypes while still allowing the identification of structurally related compounds. This value represents a balance between scaffold diversity and similarity-driven prioritization.

**Table 1:**
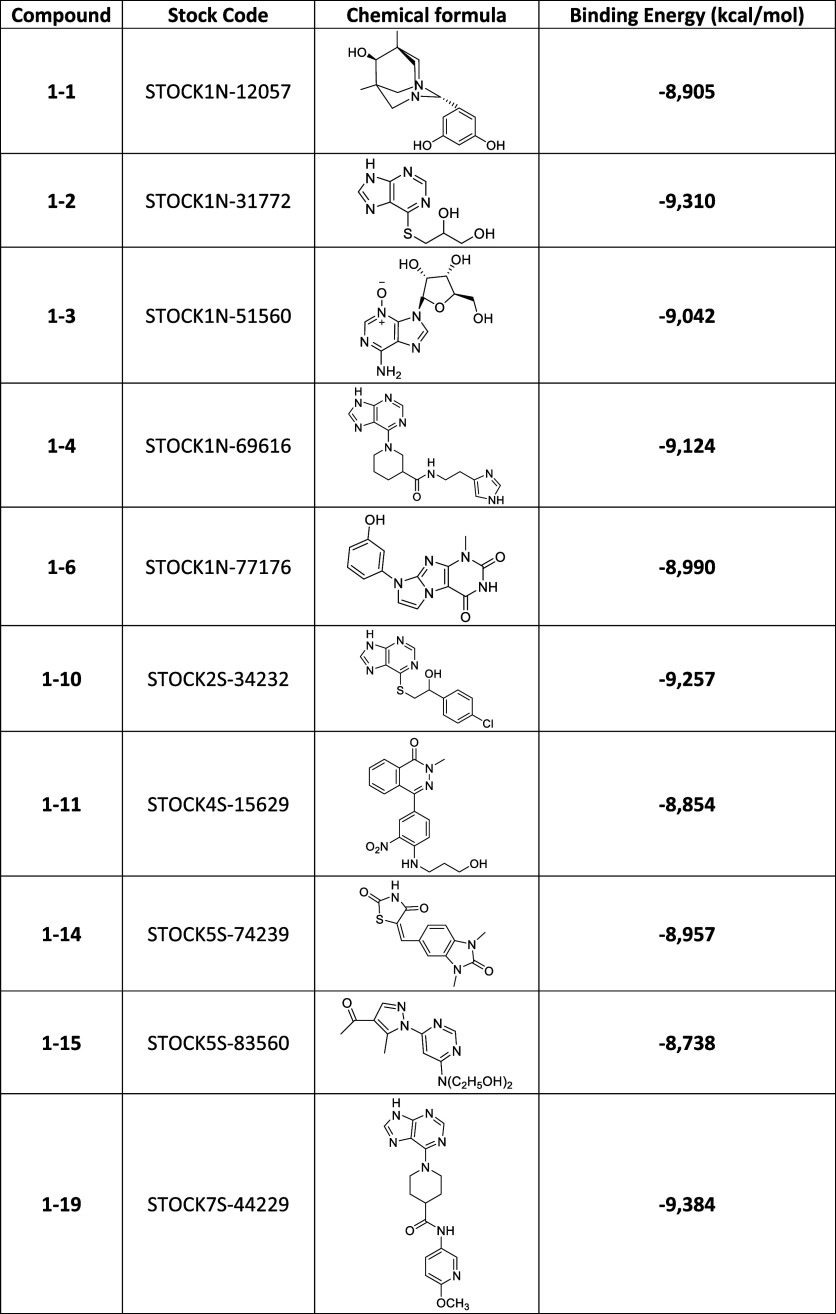
Compounds Exhibiting JAK1 Inhibitory Activity and Calculated Binding Energies

**Table 2:**
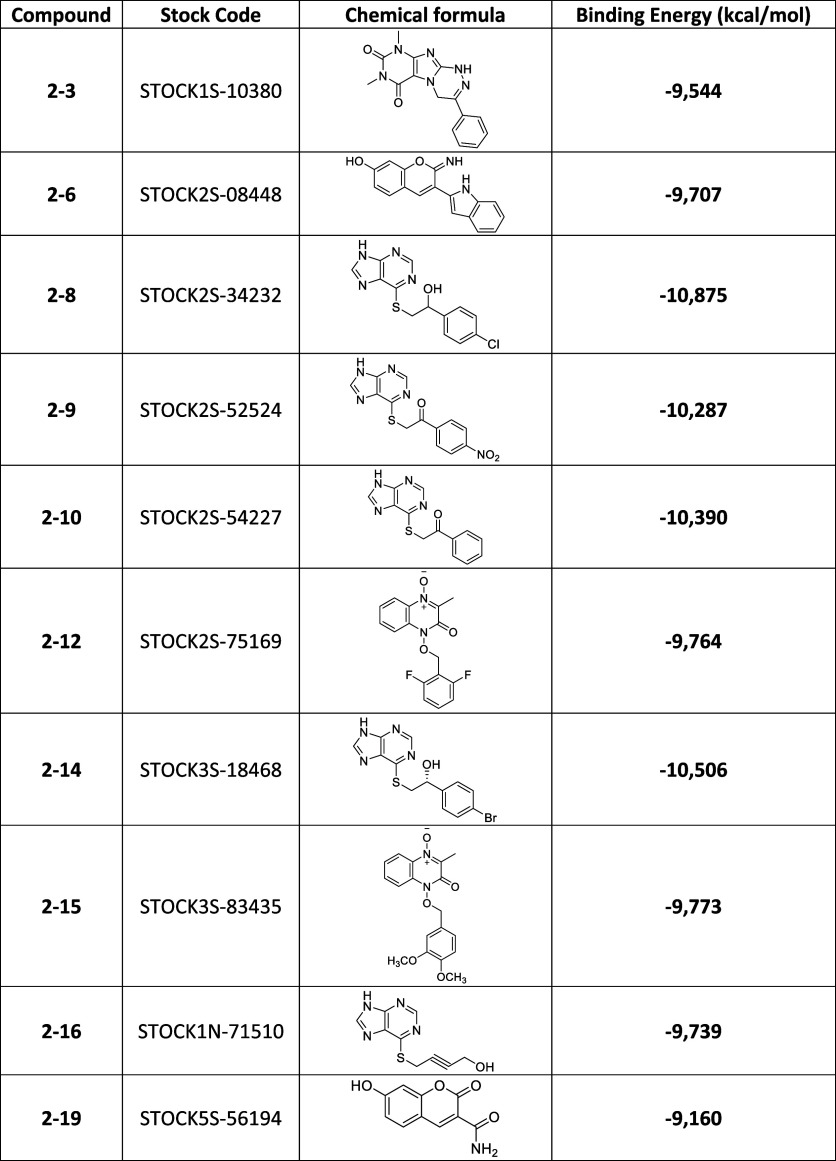
Compounds Exhibiting JAK2 Inhibitory Activity and Calculated Binding Energies

Overall, a total of 42 compounds that fulfilled the predefined docking criteria, key binding features in both enzymes, hydrogen-bonding capability, and shape-based screening filters were selected for biological activity studies. The candidate compounds identified through molecular docking studies and their corresponding binding energies are presented in Tables 1 and 2. A total of 42 compounds two-dimensional interaction files are provided in the Supporting Information.

### 3.4 Biological Activity Evaluation

The biological activities of the 42 selected compounds against JAK1 and JAK2 enzymes were assessed, and the corresponding results are presented in Table 3. Among the 42 evaluated compounds, 20 exhibited potent inhibitory activity, with IC_50_ values below 1 μM. Of these active molecules, 10 demonstrated inhibitory activity against JAK1, while the remaining 10 were effective against JAK2. Notably, compounds **1–3**, **1–4**, **1–6**, **1–10**, and **1–19** emerged as the most potent JAK1 inhibitors, whereas compounds **2–8**, **2–9**, **2–10**, **2–14**, and **2–16** were identified as the leading inhibitors of JAK2 (Table 3). It is noteworthy that not all compounds with favorable docking scores exhibited inhibitory activity *in vitro*, which reflects the inherent limitations of docking-based virtual screening approaches and underscores that empirical scoring functions function primarily as prioritization filters rather than absolute predictors of experimental binding affinities.

**Table 3:** IC_50_ Values (μM) of Compounds against JAK-1 and JAK-2 Enzymes

compounds	JAK-1 enzyme inhibition IC_50_ (μM)	compounds	JAK-2 enzyme inhibition IC_50_ (μM)
**1–1**	0.114 ± 0.005	**2–1**	>100
**1–2**	0.221 ± 0.009	**2–2**	>1000
**1–3**	0.032 ± 0.001	**2–3**	0.203 ± 0.009
**1–4**	0.083 ± 0.004	**2–4**	>100
**1–5**	>100	**2–5**	>100
**1–6**	0.055 ± 0.002	**2–6**	0.159 ± 0.007
**1–7**	>1000	**2–7**	>1000
**1–8**	>100	**2–8**	0.026 ± 0.001
**1–9**	>1000	**2–9**	0.067 ± 0.002
**1–10**	0.068 ± 0.002	**2–10**	0.080 ± 0.003
**1–11**	0.148 ± 0.006	**2–11**	>100
**1–12**	>1000	**2–12**	0.139 ± 0.006
**1–13**	>100	**2–13**	>100
**1–14**	0.277 ± 0.011	**2–14**	0.039 ± 0.001
**1–15**	0.206 ± 0.008	**2–15**	0.238 ± 0.011
**1–16**	>100	**2–16**	0.047 ± 0.002
**1–17**	>1000	**2–17**	>1000
**1–18**	>1000	**2–18**	>100
**1–19**	0.072 ± 0.003	**2–19**	0.162 ± 0.005
**1–20**	>1000	**2–20**	>1000
**1–21**	>100	**2–21**	>1000
Upadacitinib	0.003 ± 0.0001	staurosporine	0.002 ± 0.0001

Structural analysis of the compounds exhibiting activity against JAK1 and/or JAK2 revealed that nine molecules shared a common 6-substituted-9*H*-purine scaffold (**1–2**, **1–3**, **1–4**, **1–10/2–8**, **1–19**, **2–9**, **2–10**, **2–14**, and **2–16**). Among these, seven compounds carried a sulfur-containing substituent at the C-6 position, whereas two incorporated a piperidine moiety. The active purine derivatives identified in this study preserve the canonical hinge-binding characteristics commonly observed in ATP-competitive kinase inhibitors. This behavior is consistent with the conserved organization of kinase ATP-binding sites, in which heterocyclic systems capable of mimicking adenine interactions frequently serve as hinge-binding motifs. Despite these shared interaction features, the identified compounds differ from clinically established JAK inhibitors in the structural organization of the substituents extending from the purine core. In particular, the present findings suggest that sulfur-substituted alkyl groups directly attached at the C-6 position of the 9H-purine scaffold can be favorably accommodated within the JAK ATP-binding site while maintaining productive hinge-binding interactions. The observed activity profiles suggest that sulfur-containing C-6 substitutions are compatible with productive JAK binding and may provide additional SAR insights for the future development of structurally differentiated JAK inhibitors.

A more detailed structure–activity relationship (SAR) analysis of the purine-based derivatives revealed that substitution patterns on the core scaffold critically influence inhibitory activity. While the purine nucleus is essential for establishing canonical hinge-region interactions characteristic of ATP-competitive kinase inhibitors, substitutions at positions other than C-6 were generally poorly tolerated, suggesting a stringent steric and electronic requirement around the core scaffold.

In particular, compounds bearing substituents at the C-2 position (e.g., **1–13** and **2–7**) were inactive, suggesting that steric constraints within the hinge region likely impede the adoption of a productive binding orientation upon substitution at this position. Similarly, introduction of hydrogen bond donor or acceptor functionalities at the C-8 position (e.g., **1–17**, **2–17**, **2–20**, and **2–21**) also resulted in a complete loss of activity, which may be attributed to perturbation of the optimal hydrogen-bonding network and/or unfavorable desolvation penalties within the binding pocket.

Notably, substitution at the C-6 position was well tolerated and, in several cases, beneficial. Compounds bearing bulky substituents at this position such as sulfur-containing groups or cyclic amines (e.g., piperidine) (**1–2**, **1–4**, **1–10**, **1–19**, **2–8**, **2–9**, **2–10**, and **2–16**) exhibited enhanced activity, suggesting that this region of the binding pocket can accommodate sterically larger groups and likely corresponds to a hydrophobic subpocket.

In addition, several of the most active compounds contained polar functional groups attached to the C-6 substituent, capable of acting as hydrogen bond donors or acceptors. These functionalities may contribute to ligand stabilization through water-mediated interactions and/or by facilitating favorable orientation within partially solvent-exposed regions of the binding site. This observation is further supported by molecular dynamics simulations, which indicated the involvement of water-mediated hydrogen bonding networks in stabilizing the ligand–protein complexes.

Interestingly, compound **1–3**, which bears a substituted furan moiety at a different position, also displayed high activity. This may indicate an alternative binding orientation, in which the ligand adopts a conformation that preserves hinge interactions via the purine NH_2_ group while allowing additional substituents to extend into less sterically constrained regions of the binding pocket.

Overall, these findings suggest that while the purine core is critical for hinge binding, selective substitution at the C-6 position represents a key strategy for optimizing activity, whereas modifications at other positions are generally less favorable. The combination of steric compatibility and appropriately positioned polar functionalities appears to play a central role in determining inhibitory potency.

In addition to the purine-based series, other scaffold classes were also identified among the selected compounds. The compounds **2–12** and **2–15**, active against JAK2, featured a dihydroquinoxaline scaffold. In both active dihydroquinoxaline derivatives, the hydroxyl group attached to the N-1 position was predicted to form a hydrogen-bond interaction with the hinge-region residue Leu932, thereby contributing to anchoring of the scaffold within the ATP-binding site. In addition, the aryloxybenzyl substituent located at the C-4 position extends toward the same peripheral pocket region occupied by the C-6 substituents of the active purine derivatives, where it contributes to binding through hydrophobic interactions. A similar binding orientation was observed for both dihydroquinoxaline derivatives, suggesting a conserved interaction pattern within this scaffold class. Interestingly, this overall binding organization partially resembles that of the active purine-based inhibitors, particularly with respect to the spatial separation between the hinge-binding motif and the hydrophobic peripheral substituent. These observations suggest that further modification of the aryl–alkyl extension at the C-4 position may represent a promising strategy for future optimization of this scaffold. Collectively, the obtained findings indicate that the dihydroquinoxaline ring system may represent an alternative heterocyclic framework capable of supporting productive JAK inhibitory activity.

The 2*H*-chromene scaffold also emerged as a distinct subgroup among the JAK2-active compounds. Both active derivatives (**2–6** and **2–19**) displayed a broadly similar binding organization, although their predicted interaction pattern differed from those observed for the purine and dihydroquinoxaline scaffolds. In both compounds, the hydroxyl group located at the C-7 position was predicted to establish hydrogen-bond and polar interactions with the hinge-region residues Glu930 and Leu932, thereby contributing to anchoring of the scaffold within the ATP-binding site. In contrast, the substituents attached at the C-3 position were oriented toward the peripheral hydrophobic pocket region. While compound **2–6** contains a benzimidazole moiety at this position, compound **2–19** bears an amide-containing substituent. The observed activity of both derivatives, together with the apparent tolerance of the relatively bulky benzimidazole ring in compound **2–6**, suggests that the C-3 position of the 2*H*-chromene scaffold may represent a suitable region for further structural optimization. Collectively, these findings indicate that the 2*H*-chromene scaffold may also provide a promising framework for future JAK2 inhibitor development.

In contrast, the thiazolidinedione scaffold did not display a consistent activity pattern, indicating that this ring system does not provide a reliable hinge-compatible anchoring motif. Four of the evaluated compounds (**1–9**, **1–12**, **1–14**, and **2–18**) contained a thiazolidinedione moiety; however, among these, only compound **1–14** exhibited pronounced inhibitory activity, selectively targeting JAK1. Notably, compound **1–14** displayed the lowest IC_50_ value among all 20 active compounds identified in this study.

Within the *in silico*-guided selection pipeline, compound **2–16**, which was prioritized through the shape-based screening approach, demonstrated potent JAK2 inhibition with an IC_50_ value of 0.047 ± 0.002 μM, whereas compound **1–5** did not display measurable inhibitory activity. In contrast, STOCK2S-34232 (compound **1–10/2**–**8**), which was independently prioritized in both the JAK1 and JAK2 workflows and therefore advanced to biological evaluation irrespective of its predicted binding mode, exhibited remarkable dual inhibitory potency, with IC_50_ values of 0.068 ± 0.002 μM against JAK1 and 0.026 ± 0.001 μM against JAK2. Selectivity between JAK1 and JAK2 remains intrinsically challenging due to the high degree of conservation within their ATP-binding sites, where direct targeting of the ATP pocket typically results in highly similar binding modes across both kinases. In this context, subtype preference is generally governed not by the canonical hinge hydrogen bonds alone, but by subtle differences in the hinge-adjacent region, front-pocket geometry, and solvent-exposed residues. At the residue level, the hinge framework is closely related but not identical, with Glu957–Phe958–Leu959 in JAK1 and Glu930–Tyr931–Leu932 in JAK2, while another notable distinction involves the solvent-exposed Glu966/Asp939 variation. By contrast, the gatekeeper residue is methionine in both kinases, limiting gatekeeper-driven selectivity.^65,66^

Compound **1–10/2–8**, which emerged from both screening pipelines, showed only an approximately 3-fold difference in IC_50_ values between JAK1 and JAK2, indicating a dual inhibitory rather than a selective profile. This behavior is consistent with its docking poses, in which the compound forms conserved hinge-region hydrogen bonds with Glu957/Leu959 in JAK1 and Glu930/Leu932 in JAK2, while not clearly exploiting the more discriminating residue-level differences around Phe958/Tyr931 or Glu966/Asp939. Taken together, these findings suggest that the observed dual activity is primarily driven by conserved hinge-binding interactions, rather than by isoform-specific contacts capable of conferring pronounced selectivity.

From a pharmacological perspective, selective JAK1 inhibition has been associated with improved safety profiles in inflammatory diseases, whereas dual JAK1/JAK2 inhibition has established clinical efficacy in disorders such as rheumatoid arthritis and myeloproliferative diseases. Accordingly, selective and dual inhibition profiles may both be considered therapeutically valuable depending on the clinical context, and should therefore be regarded as complementary rather than mutually exclusive outcomes in early stage drug discovery.

Collectively, these findings suggest that 6-substituted-9*H*-purine, 2*H*-chromene, and dihydroquinoxaline frameworks may represent promising scaffolds for the rational design of novel JAK inhibitors. Beyond their structural differentiation, the identified scaffolds also exhibited encouraging pharmacological characteristics. Multiple representatives displayed nanomolar inhibitory activity against JAK1 or JAK2 together with generally favorable predicted ADME profiles, including acceptable oral absorption and favorable physicochemical properties. These findings provide a pharmacological rationale for the further development of these scaffold classes as potential JAK inhibitor leads.

### 3.5 Insights From the *In Silico* Analysis

To characterize the potential binding interactions of the most potent inhibitors within the active sites of JAK1 and JAK2, molecular docking studies were performed using the crystal structures of JAK1 (PDB ID: 4EI4)^48^ and JAK2 (PDB ID: 6VGL).^49^ Docking calculations were carried out with the Glide software, and the most plausible binding poses were generated employing the GlideScore SP scoring function. The resulting docking poses and interaction patterns for JAK1 are illustrated in Figure 3, while the corresponding binding modes for JAK2 are presented in Figure 4.

**Figure 3:**
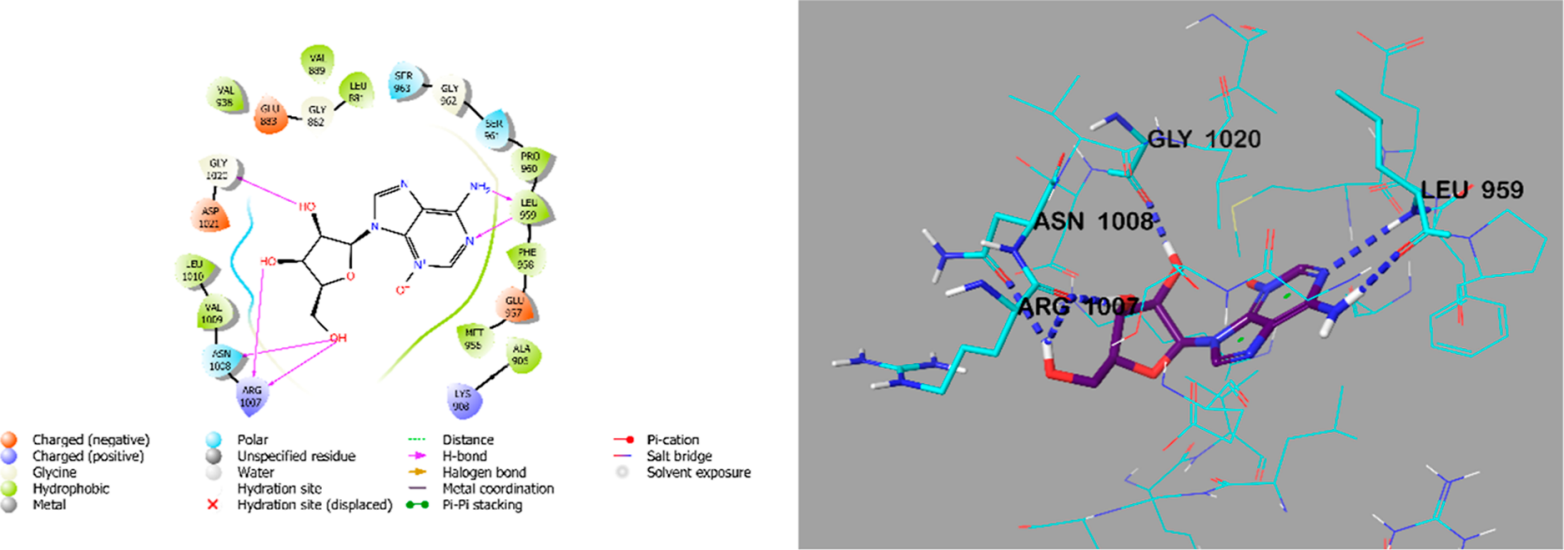
Two-dimensional (2D) and three-dimensional (3D) interaction profiles of compound **1–3** within the active site of JAK1 (PDB ID: 4EI4).

**Figure 4:**
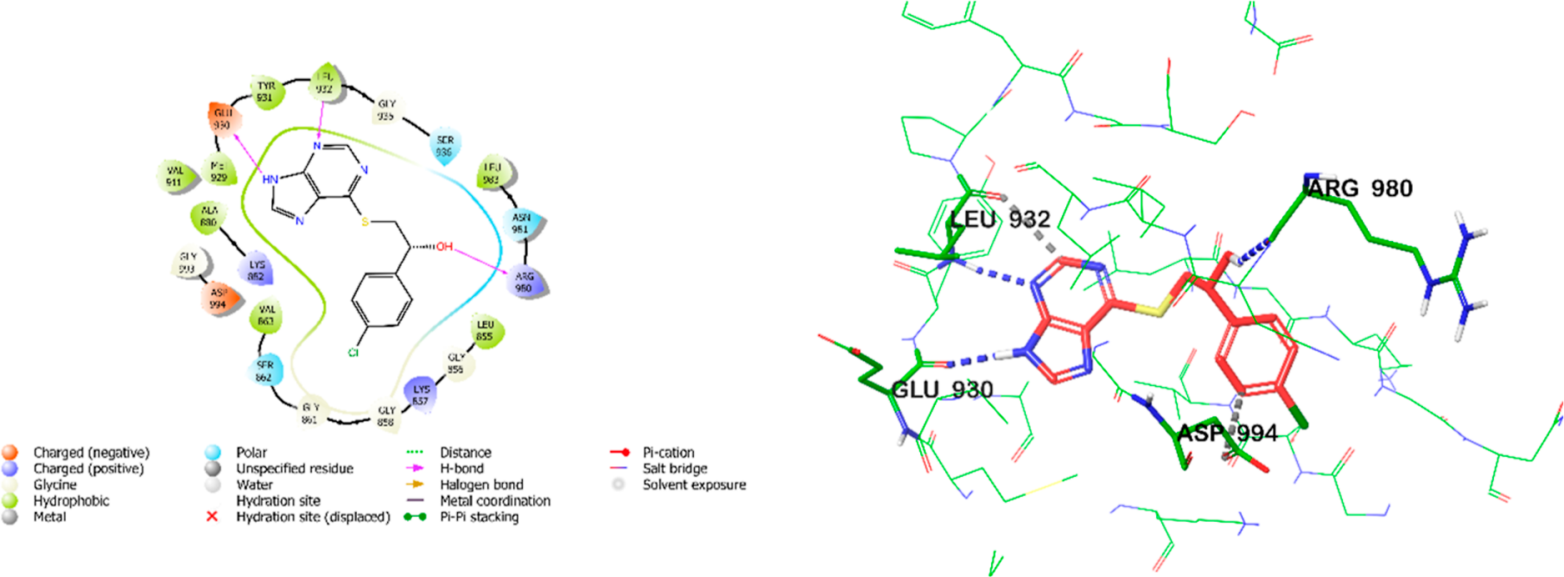
Two-dimensional (2D) and three-dimensional (3D) interaction profiles of compound **2–8** within the active site of JAK2 (PDB ID: 6VGL).

### 3.6 Molecular Docking Studies on the JAK-1 Enzyme

The two-dimensional and three-dimensional binding interactions of compounds **1–3** (Figure 3), **1–4, 1–6, 1–10,** and **1–19** (Supporting Information, Section S8) within the active side of the JAK1 enzyme depicted, analysis of the resulting docking poses revealed that hydrogen-bond interactions with Leu959 constitute a conserved feature across all five compounds. In addition, compounds **1–3**, **1–4**, and **1–10** were found to establish hydrogen bonds with Arg1007. Another recurrent interaction involves hydrogen bonding with Glu957, which was observed for derivatives **1–4**, **1–6, 1–10**, and **1–19**.

### 3.7 Molecular Docking Studies on the JAK-2 Enzyme

The two-dimensional and three-dimensional binding interactions of compounds **2–8** (Figure 4), **2–9**, **2–10**, **2–14**, and **2–16** (Supporting Information, Section S9) within the active side of the JAK2 enzyme presented, analysis of the obtained docking poses revealed several common interaction patterns among the five compounds. Notably, the adenine moiety present in all structures was found to form two key hydrogen bonds with Glu930 and Leu932, corresponding to the hinge region of the kinase. In addition, interactions with Asp994 were observed for compounds **2–8**, **2–9**, and **2–14**. Furthermore, hydrogen-bond interactions with Arg980 were identified in derivatives **2–8** and **2–14**.

### 3.8 ADME–Toxicity Profiling of Active Compounds

The ADME–Tox properties of the active compounds were further evaluated to assess their pharmacokinetic suitability and potential safety liabilities. As the initial compound library had already been filtered based on drug-likeness criteria, this analysis primarily aimed to discriminate among the active compounds with respect to key parameters, including solubility, permeability, absorption, distribution, plasma protein binding, and toxicity-related risks.

The solubility profiles of the active compounds appeared favorable across different prediction systems. According to ESOL, Ali, and Silicos-IT classifications, the compounds were generally predicted to be soluble or moderately soluble, indicating that poor aqueous solubility is unlikely to represent a major limitation for this series.

The QPlogHERG parameter was evaluated as an indicator of potential hERG channel inhibition and cardiotoxicity risk. QPlogHERG values greater than −5 are generally considered acceptable, whereas values below −5 may indicate increased hERG-related liability. Among the active compounds, 8 of 20 were within the acceptable range, while 12 compounds showed values slightly below this threshold. Therefore, although the predicted hERG risk does not appear prohibitive, it should be considered during future optimization.

Caco-2 permeability was assessed as an indicator of intestinal membrane permeability. Values below 25 nm/s are generally considered low, values between 25 and 500 nm/s indicate moderate permeability, and values above 500 nm/s indicate high permeability. In this series, 19 of 20 active compounds showed acceptable Caco-2 permeability values above 25 nm/s, including 7 compounds with high predicted permeability above 500 nm/s. Only compound **2–8** showed low Caco-2 permeability.

The predicted blood–brain barrier penetration values (QPlogBB) were within the recommended QikProp range of −3.0 to 1.2 for all active compounds. However, most values were low or negative, suggesting limited central nervous system penetration. This may be considered favorable for JAK inhibitors intended for peripheral inflammatory or dermatological indications, where CNS exposure is not required.

MDCK permeability (QPPMDCK), reflecting general membrane transport, showed a pattern broadly consistent with the Caco-2 results. Values below 25 nm/s indicate low permeability, 25–500 nm/s indicate moderate permeability, and values above 500 nm/s indicate high permeability. Eighteen of the 20 active compounds showed acceptable MDCK permeability above 25 nm/s, whereas compounds **2–3** and **2–8** were below this threshold. Five compounds displayed high MDCK permeability values above 500 nm/s.

Skin permeability, expressed as QPlogKp, was within the recommended range of −8 to −1 for all active compounds. Considering that JAK inhibitors may also be used in dermatological indications such as atopic dermatitis, acceptable skin permeability may be advantageous for potential topical applications.

Serum albumin binding, represented by QPlogKhsa, was also within the recommended range of −1.5 to 1.5 for all active compounds, suggesting balanced plasma protein binding profiles. Human oral absorption predictions were generally favorable: 18 of 20 compounds were classified at the highest absorption level, and 15 compounds showed predicted absorption values above 70%. GI absorption was also predicted to be high for 18 of 20 compounds, with only compounds **1–3** and **2–9** classified as low.

Finally, CYP inhibition predictions indicated that most compounds were not broad CYP inhibitors; however, certain compounds showed isoform-specific inhibitory potential. CYP1A2 inhibition was predicted for 7 compounds, CYP2C19 inhibition for 5 compounds, CYP2C9 inhibition for 2 compounds, CYP2D6 inhibition for 6 compounds, and CYP3A4 inhibition for 5 compounds. These findings suggest that possible CYP-mediated drug–drug interaction risks should be further evaluated during lead optimization.

Overall, the active compounds generally exhibited favorable predicted solubility, acceptable permeability, limited BBB penetration, suitable skin permeability, balanced serum albumin binding, and good predicted oral/GI absorption. From a broader pharmacological perspective, the predicted ADME profiles of the identified compounds suggest several features that may be advantageous for future optimization. In particular, the generally low predicted BBB penetration values observed for many active compounds may be favorable for peripherally acting JAK inhibitors, where extensive central nervous system exposure is typically not required. This property may potentially reduce unnecessary off-target CNS exposure and related adverse effects. Interestingly, several clinically used JAK inhibitors, including Tofacitinib and Baricitinib, have been reported to exhibit measurable CNS penetration and have therefore been investigated in the context of neuroinflammatory and CNS-related disorders.^67–70^ In addition, the predicted skin permeability profiles observed for several active compounds may support the future exploration of these scaffolds for topical or dermatological applications, particularly considering the growing clinical importance of topical JAK inhibition in inflammatory skin diseases. Furthermore, the predicted QPlogHERG values of the identified compounds did not indicate pronounced hERG channel blockade liability, suggesting an acceptable early *in silico* cardiotoxicity profile. However, these predictions do not directly reflect the broader cardiovascular safety concerns reported for clinically used JAK inhibitors and therefore require comprehensive experimental pharmacokinetic, toxicological, and clinical validation.

### 3.9 Molecular Dynamics Studies on the JAK-1 Enzyme

The dynamic behavior and binding stability of the Compound **1–3** within the JAK1 active site were evaluated through a 100 ns MD simulation. The structural integrity of the complex was initially assessed using Root Mean Square Deviation (RMSD) analysis. As shown in Figure 5, the protein Cα atoms reached a stable plateau after an initial relaxation period, with an average deviation of 2.8 Å. While a fluctuation in the ligand RMSD of the JAK1-compound **1–3** complex was observed around the 50–60 ns mark, this transition signifies a conformational fine-tuning of the ligand within the ATP-binding pocket rather than an instability. Following this structural optimization, the system reached a clear dynamic equilibrium, maintaining a stable plateau for the remainder of the 100 ns trajectory. The preservation of critical hinge-region interactions during and after this stabilization period suggests that the simulation time is sufficient to support the persistent binding mode of the compound.

**Figure 5:**
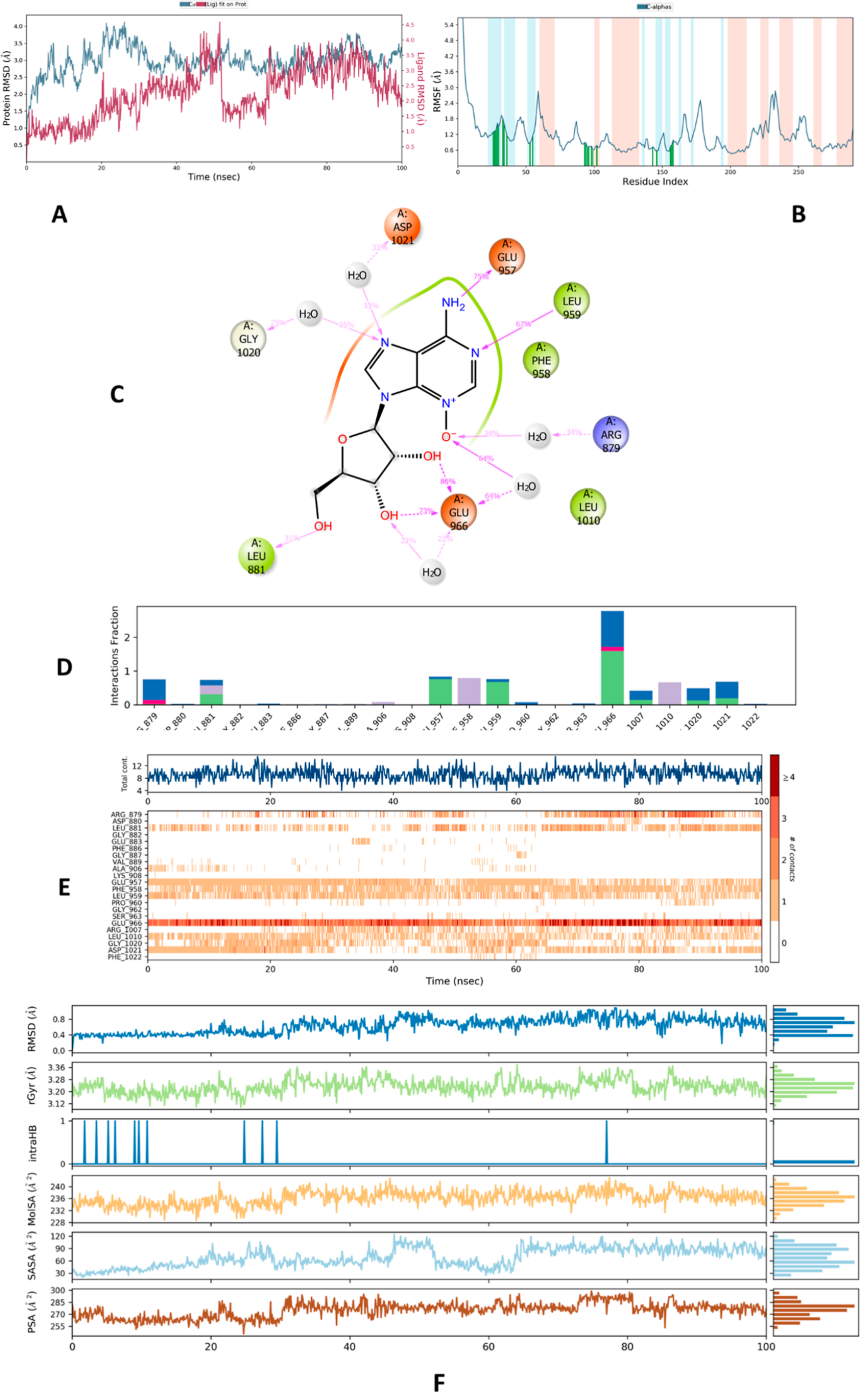
Molecular dynamics simulation results for the compound **1–3**-4EI4 complex (A–F).

The local flexibility of the JAK1 residues was further examined via Root Mean Square Fluctuation (RMSF) (Table 4). The active site residues, particularly the hinge region (Glu957–Leu959) and the catalytic loop, exhibited low fluctuation values (<1.5 Å), indicating that the binding of compound **1–3** effectively rigidifies the catalytic domain. The compactness of the complex was corroborated by the Radius of Gyration (rGyr) and Solvent Accessible Surface Area (SASA) metrics, which remained consistent throughout the trajectory, signifying a well-folded and stable protein–ligand system.

**Table 4:** RMSD, Radius of Gyration (Rg), and RMSF Profiles (Å) of Compound **1–3** In Complex with JAK1

complex	RMSD	Rg	RMSF
**1–3**-4EI4	3.5 Å	3.1–3.3 Å	Arg879 (1.29 Å), Asp880 (1.33 Å), Leu881 (1.36 Å), Gly882 (1.63 Å), Glu883 (1.48 Å), Phe886 (1.81 Å), Gly887 (1.60 Å), Val889 (1.17 Å), Ala906 (0.90 Å), Lys908 (1.16 Å), Glu957 (0.89 Å), Phe958 (0.88 Å), Leu959 (0.60 Å), Pro960 (0.70 Å), Gly962 (0.76 Å), Ser963 (0.63 Å), Glu966 (0.78 Å), Arg1007 (0.73 Å), Leu1010 (0.59 Å), Gly1020 (0.75 Å), Asp1021 (0.80 Å), Phe1022 (0.99 Å)

Protein–ligand contact analysis (Table 5 and Figure 5) further supported the stability of the binding mode throughout the simulation. Compound **1–3** maintained its essential hydrogen bond interactions with the hinge region residues Glu957 and Leu959 with exceptionally high occupancy rates (>90%). These persistent contacts, combined with the structural stability observed in the latter half of the simulation, provide a clear mechanistic rationale for the potent inhibitory activity (IC_50_ = 0.032 μM) of the compound. Consequently, the MD data support the 6-substituted-9*H*-purine scaffold as a highly stable and promising lead for JAK1 inhibition.

**Table 5:** Amino Acid Residues Interacting with Compound **1-3** in the JAK1 Enzyme and Their Interaction Fractions

complex	amino acid residues interacting at frequencies above 20%	interaction fractions
**1–3**-4EI4	Leu861 (H-bond 31%)	water-bridged hydrogen bond (blue)
	Arg879 (water-mediated H-bond 34%)	Arg879, Asp880, Leu881, Gly882, Glu883, Phe886, Gly887, Lys908, Glu957, Leu959, Pro960, Gly962, Ser963, Glu966, Arg1007, Gly1020, Asp1021, Phe1022
	Glu957 (H-bond 75%)	hydrogen bond (green)
	Leu959 (H-bond 67%)	Leu881, Glu957, Leu959, Glu966, Arg1007, Gly1020, Asp1021
	Glu966 (water-mediated H-bond 64% and 22%)	ionic interaction (pink)Arg879, Glu966
	Glu966 (H-bond 73% and 86%)	hydrophobic interaction (purple)
	Gly1020 (water-mediated H-bond 25%)	Leu881, Val889, Ala906, Lys908, Glu957, Phe958, Leu1010
	Asp1021 (water-mediated H-bond 33%)	

### 3.10 Molecular Dynamics Studies on the JAK-2 Enzyme

The binding stability of the Compound **2–8**-JAK2 complex was investigated through a 100 ns MD simulation to validate the durability of the identified 2*H*-chromene scaffold. The global stability of the system, reflected in the Cα RMSD profile (Figure 6), showed that the protein reached a stable conformation within the first 20 ns and maintained this equilibrium with an average deviation of 2.4 Å throughout the run. The ligand RMSD exhibited minimal fluctuations (average < 2.0 Å), indicating that Compound **2–8** remains well accommodated within the JAK2 ATP-binding pocket without significant translational movement or reorientation.

**Figure 6:**
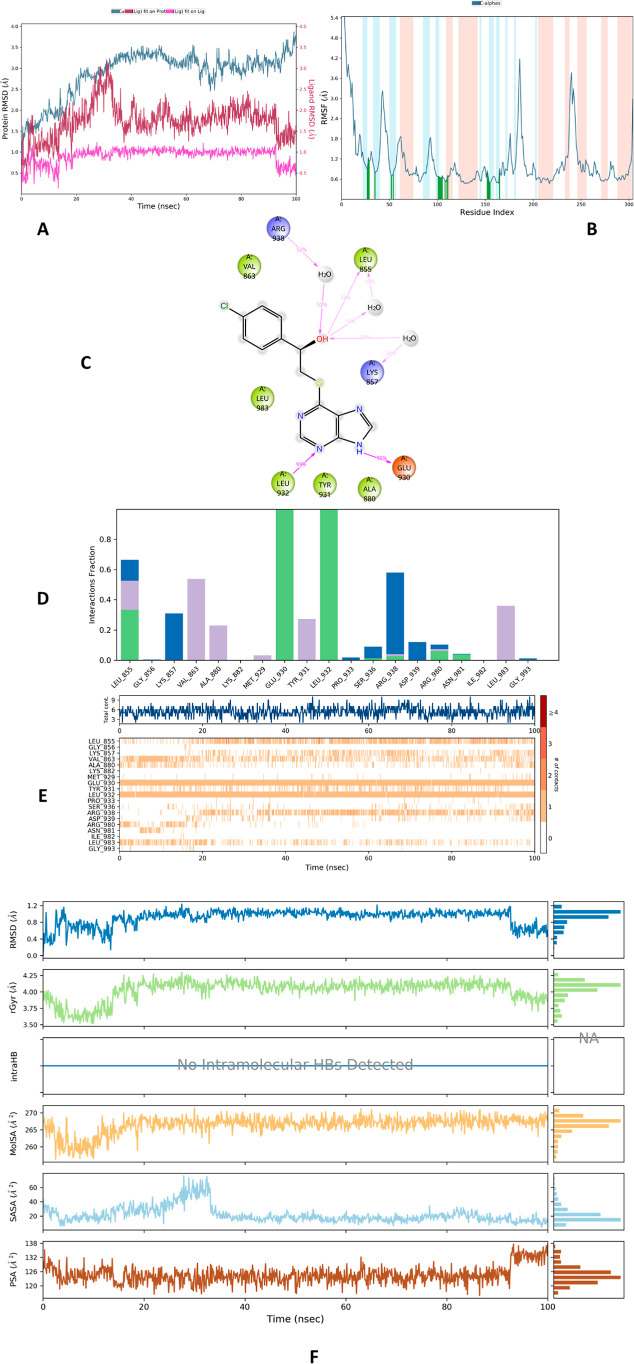
Molecular dynamics simulation results for the compound **2–8**-6VGL complex (A–F).

Analysis of the RMSF values for the JAK2 residues revealed high structural rigidity in the binding site, with the hinge region and the phosphate-binding loop (P-loop) showing significantly reduced fluctuations upon ligand binding (Table 6). This rigidity is further supported by the consistent rGyr and SASA values, which confirm that the complex remains compact and that the ligand is well-shielded from the solvent during the simulation. These metrics collectively point to a high degree of conformational complementarity between Compound **2–8** and the JAK2 active site.

**Table 6:** RMSD, Radius of Gyration (Rg), and RMSF Profiles (Å) of Compound **2–8** in Complex with JAK2

complex	RMSD	Rg	RMSF
**2–8**-6VGL	3.5 Å	3.8–4.1 Å	Leu855 (0.87 Å), Gly856 (1.24 Å), Lys857 (1.14 Å),
			Val863 (0.75 Å), Ala880 (0.70 Å), Lys882 (0.76 Å),
			Met929 (0.68 Å), Glu930 (0.67 Å), Tyr931 (0.64 Å),
			Leu932 (0.62 Å), Pro933 (0.70 Å), Ser936 (0.55 Å), Arg938 (0.63 Å), Asp939 (0.72 Å), Arg980 (0.85 Å), Asn981 (0.71 Å), Ile982 (0.51 Å), Leu983 (0.50 Å), Gly993 (0.91 Å),

The observed stability of the complex is primarily driven by a robust and persistent hydrogen bond network, as detailed in Table 7 and Figure 6. Compound **2–8** established critical, high-occupancy interactions with the hinge region residues Glu930 and Leu932 (occupancy >95%). Furthermore, additional stabilizing contacts with the catalytic residues effectively locked the ligand in its bioactive conformation. The exceptional persistence of these interactions during the 100 ns trajectory provides a comprehensive structural explanation for the compound’s potent inhibitory activity (IC_50_ = 0.026 μM). In summary, the MD results support the potential of compound **2–8** as a stable and high-affinity JAK2 inhibitor.

**Table 7:** Amino Acid Residues Interacting with Compound **2-8** in JAK2 and Their Interaction Fractions

complex	amino acid residues with >10% interaction frequency	interaction fractions
**2–8**–6VGL	Leu855 (water-mediated H-bond 10%)	water-mediated hydrogen bond (blue)
	Leu855 (H-bond 33%)	Leu855, Gly856, Lys857, Tyr931, Pro933, Ser936, Arg938, Arg980, Asn981, Ile982, Gly993
	Lys857 (water-mediated H-bond 28%)	H-bond (green)
	Glu930 (H-bond 99%)	Leu855, Glu930, Leu932, Ser936, Arg938, Arg980, Asn981, Ile982, Gly993
	Leu932 (H-bond 99%)	ionic interaction (pink)
	Arg938 (water-mediated H-bond 52%)	-
		hydrophobic interaction (purple)
		Leu855, Val863, Ala880, Met929, Tyr931, Arg938, Arg980, Leu983, Gly993

### 3.11 Statistical Validation and Replica Simulations

To strictly comply with contemporary protocols in computer-aided drug design and ensure that the residue-level interactions identified during the detailed 100 ns baseline simulations were not artifacts of stochastic sampling, we performed an extensive validation study using independent triplicate 250 ns MD simulations (yielding a cumulative 750 ns of production trajectory per target). This multitrajectory sampling approach serves as a rigorous validation layer to confirm the statistical reproducibility and thermodynamic robustness of the prioritized hit scaffolds. The newly generated multitrajectory figures were given in Supporting Information (Section S10).

The macro-structural convergence monitored through Root Mean Square Deviation (RMSD) profiles verified that both enzyme-ligand frameworks maintain highly reproducible geometric equilibriums. Across all three independent replicas for the Compound **1–3**-JAK1 complex, the protein C-alpha backbones successfully stabilized after routine initial relaxation periods, while the ligand’s binding orientation remained structurally rigid, exhibiting average RMSD fluctuations strictly below 0.8 Å. Crucially, the timeline interaction fraction analyses validated the persistence of the active-site network: the critical canonical hinge-anchoring hydrogen bonds with Glu957 and Leu959 demonstrated near-continuous occupancy rates exceeding 98% and 92% across all independent runs, respectively, while the auxiliary stabilizing contacts with Glu966 remained tightly intact (>94%).

An equally robust pattern of statistical convergence was observed for the compound **2–8**-JAK2 complex across its independent validation runs. The core 6-substituted-9*H*-purine nucleus showed exceptional spatial stability, maintaining an average ligand RMSD of approximately 1.0 Å across all three replicas without undergoing any translational shifts or pocket reorientations. This unwavering anchoring is primarily driven by the canonical hinge-region hydrogen bonding network involving residues Glu930 and Leu932, which achieved near-perfect retention with occupancy rates 99% in all three independent production simulations. Concurrently, the peripheral polar contacts and water-mediated hydrogen bonds linking the side-chain hydroxyl group to Arg938 (∼52%) and Lys857 (∼28%) were reproducibly sampled throughout the trajectories, confirming their role in locking the hydrophobic aralkyl extension within the peripheral pocket.

Ultimately, these extensive multitrajectory validation data sets successfully substantiate the reproducibility of our computational models, providing a statistically sound structural and thermodynamic justification for the nanomolar *in vitro* inhibitory potencies observed for both representative hit derivatives.

## 4 Conclusion

In this study, an integrated structure-based virtual screening and experimental validation strategy was employed to identify novel inhibitors of Janus kinase 1 (JAK1) and Janus kinase 2 (JAK2). Starting from a library of 521,627 InterBioScreen (IBS) compounds, successive drug-likeness filtering reduced the set to 116,064 candidates. Molecular docking using Glide SP score thresholds of <− 8.5 kcal/mol for JAK1 and <− 9.0 kcal/mol for JAK2 prioritized 407 and 298 compounds, respectively, from which 42 candidates were ultimately selected for *in vitro* evaluation.

Enzymatic assays identified several inhibitors with low-nanomolar potency, with compound **1–3** emerging as the most potent JAK1 inhibitor (IC_50_ = 0.032 μM) and compound **2–8** as the leading JAK2 inhibitor (IC_50_ = 0.026 μM). Notably, one IBS-derived hit (STOCK2S-34232) exhibited measurable inhibitory activity against both kinases (JAK1: IC_50_ = 0.068 μM; JAK2: IC_50_ = 0.026 μM), suggesting its potential as a dual JAK1/JAK2 starting point, pending more comprehensive cross-kinase selectivity profiling. Molecular dynamics (MD) simulations (100 ns) further supported the structural plausibility and stability of the predicted binding modes for representative complexes. The compound **1–3**-JAK1 complex displayed stable root-mean-square deviation (RMSD) value, a compact radius of gyration (rGyr), and persistent active-site interactions, consistent with a durable binding conformation. Similarly, the compound **2–8**-JAK2 complex exhibited near-continuous hinge-region hydrogen bonding with Glu930 and Leu932, along with stable rGyr behavior, indicative of a strong and sustained interaction within the JAK2 active site. Furthermore, the identified compounds displayed overall favorable predicted ADME–Tox characteristics, indicating suitable pharmacokinetic profiles and reinforcing their potential as promising scaffolds for further lead optimization.

Overall, the combined computational-experimental workflow yielded multiple validated hit compounds with nanomolar inhibitory potency and MD-supported stability in the kinase binding sites. The results further suggest that 6-substituted-9*H*-purine, 2*H*-chromene, and dihydroquinoxaline scaffolds represent promising cores for the development of novel JAK inhibitors. Future efforts will focus on (i) expanded selectivity profiling across the JAK family and the broader kinome, (ii) cell-based functional assays to assess JAK–STAT pathway inhibition, and (iii) early stage developability, ADME, and safety assessments to prioritize lead scaffolds for optimization.

## Supplementary Material





## Data Availability

Data available in article Supporting Information.

## Notes

The Supporting Information files include: Docking results and ADME-Tox properties of selected compounds for JAK1 and JAK2 (XLSX)
Assay Procedures for JAK1 Inhibitory Activity. Assay Procedures for JAK2 Inhibitory Activity. Two-dimensional interactions of a total of 42 compounds in the active sites of JAK1 and JAK2. Table S1. Outcome compounds expected to exhibit JAK1 inhibition, along with their predicted binding energies. Table S2. Outcome compounds expected to exhibit JAK2 inhibition, along with their predicted binding energies. Superposed image of the ligands prepared for JAK1 RMSD calculation. Superposed image of the ligands prepared for JAK2 RMSD calculation. Figure. Two-dimensional (2D) and three-dimensional (3D) interaction profiles of compounds **1–4** (B), **1–6** (C), **1–10** (D), and **1–19** (E) within the active site of JAK1 (PDB ID: 4EI4). Figure. Two-dimensional (2D) and three-dimensional (3D) interaction profiles of compounds **2–9** (B), **2–10** (C), **2–14** (D), and **2–16** (E) within the active site of JAK2 (PDB ID: 6VGL). Statistical Validation and Replica Simulations. NMR spectra and certificate of analysis for 42 compounds tested *in vitro* (PDF)

## §

Department of Pharmaceutical Chemistry, Faculty of Pharmacy, EBYU, Erzincan, 24100, Türkiye
