# Supplementary material for: Identification of Novel Small-Molecule Janus Kinase 1 and 2 Inhibitors Through Structure-Based Virtual Screening, In Vitro Assays, and Molecular Dynamics Simulations
Source: ACS Omega. 2026 Jul 25;11(31):46119–35. doi: 10.1021/acsomega.6c02611 (PMC13470719; doi:10.1021/acsomega.6c02611)
Supplement: Supplementary file 1 [file ao6c02611_si_002.pdf]

Identification of Novel Small-Molecule Janus Kinase 1 and 2 Inhibitors through Structure-Based Virtual Screening, *In Vitro* Assays, and Molecular Dynamics Simulations

Ahmet Avci[a], Hayrünnisa Taşci[a,‡], Begüm Nurpelin Sağlık Özkan[b], Birsen Tozkoparan[a], Nesrin Gökhan Kelekçi[a,*]

[a] Department of Pharmaceutical Chemistry, Faculty of Pharmacy, Hacettepe University, Ankara, 06100, Türkiye.

[b] Department of Pharmaceutical Chemistry, Faculty of Pharmacy, Anadolu University, Eskişehir, 26470, Türkiye.

[‡] Present Address: Department of Pharmaceutical Chemistry, Faculty of Pharmacy, EBYU, Erzincan, 24100, Türkiye.

*** Corresponding author**

Nesrin Gökhan Kelekçi

Department of Pharmaceutical Chemistry, Faculty of Pharmacy, Hacettepe University,
Ankara, 06100, Türkiye
Tel.: +903123053017; fax:+903123114777
e-mail: onesrin@hacettepe.edu.tr

Content

1. Assay Procedures for JAK1 Inhibitory Activity

2. Assay Procedures for JAK2 Inhibitory Activity

3. Two-dimensional interactions of a total of 42 compounds in the active sites of JAK1 and JAK2 (Figures S1-S44).

-**Figure S1.** Two-dimensional interaction of (1R,3R)-3-(2-metilimidazo[4,5-d]pirolo[2,3-b]piridin-1(8H)-il)siklohekzanol in the active site of JAK1.

- **Figure S2.** Two-dimensional interaction of STOCK1N-12057 in the active site of JAK1.

- **Figure S3.** Two-dimensional interaction of STOCK1N-31772 in the active site of JAK1.

- **Figure S4.** Two-dimensional interaction of STOCK1N-51560 in the active site of JAK1.

- **Figure S5.** Two-dimensional interaction of STOCK1N-69616 in the active site of JAK1.

- **Figure S6.** Two-dimensional interaction of STOCK1N-71510 in the active site of JAK1.

- **Figure S7.** Two-dimensional interaction of STOCK1N-77176 in the active site of JAK1.

- **Figure S8.** Two-dimensional interaction of STOCK1N-79356 in the active site of JAK1.

- **Figure S9.** Two-dimensional interaction of STOCK1N-97980 in the active site of JAK1.

- **Figure S10.** Two-dimensional interaction of STOCK2S-29689 in the active site of JAK1.

- **Figure S11.** Two-dimensional interaction of STOCK2S-34232 in the active site of JAK1.

- **Figure S12.** Two-dimensional interaction of STOCK4S-15629 in the active site of JAK1.

- **Figure S13.** Two-dimensional interaction of STOCK4S-46885 in the active site of JAK1.

- **Figure S14.** Two-dimensional interaction of STOCK4S-70675 in the active site of JAK1.

- **Figure S15.** Two-dimensional interaction of STOCK5S-74239 in the active site of JAK1.

- **Figure S16.** Two-dimensional interaction of STOCK5S-83560 in the active site of JAK1.

- **Figure S17.** Two-dimensional interaction of STOCK6S-28538 in the active site of JAK1.

- **Figure S18.** Two-dimensional interaction of STOCK6S-72898 in the active site of JAK1.

- **Figure S19.** Two-dimensional interaction of STOCK6S-83203 in the active site of JAK1.

- **Figure S20.** Two-dimensional interaction of STOCK7S-44229 in the active site of JAK1.

- **Figure S21.** Two-dimensional interaction of STOCK7S-51860 in the active site of JAK1.

- **Figure S22.** Two-dimensional interaction of STOCK7S-53671 in the active site of JAK1.

- **Figure S23.** Two-dimensional interaction of Ruxolutinib in the active site of JAK2.

- **Figure S24.** Two-dimensional interaction of STOCK1S-00944 in the active site of JAK2.

- **Figure S25.** Two-dimensional interaction of STOCK1S-06278 in the active site of JAK2.

- **Figure S26.** Two-dimensional interaction of STOCK1S-10380 in the active site of JAK2.

- **Figure S27.** Two-dimensional interaction of STOCK1S-69008 in the active site of JAK2.

- **Figure S28.** Two-dimensional interaction of STOCK1S-78591 in the active site of JAK2.

- **Figure S29.** Two-dimensional interaction of STOCK2S-08448 in the active site of JAK2.

- **Figure S30.** Two-dimensional interaction of STOCK2S-18844 in the active site of JAK2.

- **Figure S31.** Two-dimensional interaction of STOCK2S-34232 in the active site of JAK2.

- **Figure S32.** Two-dimensional interaction of STOCK2S-52524 in the active site of JAK2.

- **Figure S33.** Two-dimensional interaction of STOCK2S-54227 in the active site of JAK2.

- **Figure S34.** Two-dimensional interaction of STOCK2S-60777 in the active site of JAK2.

- **Figure S35.** Two-dimensional interaction of STOCK2S-75169 in the active site of JAK2.
- **Figure S36.** Two-dimensional interaction of STOCK3S-18016 in the active site of JAK2.
- **Figure S37.** Two-dimensional interaction of STOCK3S-18468 in the active site of JAK2.
- **Figure S38.** Two-dimensional interaction of STOCK3S-83435 in the active site of JAK2.
- **Figure S39.** Two-dimensional interaction of STOCK3S-84679 in the active site of JAK2.
- **Figure S40.** Two-dimensional interaction of STOCK4S-62175 in the active site of JAK2.
- **Figure S41.** Two-dimensional interaction of STOCK4S-86355 in the active site of JAK2.
- **Figure S42.** Two-dimensional interaction of STOCK5S-56194 in the active site of JAK2.
- **Figure S43.** Two-dimensional interaction of STOCK5S-60009 in the active site of JAK2.
- **Figure S44.** Two-dimensional interaction of STOCK5S-79464 in the active site of JAK2.

4. Table S1. Outcome compounds expected to exhibit JAK1 inhibition, along with their predicted binding energies.

5. Table S2. Outcome compounds expected to exhibit JAK2 inhibition, along with their predicted binding energies.

6. Superposed image of the ligands prepared for JAK1 RMSD calculation (Figure S45).

7. Superposed image of the ligands prepared for JAK2 RMSD calculation (Figure S46).

8. Two-dimensional (2D) and three-dimensional (3D) interaction profiles of compounds 1–4 (B), 1–6 (C), 1–10 (D), and 1–19 (E) within the active site of JAK1 (PDB ID: 4EI4) (Figure S47).

9. Figure. Two-dimensional (2D) and three-dimensional (3D) interaction profiles of compounds 2–9 (B), 2–10 (C), 2–14 (D), and 2–16 (E) within the active site of JAK2 (PDB ID: 6VGL) (Figure S48).

10. Statistical Validation and Replica Simulations (Figure S49-60).

-**Figure S49:** The RMSD plots of compound **1-3** with the JAK1 protein as a function of simulation time (0 to 250 ns) captured across independent triplicate production runs (Replica 1, Replica 2, and Replica 3) in complex with JAK1 (PDB ID: 4EI4).

-**Figure S50:** The RMSF profiles compound **1-3** with the JAK1 protein captured across independent triplicate production runs (Replica 1, Replica 2, and Replica 3) in complex with JAK1 (PDB ID: 4EI4).

-Figure S51: Detailed two-dimensional (2D) ligand-protein interaction diagrams of compound **1-3** with the JAK1 protein captured across independent triplicate production runs (Replica 1, Replica 2, and Replica 3) in complex with JAK1 (PDB ID: 4EI4).

-Figure S52: Normalized protein-ligand interaction fractions of compound **1-3** with the JAK1 protein captured across independent triplicate production runs (Replica 1, Replica 2, and Replica 3) in complex with JAK1 (PDB ID: 4EI4).

-Figure S53: Timeline representation of compound **1-3** with the JAK1 protein captured across independent triplicate production runs (Replica 1, Replica 2, and Replica 3) in complex with JAK1 (PDB ID: 4EI4).

-Figure S54: Time-dependent physicochemical properties of compound **1-3** with the JAK1 protein captured across independent triplicate production runs (Replica 1, Replica 2, and Replica 3) in complex with JAK1 (PDB ID: 4EI4).

-Figure S55: The RMSD plots of compound **2-8** with the JAK2 protein as a function of simulation time (0 to 250 ns) captured across independent triplicate production runs (Replica 1, Replica 2, and Replica 3) in complex with JAK2 (PDB ID: 6VGL).

-Figure S56: The RMSF profiles compound **2-8** with the JAK2 protein captured across independent triplicate production runs (Replica 1, Replica 2, and Replica 3) in complex with JAK2 (PDB ID: 6VGL).

-Figure S57: Detailed two-dimensional (2D) ligand-protein interaction diagrams of compound **2-8** with the JAK2 protein captured across independent triplicate production runs (Replica 1, Replica 2, and Replica 3) in complex with JAK2 (PDB ID: 6VGL).

-Figure S58: Normalized protein-ligand interaction fractions of compound **2-8** with the JAK2 protein captured across independent triplicate production runs (Replica 1, Replica 2, and Replica 3) in complex with JAK2 (PDB ID: 6VGL).

-Figure S59: Timeline representation of compound **2-8** with the JAK2 protein captured across independent triplicate production runs (Replica 1, Replica 2, and Replica 3) in complex with JAK2 (PDB ID: 6VGL).

-Figure S60: Time-dependent physicochemical properties of compound **2-8** with the JAK2 protein captured across independent triplicate production runs (Replica 1, Replica 2, and Replica 3) in complex with JAK2 (PDB ID: 6VGL).

11. NMR spectra and certificate of analysis for 42 compounds tested *in vitro*.

-**Figure S61.** 1H-NMR spectrum of STOCK1N-12057

-**Figure S62.** 1H-NMR spectrum of STOCK1N-31772

-Figure S63. 1H-NMR spectrum of STOCK1N-51560

-Figure S64. 1H-NMR spectrum of STOCK1N-69616

-Figure S65. 1H-NMR spectrum of STOCK1N-71510

-Figure S66. 1H-NMR spectrum of STOCK1N-77176

-Figure S67. 1H-NMR spectrum of STOCK1N-79356

-Figure S68. 1H-NMR spectrum of STOCK1N-97980

-Figure S69. 1H-NMR spectrum of STOCK1S-06278

-Figure S70. 1H-NMR spectrum of STOCK1S-69008

-Figure S71. 1H-NMR spectrum of STOCK2S-08448

-Figure S72. 1H-NMR spectrum of STOCK2S-18844

-Figure S73. 1H-NMR spectrum of STOCK2S-29689

-Figure S74. 1H-NMR spectrum of STOCK2S-34232

-Figure S75. 1H-NMR spectrum of STOCK2S-52524

-Figure S76. 1H-NMR spectrum of STOCK2S-54227

-Figure S77. 1H-NMR spectrum of STOCK2S-60777

-Figure S78. 1H-NMR spectrum of STOCK2S-75169

-Figure S79. 1H-NMR spectrum of STOCK3S-18016

-Figure S80. 1H-NMR spectrum of STOCK3S-18468

-Figure S81. 1H-NMR spectrum of STOCK3S-83435

-Figure S82. 1H-NMR spectrum of STOCK3S-84679

-Figure S83. 1H-NMR spectrum of STOCK4S-15629

-Figure S84. 1H-NMR spectrum of STOCK4S-33920

-Figure S85. 1H-NMR spectrum of STOCK4S-46885

-Figure S86. 1H-NMR spectrum of STOCK4S-62175

-Figure S87. 1H-NMR spectrum of STOCK4S-70675

-Figure S88. 1H-NMR spectrum of STOCK4S-86355

-Figure S89. 1H-NMR spectrum of STOCK5S-56194

-Figure S90. 1H-NMR spectrum of STOCK5S-60009

-Figure S91. 1H-NMR spectrum of STOCK5S-74239

-Figure S92. 1H-NMR spectrum of STOCK5S-79464

-Figure S93. 1H-NMR spectrum of STOCK5S-83560

-Figure S94. 1H-NMR spectrum of STOCK6S-28538

-Figure S95. 1H-NMR spectrum of STOCK6S-83203

-Figure S96. 1H-NMR spectrum of STOCK7S-44229

-Figure S97. 1H-NMR spectrum of STOCK7S-51860

-Figure S98. 1H-NMR spectrum of STOCK7S-53671

1. **Assay Procedures for JAK1 Inhibitory Activity**

The JAK1 inhibitory activity was evaluated using the JAK1 (Janus Kinase 1) Assay Kit according to the manufacturer's instructions. Briefly, a master mix was prepared by combining 5x Kinase Assay Buffer 1 (supplemented with 10 mM DTT), 500 µM ATP, 10X IRS1-tide substrate, and distilled water. Each well was charged with 25 µL of the master mix, followed by the addition of 5 µL of the test inhibitor solution at varying concentrations. For the positive control and blank wells, 5 µL of the inhibitor buffer was added.

The JAK1 enzyme was thawed on ice and diluted to a final concentration of approximately 5 ng/µL using 1× Kinase Assay Buffer 1. The enzymatic reaction was initiated by adding 20 µL of the diluted JAK1 enzyme to the positive control and test inhibitor wells, while the blank wells received 20 µL of 1× Kinase Assay Buffer 1. Following a 45-minute incubation at 30 °C, 50 µL of Kinase-Glo Max reagent was added to each well. The plate was then protected from light with aluminum foil and incubated at room temperature for 15 minutes. Luminescence was measured using a microplate reader to determine the kinase activity.

2. **Assay Procedures for JAK2 Inhibitory Activity**

The JAK2 inhibitory screening was conducted following the protocol established in the JAK2 (Janus Kinase 2) Assay Kit. A master mix was prepared by mixing 5× Kinase Assay Buffer 1 (enriched with 10 mM DTT), 500 µM ATP, Poly(Glu:Tyr 4:1) PTK substrate (10 mg/mL), and distilled water. Each reaction well was filled with 25 µL of the master mix, to which 5 µL of the test inhibitor solution was added. For the positive control and blank wells, 5 µL of inhibitor-free buffer was used.

The JAK2 enzyme was thawed on ice and subsequently diluted to a concentration of approximately 2.5 ng/µL using 1× Kinase Assay Buffer 1. The kinase reaction was initiated by adding 20 µL of the diluted JAK2 enzyme to the test inhibitor and positive control wells; the blank wells received an equal volume of 1× Kinase Assay Buffer 1. The mixture was incubated at 30 °C for 45 minutes. To quantify the activity, 50 µL of Kinase-Glo Max reagent was added to each well, and the plate was incubated in the dark (covered with aluminum foil) at room temperature for 15 minutes. Luminescence was measured using a microplate reader.

3. **A total of 42 compounds two-dimensional interactions**
   JAK1

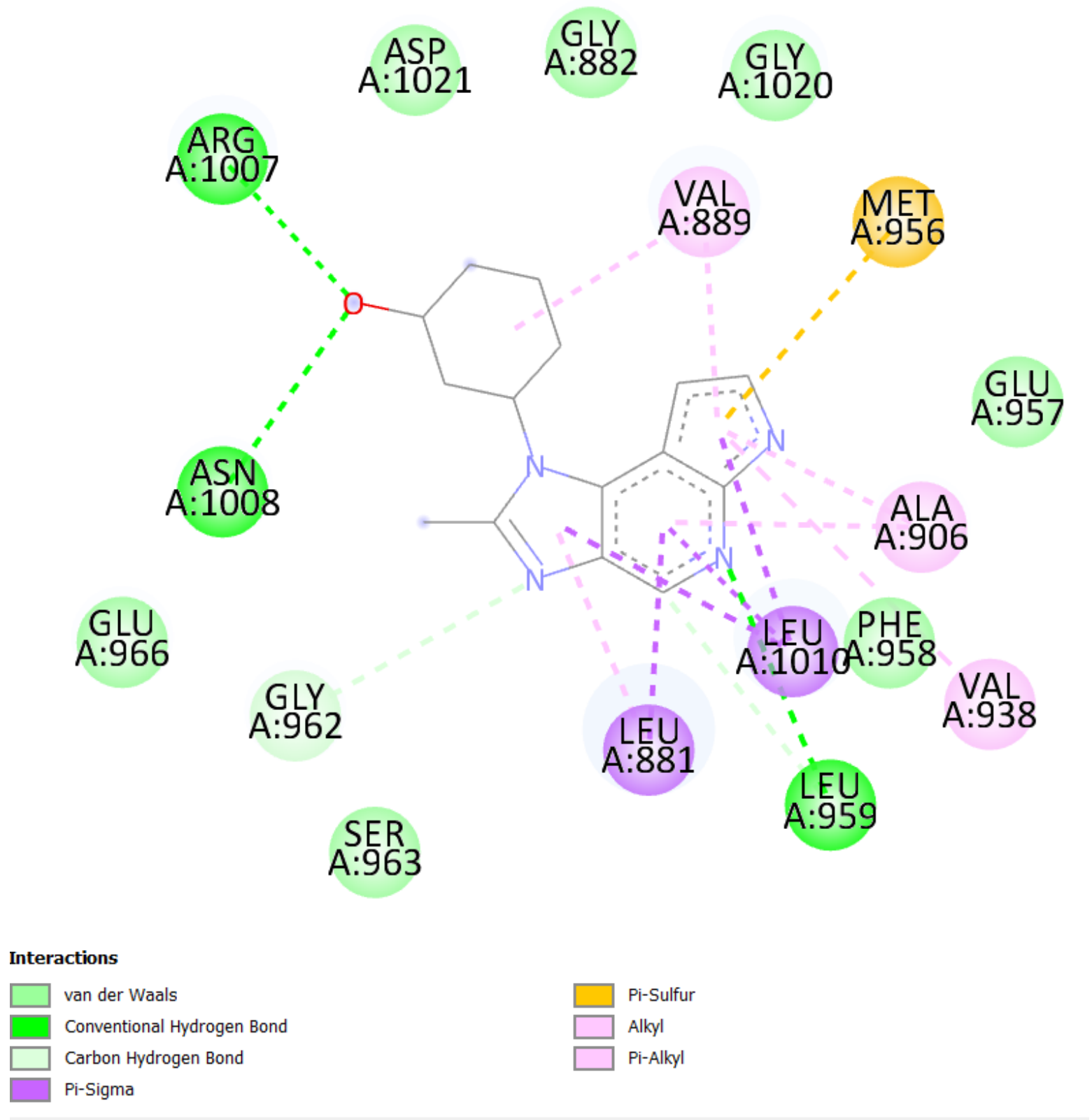

Figure S1. Two-dimensional interaction of (1R,3R)-3-(2-metilimidazo[4,5-d]pirolo[2,3-b]piridin-1(8H)-il)siklohekzanol in the active site of JAK1.

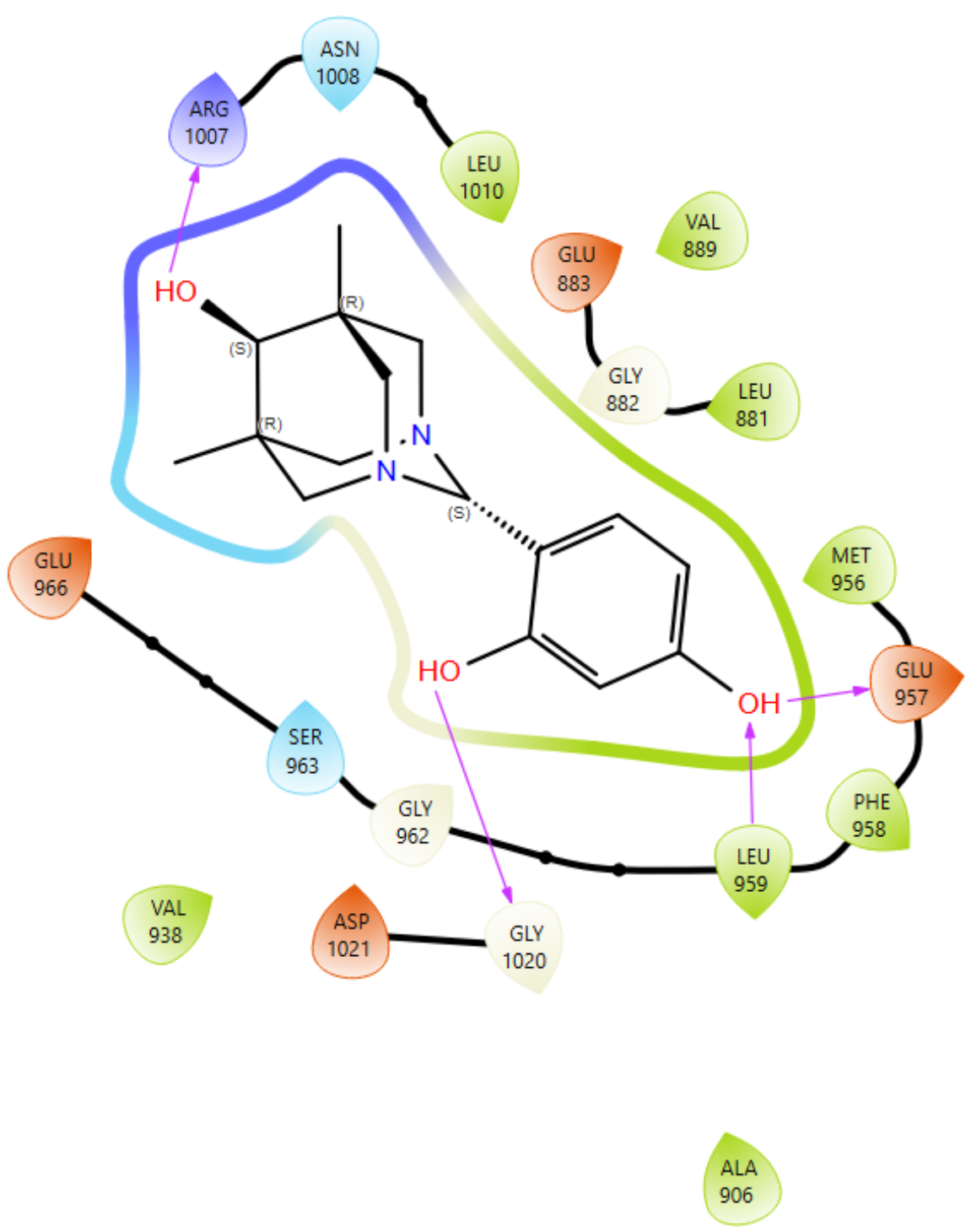

Figure S2. Two-dimensional interaction of STOCK1N-12057 in the active site of JAK1.

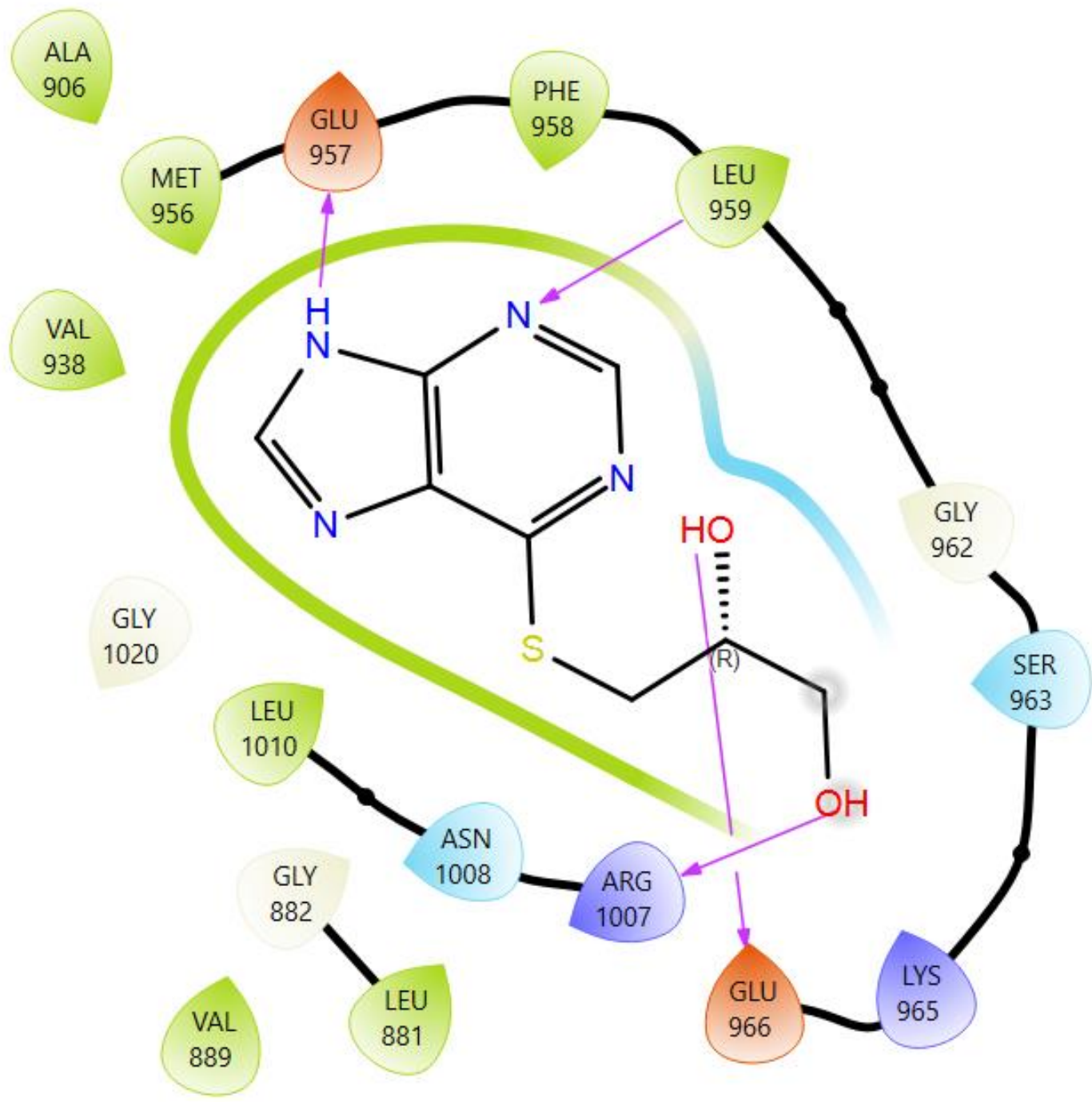

Figure S3. Two-dimensional interaction of STOCK1N-31772 in the active site of JAK1.

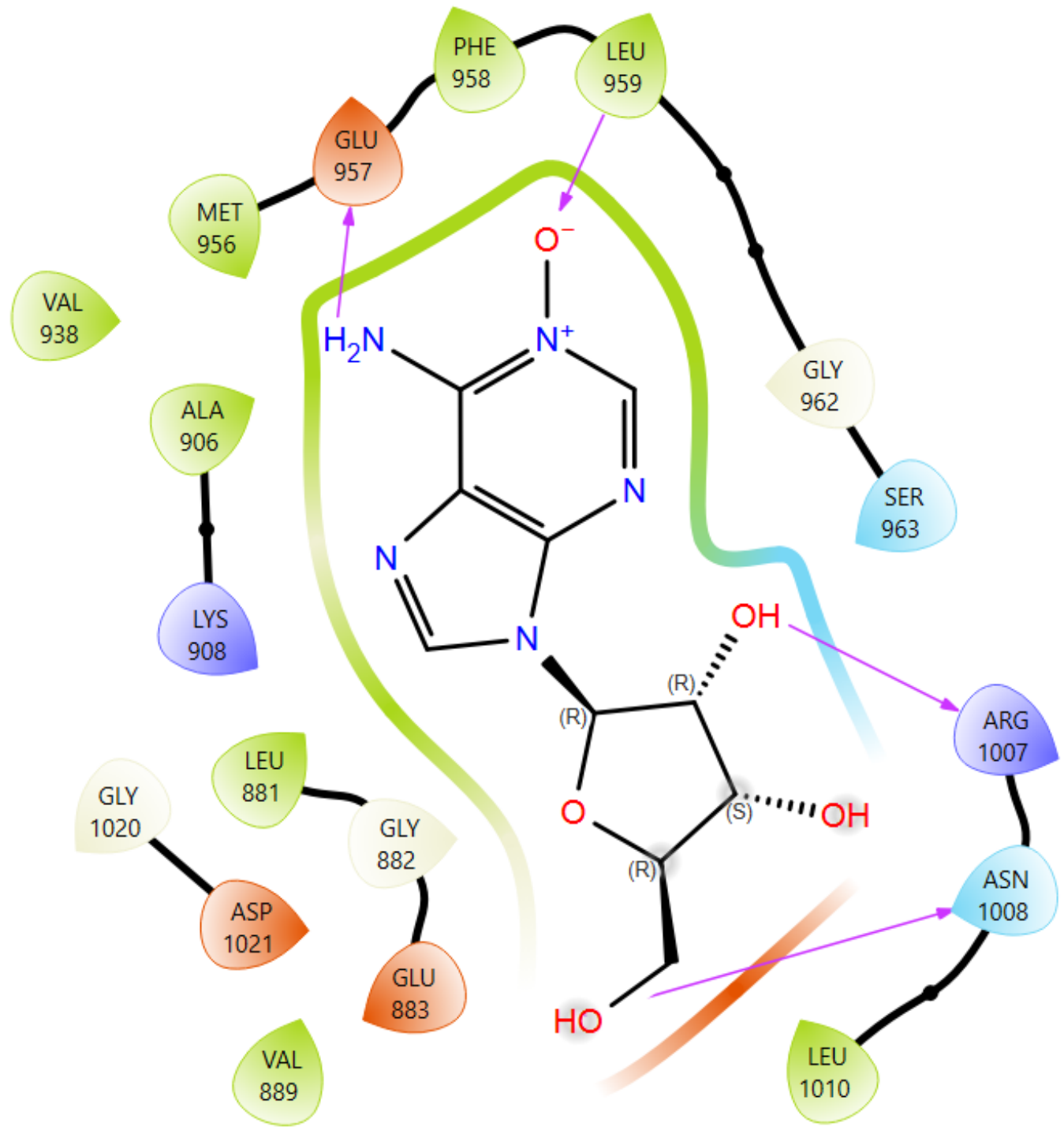

Figure S4. Two-dimensional interaction of STOCK1N-51560 in the active site of JAK1.

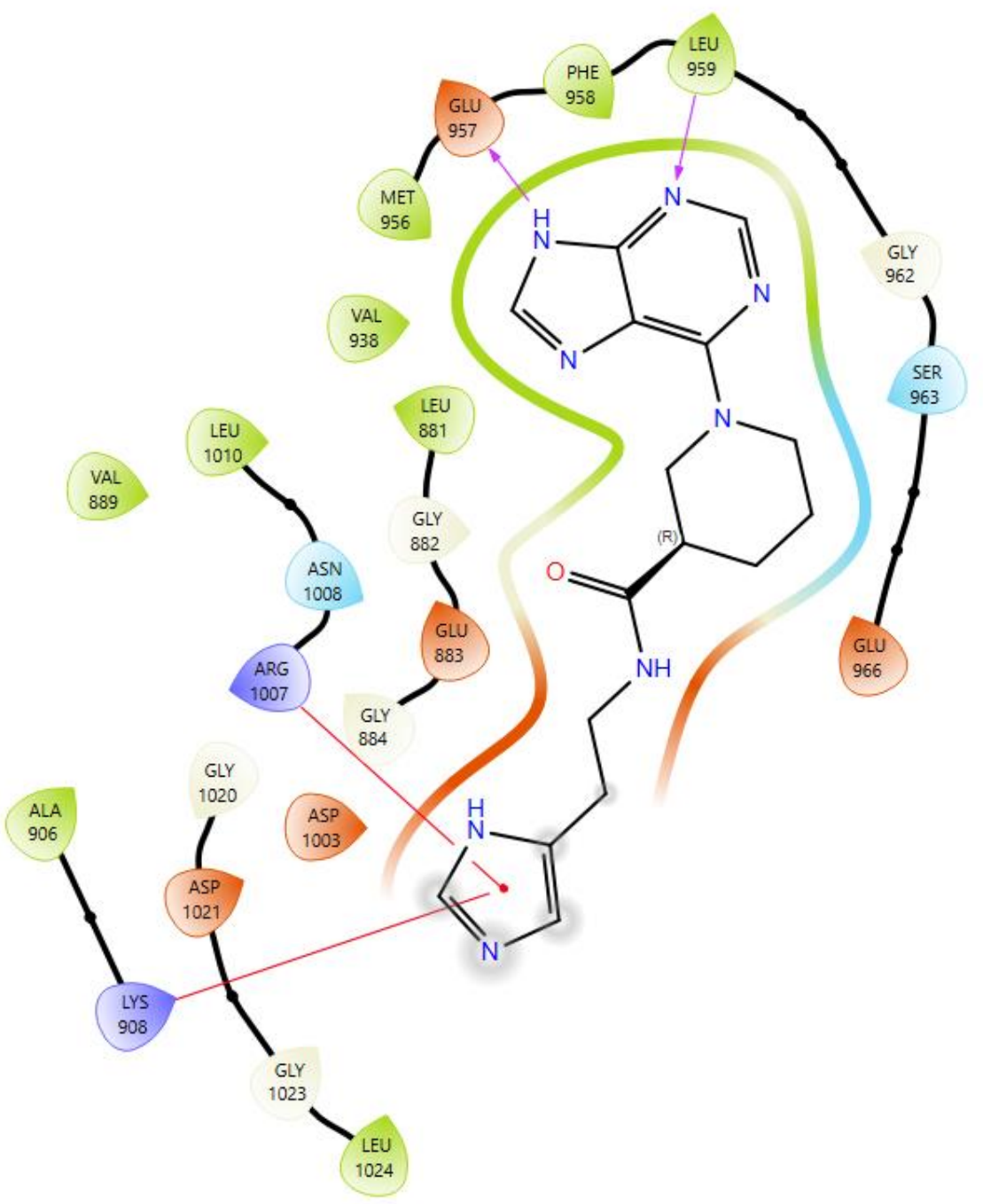

Figure S5. Two-dimensional interaction of STOCK1N-69616 in the active site of JAK1.

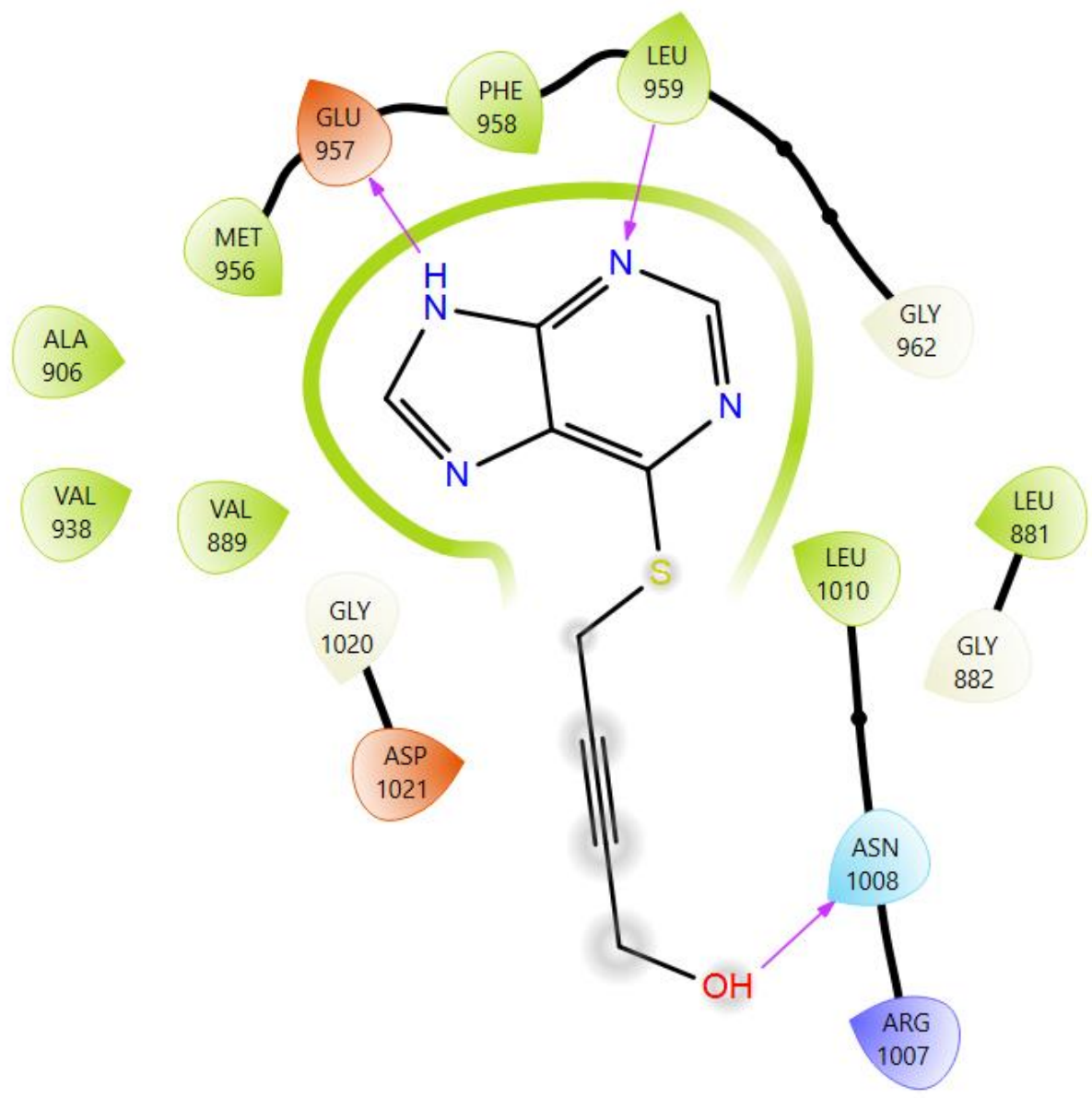

Figure S6. Two-dimensional interaction of STOCK1N-71510 in the active site of JAK1.

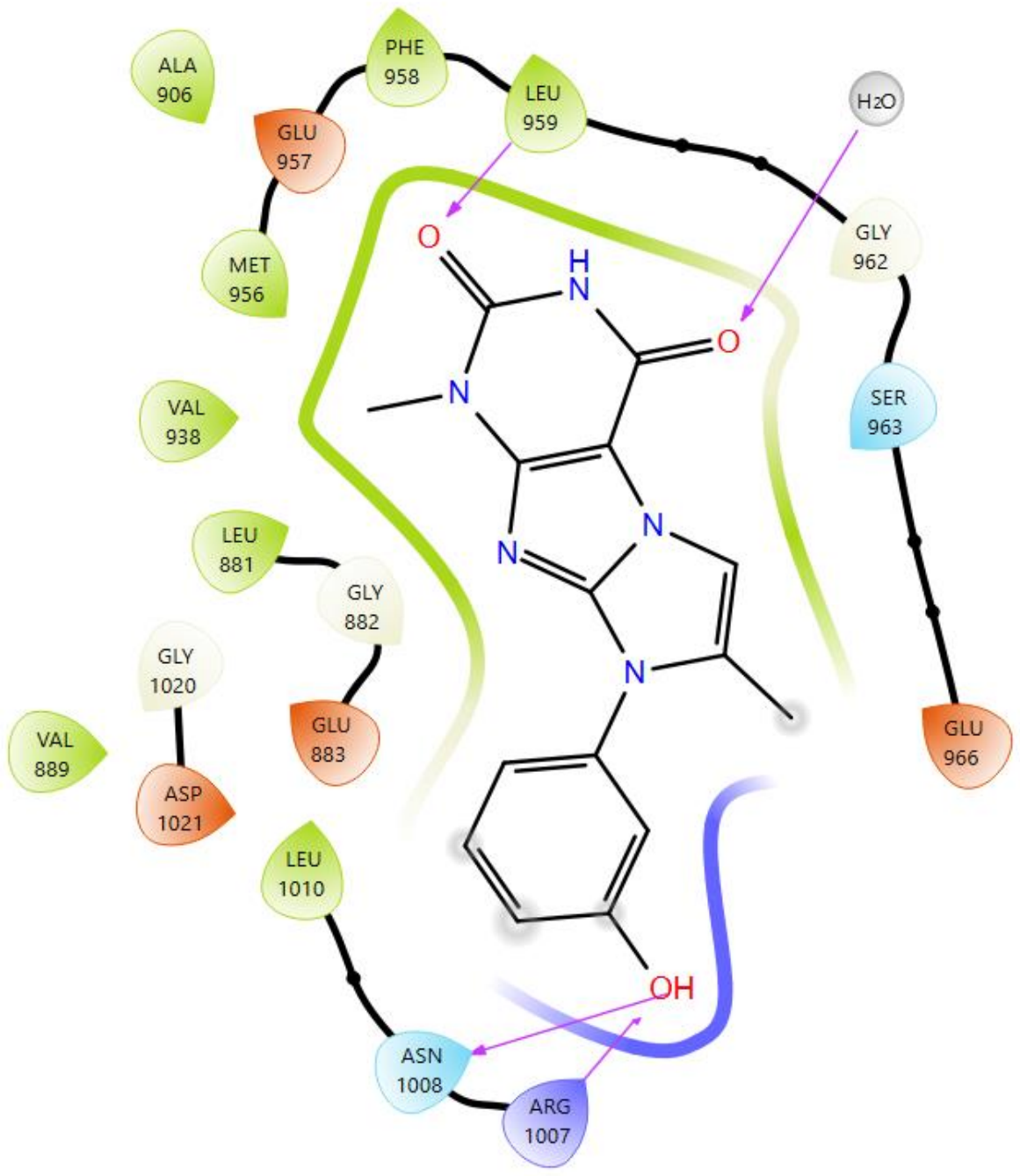

Figure S7. Two-dimensional interaction of STOCK1N-77176 in the active site of JAK1.

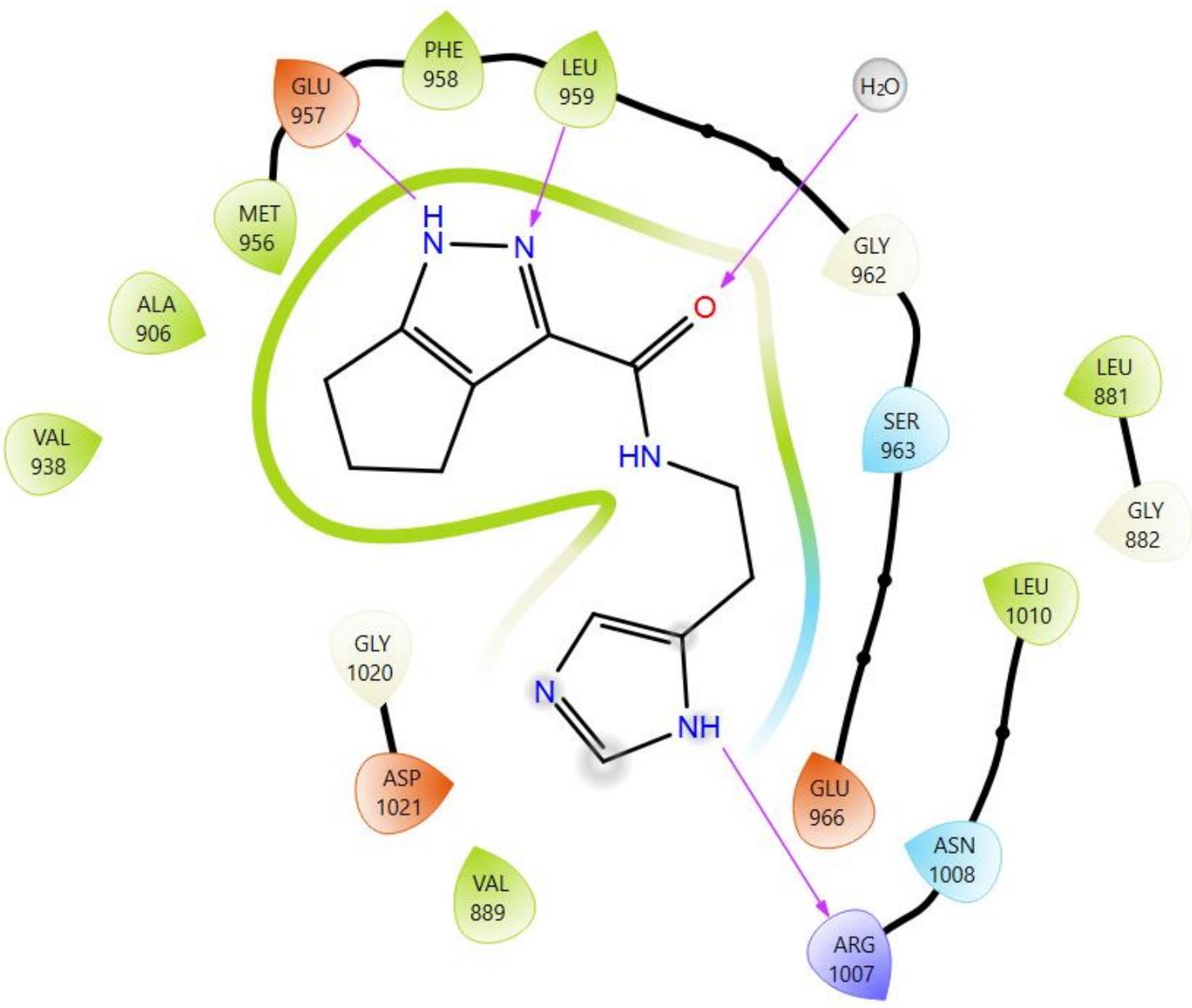

Figure S8. Two-dimensional interaction of STOCK1N-79356 in the active site of JAK1.

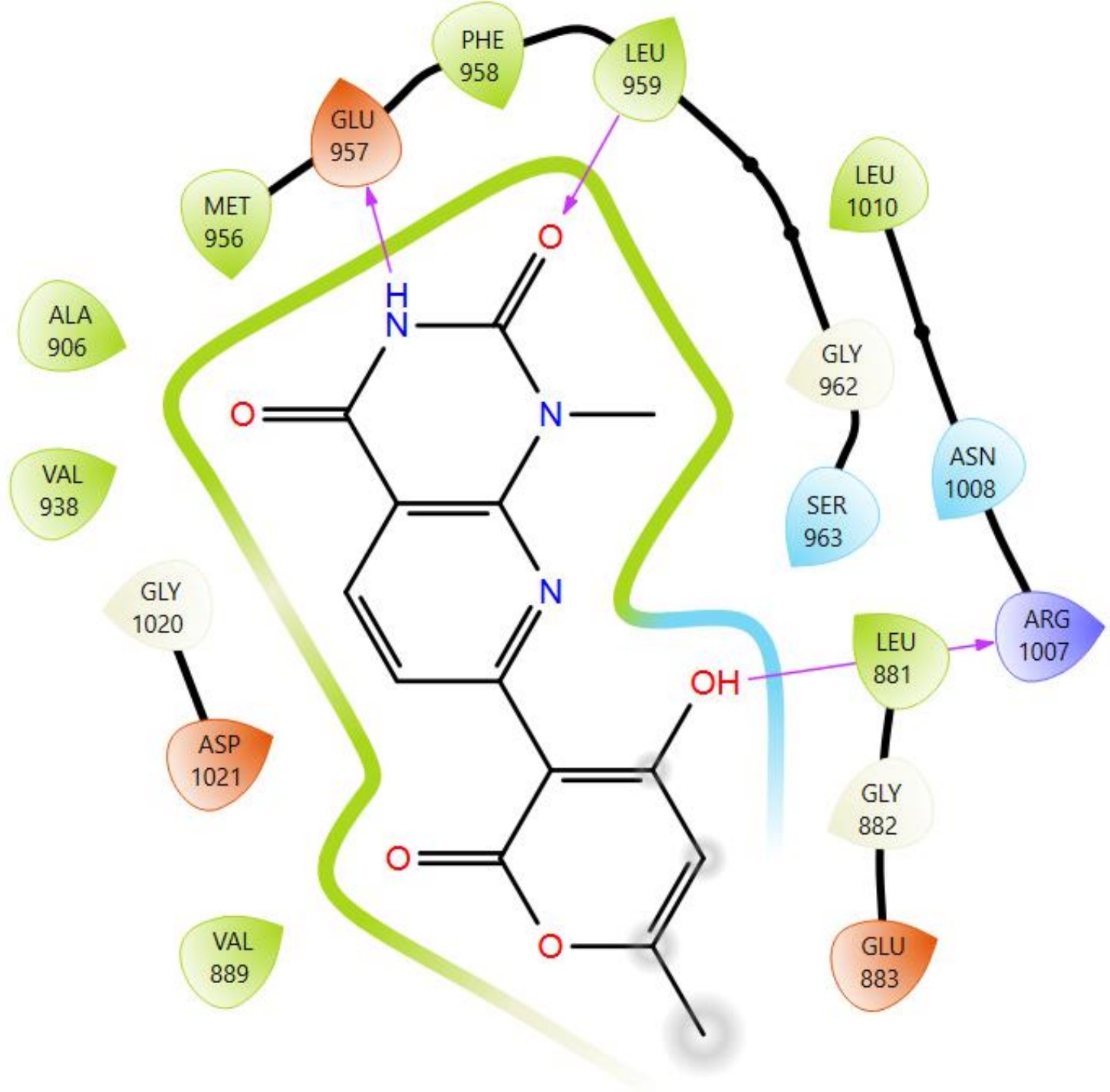

Figure S9. Two-dimensional interaction of STOCK1N-97980 in the active site of JAK1.

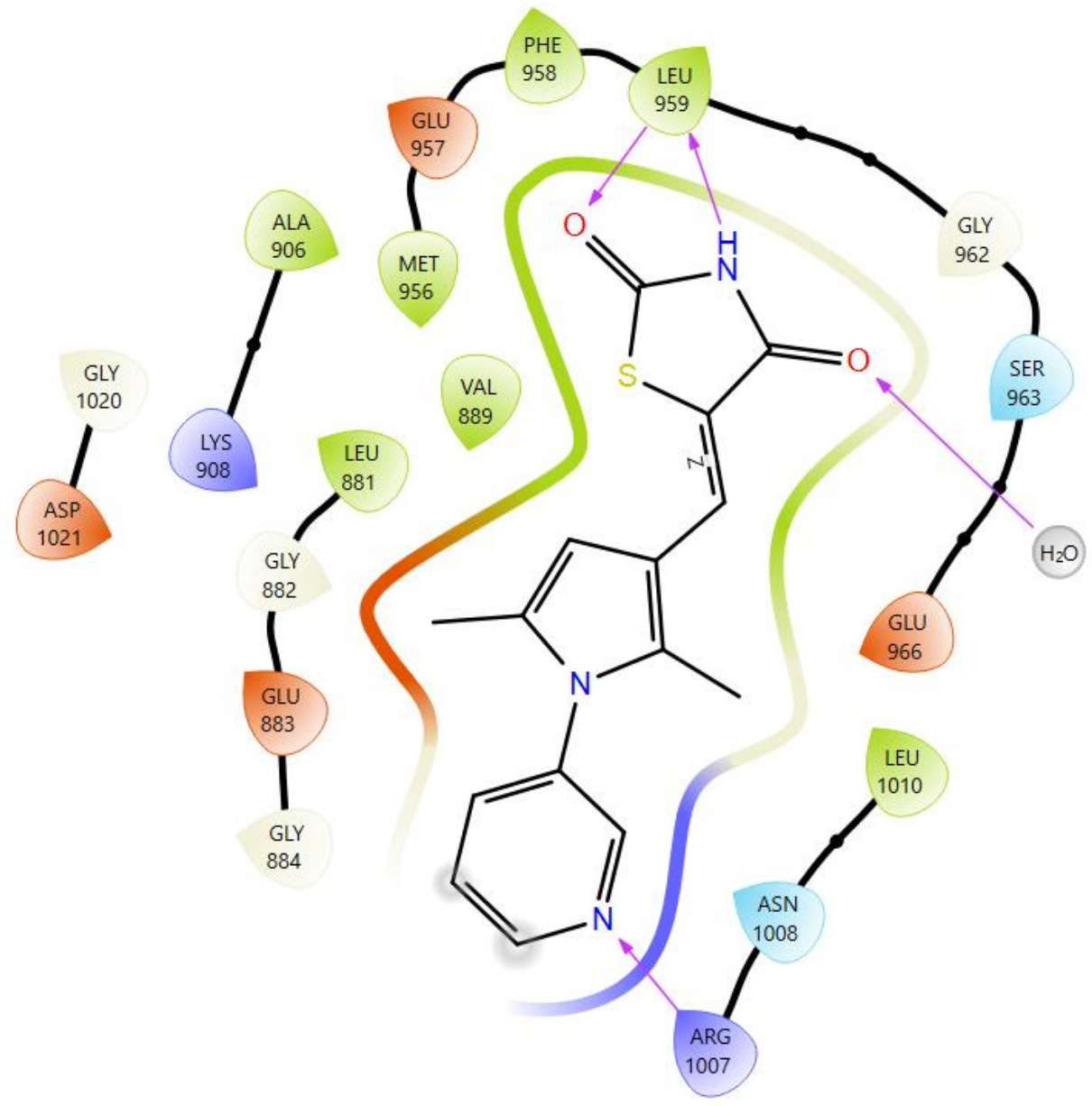

Figure S10. Two-dimensional interaction of STOCK2S-29689 in the active site of JAK1.

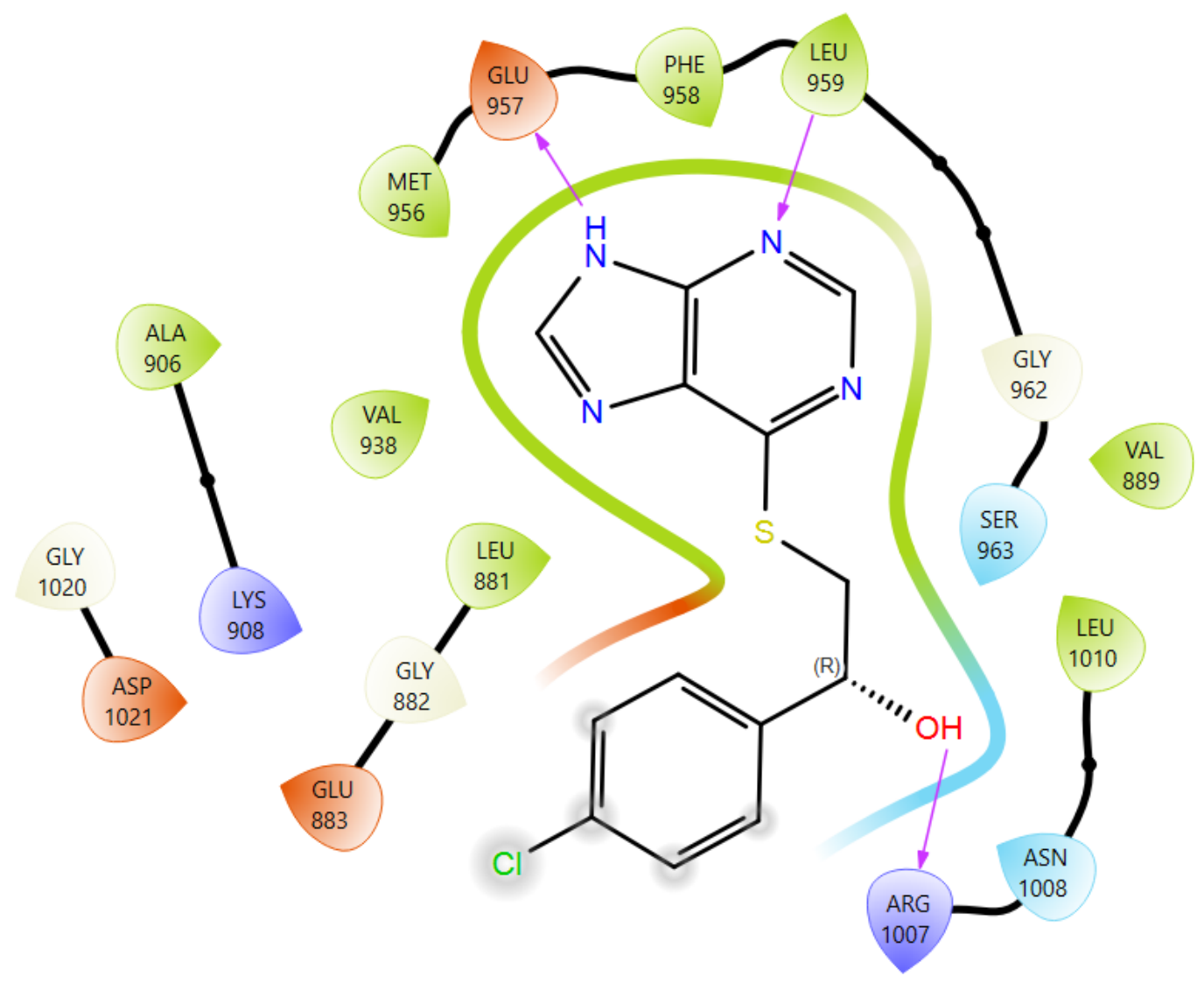

Figure S11. Two-dimensional interaction of STOCK2S-34232 in the active site of JAK1.

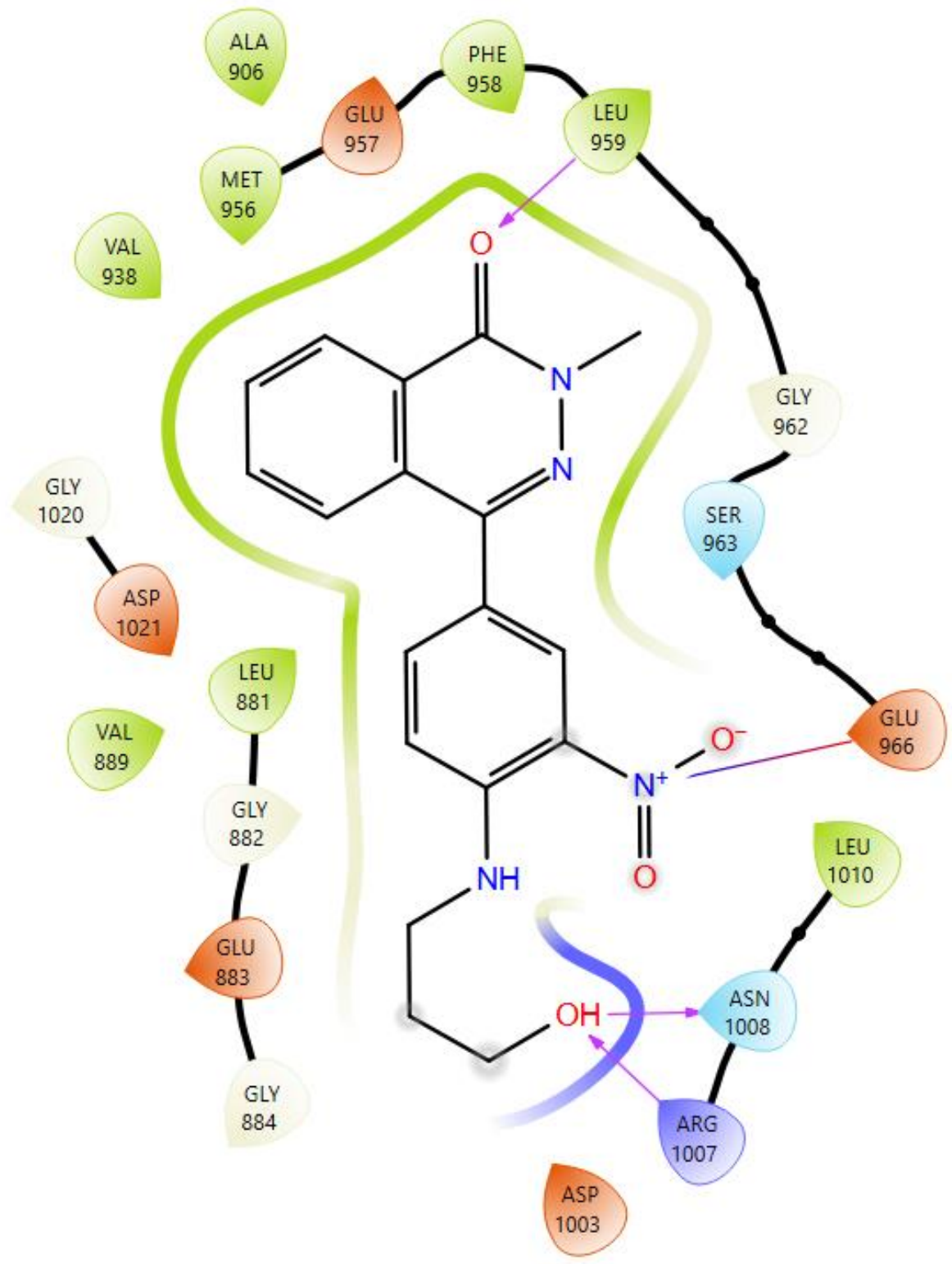

Figure S12. Two-dimensional interaction of STOCK4S-15629 in the active site of JAK1.

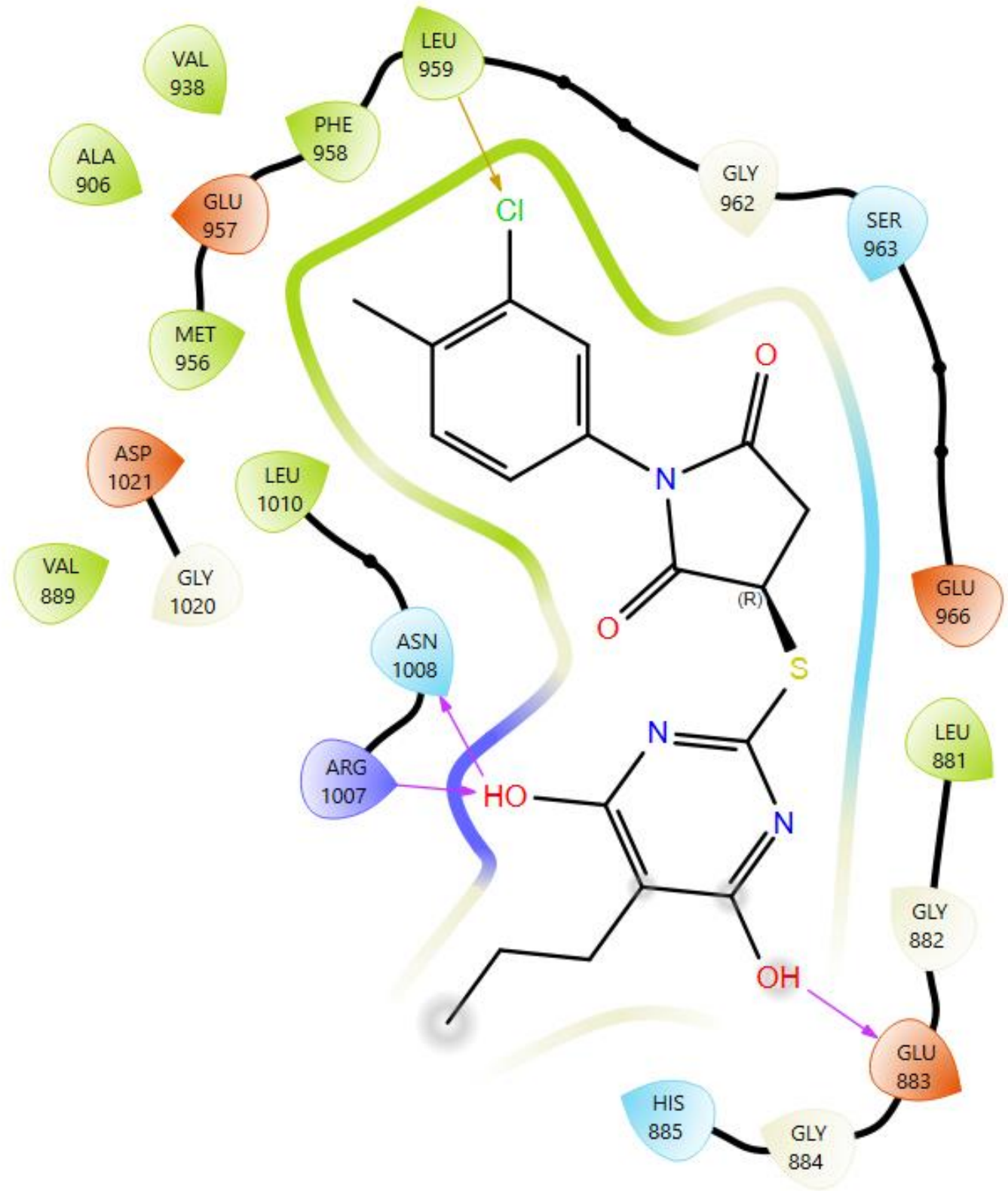

Figure S13. Two-dimensional interaction of STOCK4S-46885 in the active site of JAK1.

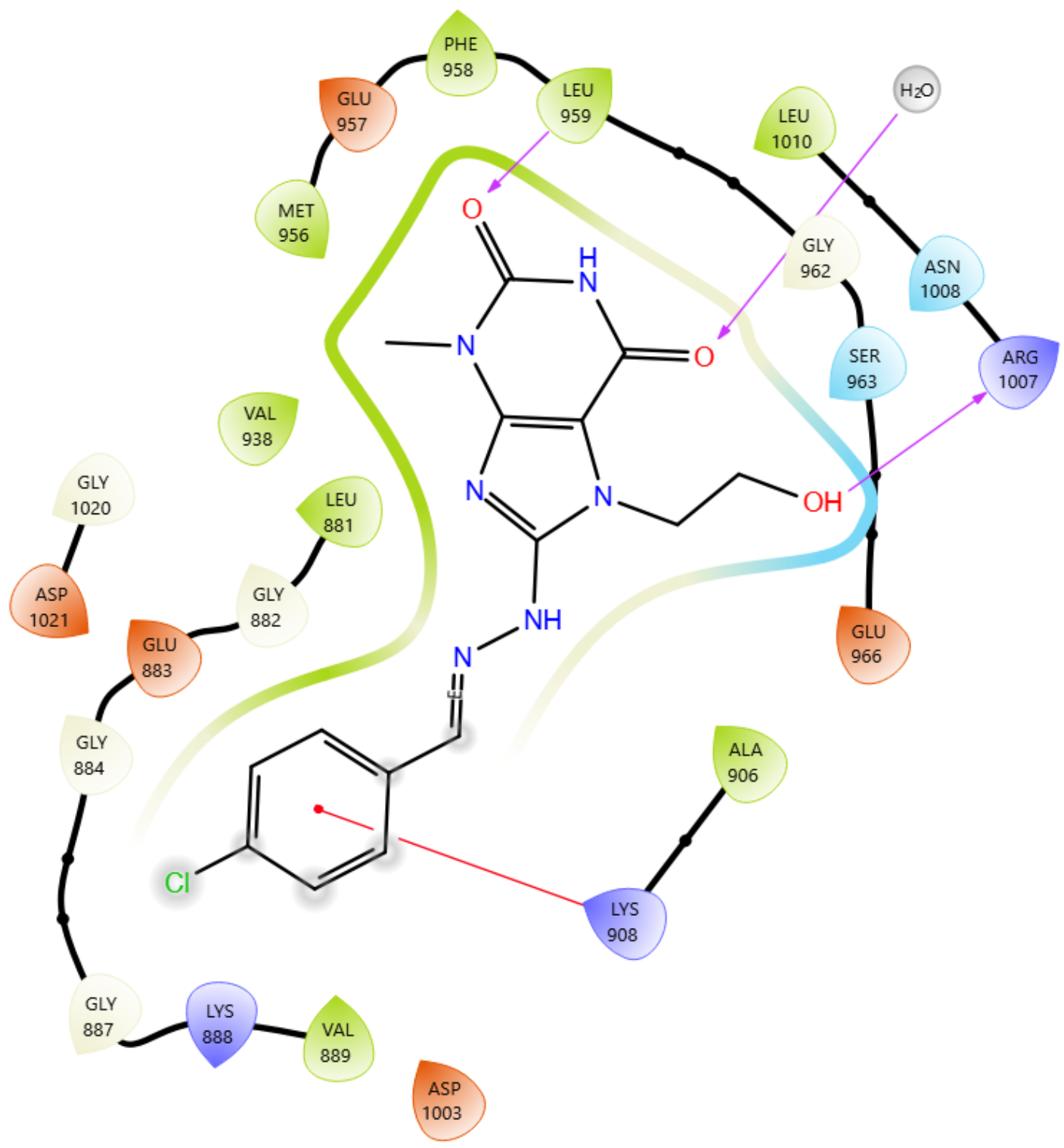

Figure S14. Two-dimensional interaction of STOCK4S-70675 in the active site of JAK1.

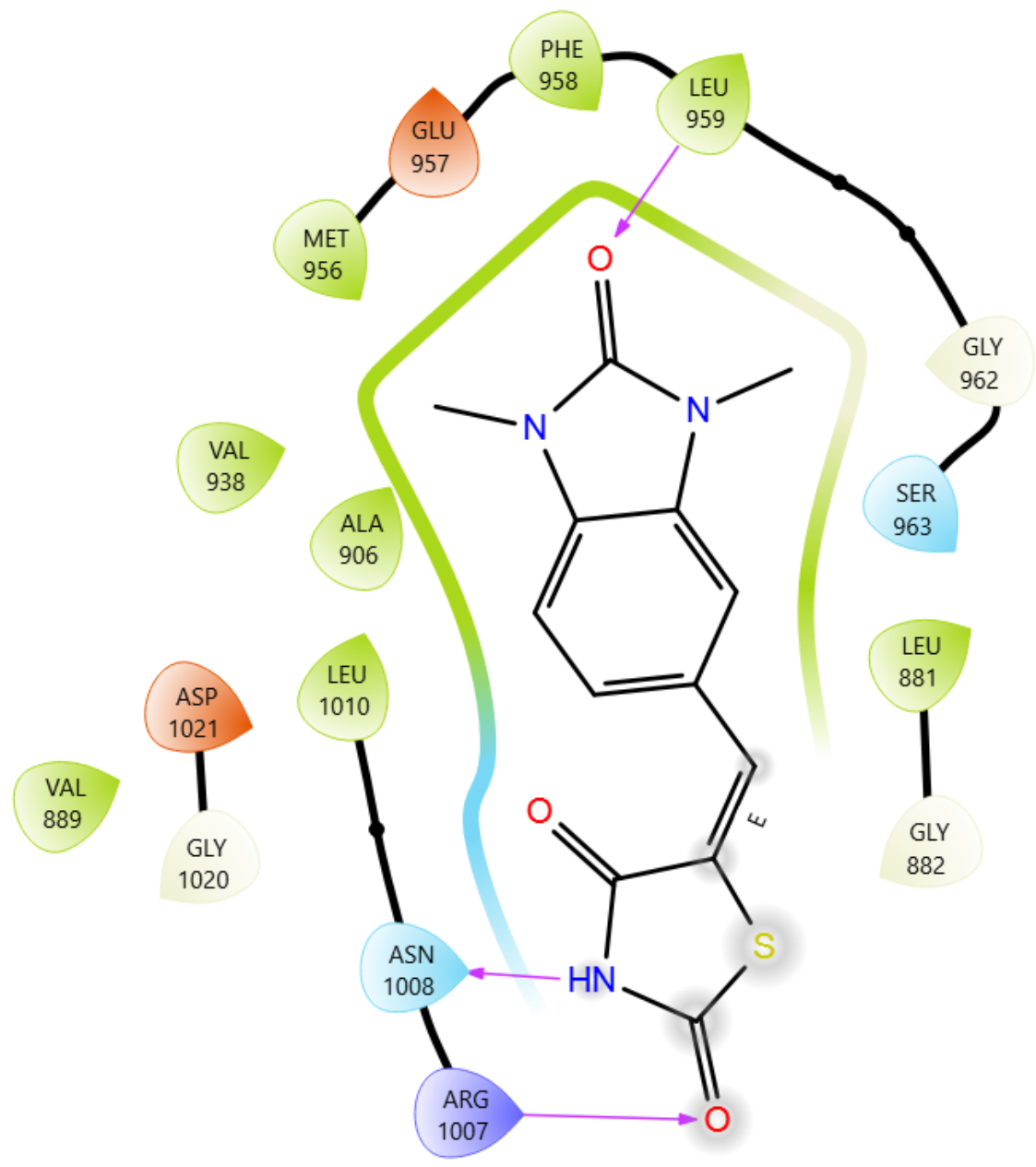

Figure S15. Two-dimensional interaction of STOCK5S-74239 in the active site of JAK1.

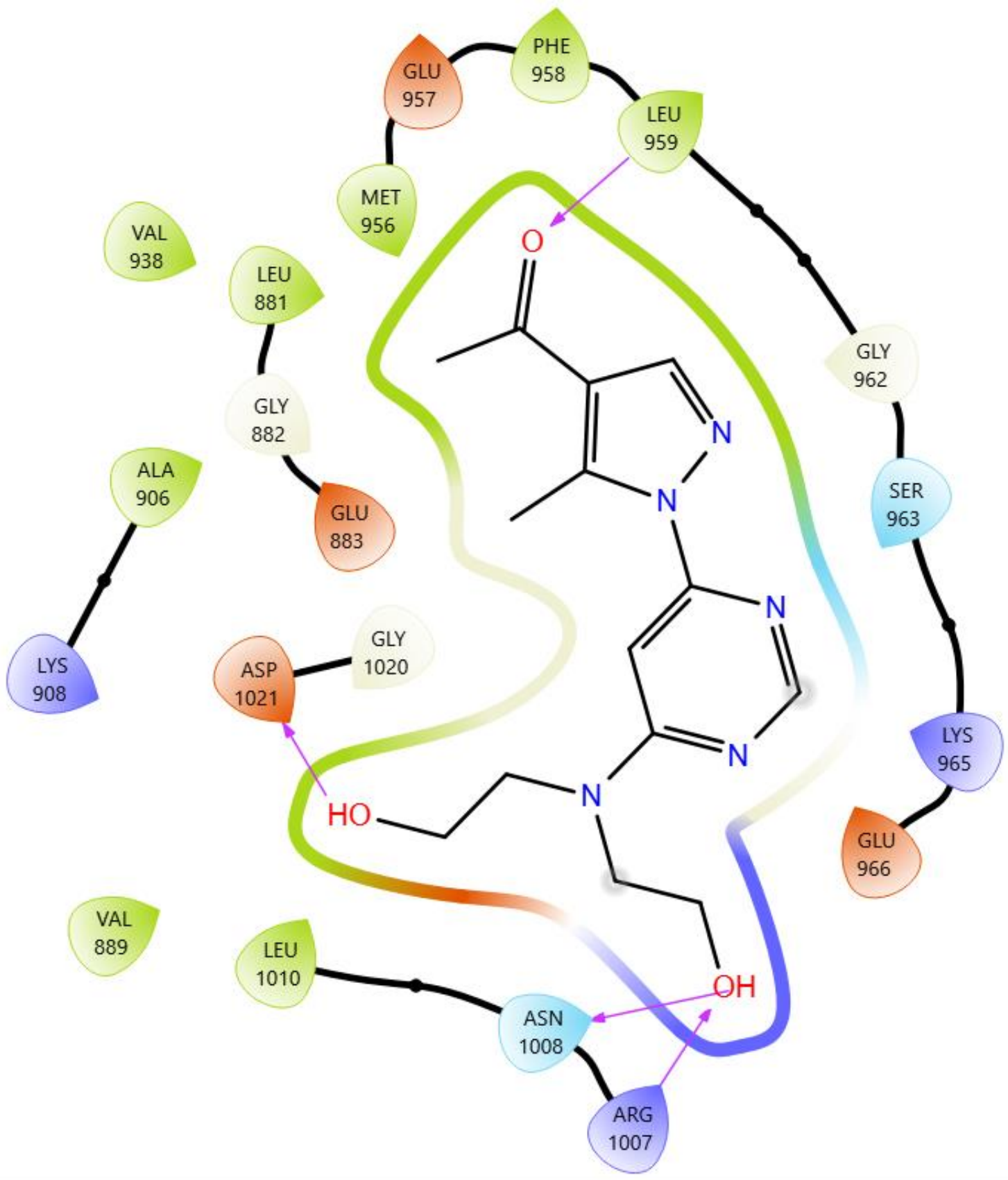

Figure S16. Two-dimensional interaction of STOCK5S-83560 in the active site of JAK1.

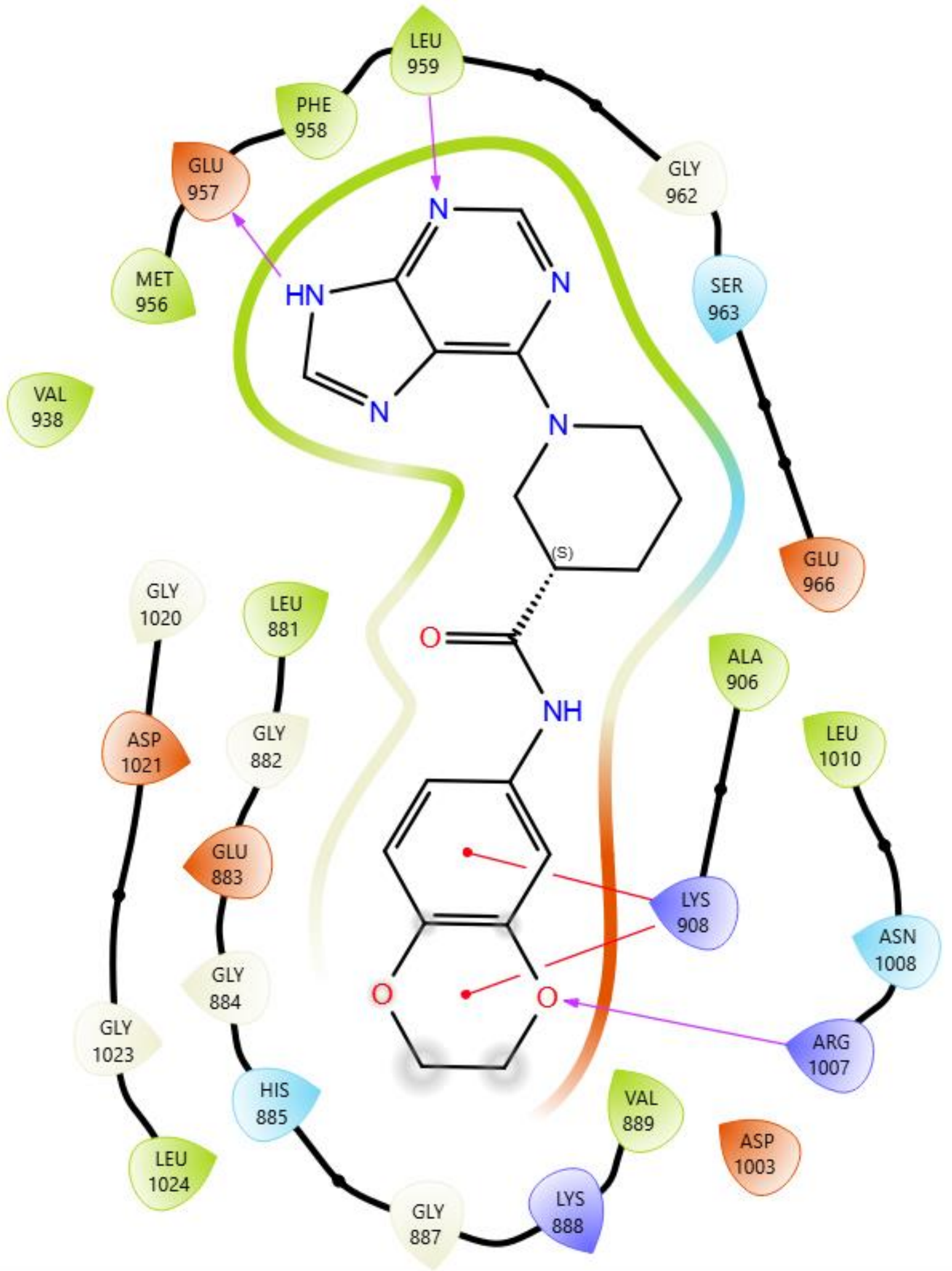

Figure S17. Two-dimensional interaction of STOCK6S-28538 in the active site of JAK1.

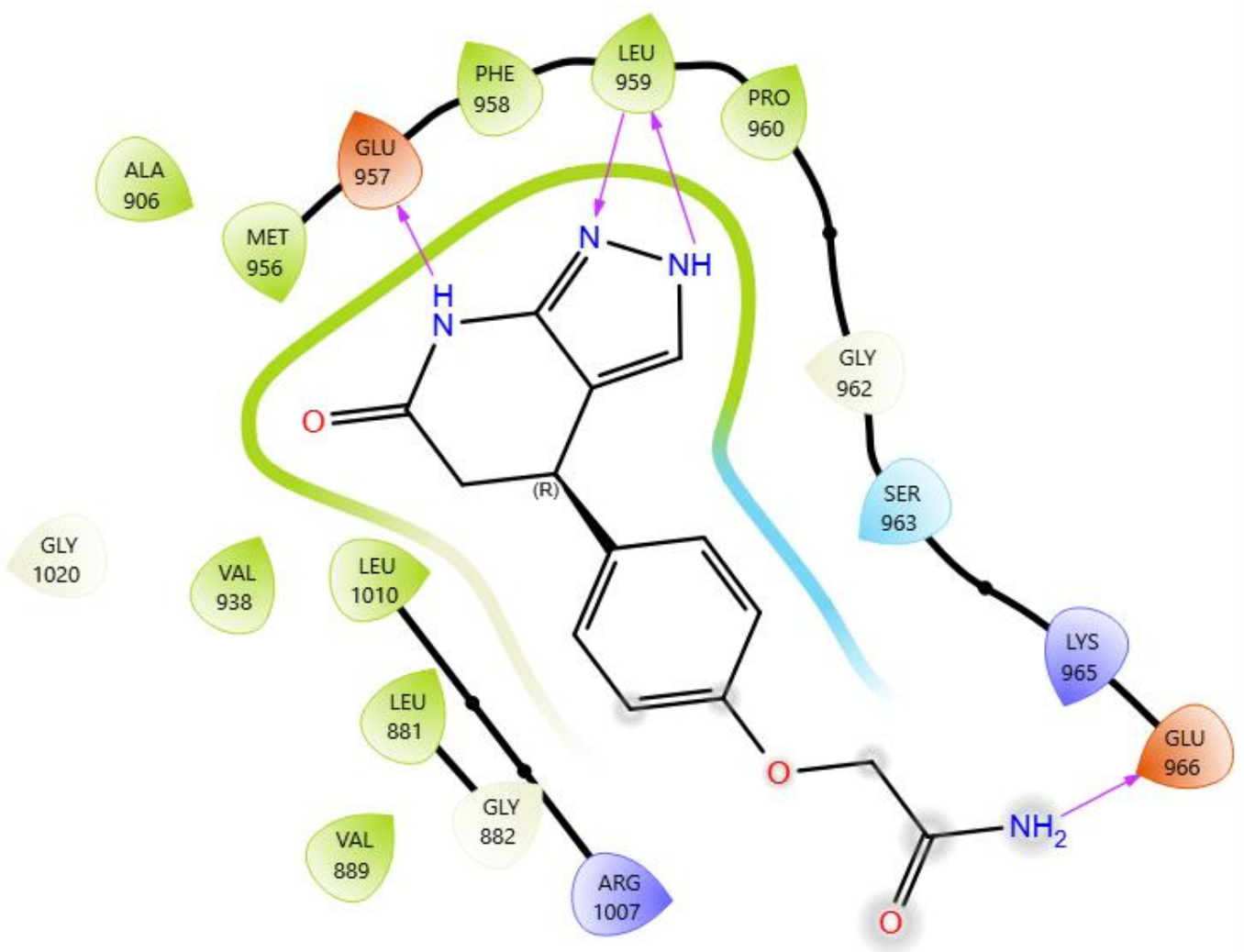

Figure S18. Two-dimensional interaction of STOCK6S-72898 in the active site of JAK1.

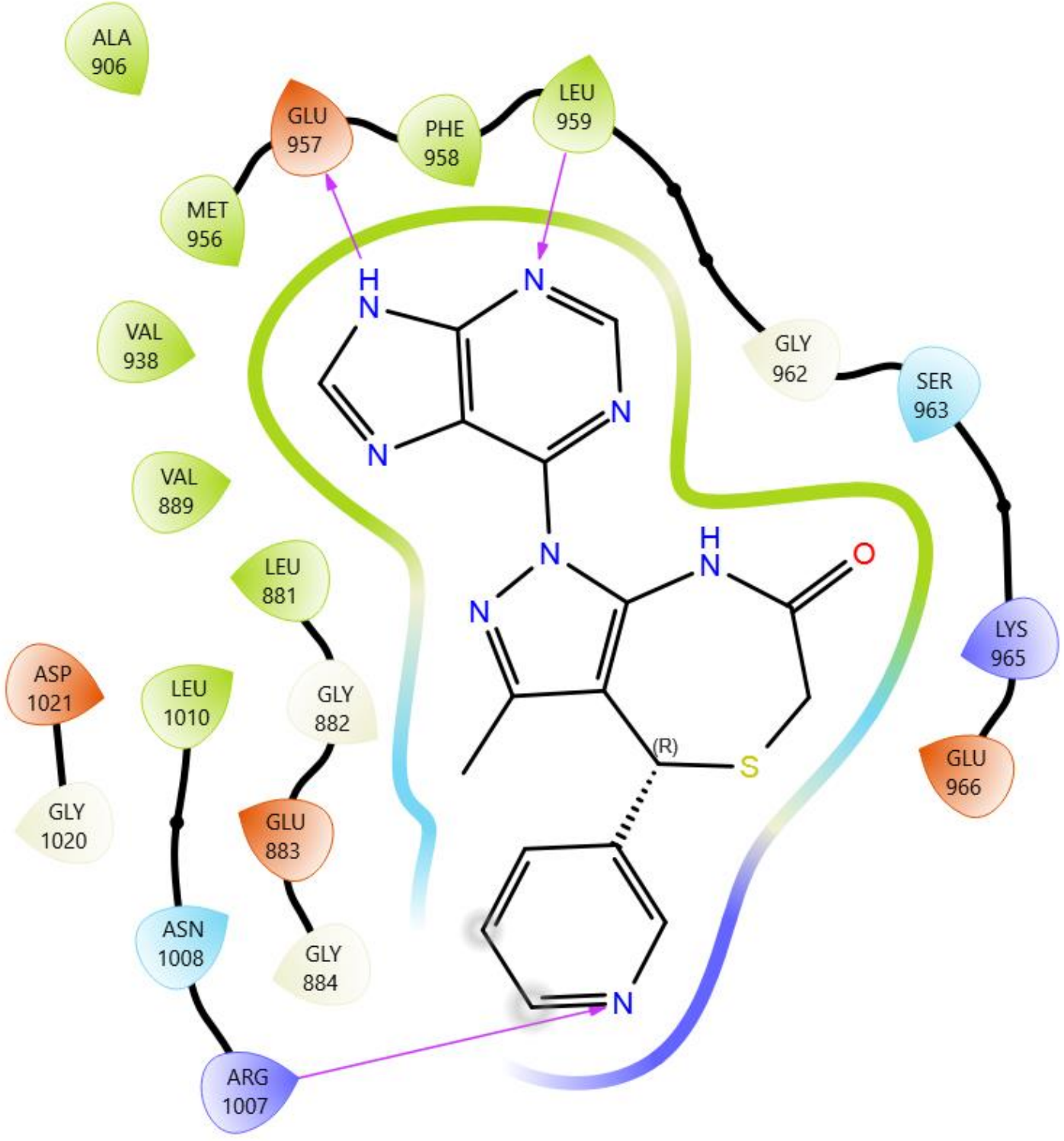

Figure S19. Two-dimensional interaction of STOCK6S-83203 in the active site of JAK1.

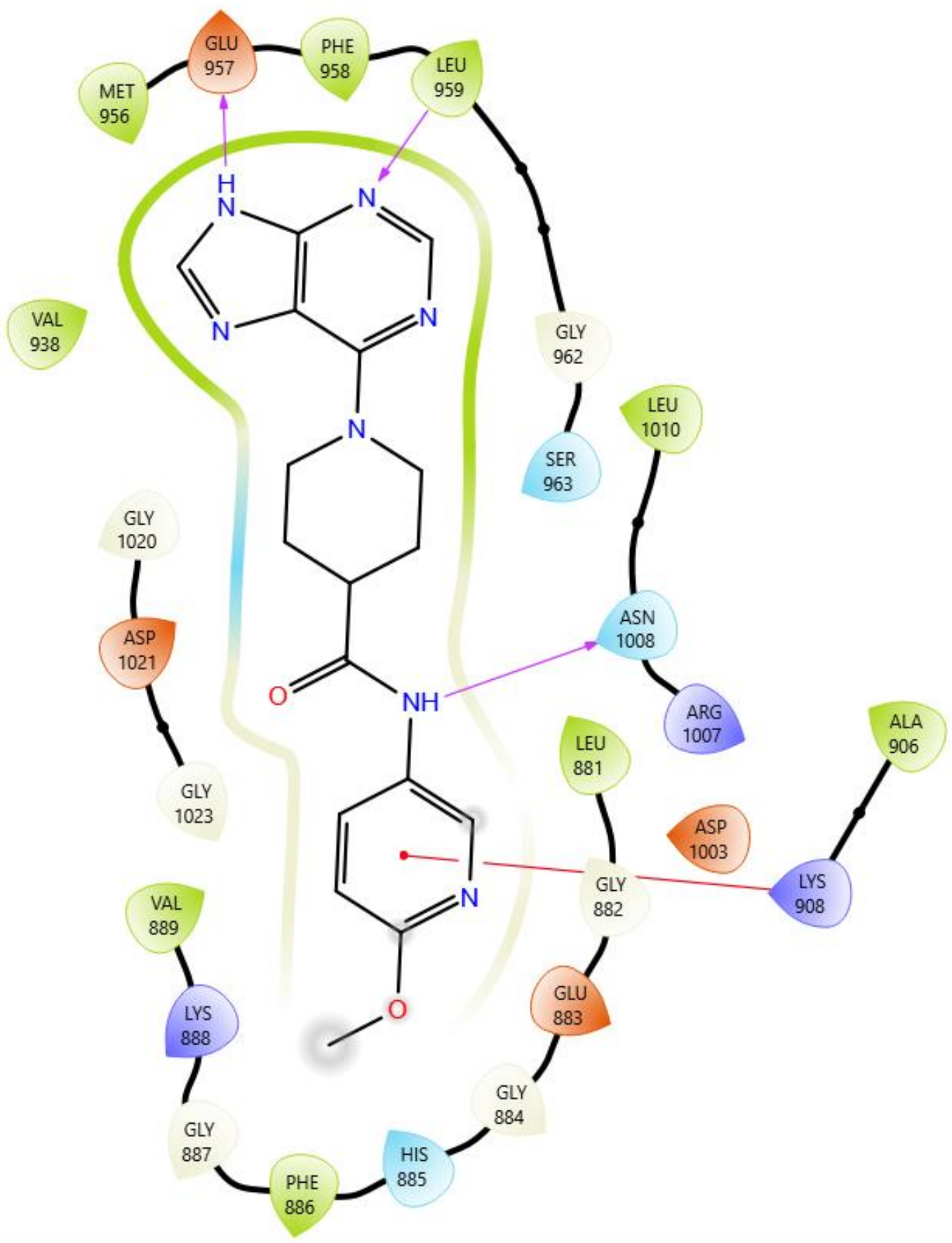

Figure S20. Two-dimensional interaction of STOCK7S-44229 in the active site of JAK1.

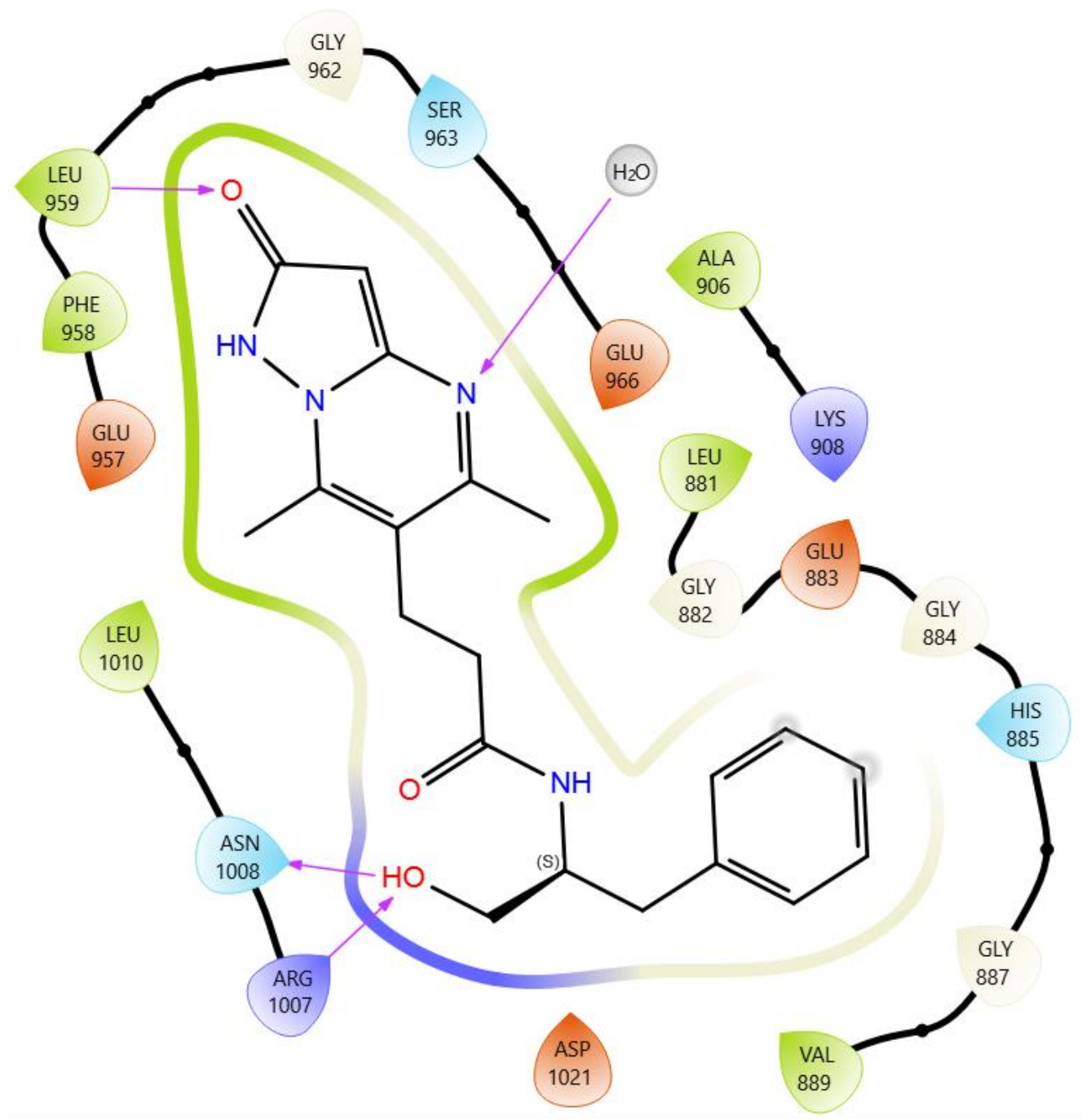

Figure S21. Two-dimensional interaction of STOCK7S-51860 in the active site of JAK1.

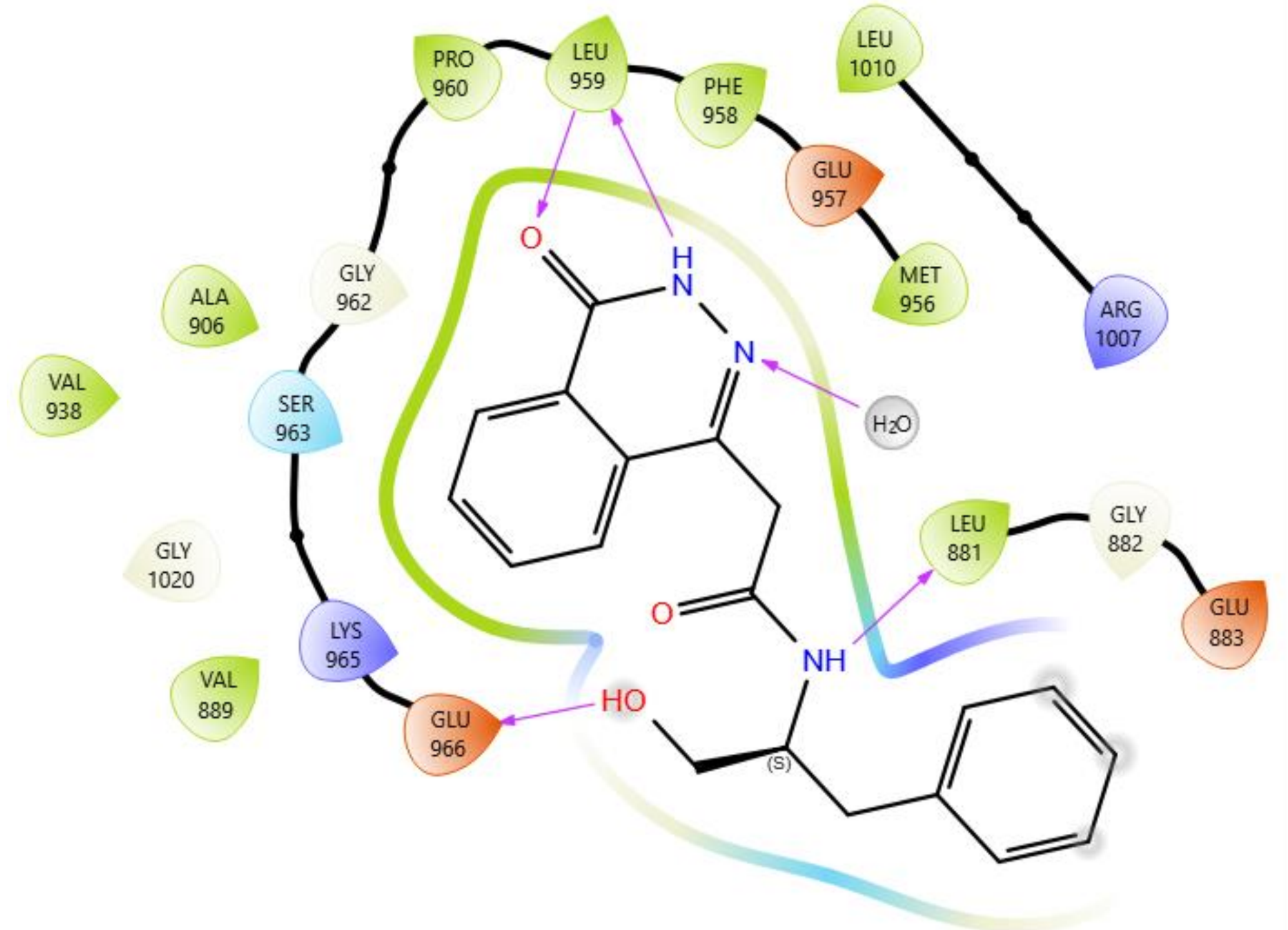

Figure S22. Two-dimensional interaction of STOCK7S-53671 in the active site of JAK1.

JAK2

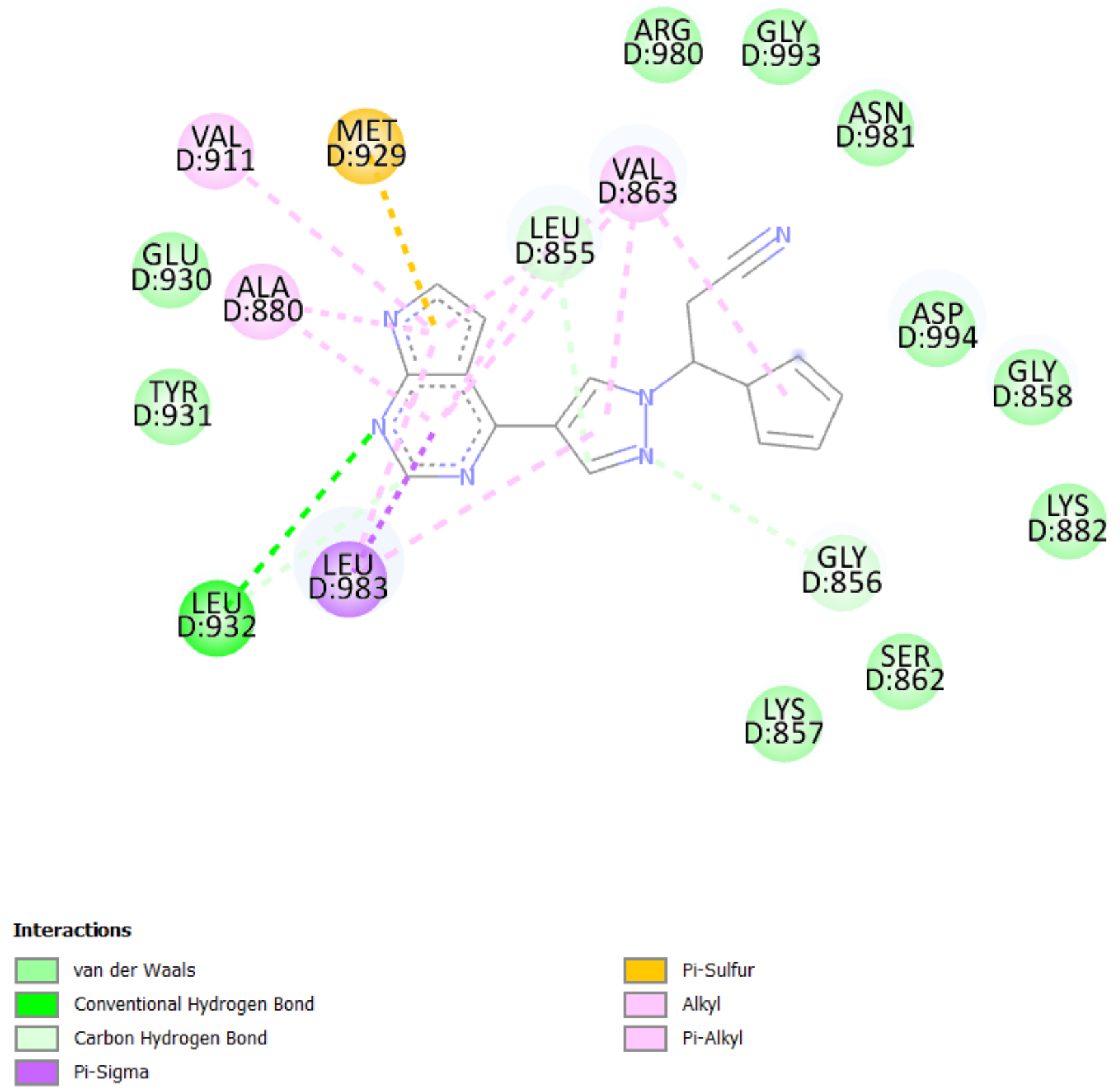

Figure S23. Two-dimensional interaction of Ruxolutinib in the active site of JAK2.

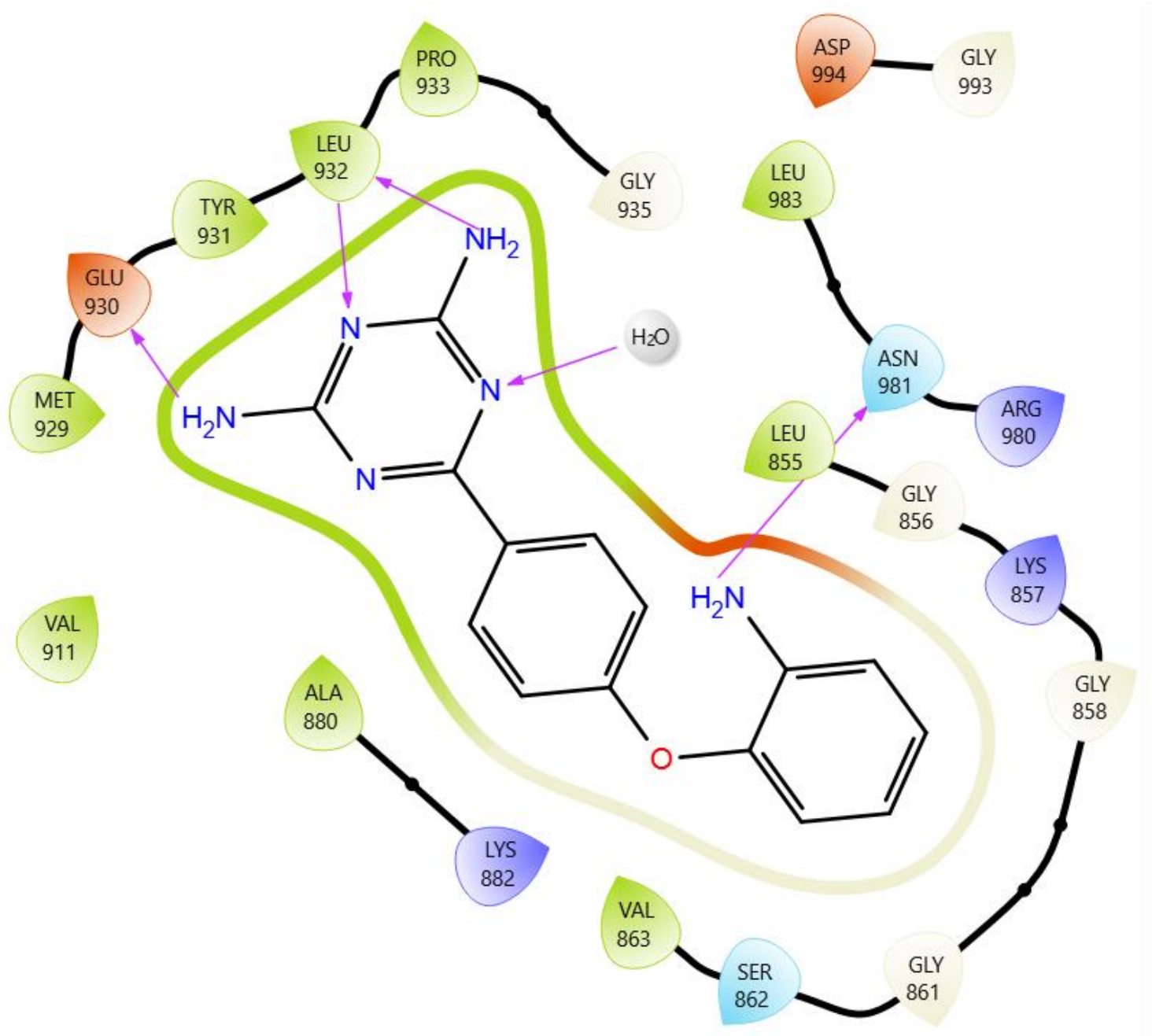

Figure S24. Two-dimensional interaction of STOCK1S-00944 in the active site of JAK2.

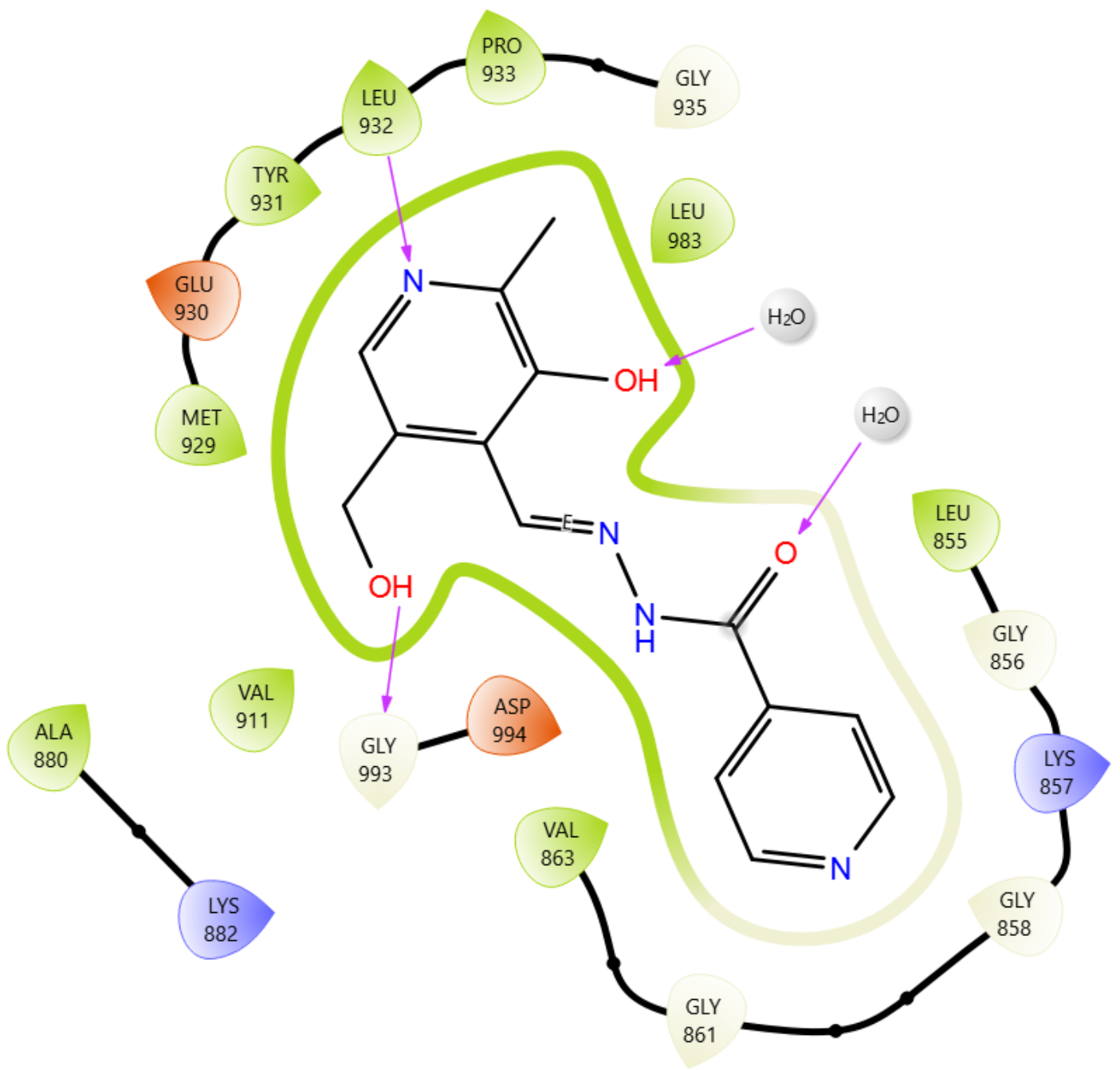

Figure S25. Two-dimensional interaction of STOCK1S-06278 in the active site of JAK2.

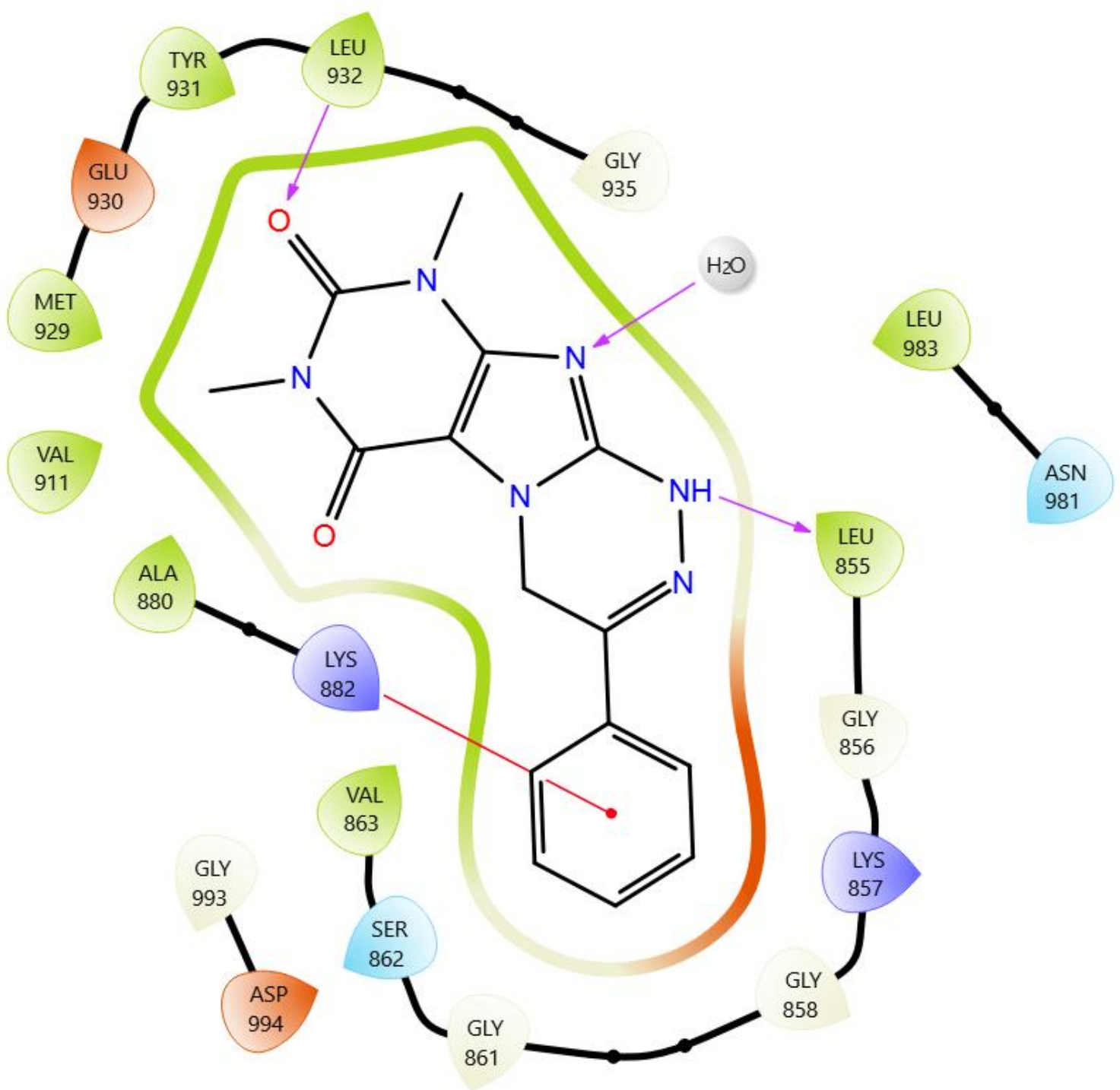

Figure S26. Two-dimensional interaction of STOCK1S-10380 in the active site of JAK2.

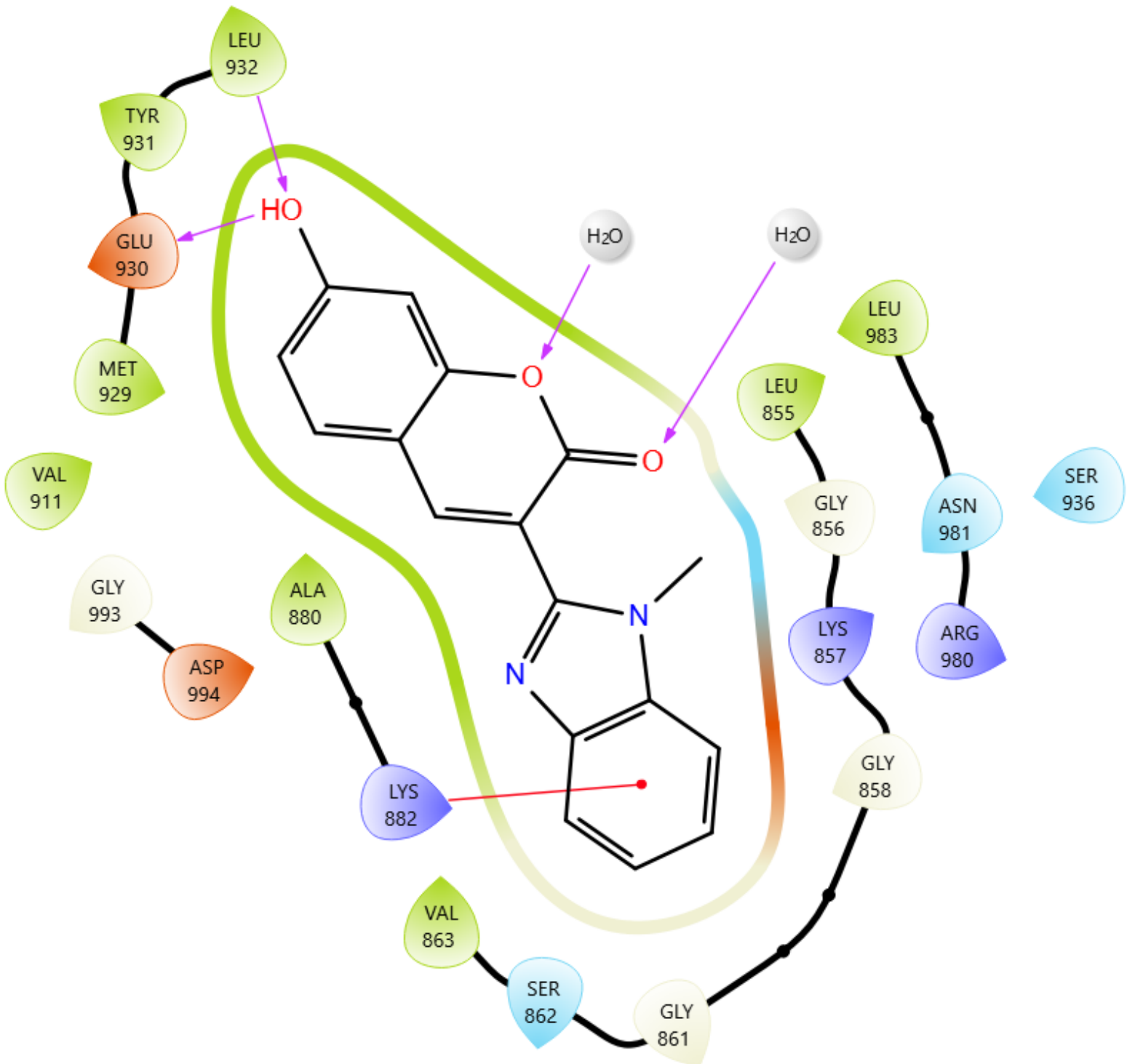

Figure S27. Two-dimensional interaction of STOCK1S-69008 in the active site of JAK2.

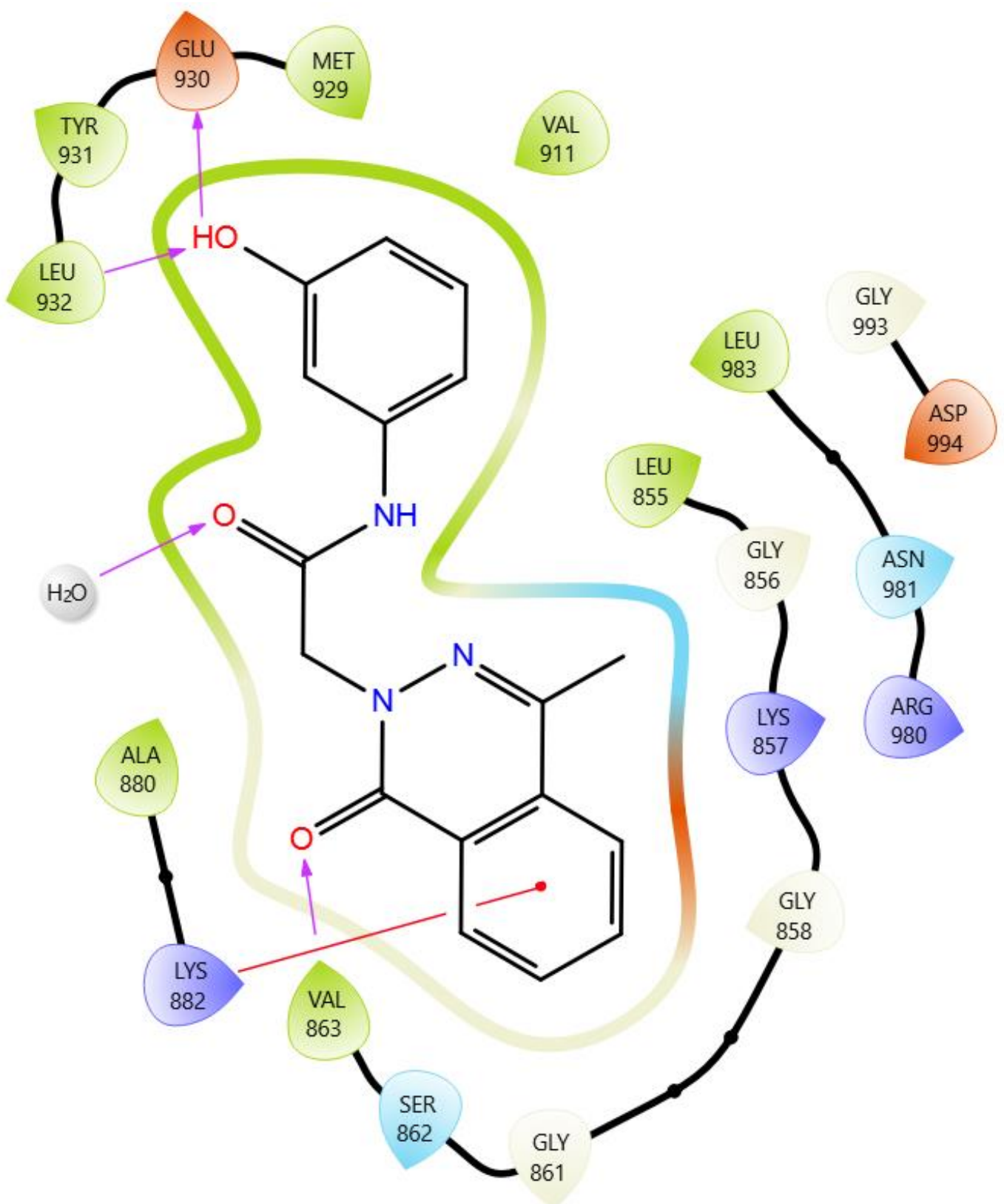

Figure S28. Two-dimensional interaction of STOCK1S-78591 in the active site of JAK2.

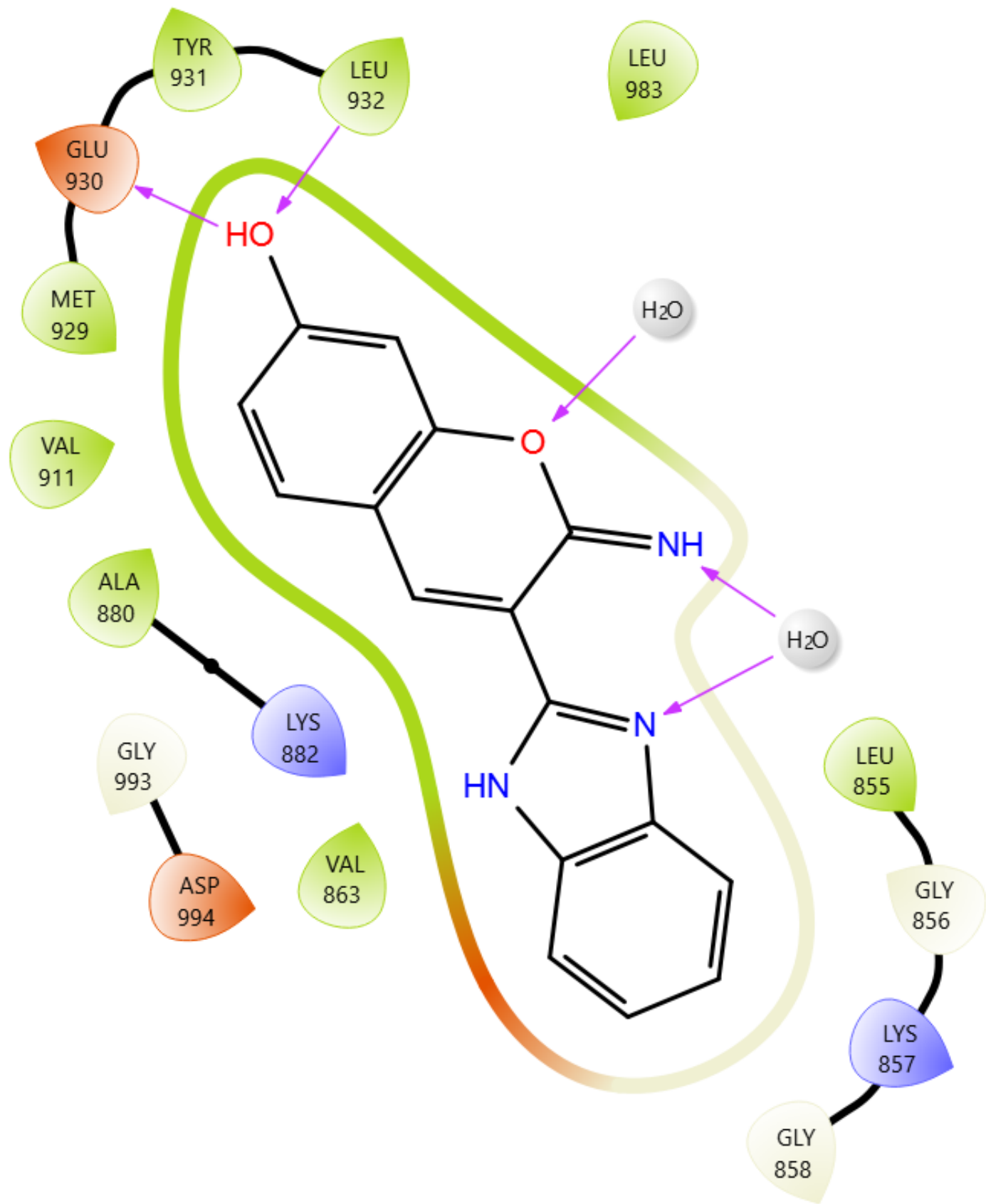

Figure S29. Two-dimensional interaction of STOCK2S-08448 in the active site of JAK2.

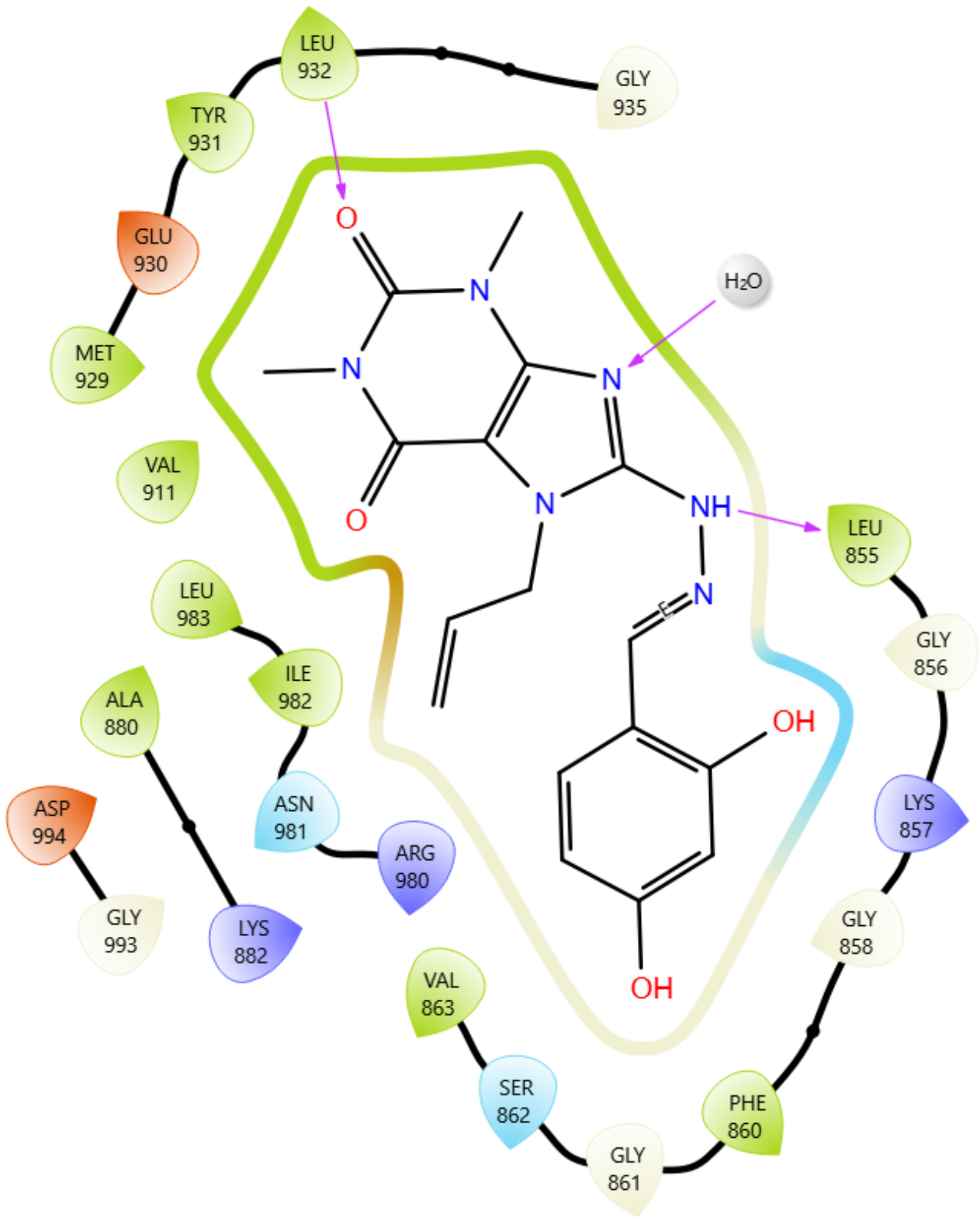

Figure S30. Two-dimensional interaction of STOCK2S-18844 in the active site of JAK2.

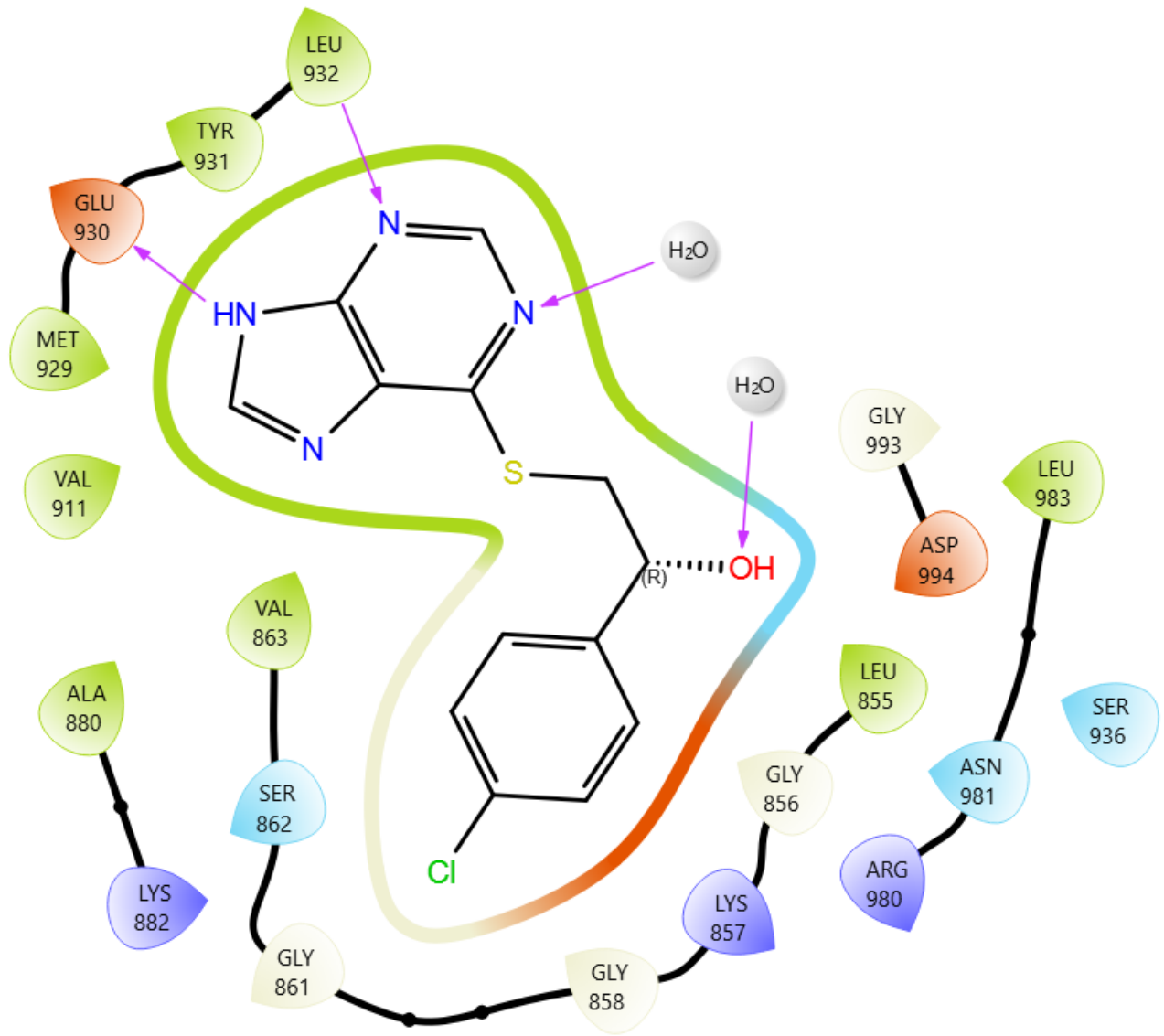

Figure S31. Two-dimensional interaction of STOCK2S-34232 in the active site of JAK2.

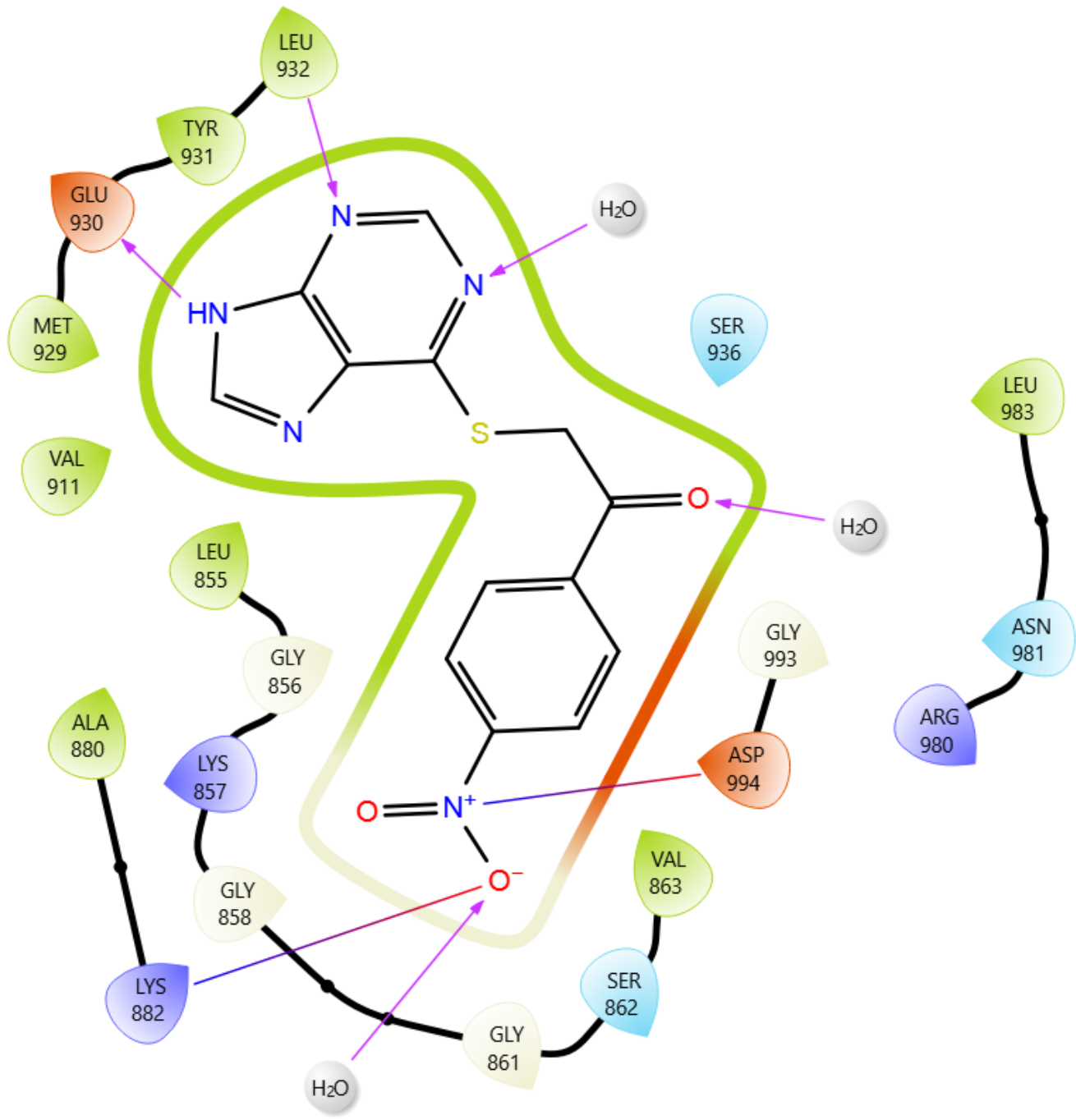

Figure S32. Two-dimensional interaction of STOCK2S-52524 in the active site of JAK2.

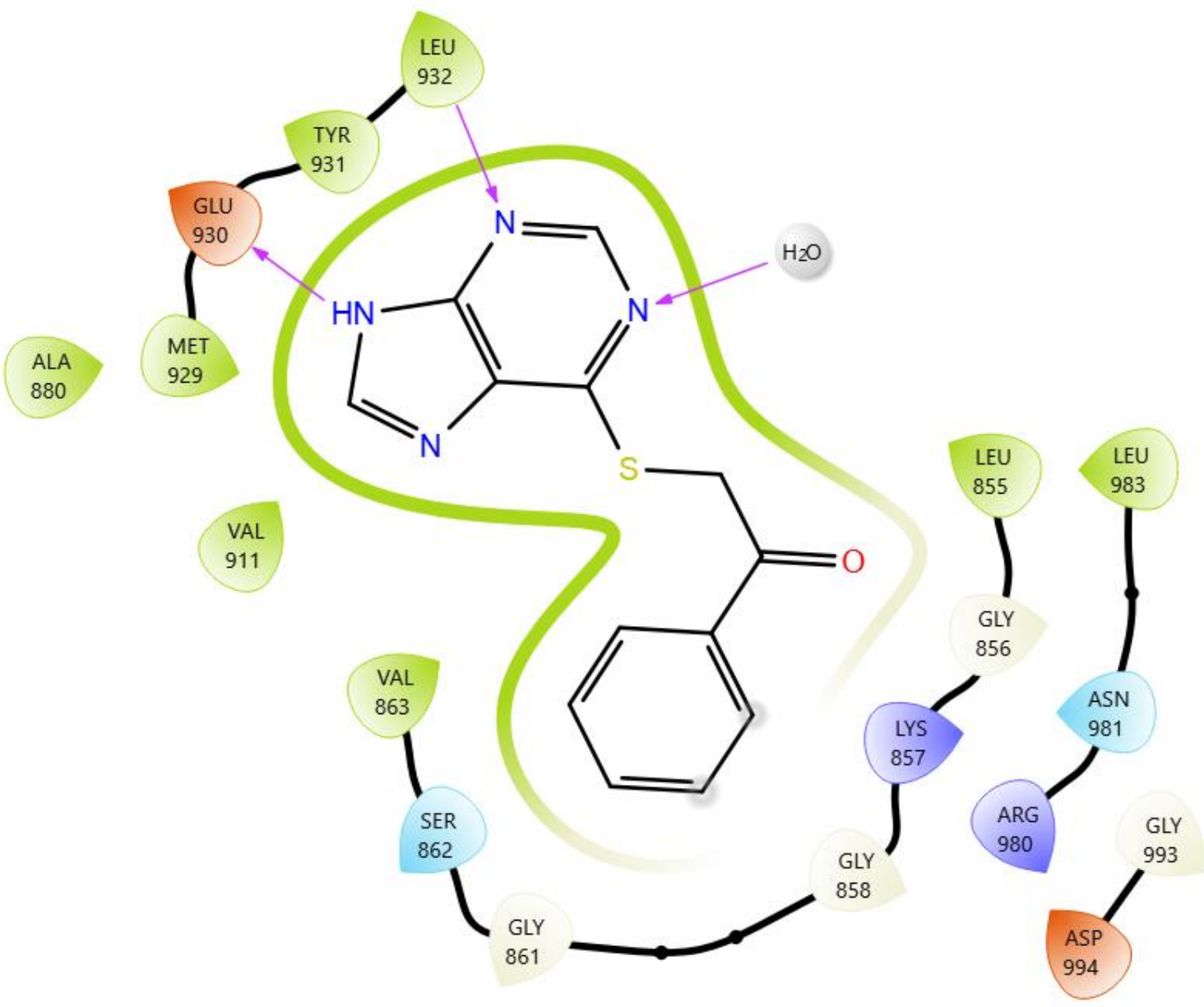

Figure S33. Two-dimensional interaction of STOCK2S-54227 in the active site of JAK2.

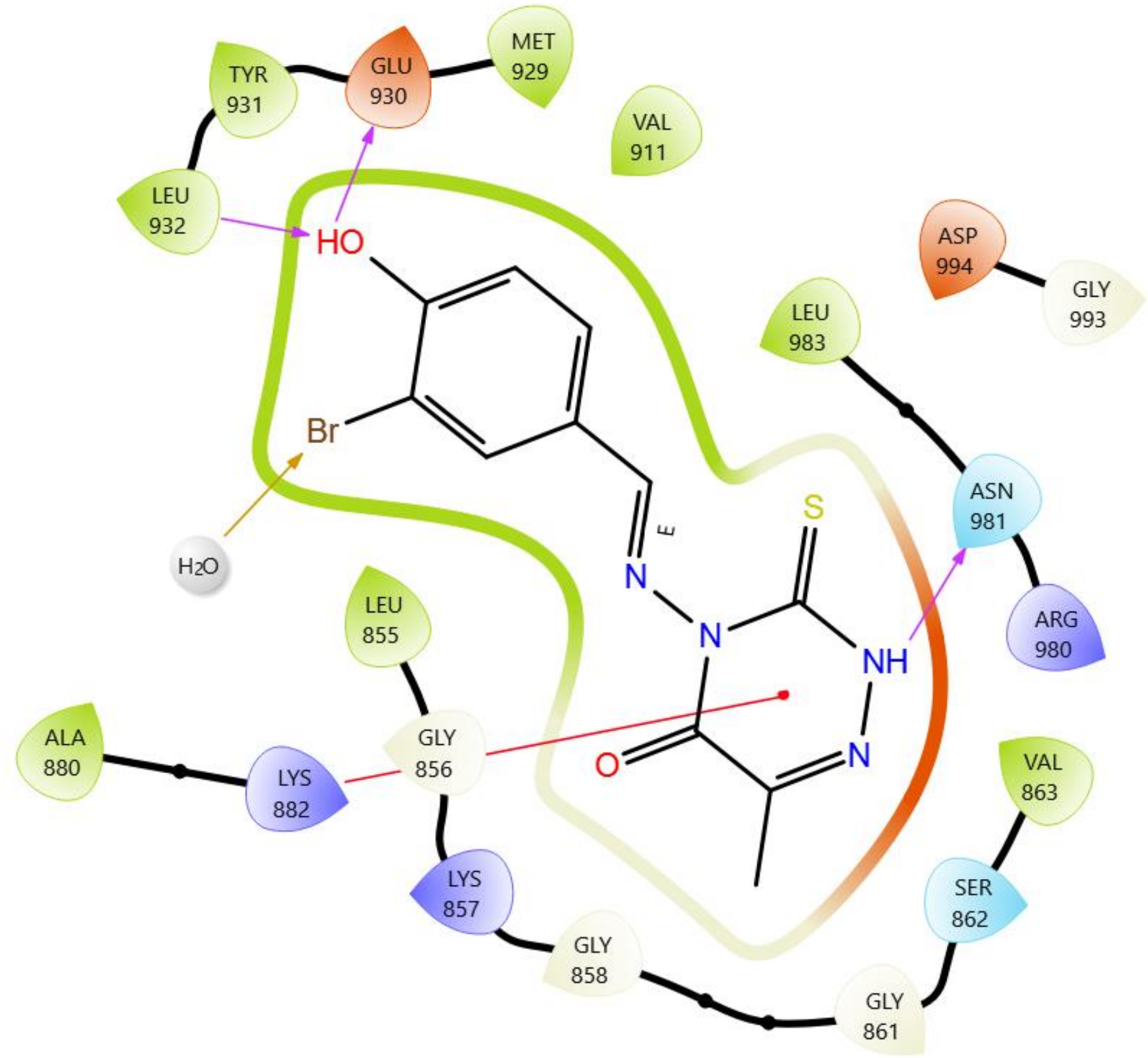

Figure S34. Two-dimensional interaction of STOCK2S-60777 in the active site of JAK2.

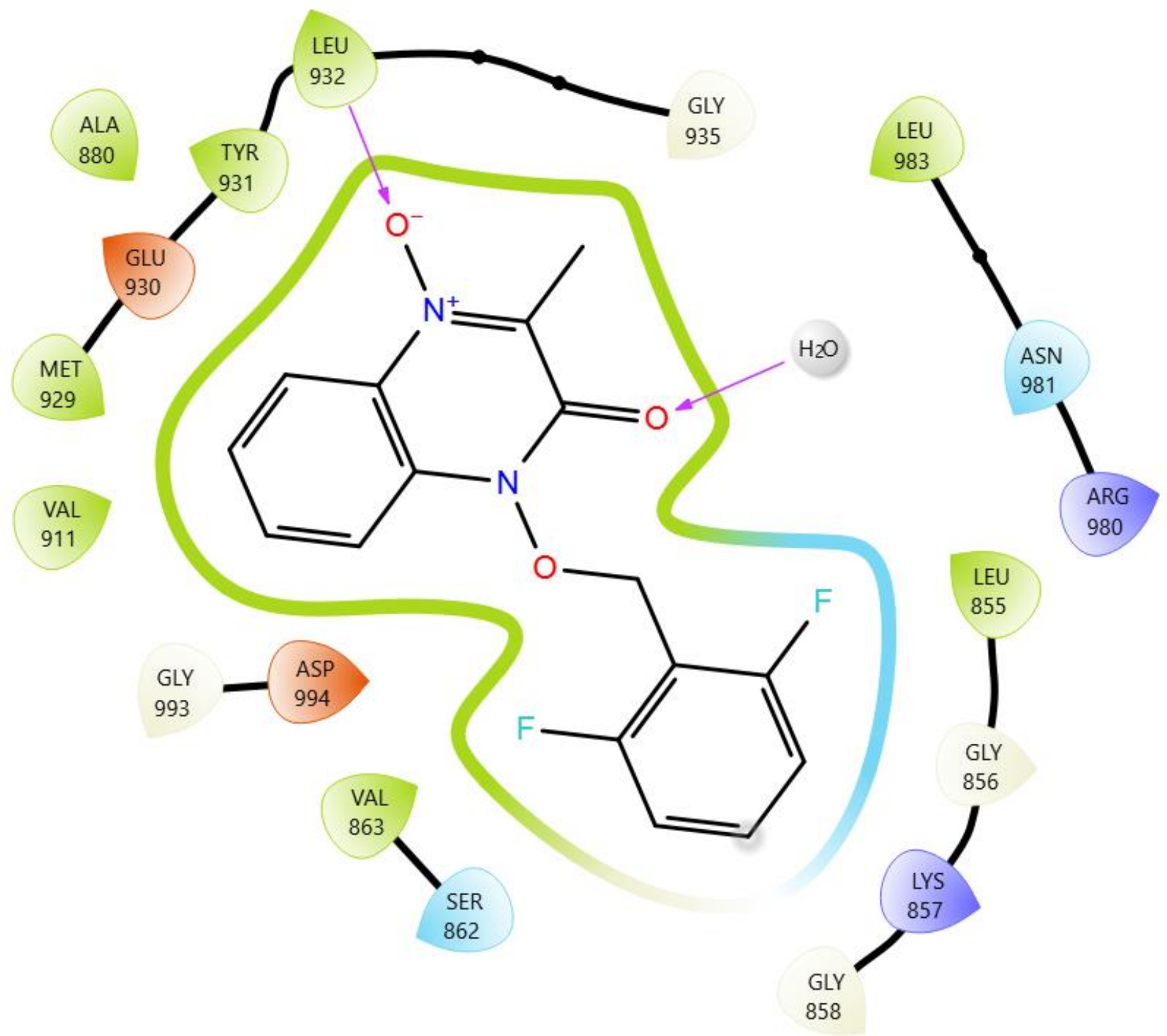

Figure S35. Two-dimensional interaction of STOCK2S-75169 in the active site of JAK2.

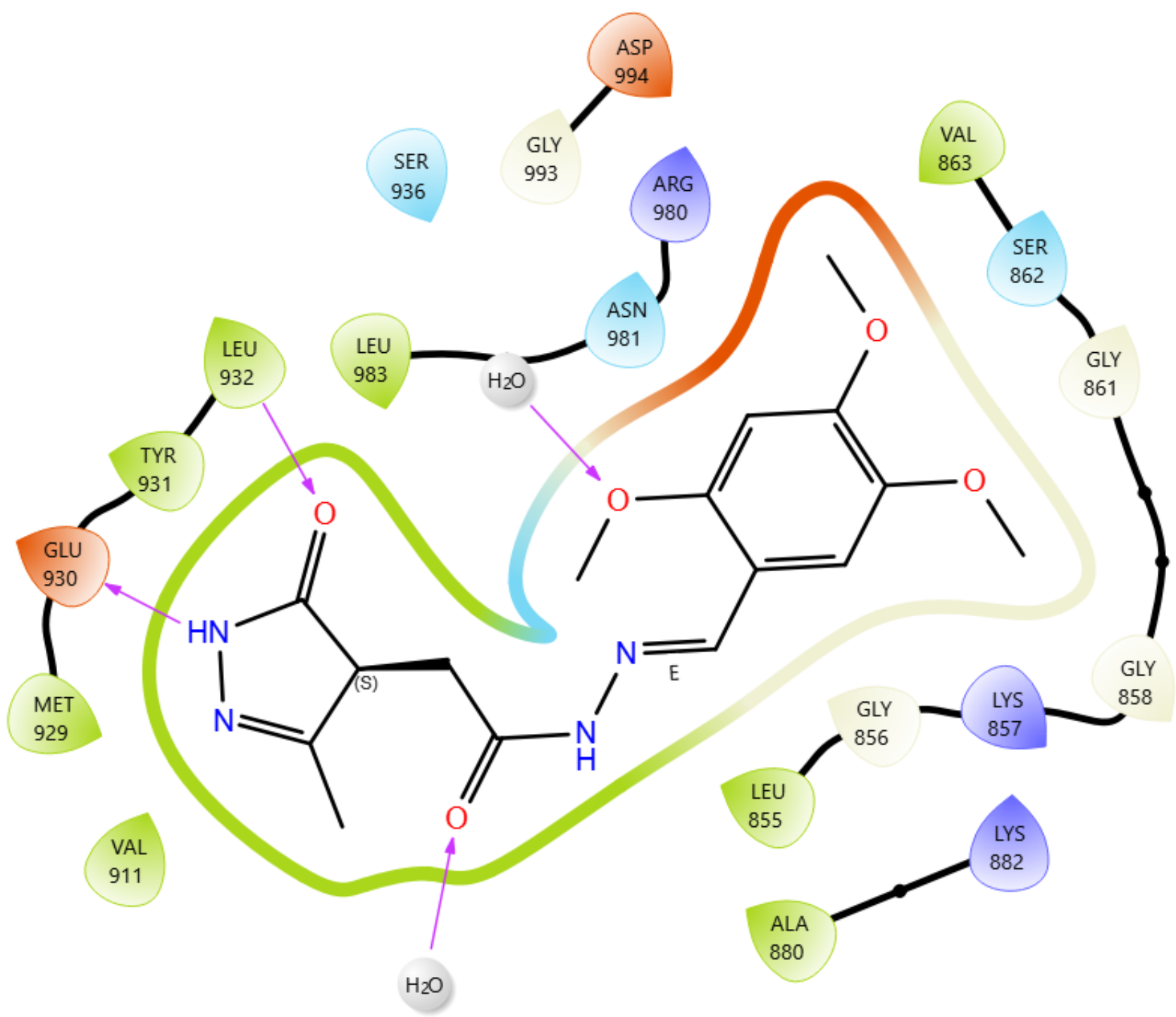

Figure S36. Two-dimensional interaction of STOCK3S-18016 in the active site of JAK2.

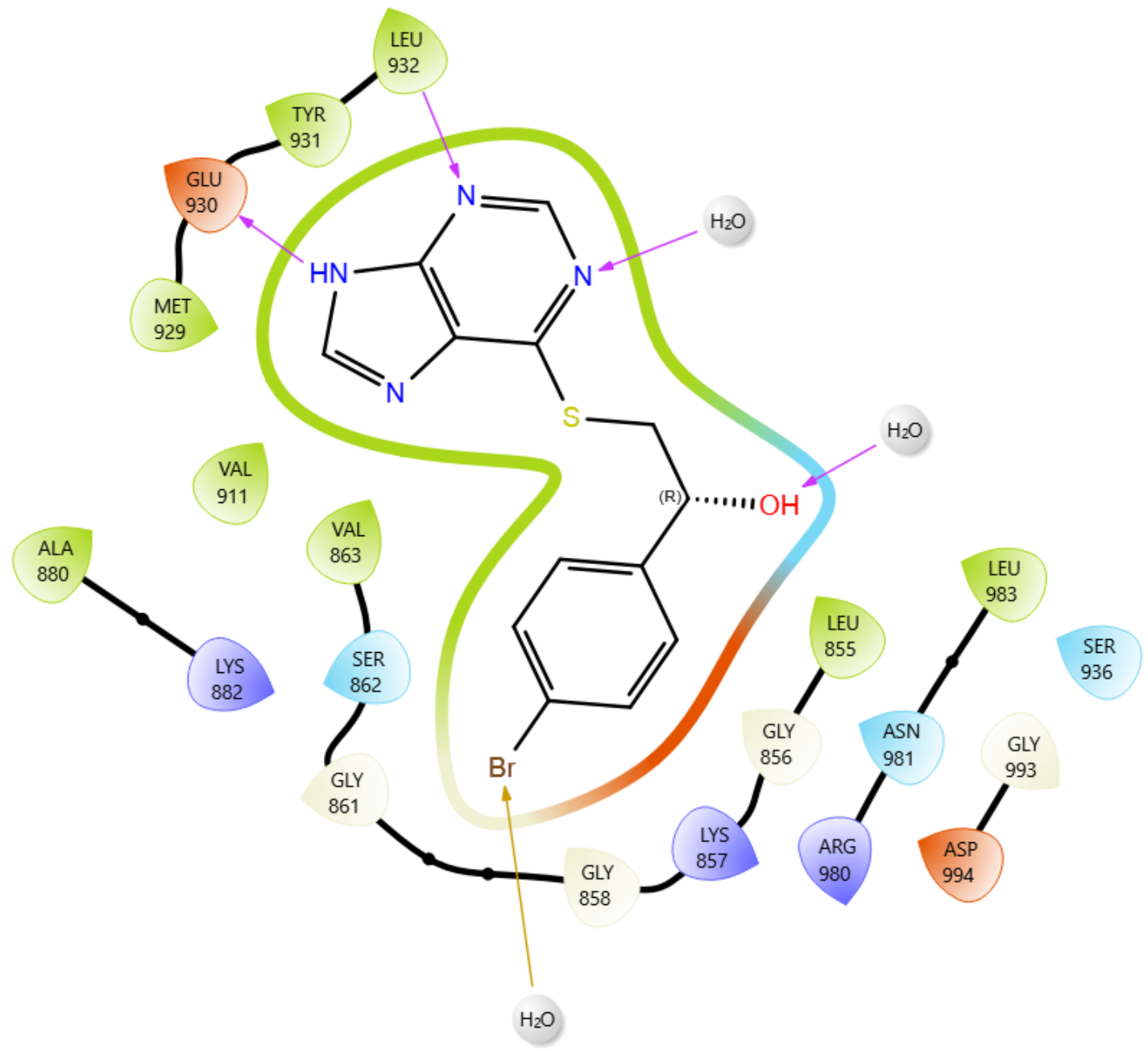

Figure S37. Two-dimensional interaction of STOCK3S-18468 in the active site of JAK2.

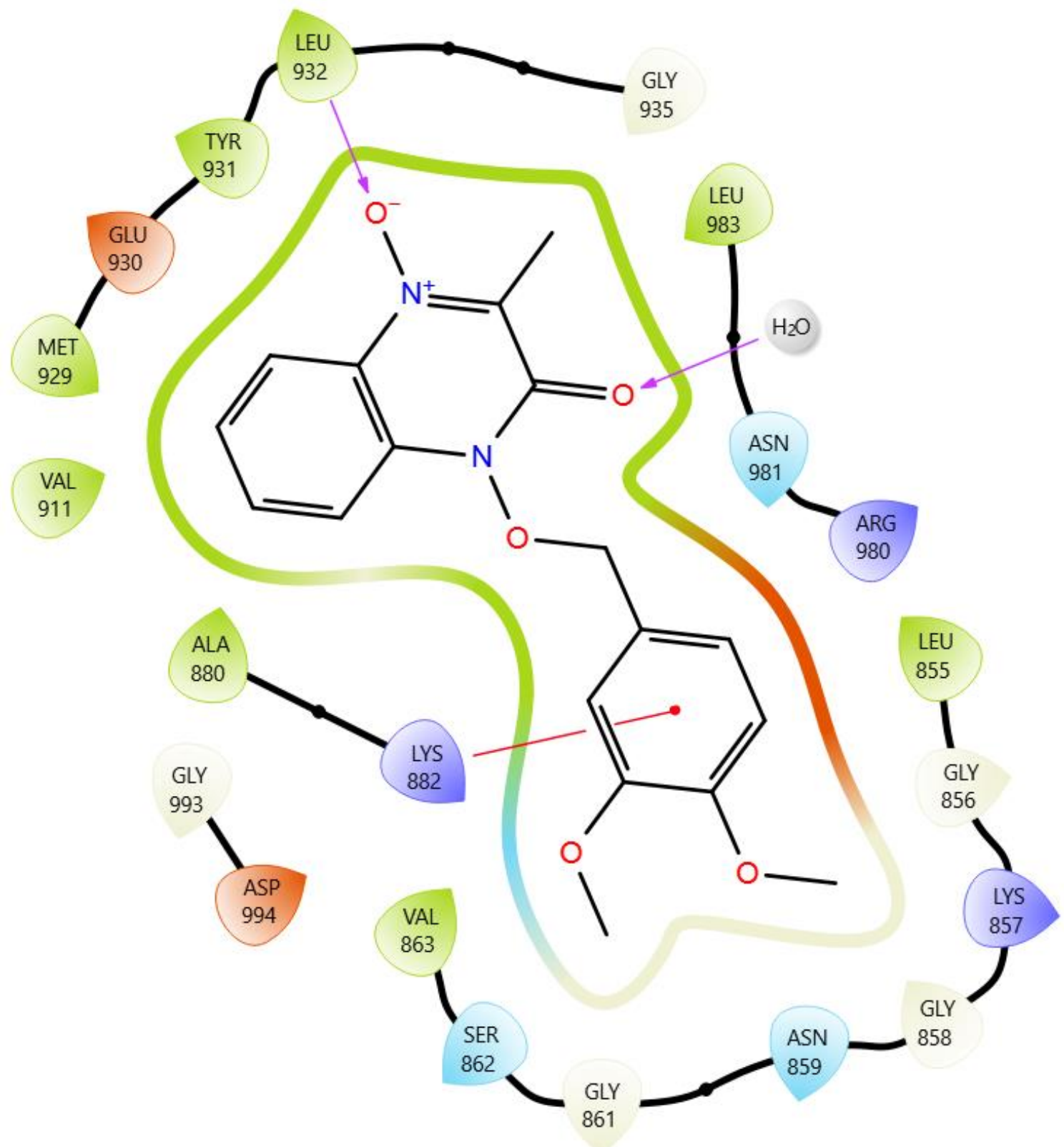

Figure S38. Two-dimensional interaction of STOCK3S-83435 in the active site of JAK2.

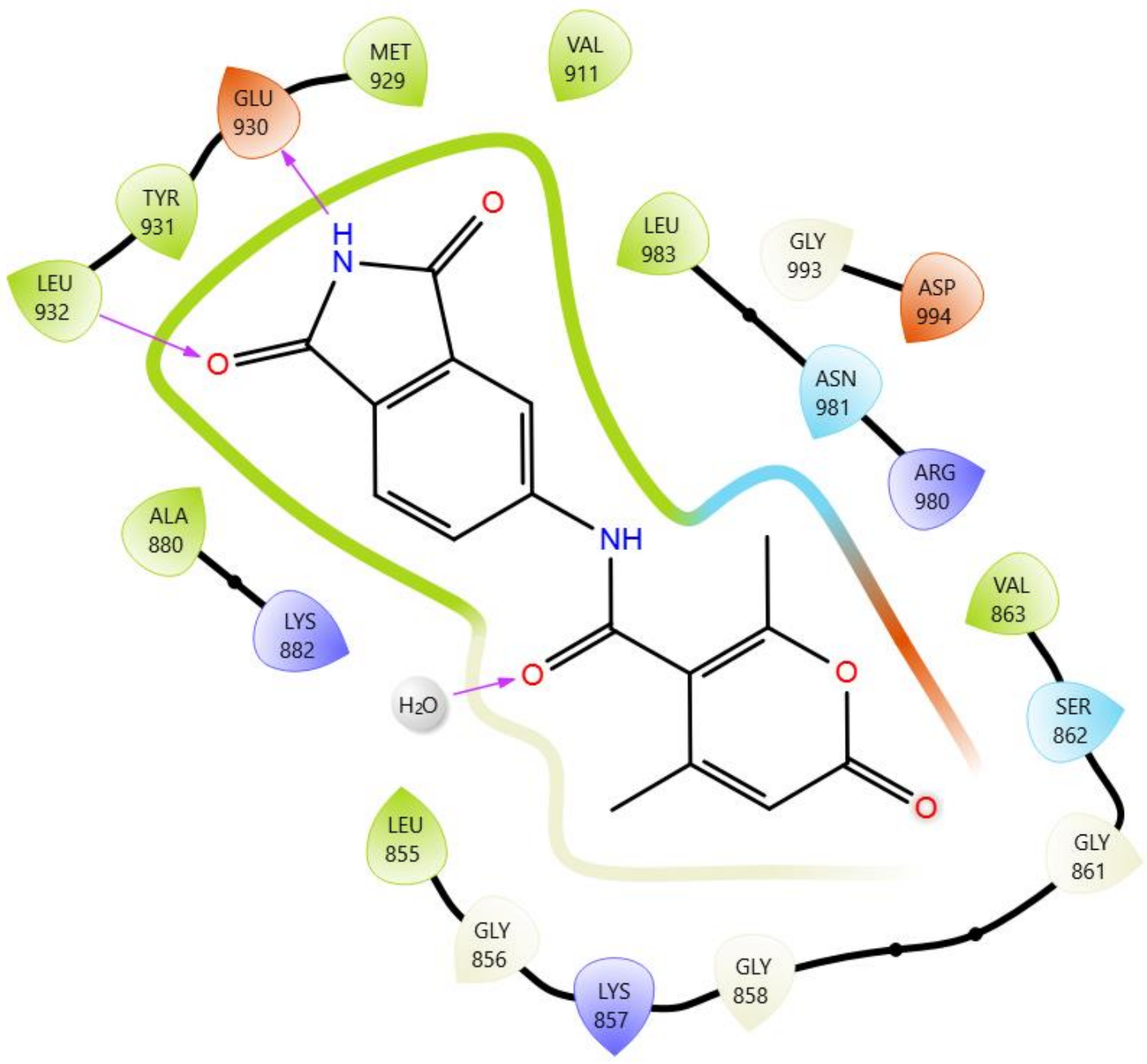

Figure S39. Two-dimensional interaction of STOCK3S-84679 in the active site of JAK2.

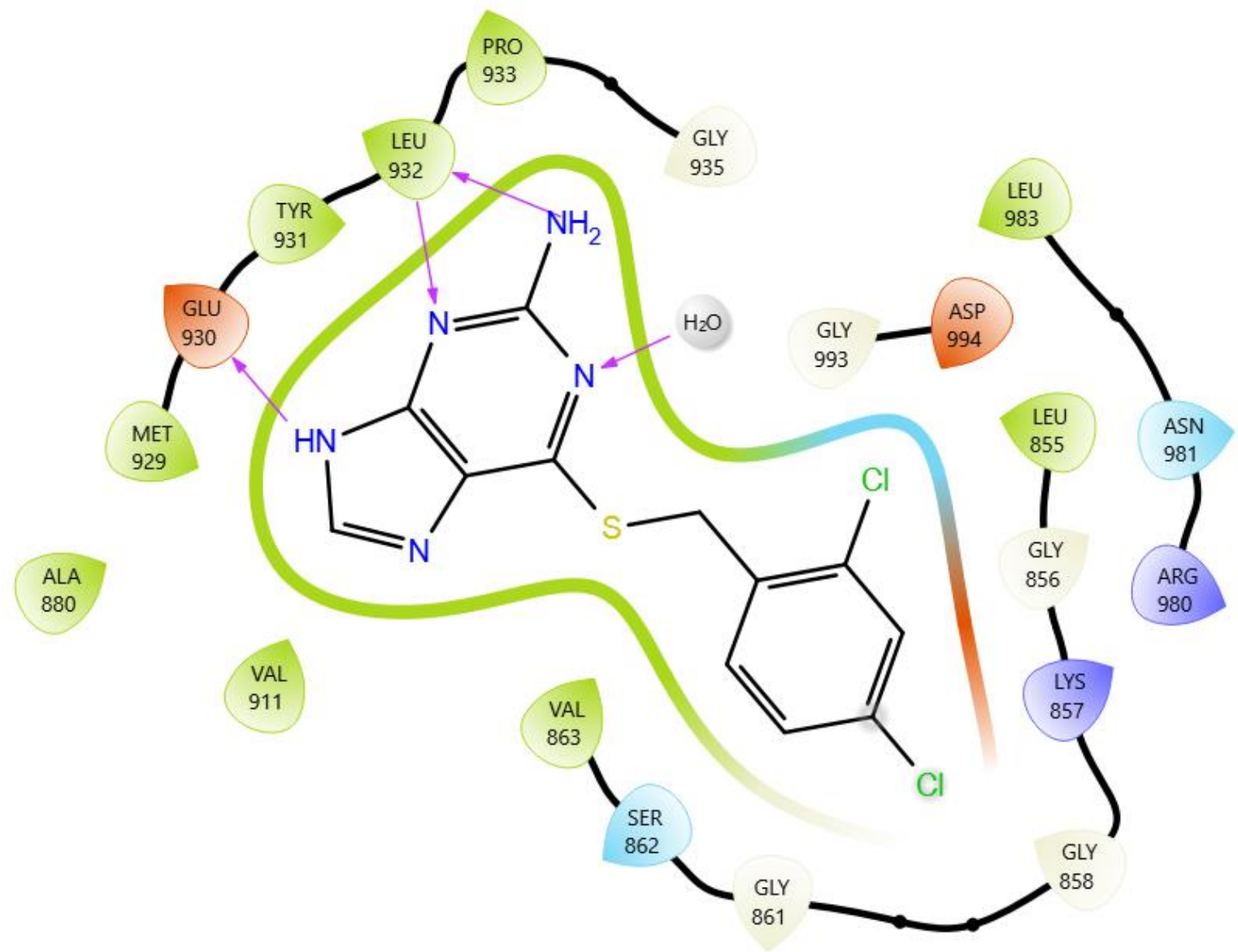

Figure S40. Two-dimensional interaction of STOCK4S-62175 in the active site of JAK2.

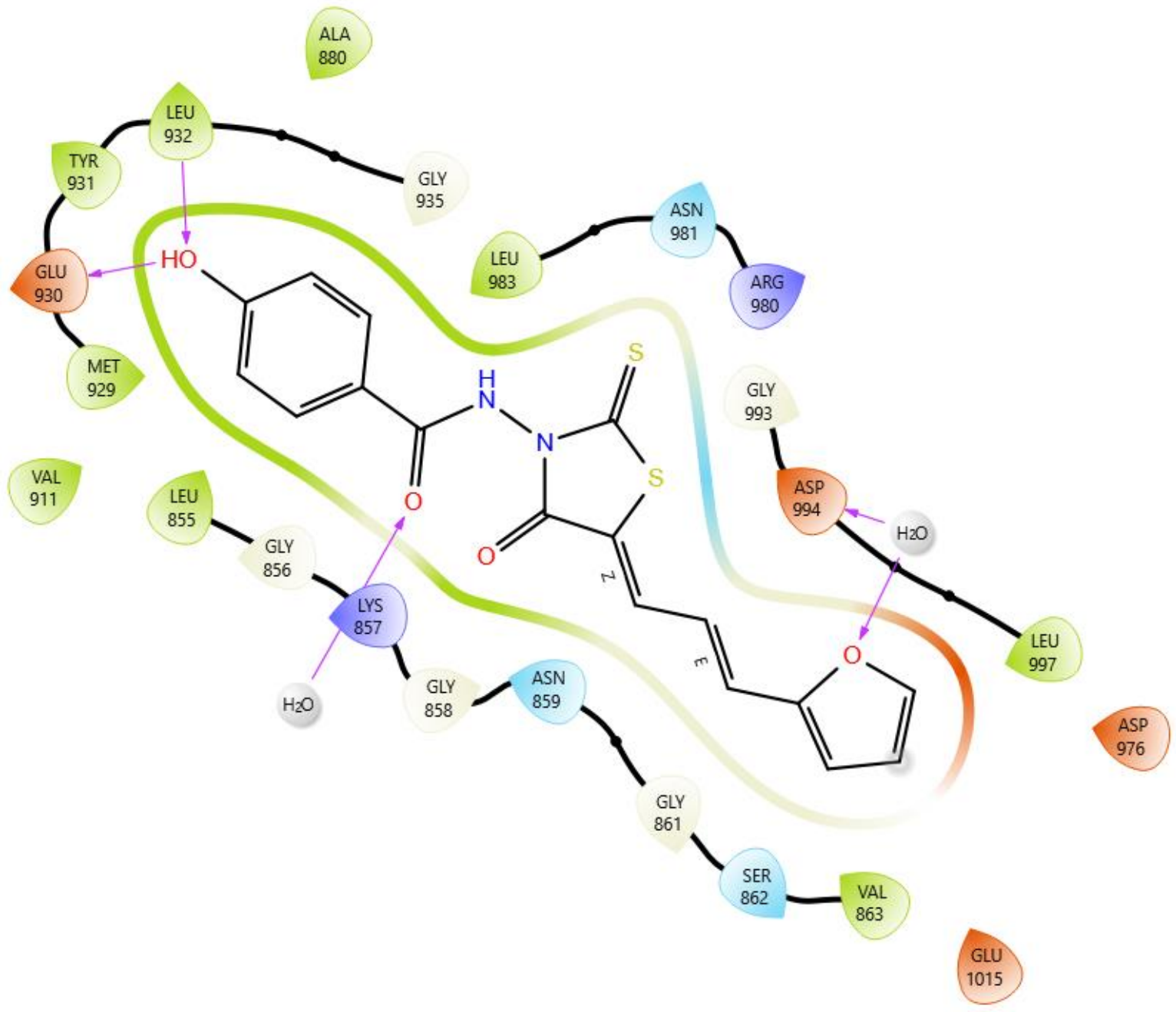

Figure S41. Two-dimensional interaction of STOCK4S-86355 in the active site of JAK2.

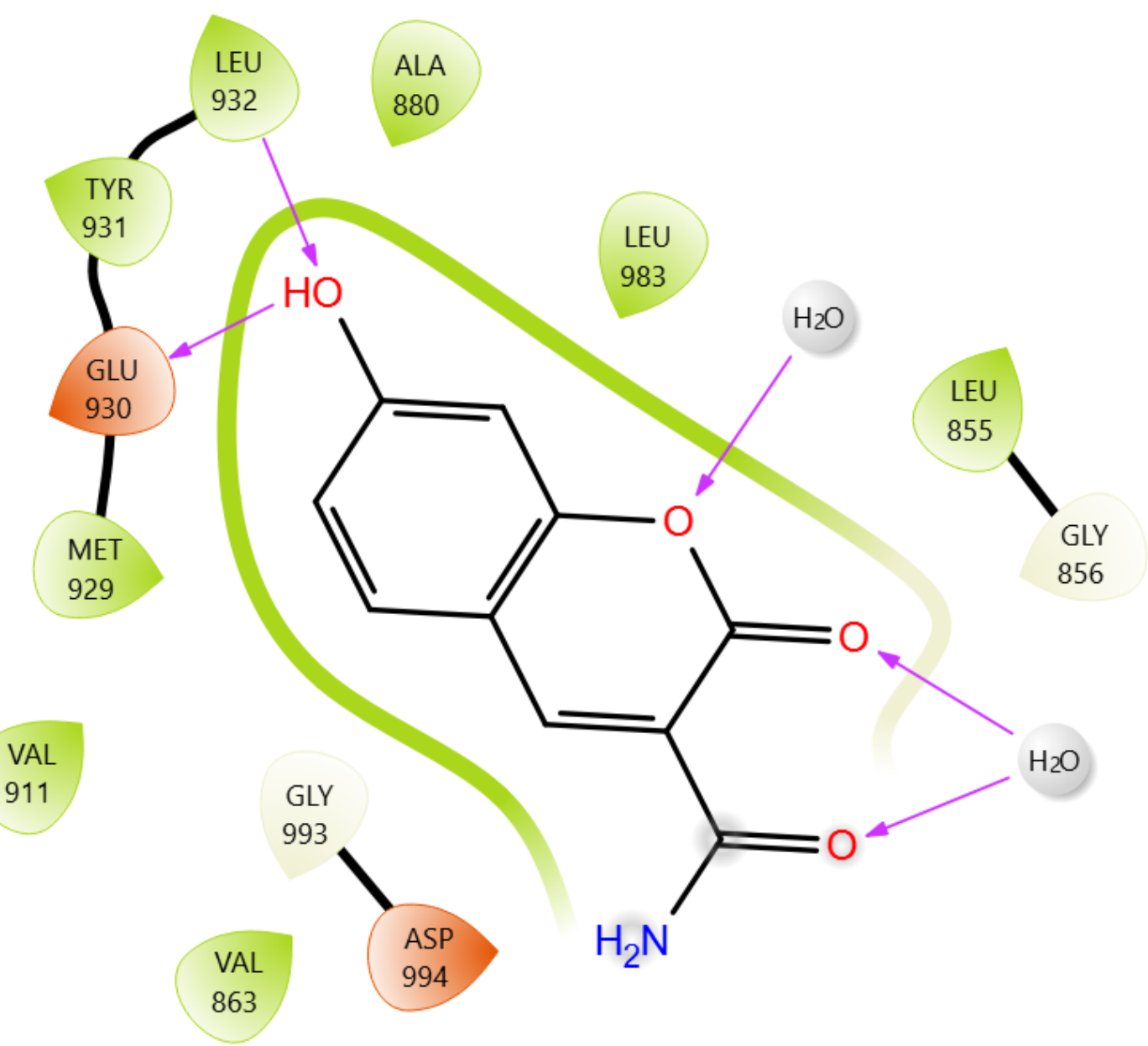

Figure S42. Two-dimensional interaction of STOCK5S-56194 in the active site of JAK2.

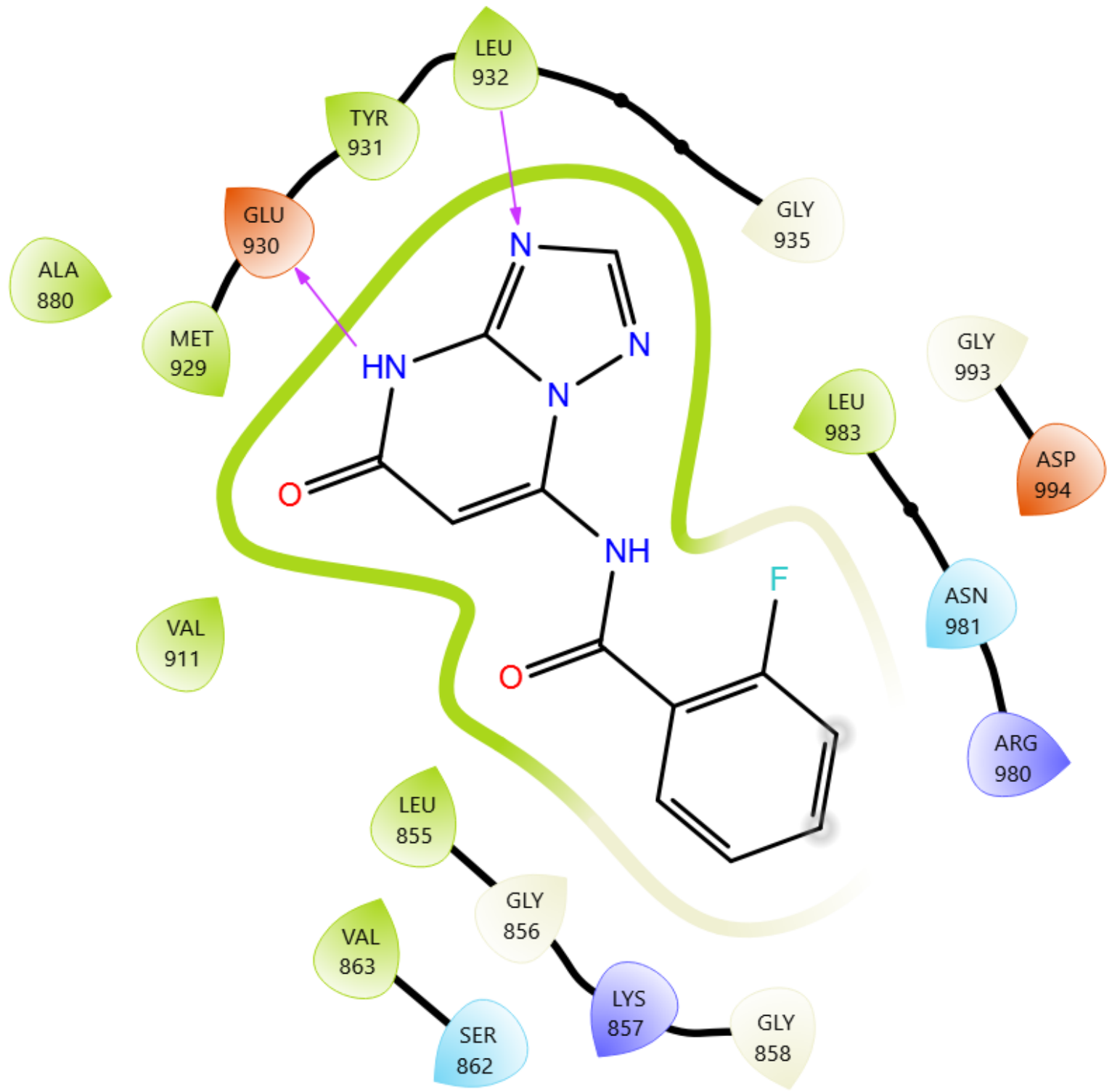

Figure S43. Two-dimensional interaction of STOCK5S-60009 in the active site of JAK2.

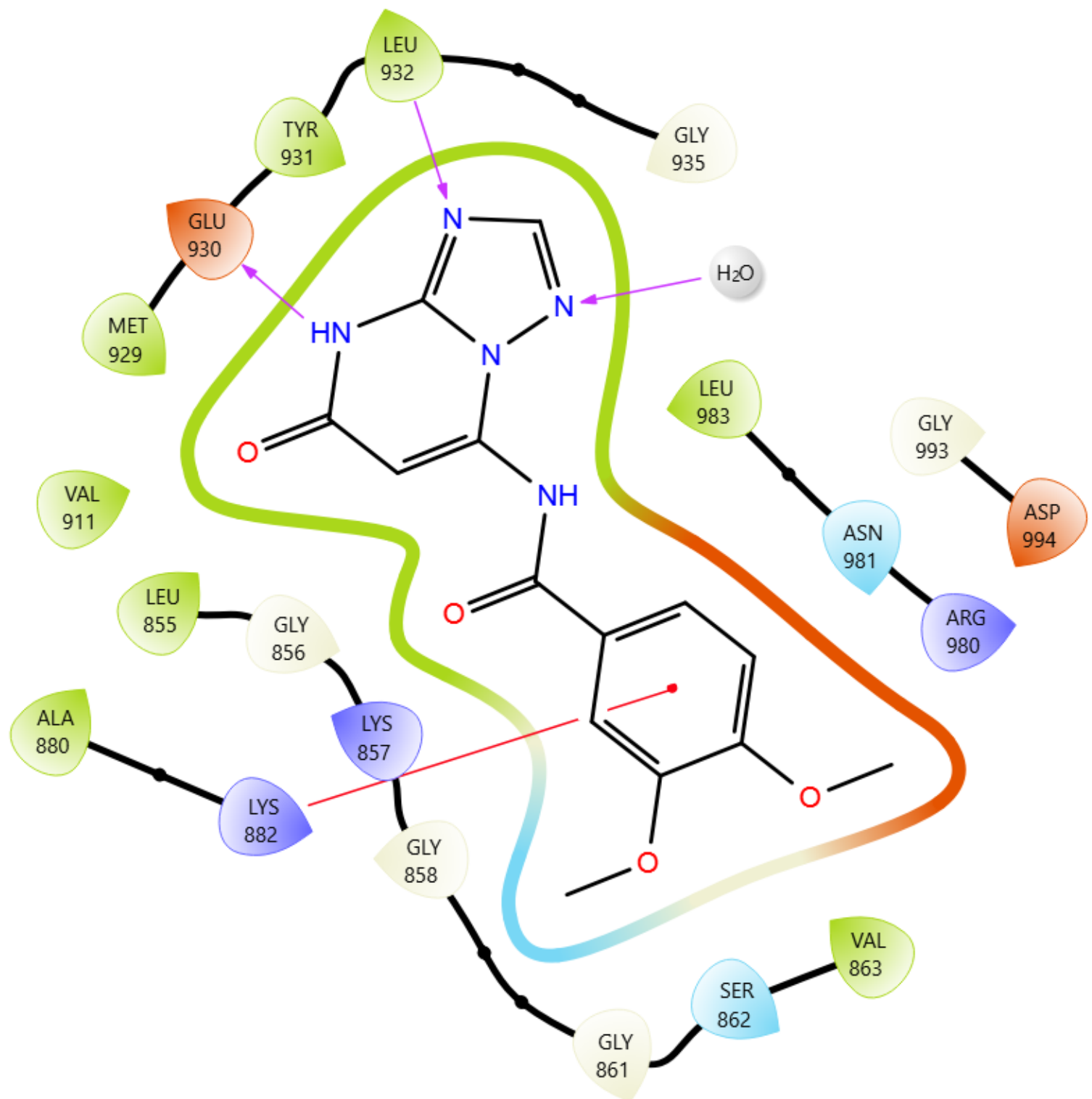

Figure S44. Two-dimensional interaction of STOCK5S-79464 in the active site of JAK2.

4. Table S1. Outcome compounds expected to exhibit JAK1 inhibition, along with their predicted binding energies.

Compound	Stock Code	Chemical formula	Binding Energy (kcal/mol)
**1-1**	STOCK1N-12057	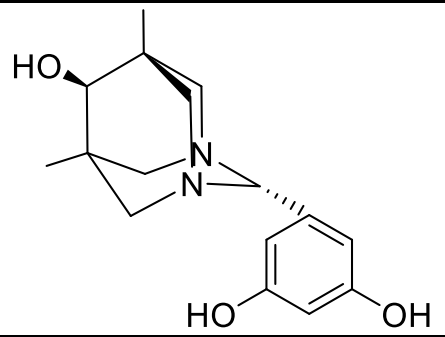	**-8,905**
**1-2**	STOCK1N-31772	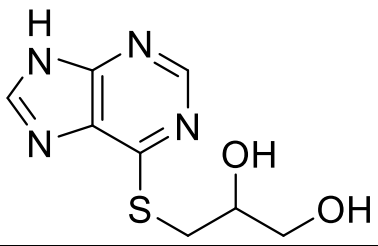	**-9,310**
**1-3**	STOCK1N-51560	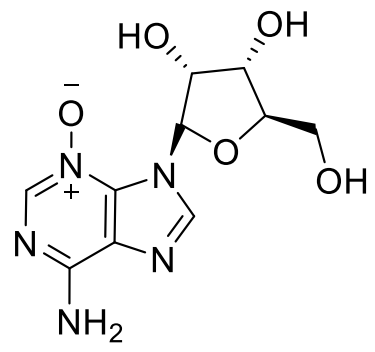	**-9,042**
**1-4**	STOCK1N-69616	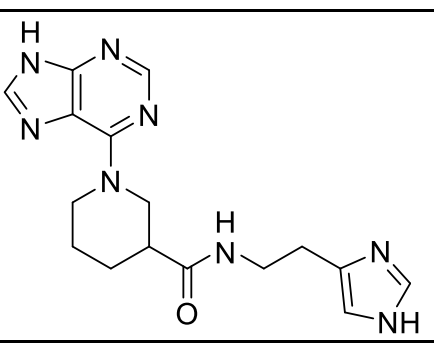	**-9,124**
**1-5**	STOCK3S-84679	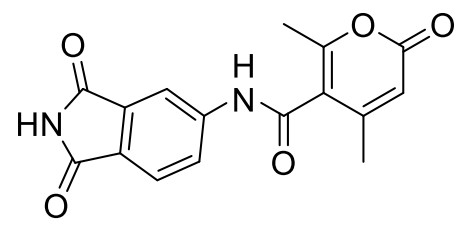	**-8,827**
**1-6**	STOCK1N-77176	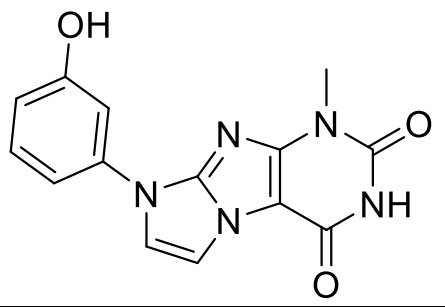	**-8,990**
**1-7**	STOCK1N-79356	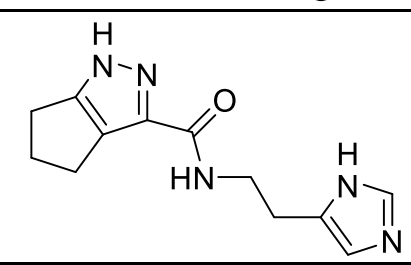	**-9,464**
**1-8**	STOCK1N-97980	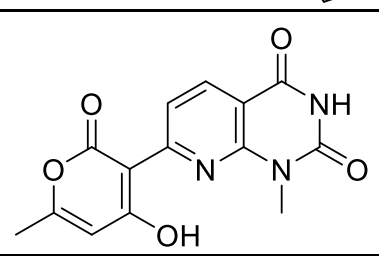	**-8,842**
**1-9**	STOCK2S-29689	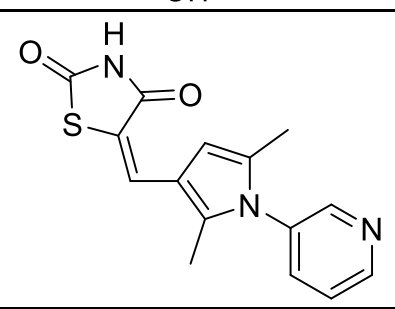	**-9,564**
**1-10**	STOCK2S-34232	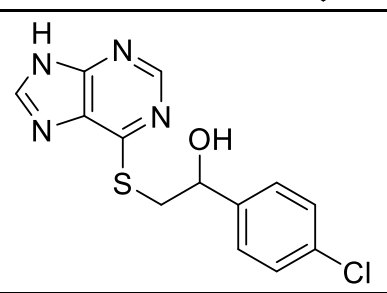	**-9,257**
**1-11**	STOCK4S-15629	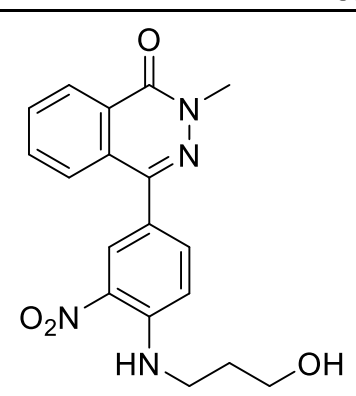	**-8,854**

**1-12**	STOCK4S-46885	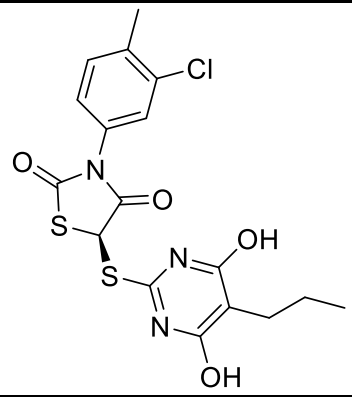	**-9,162**
**1-13**	STOCK4S-70675	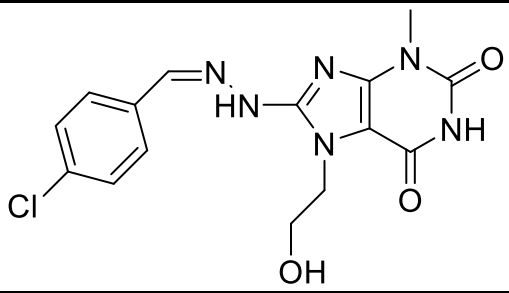	**-8,888**
**1-14**	STOCK5S-74239	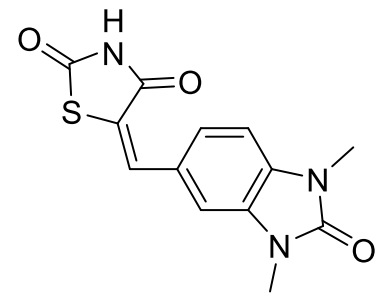	**-8,957**
**1-15**	STOCK5S-83560	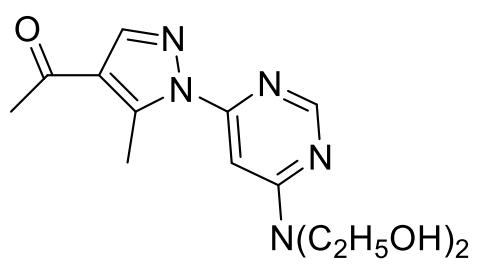	**-8,738**
**1-16**	STOCK6S-28538	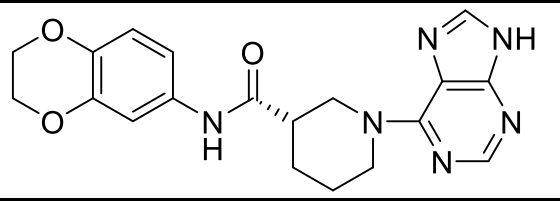	**-9,655**
**1-17**	STOCK6S-72898	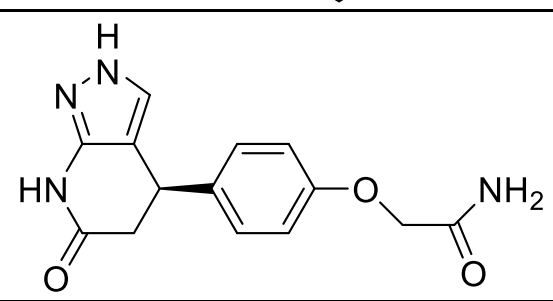	**-10,161**
**1-18**	STOCK6S-83203	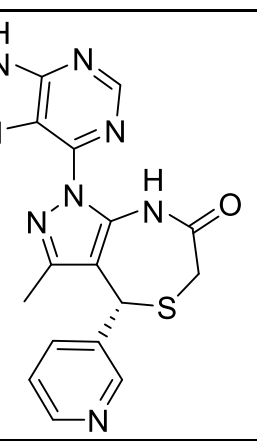	**-9,232**
**1-19**	STOCK7S-44229	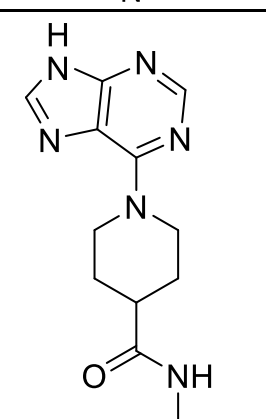	**-9,384**
**1-20**	STOCK7S-51860	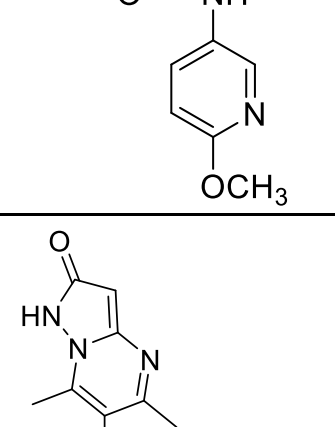	**-9,027**
**1-21**	STOCK7S-53671	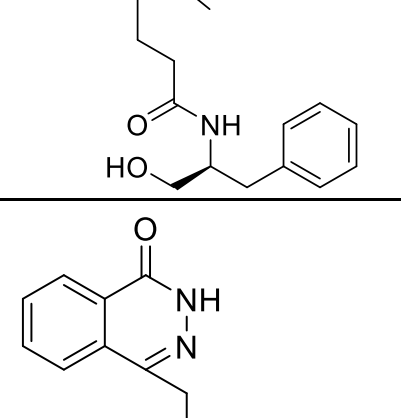	**-9,574**

5. Table S2. Compounds predicted to exhibit JAK2 inhibitory activity and their calculated binding energies

Compound	Stock Code	Chemical formula	Binding Energy (kcal/mol)
**2-1**	STOCK1S-00944	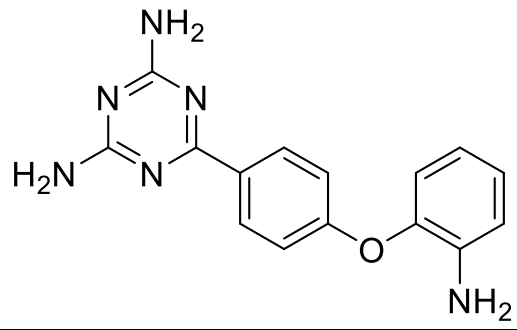	**-9,308**
**2-2**	STOCK1S-06278	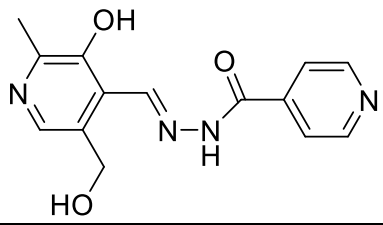	**-9,326**
**2-3**	STOCK1S-10380	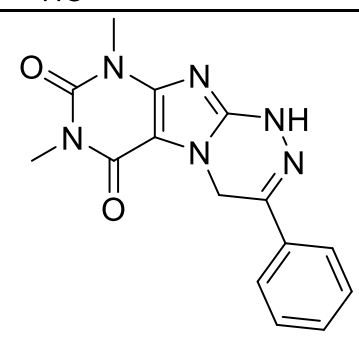	**-9,544**
**2-4**	STOCK1S-69008	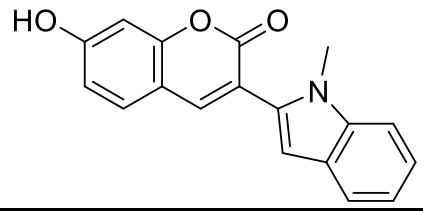	**-9,845**
**2-5**	STOCK1S-78591	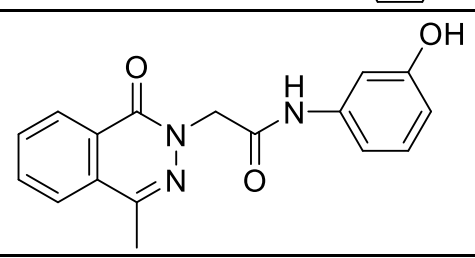	**-9,318**
**2-6**	STOCK2S-08448	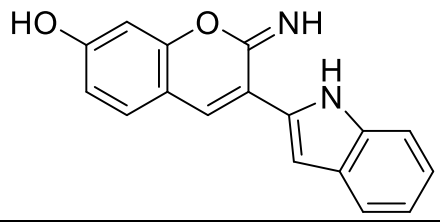	**-9,707**
**2-7**	STOCK2S-18844	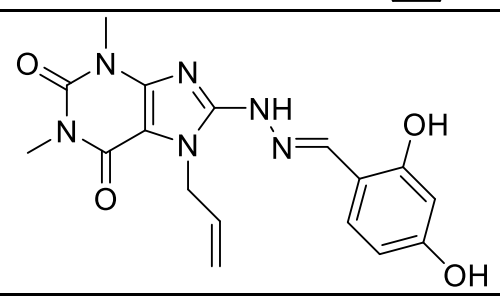	**-10,226**
**2-8**	STOCK2S-34232	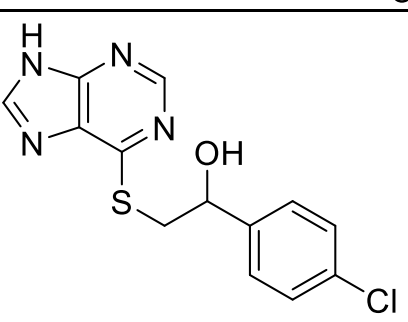	**-10,875**
**2-9**	STOCK2S-52524	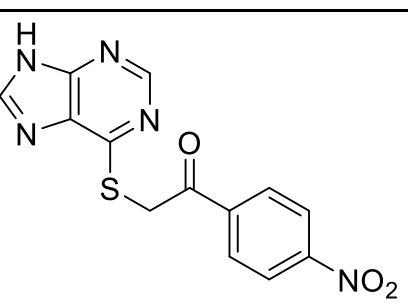	**-10,287**
**2-10**	STOCK2S-54227	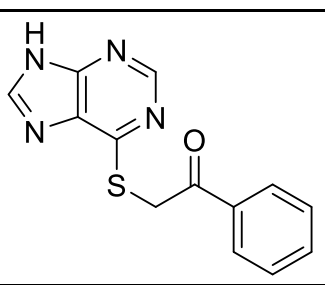	**-10,390**
**2-11**	STOCK2S-60777	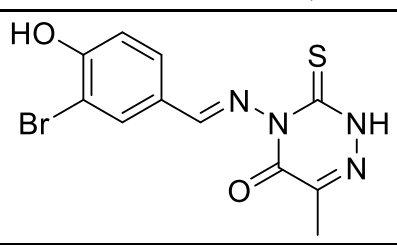	**-9,101**
**2-12**	STOCK2S-75169	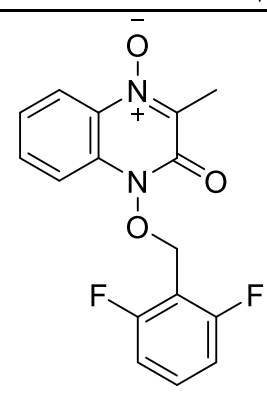	**-9,764**

**2-13**	STOCK3S-18016	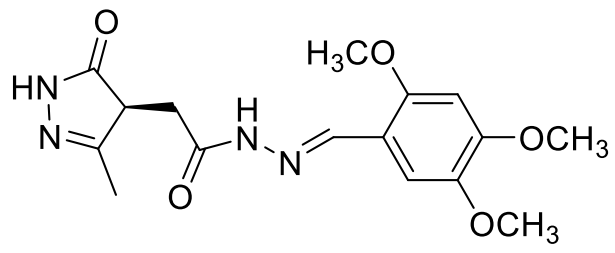	**-9,291**
**2-14**	STOCK3S-18468	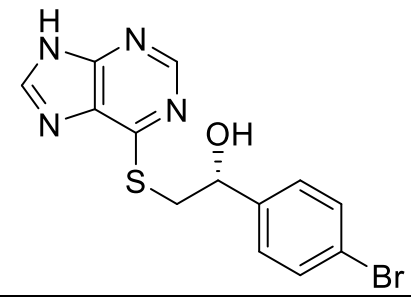	**-10,506**
**2-15**	STOCK3S-83435	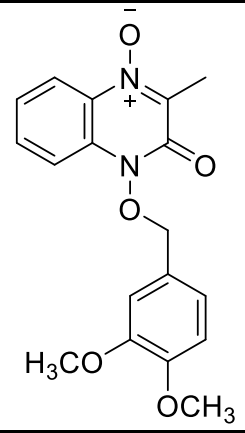	**-9,773**
**2-16**	STOCK1N-71510	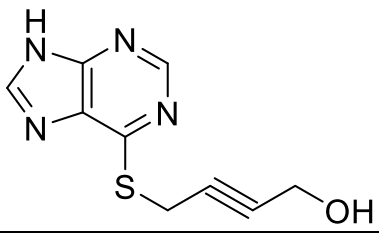	**-9,739**
**2-17**	STOCK4S-62175	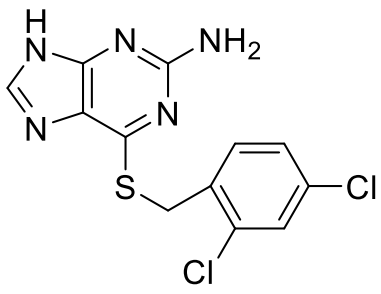	**-10,417**
**2-18**	STOCK4S-86355	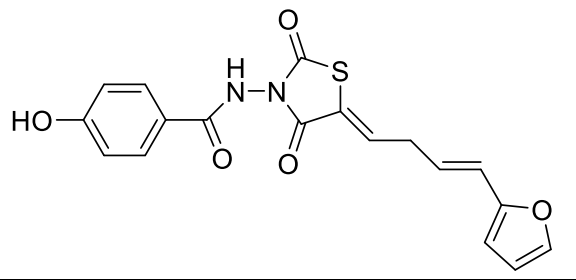	**-9,242**
**2-19**	STOCK5S-56194	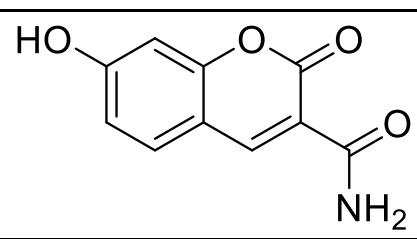	**-9,160**
**2-20**	STOCK5S-60009	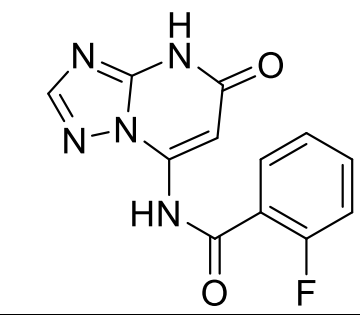	**-9,497**
**2-21**	STOCK5S-79464	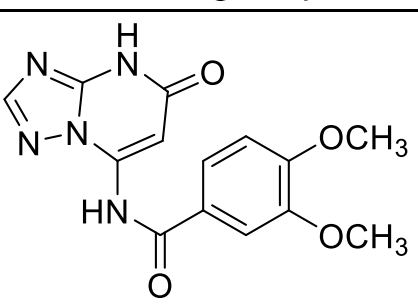	**-9,557**

6. Superposed image of the ligands prepared for JAK1 RMSD calculation

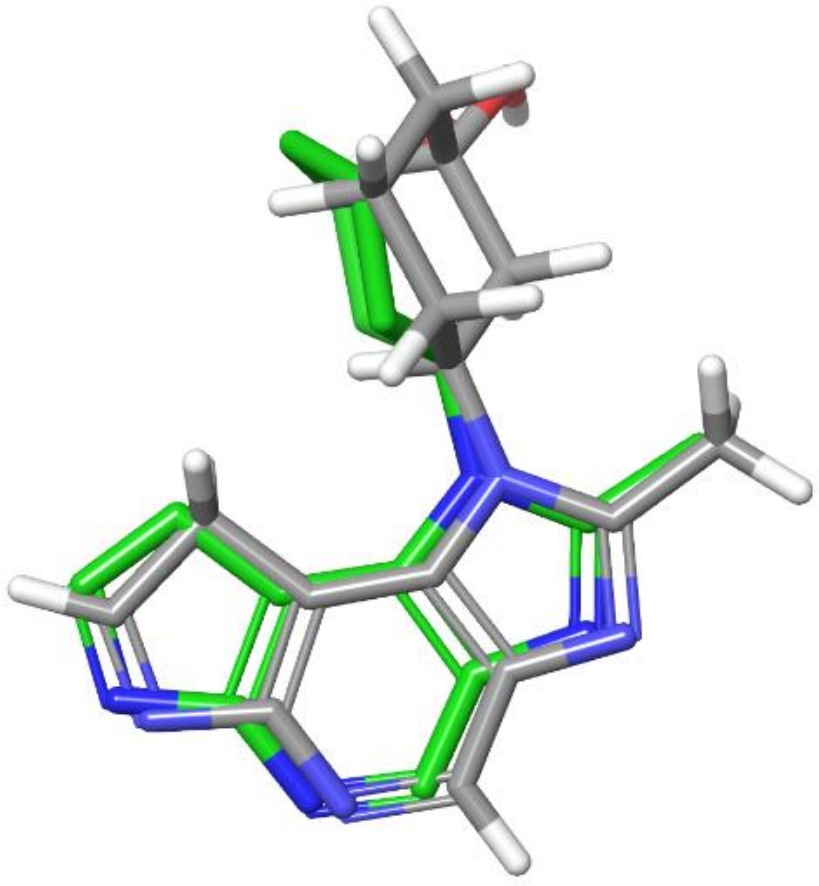

Figure S45. Superposed image of the ligands prepared for JAK1 RMSD calculation. The native ligand is colored green, and the prepared ligand is colored gray.

7. Superposed image of the ligands prepared for JAK2 RMSD calculation

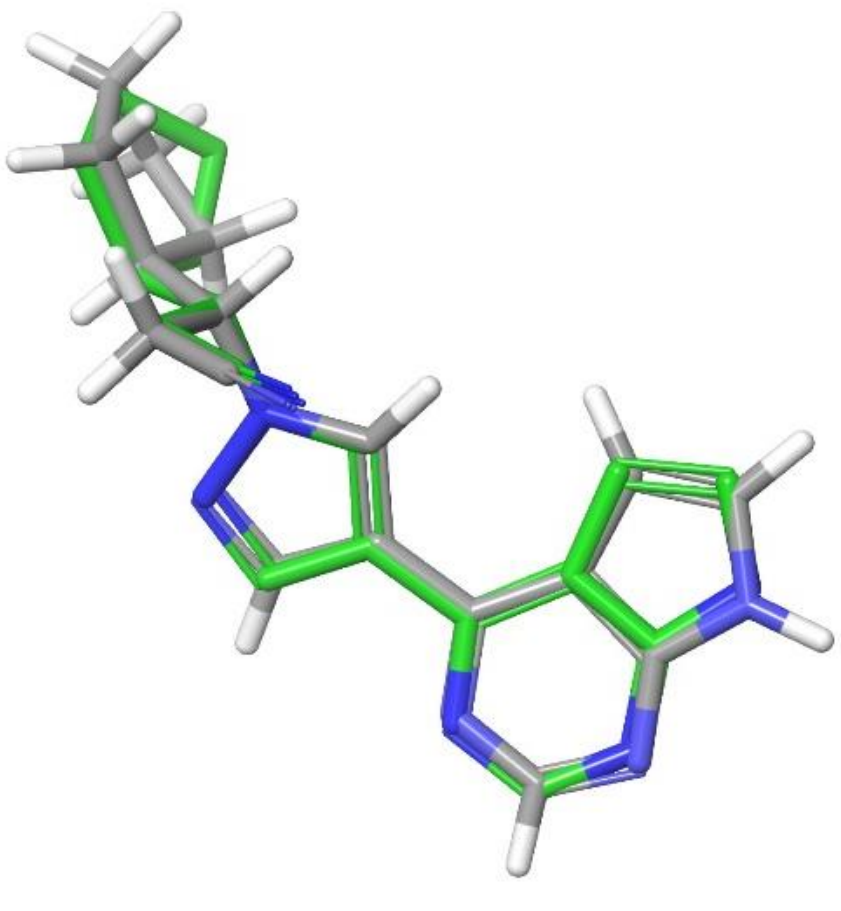

Figure S46. Superposed image of the ligands prepared for JAK2 RMSD calculation. The native ligand is colored green, and the prepared ligand is colored gray.

8. Two-dimensional (2D) and three-dimensional (3D) interaction profiles of compounds 1–4 (B), 1–6 (C), 1–10 (D), and 1–19 (E) within the active site of JAK1 (PDB ID: 4EI4).

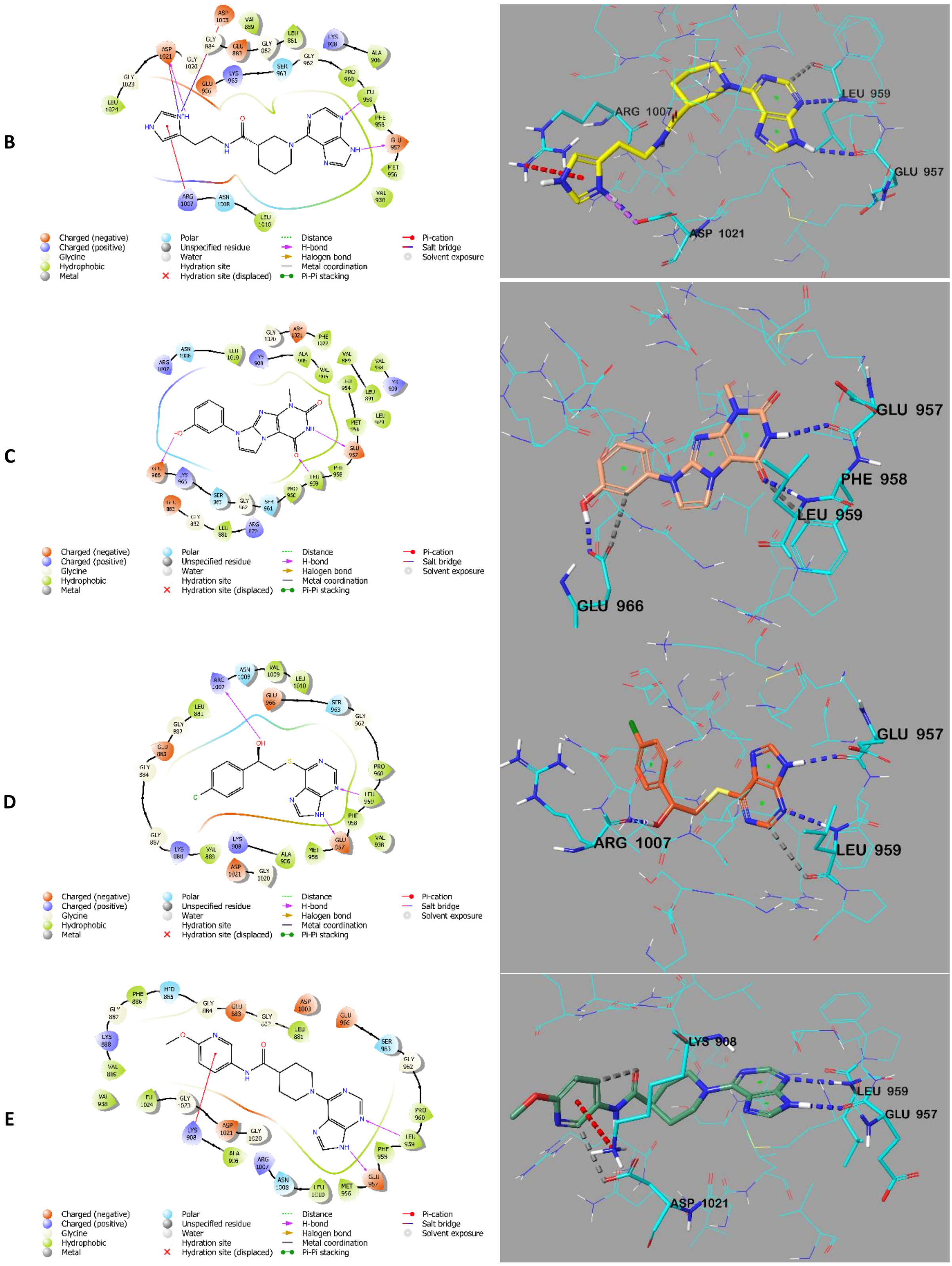

Figure S47. Two-dimensional (2D) and three-dimensional (3D) interaction profiles of compounds 1–4 (B), 1–6 (C), 1–10 (D), and 1–19 (E) within the active site of JAK1 (PDB ID: 4EI4)

9. Two-dimensional (2D) and three-dimensional (3D) interaction profiles of compounds 2–9 (B), 2–10 (C), 2–14 (D), and 2–16 (E) within the active site of JAK2 (PDB ID: 6VGL).

B

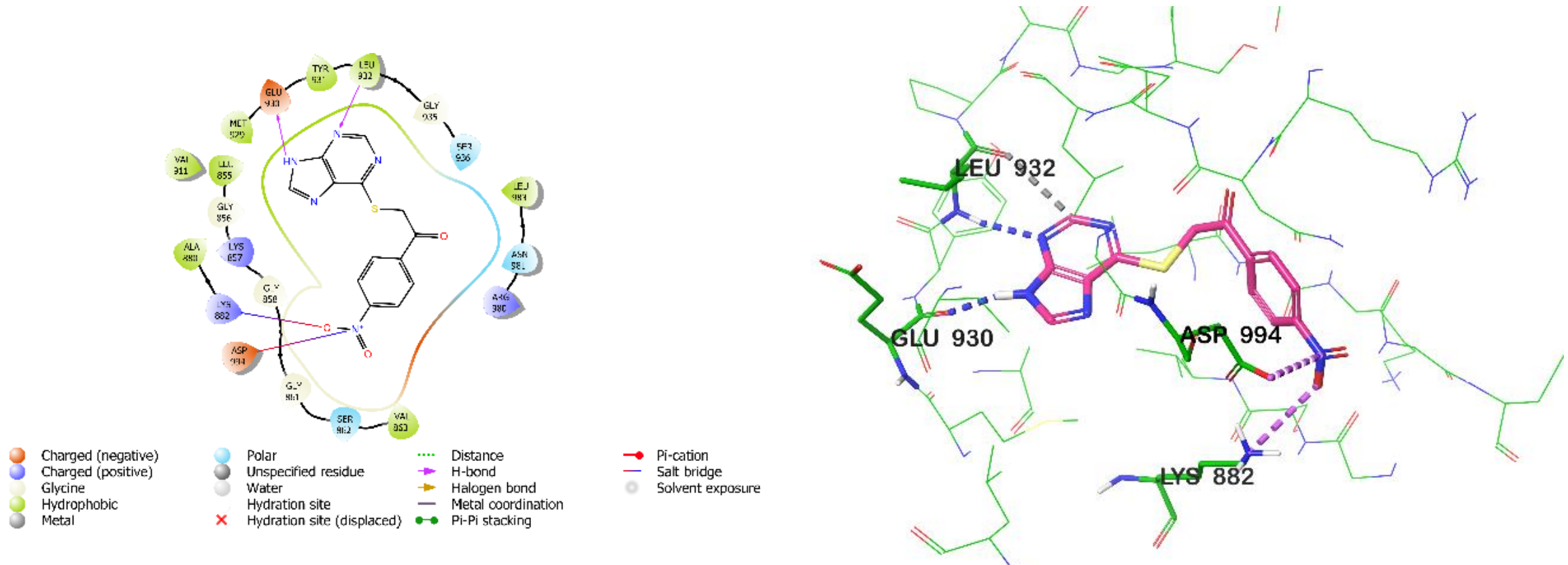

C

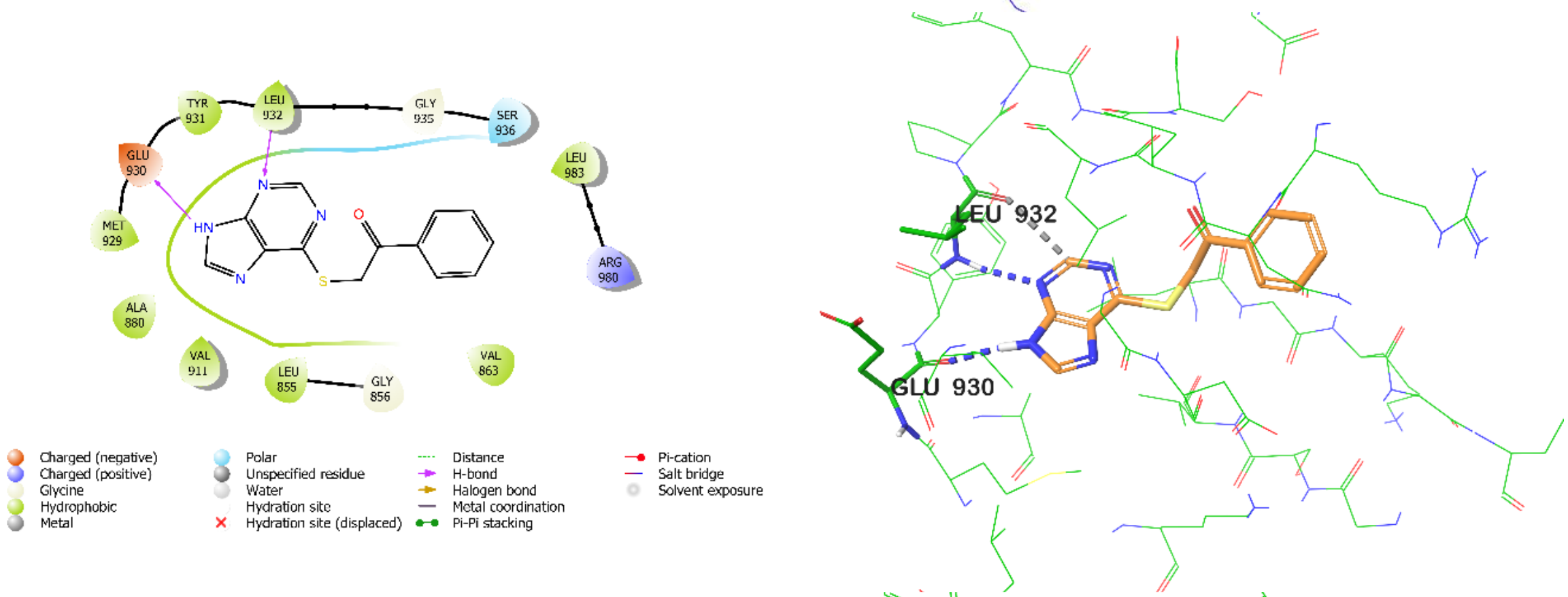

D

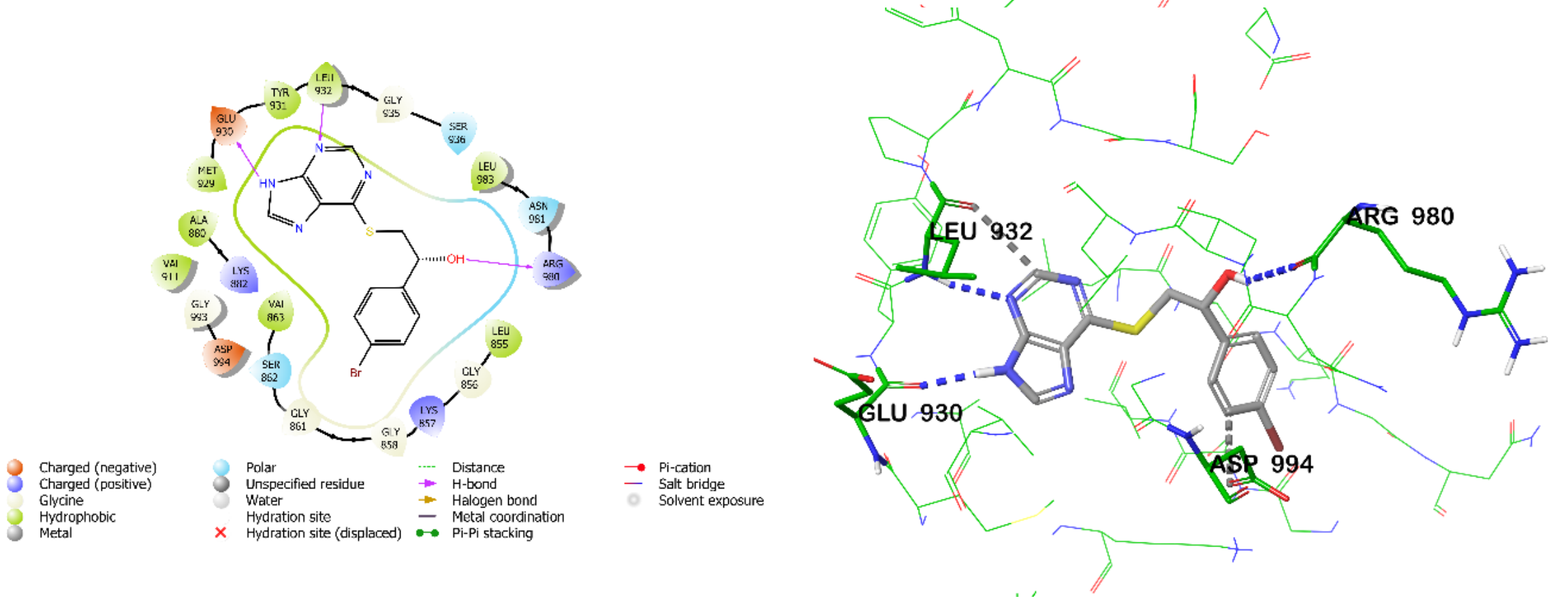

E

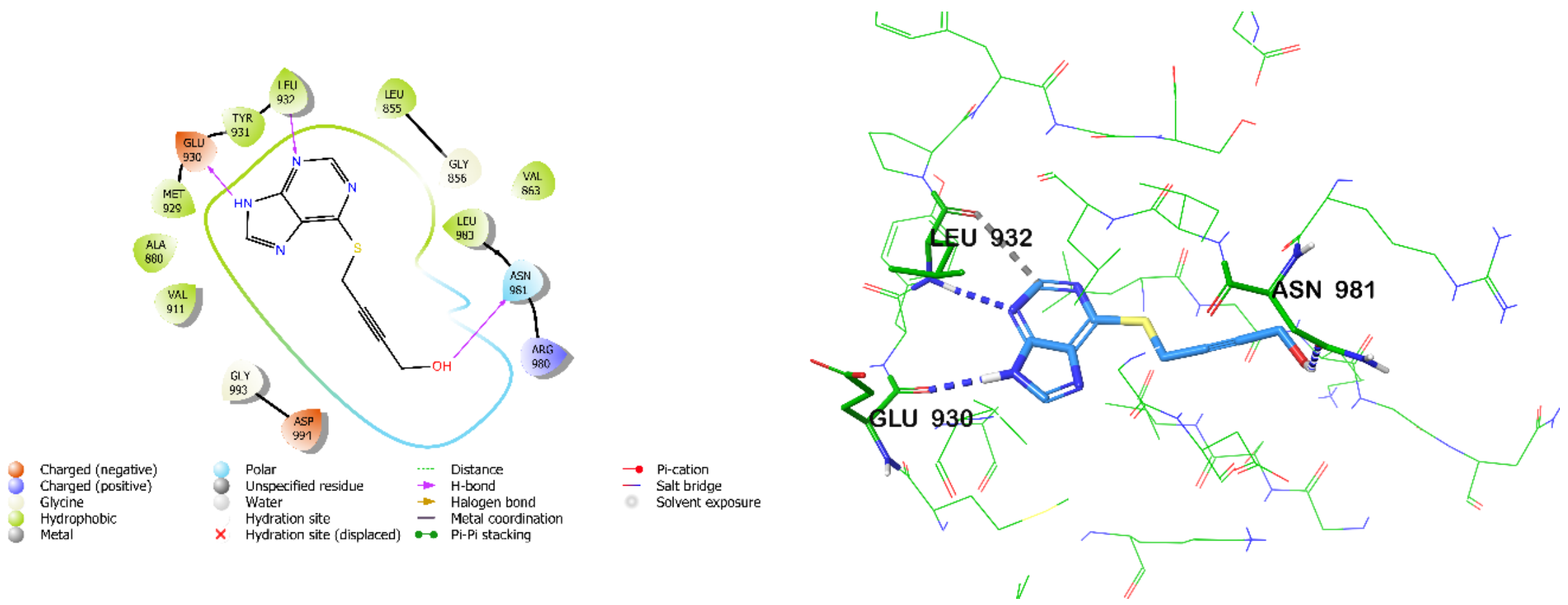

Figure S48. Two-dimensional (2D) and three-dimensional (3D) interaction profiles of compounds **2–9** (**B**), **2–10** (**C**), **2–14** (**D**), and **2–16** (**E**) within the active site of JAK2 (PDB ID: 6VGL).

10. Statistical Validation and Replica Simulations

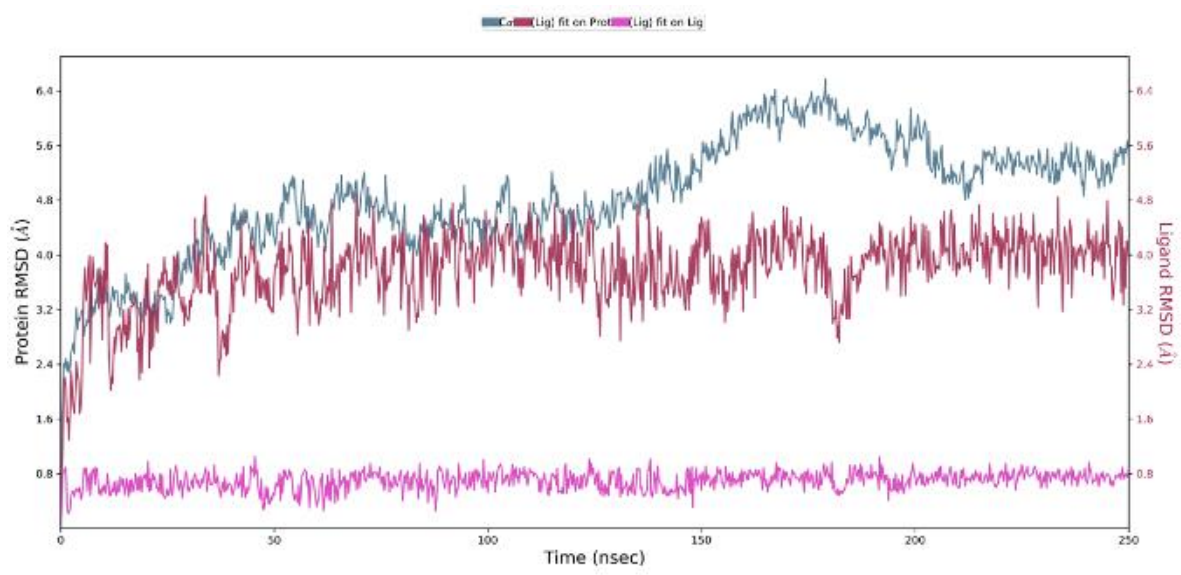

Compound **1-3** in complex with JAK1 (1)

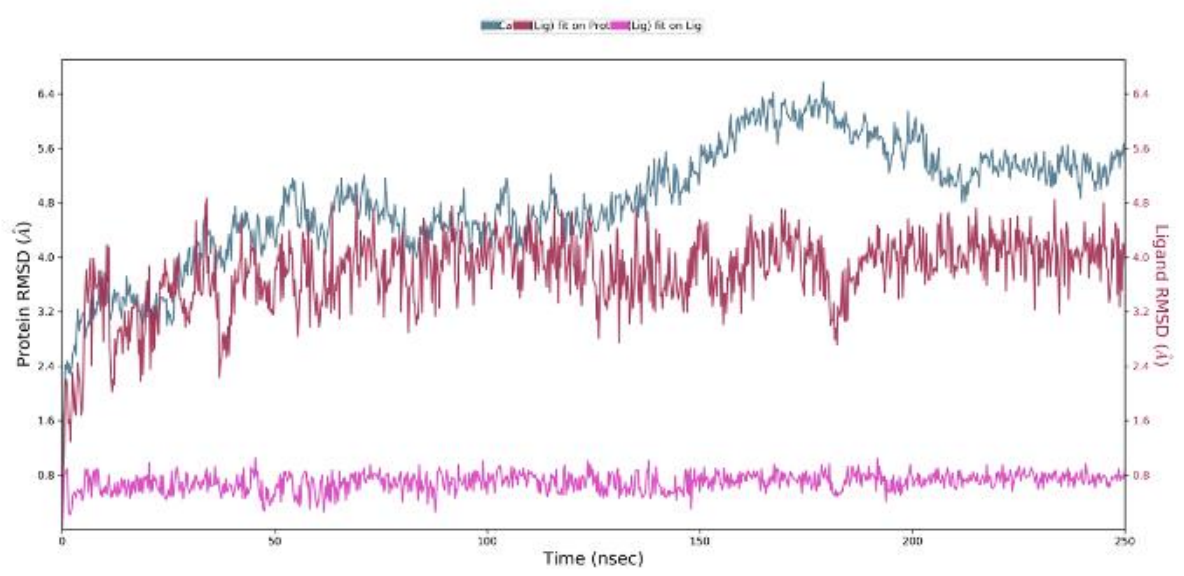

Compound **1-3** in complex with JAK1 (2)

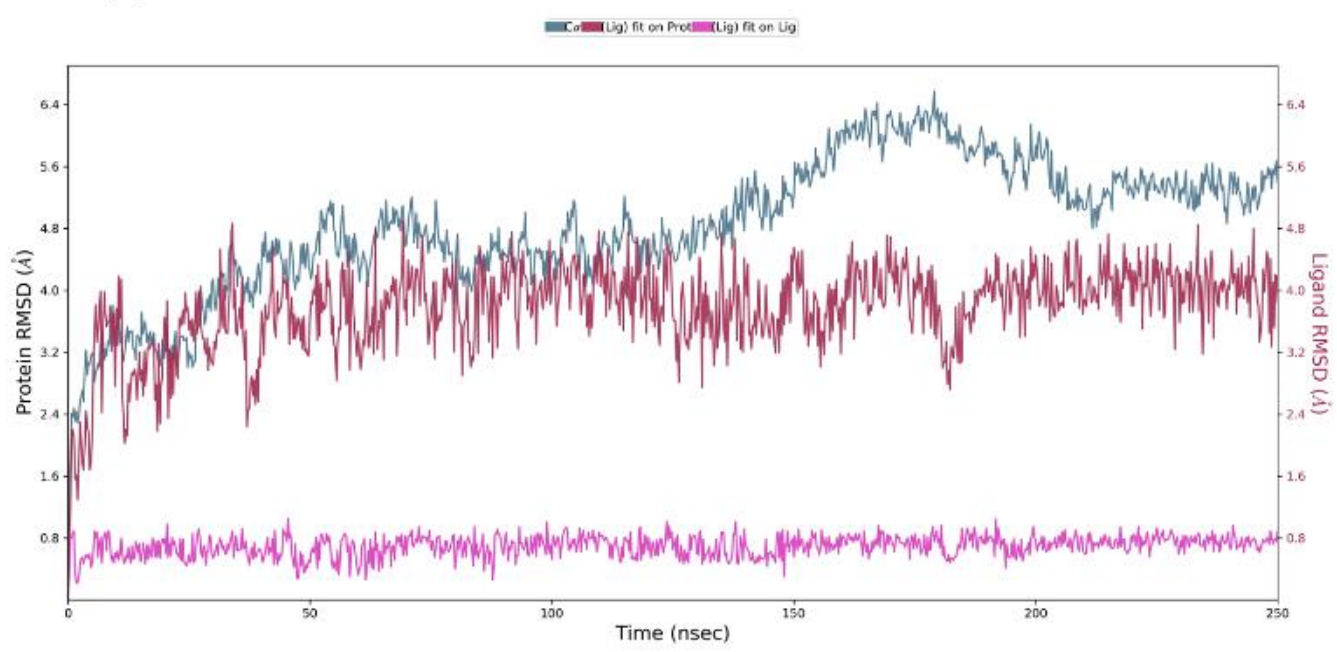

Compound **1-3** in complex with JAK1 (3)

Figure S49: The RMSD plots of compound **1-3** with the JAK1 protein as a function of simulation time (0 to 250 ns) captured across independent triplicate production runs (Replica 1, Replica 2, and Replica 3) in complex with JAK1 (PDB ID: 4EI4).

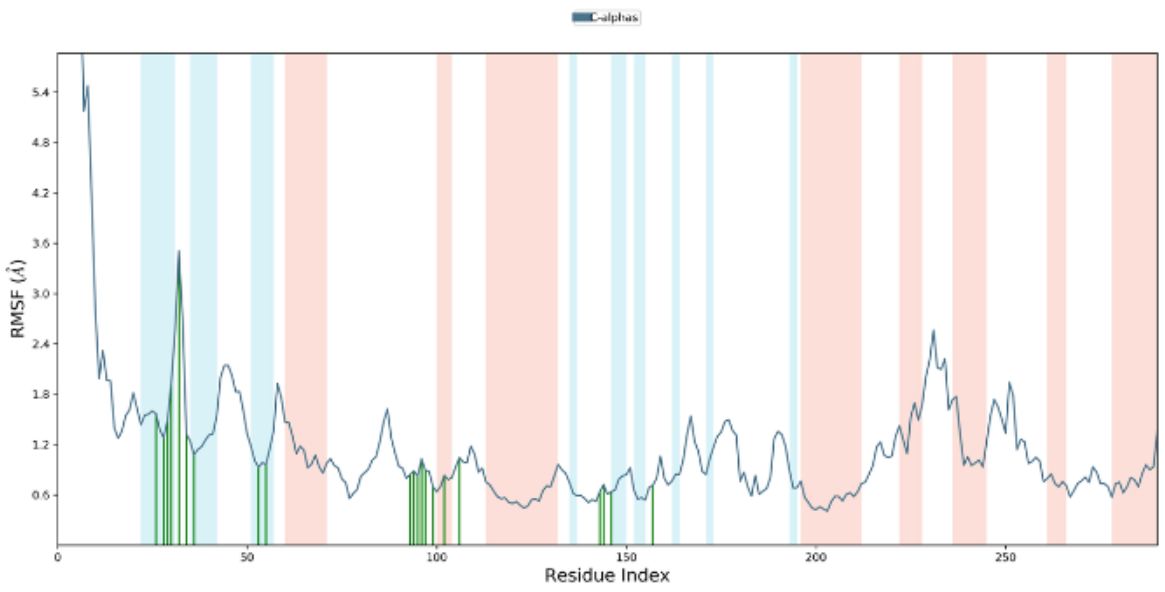

Compound **1-3** in complex
with JAK1 (1)

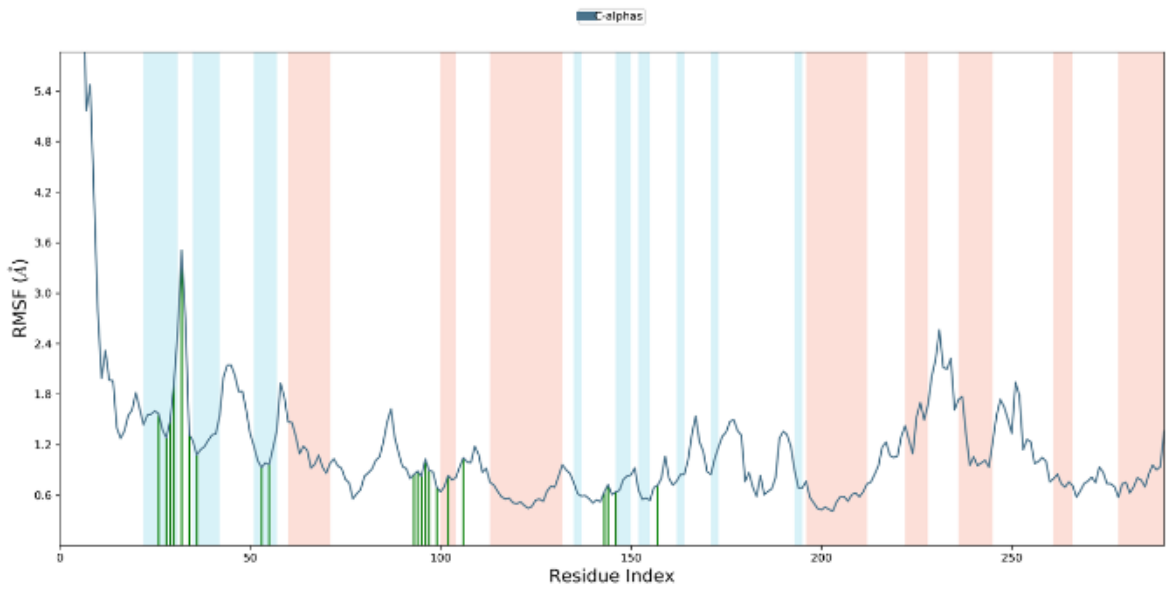

Compound **1-3** in complex
with JAK1 (2)

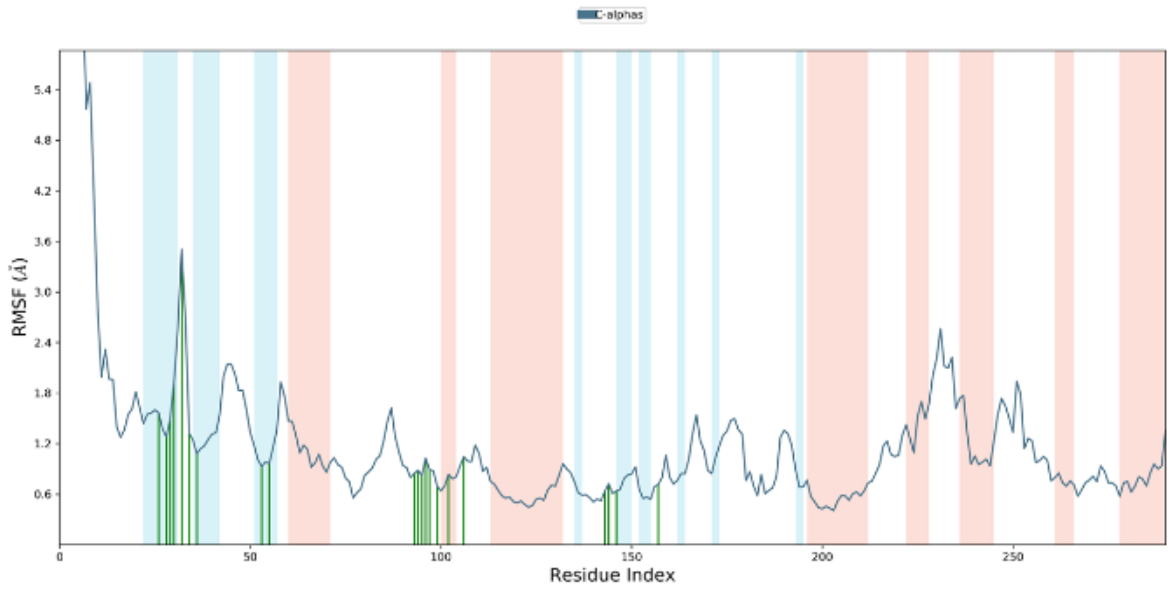

Compound **1-3** in complex
with JAK1 (3)

Figure S50: The RMSF profiles compound **1-3** with the JAK1 protein captured across independent triplicate production runs (Replica 1, Replica 2, and Replica 3) in complex with JAK1 (PDB ID: 4EI4).

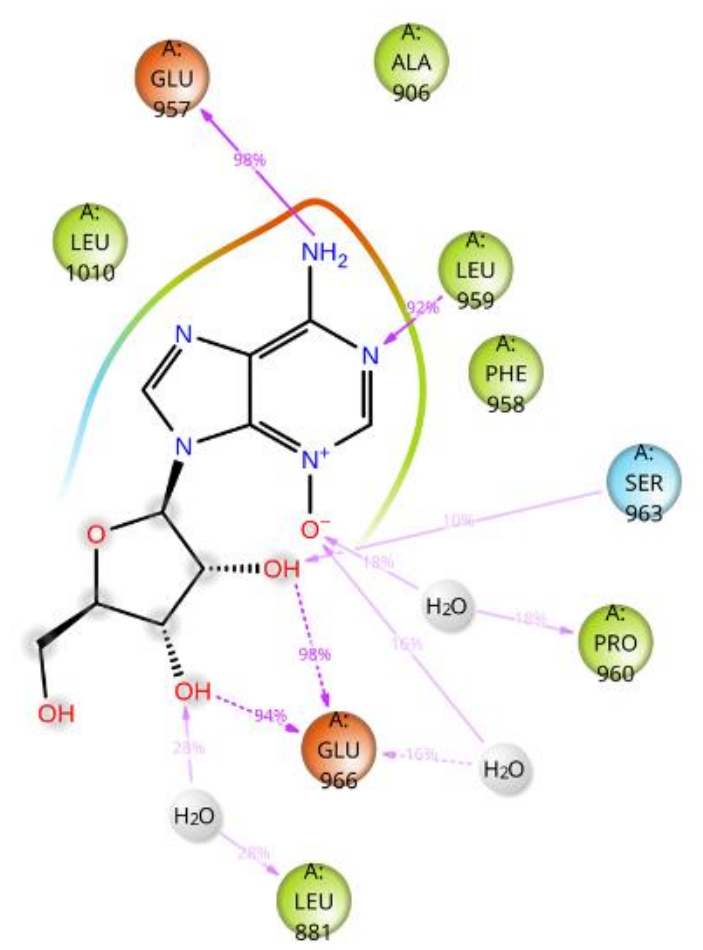

Compound **1-3** in complex
with JAK1 (1)

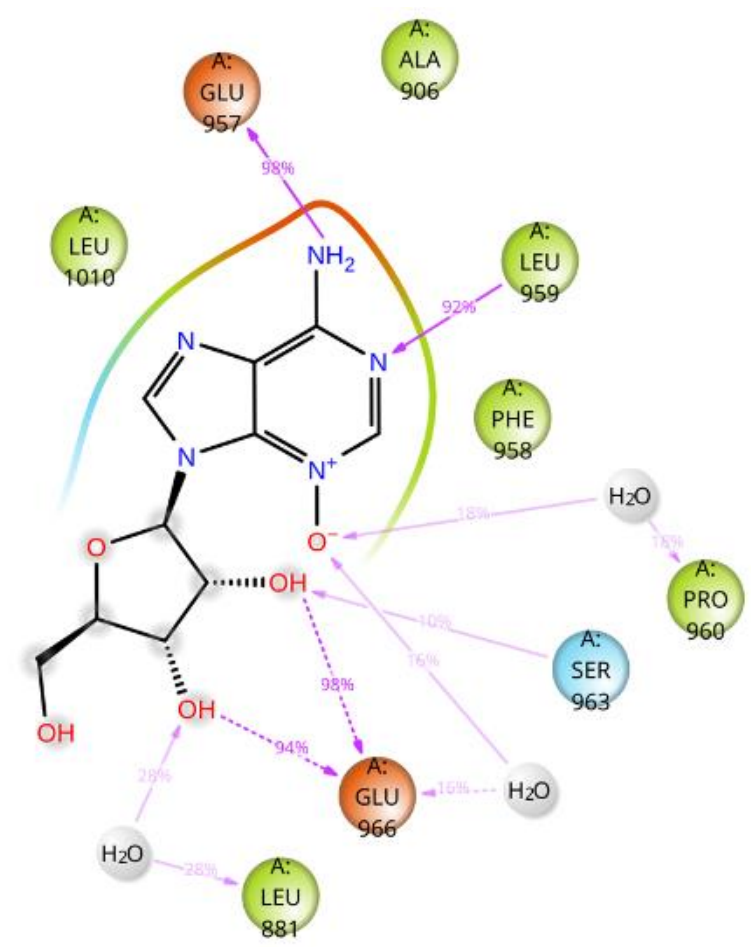

Compound **1-3** in complex
with JAK1 (2)

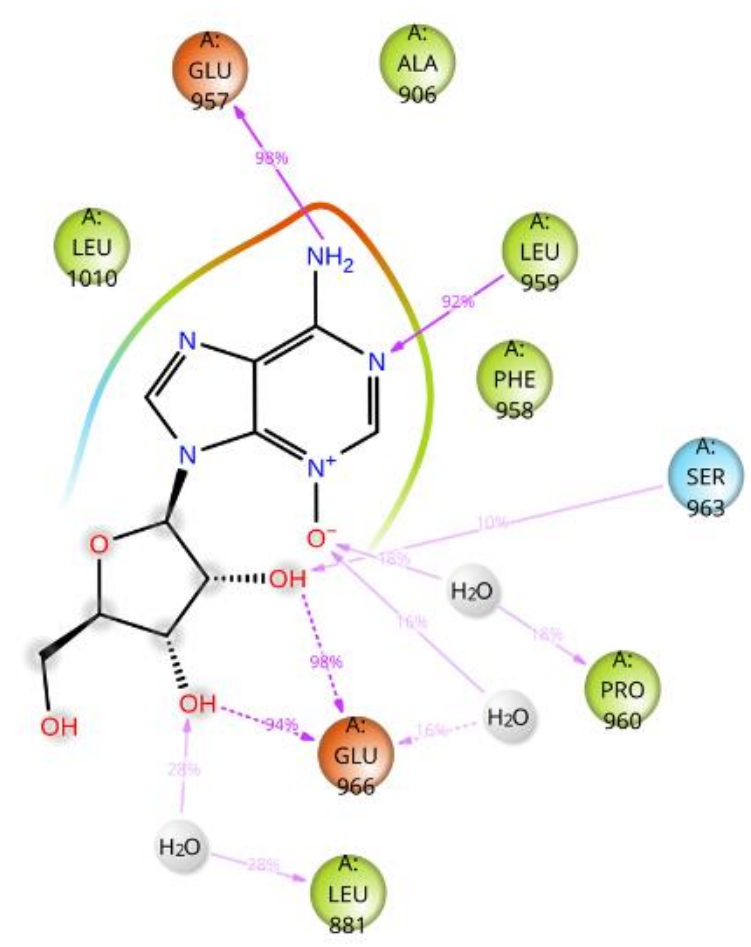

Compound **1-3** in complex
with JAK1 (3)

Figure S51: Detailed two-dimensional (2D) ligand-protein interaction diagrams of compound **1-3** with the JAK1 protein captured across independent triplicate production runs (Replica 1, Replica 2, and Replica 3) in complex with JAK1 (PDB ID: 4EI4).

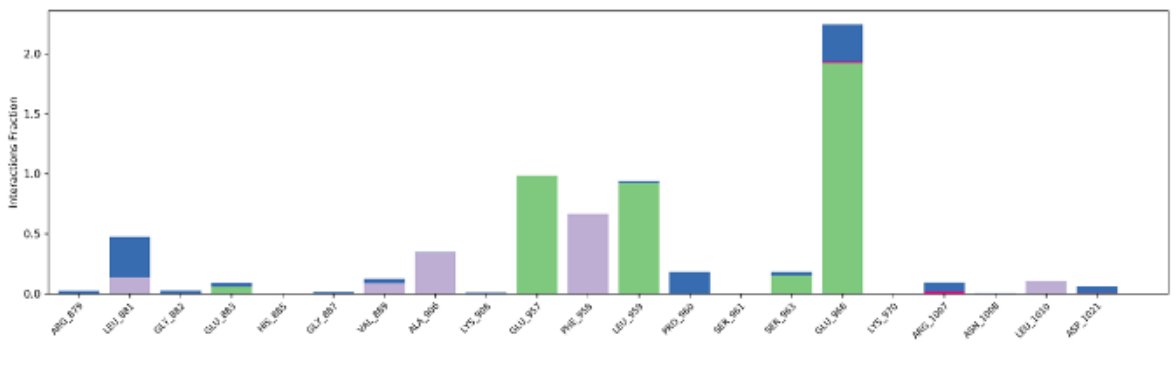

Compound **1-3** in complex
with JAK1 (1)

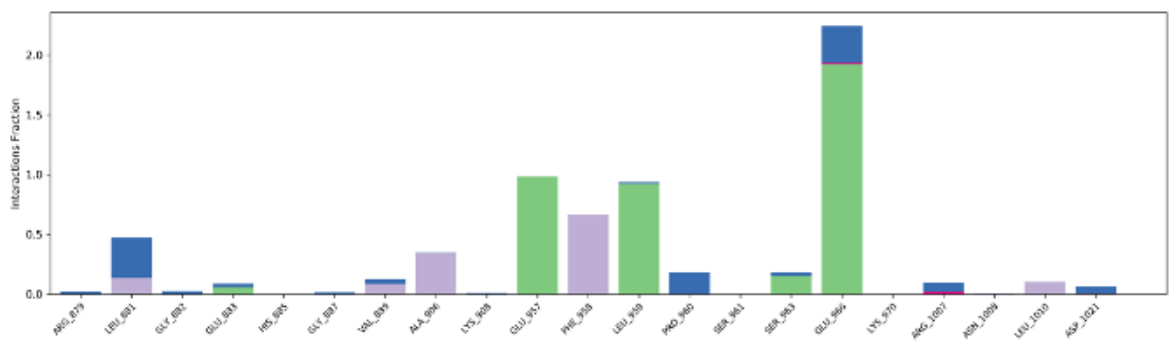

Compound **1-3** in complex
with JAK1 (2)

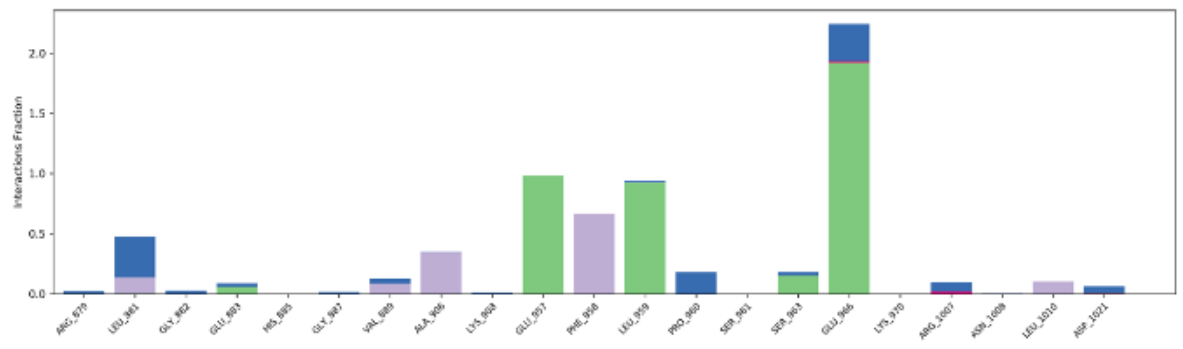

Compound **1-3** in complex
with JAK1 (3)

Figure S52: Normalized protein-ligand interaction fractions of compound **1-3** with the JAK1 protein captured across independent triplicate production runs (Replica 1, Replica 2, and Replica 3) in complex with JAK1 (PDB ID: 4EI4).

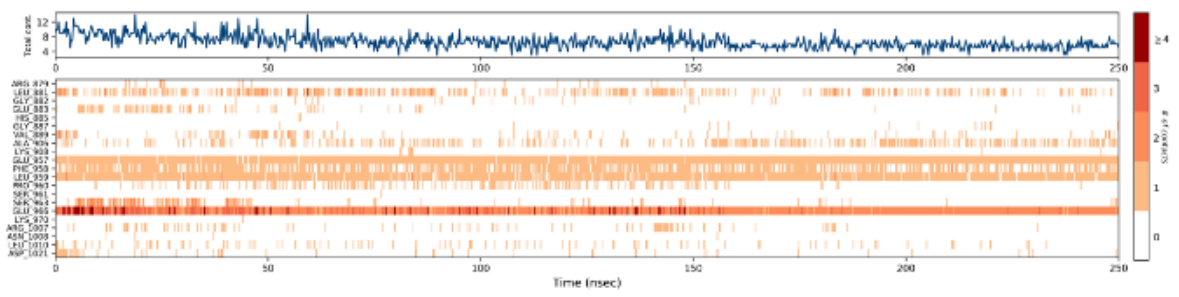

Compound **1-3** in complex
with JAK1 (1)

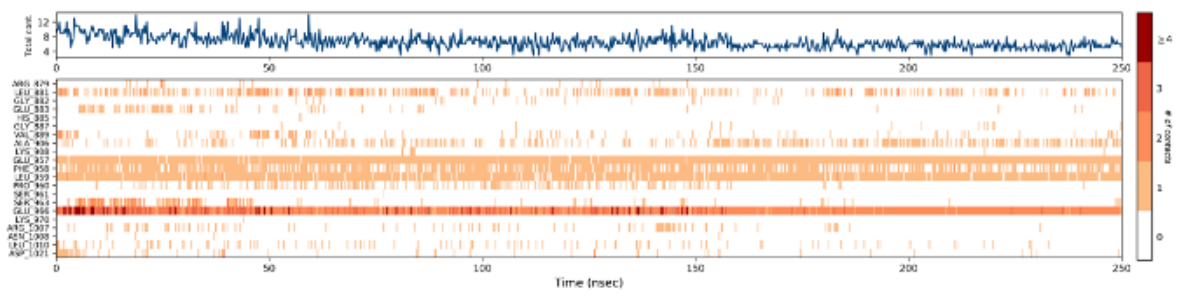

Compound **1-3** in complex
with JAK1 (2)

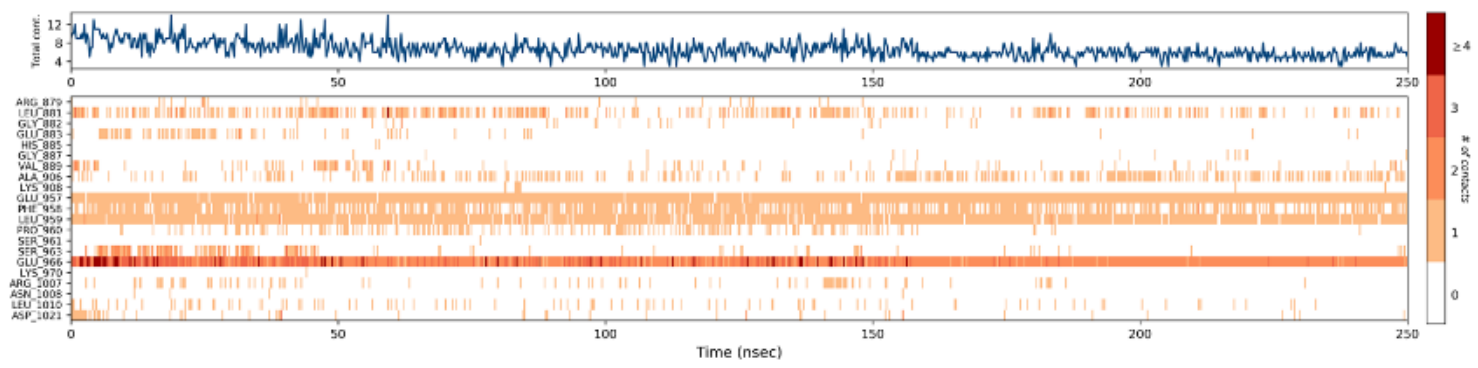

Compound **1-3** in complex
with JAK1 (3)

Figure S53: Timeline representation of compound **1-3** with the JAK1 protein captured across independent triplicate production runs (Replica 1, Replica 2, and Replica 3) in complex with JAK1 (PDB ID: 4EI4).

Compound **1-3** in complex with JAK1 (1)

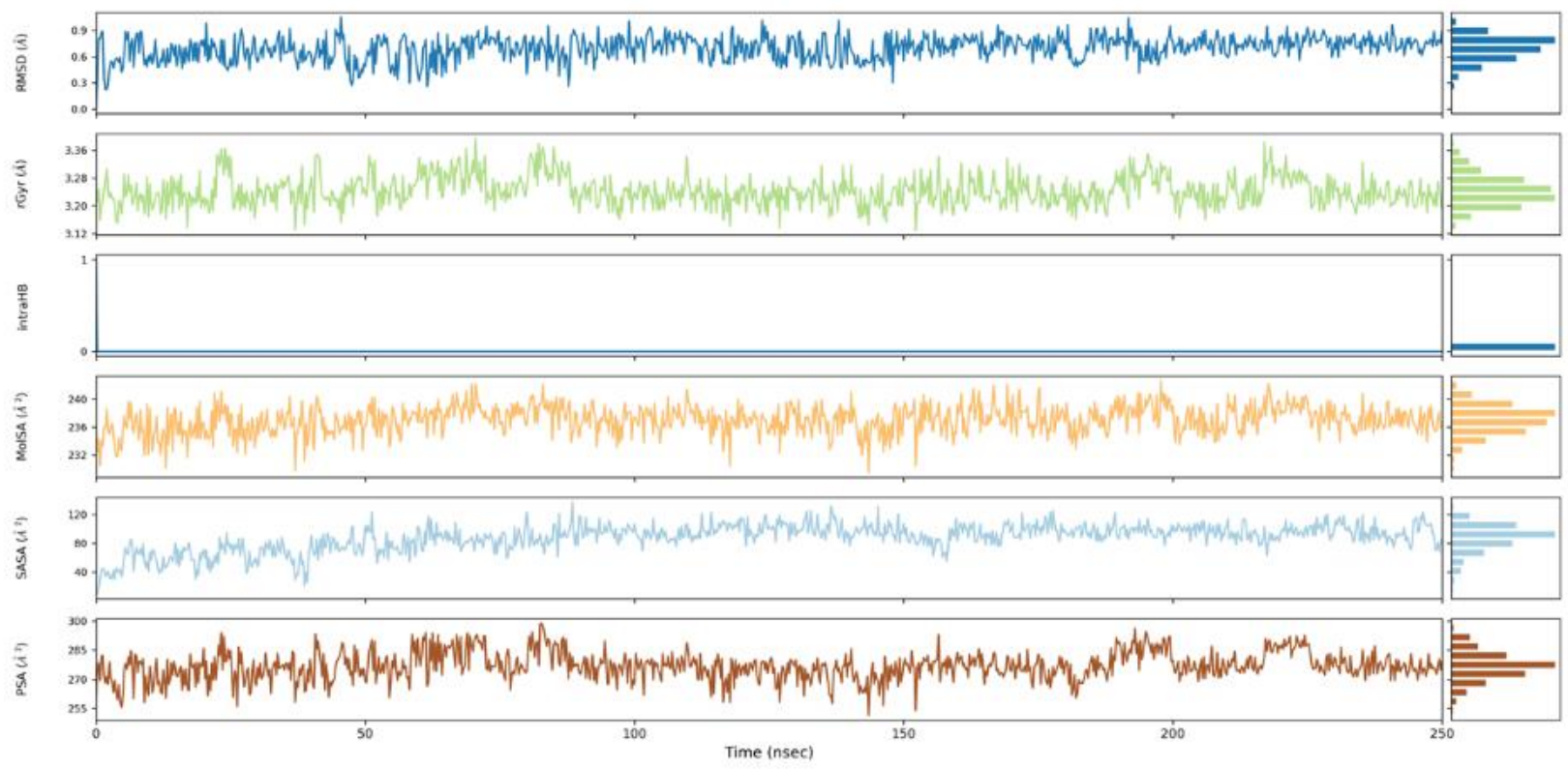

Compound **1-3** in complex with JAK1 (2)

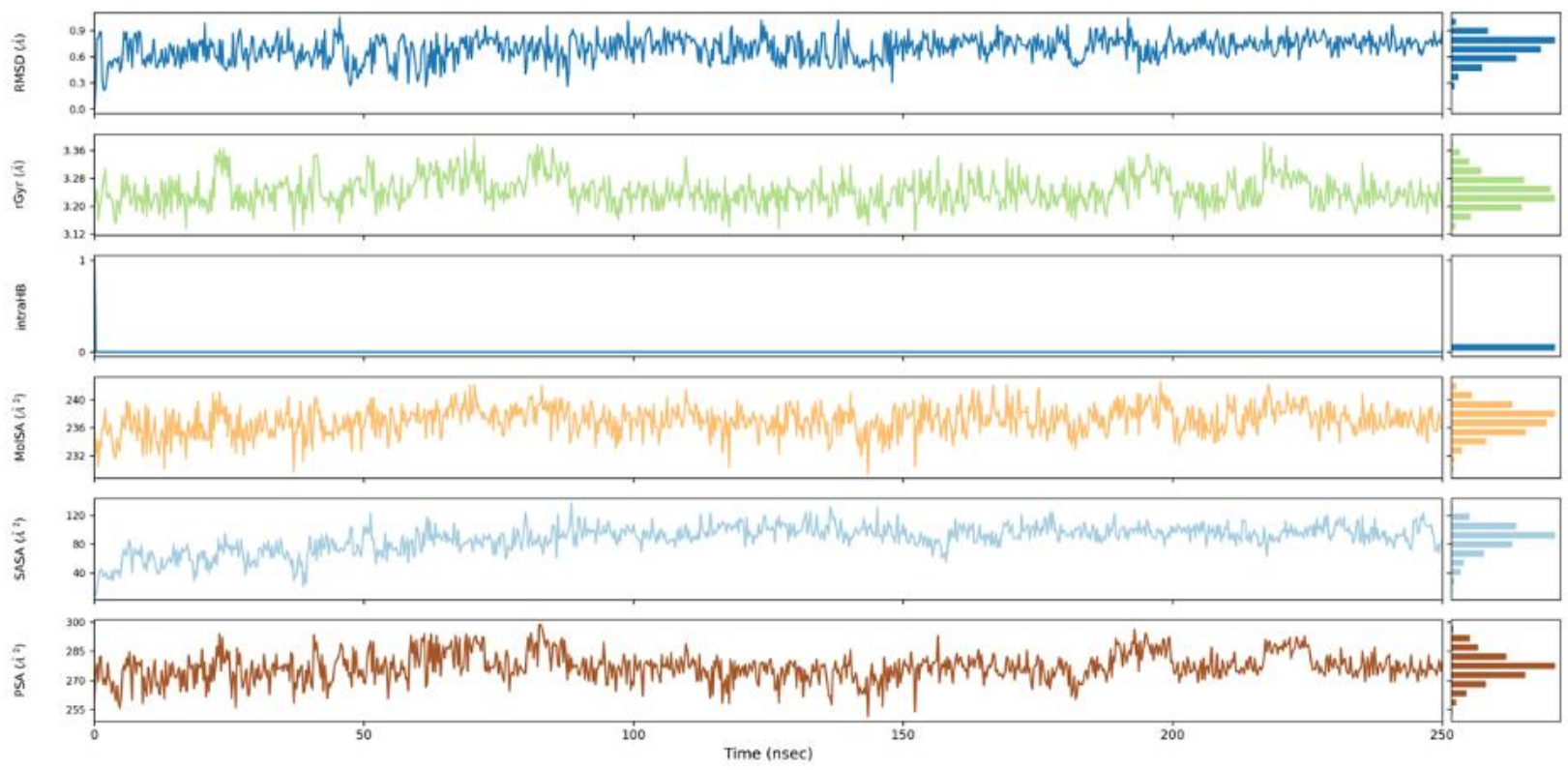

Compound **1-3** in complex with JAK1 (3)

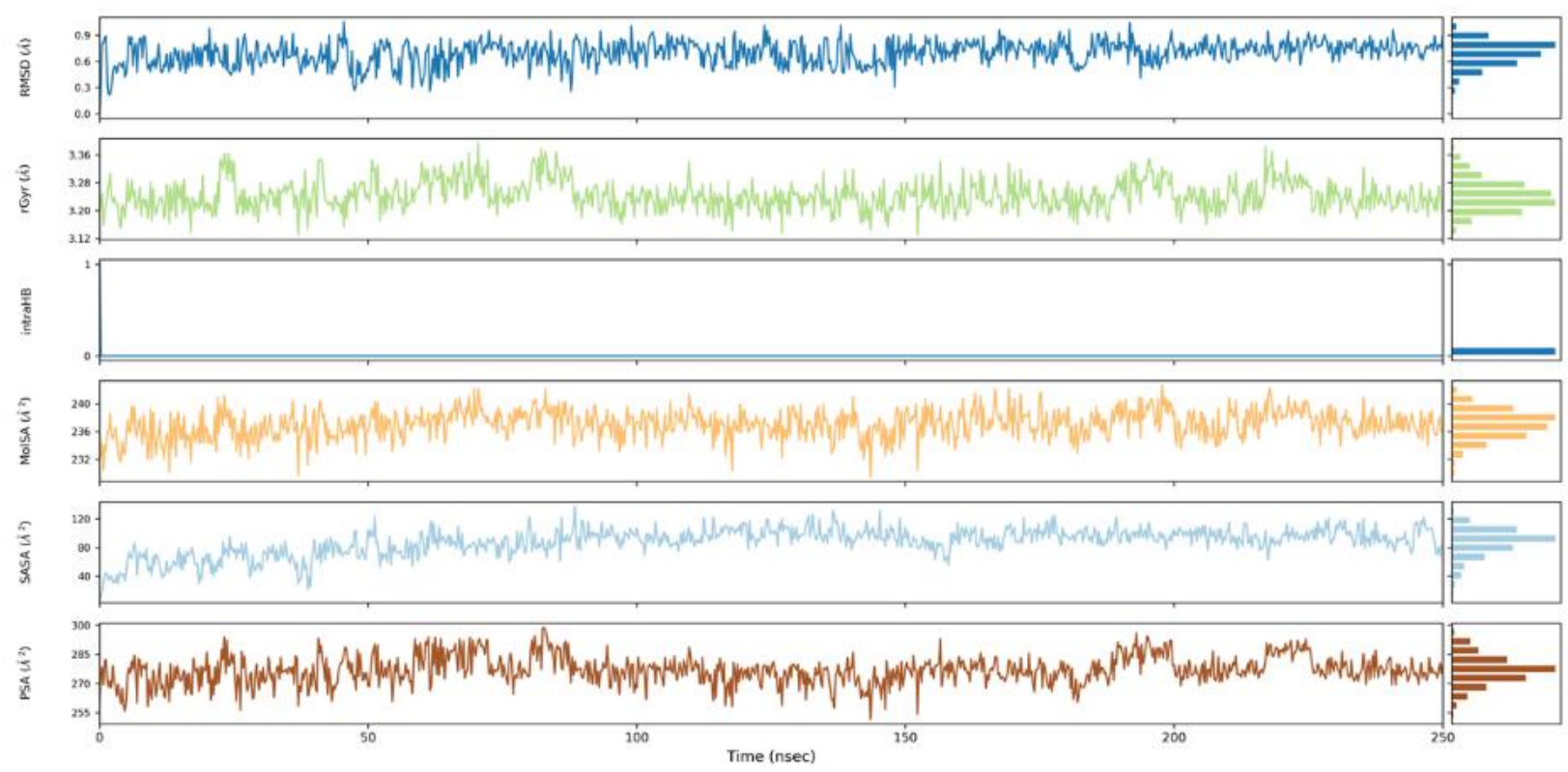

Figure S54: Time-dependent physicochemical properties of compound **1-3** with the JAK1 protein captured across independent triplicate production runs (Replica 1, Replica 2, and Replica 3) in complex with JAK1 (PDB ID: 4EI4).

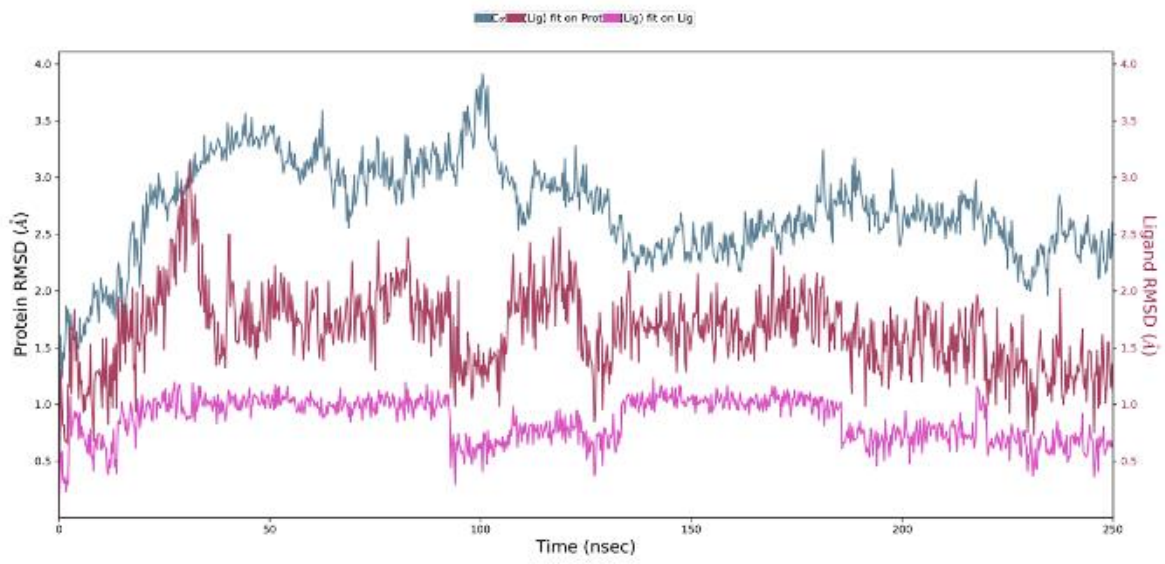

Compound **2-8** in complex
with JAK2 (1)

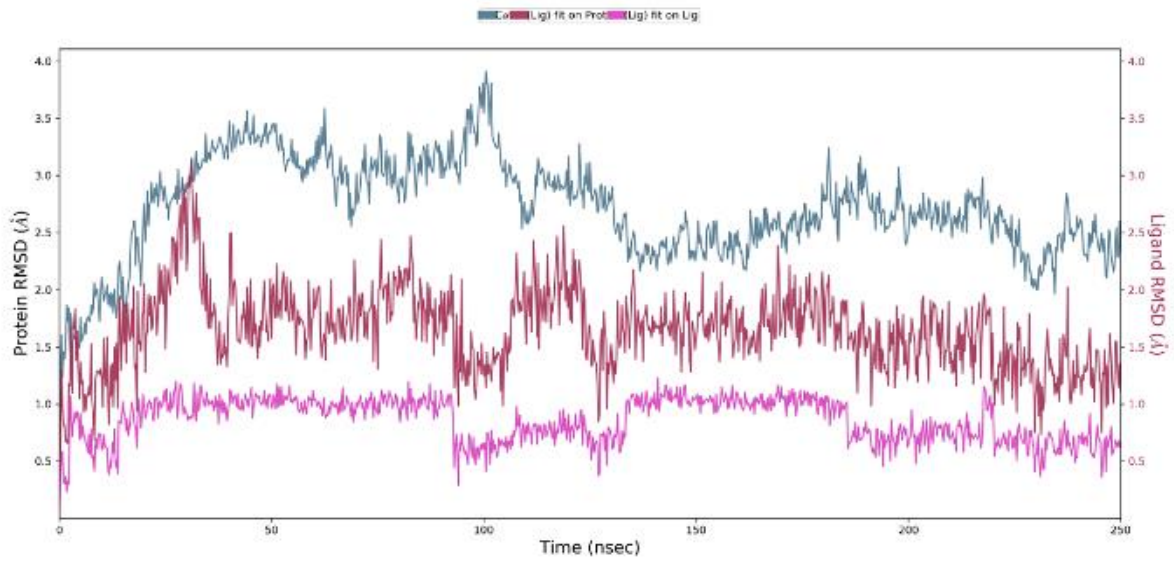

Compound **2-8** in complex
with JAK2 (2)

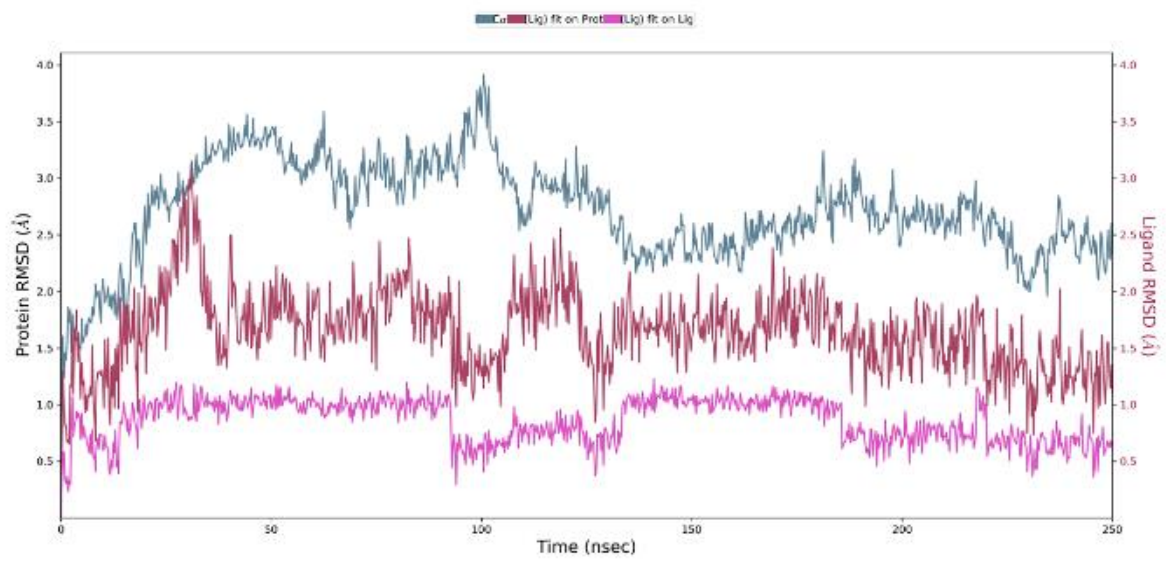

Compound **2-8** in complex
with JAK2 (3)

Figure S55: The RMSD plots of compound **2-8** with the JAK2 protein as a function of simulation time (0 to 250 ns) captured across independent triplicate production runs (Replica 1, Replica 2, and Replica 3) in complex with JAK2 (PDB ID: 6VGL).

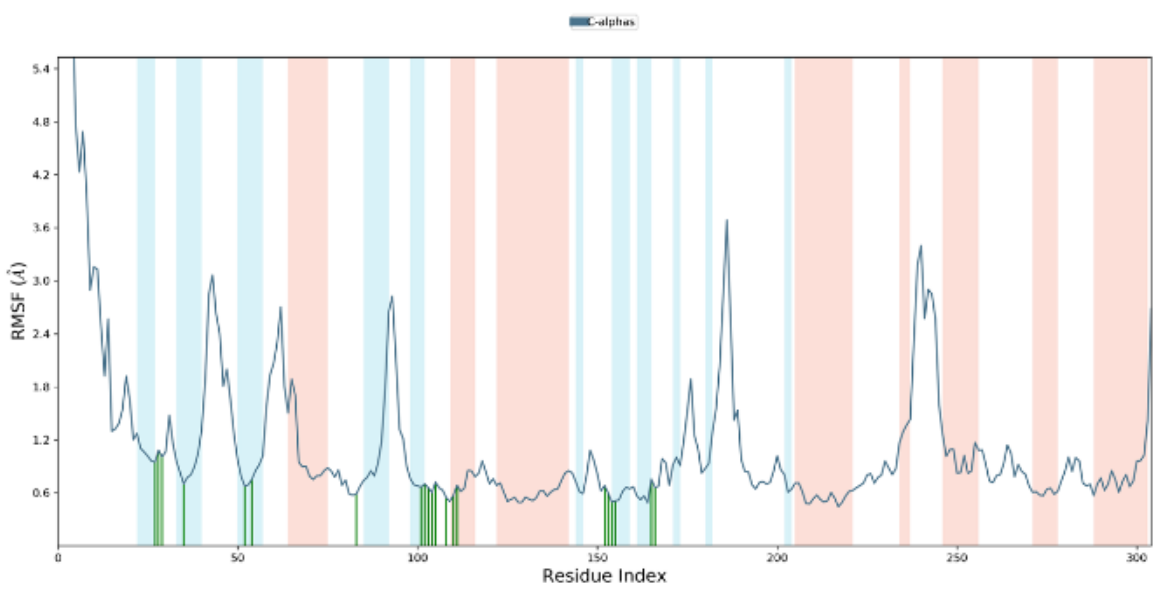

Compound **2-8** in complex
with JAK2 (1)

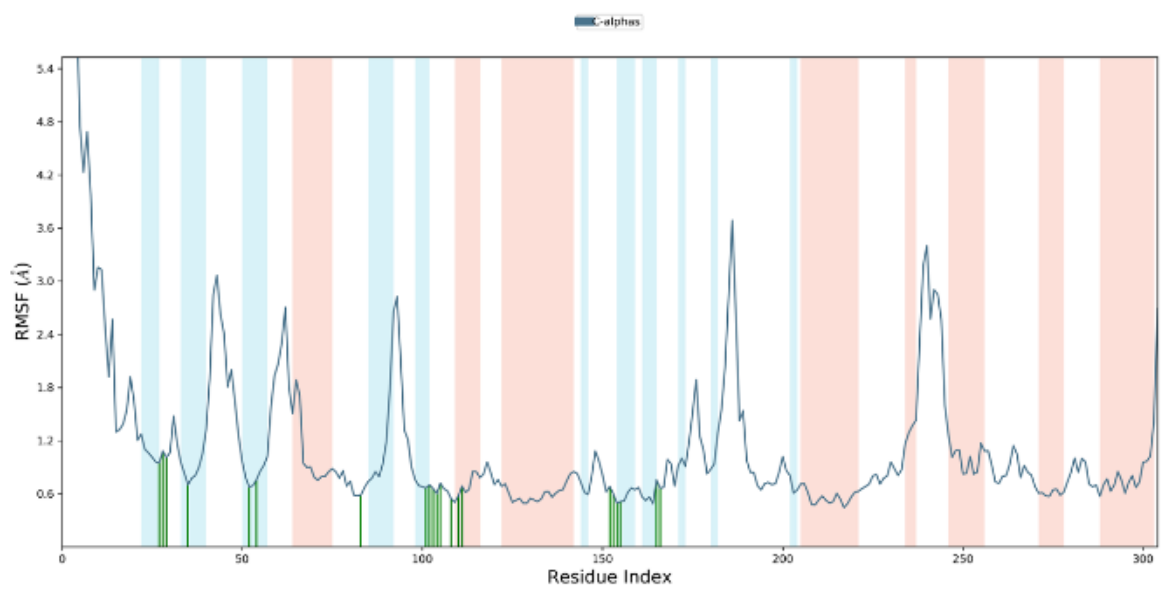

Compound **2-8** in complex
with JAK2 (2)

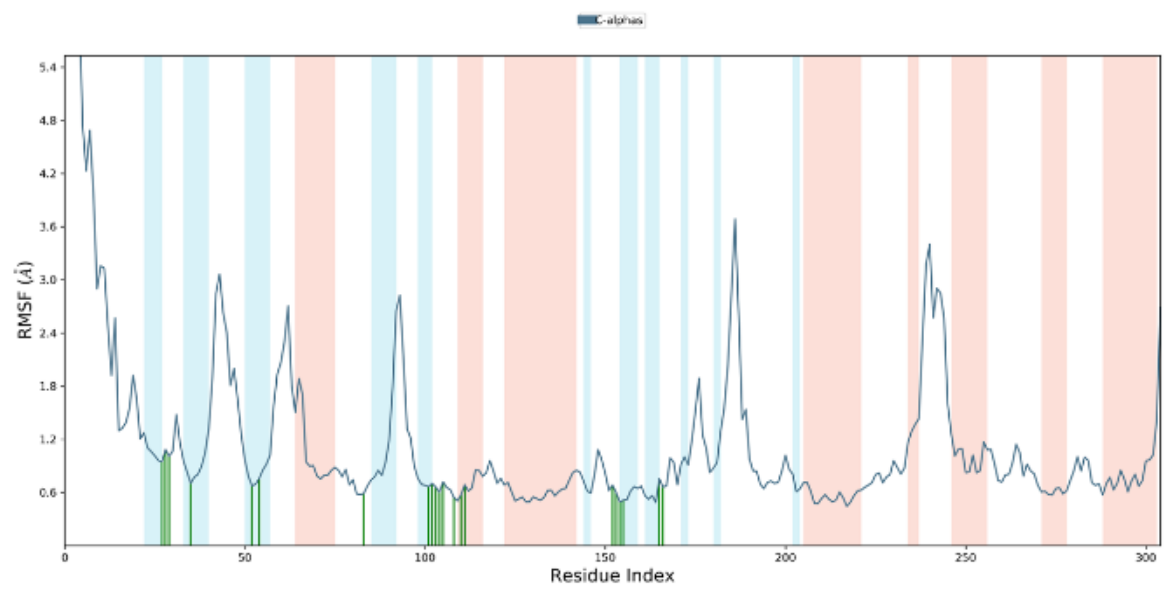

Compound **2-8** in complex
with JAK2 (3)

Figure S56: The RMSF profiles compound **2-8** with the JAK2 protein captured across independent triplicate production runs (Replica 1, Replica 2, and Replica 3) in complex with JAK2 (PDB ID: 6VGL).

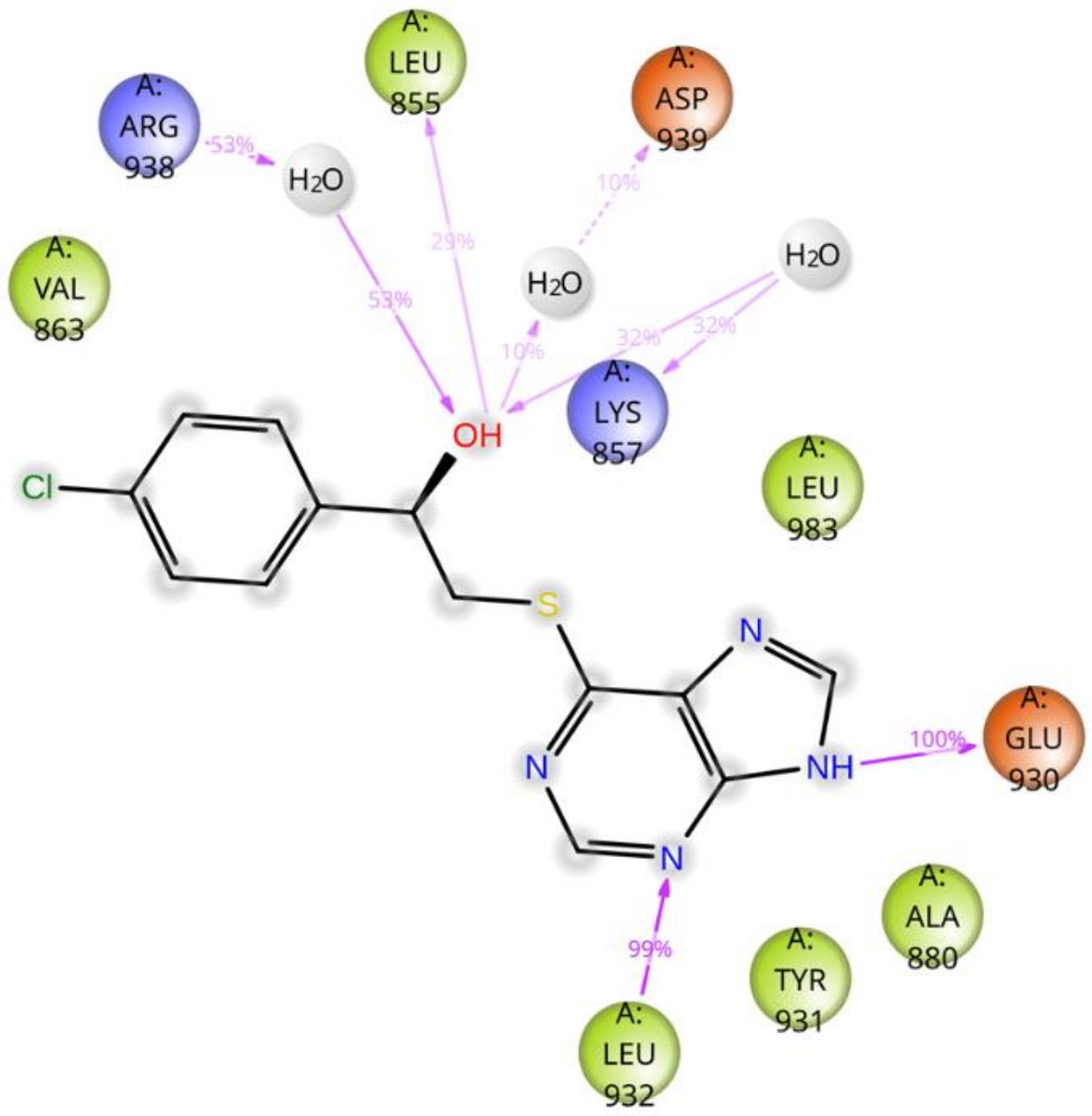

Compound **2-8** in complex with JAK2 (1)

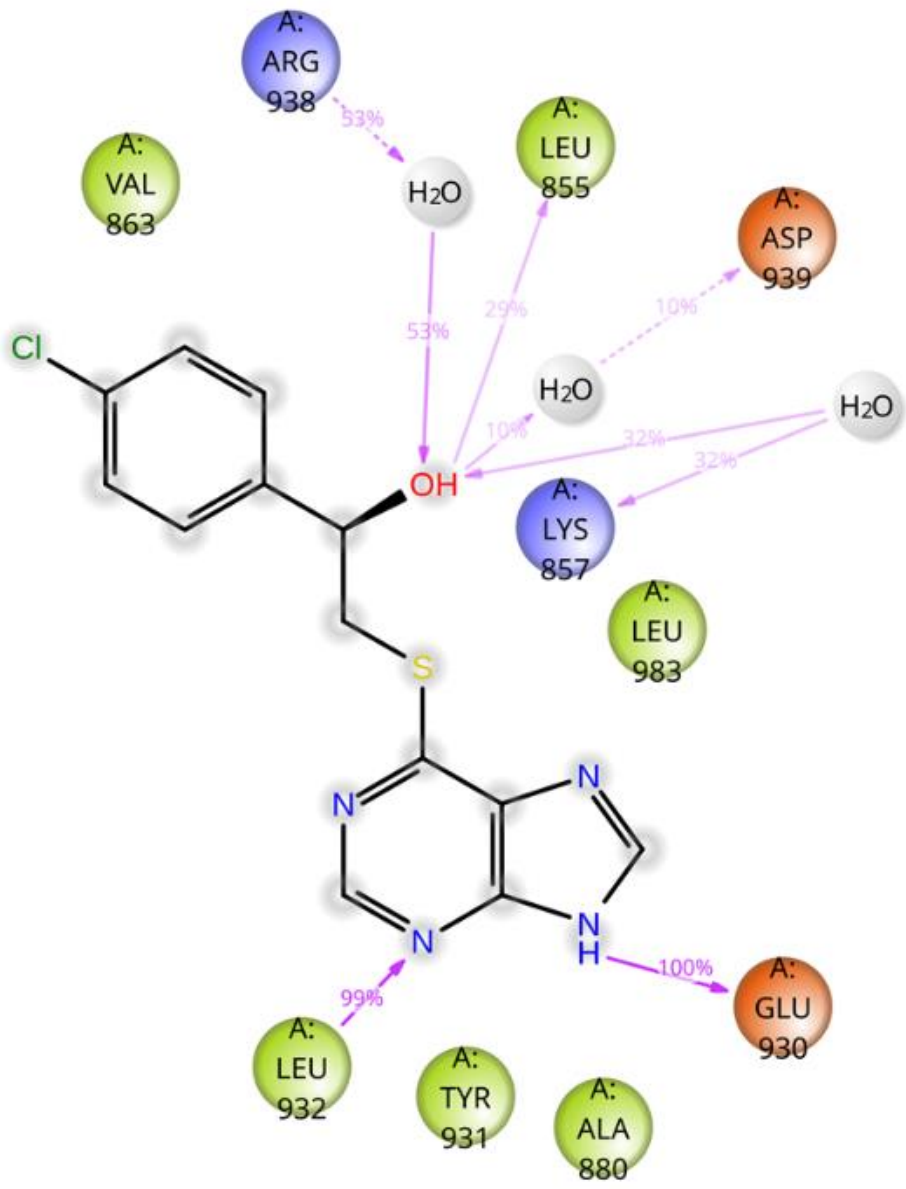

Compound **2-8** in complex with JAK2 (2)

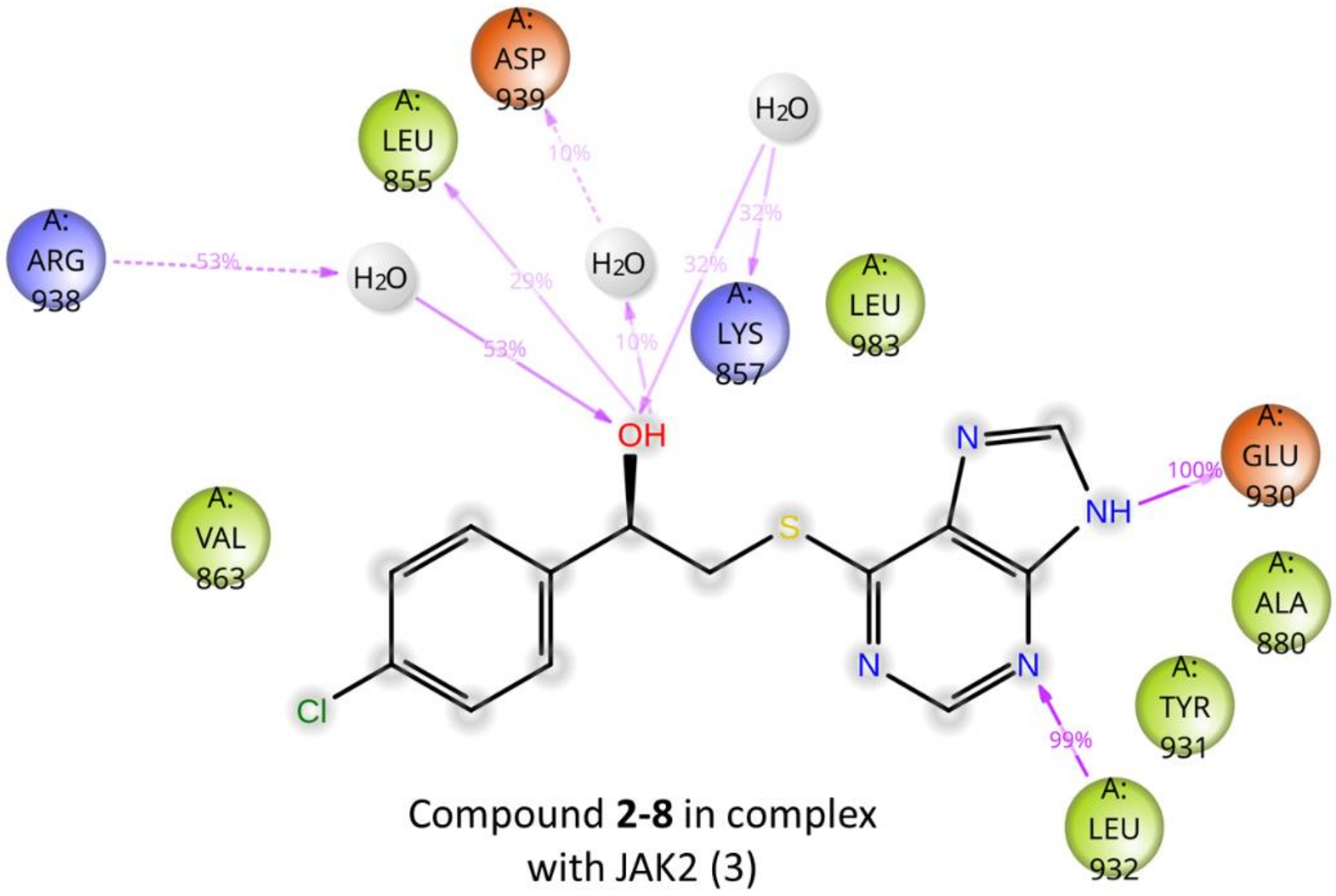

Compound **2-8** in complex with JAK2 (3)

Figure S57: Detailed two-dimensional (2D) ligand-protein interaction diagrams of compound **2-8** with the JAK2 protein captured across independent triplicate production runs (Replica 1, Replica 2, and Replica 3) in complex with JAK2 (PDB ID: 6VGL).

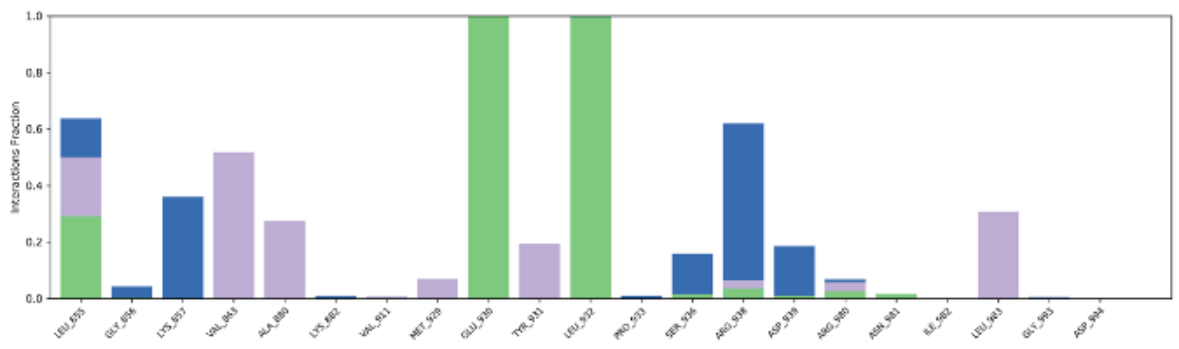

Compound **2-8** in complex with JAK2 (1)

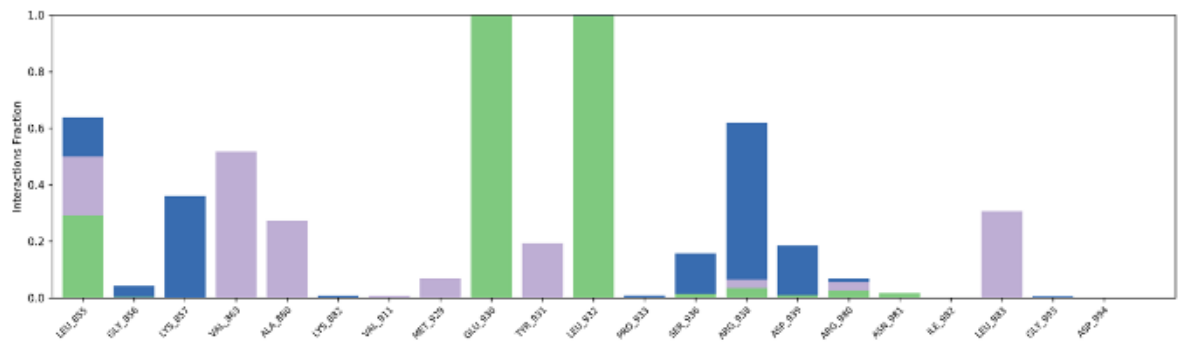

Compound **2-8** in complex with JAK2 (2)

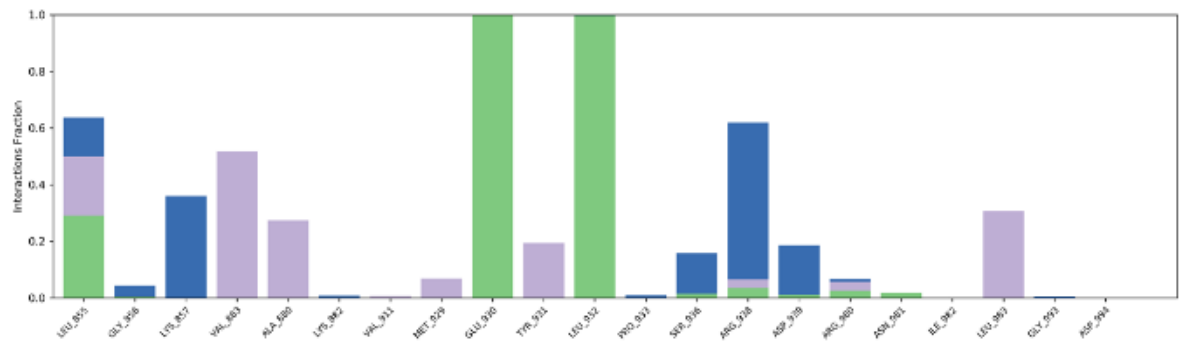

Compound **2-8** in complex with JAK2 (3)

Figure S58: Normalized protein-ligand interaction fractions of compound **2-8** with the JAK2 protein captured across independent triplicate production runs (Replica 1, Replica 2, and Replica 3) in complex with JAK2 (PDB ID: 6VGL).

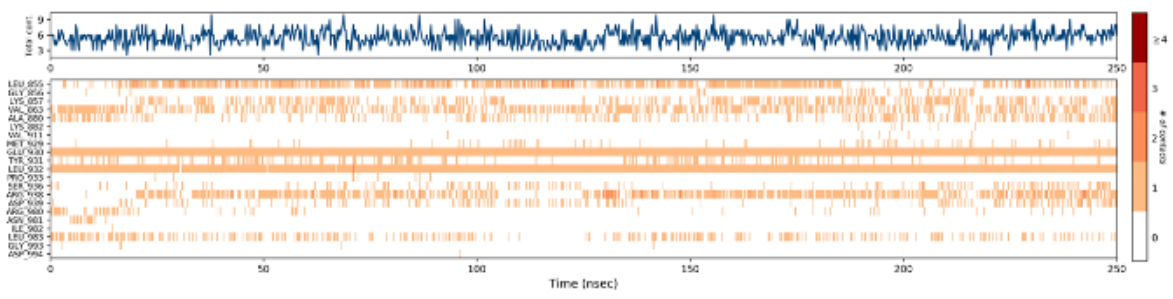

Compound **2-8** in complex with JAK2 (1)

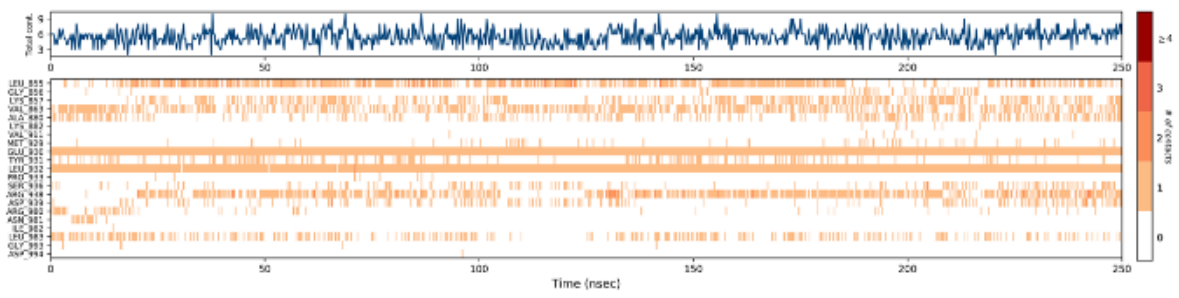

Compound **2-8** in complex with JAK2 (2)

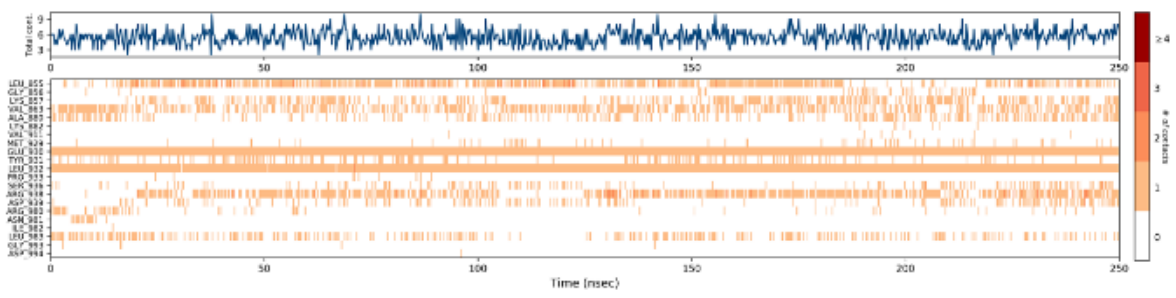

Compound **2-8** in complex with JAK2 (3)

Figure S59: Timeline representation of compound **2-8** with the JAK2 protein captured across independent triplicate production runs (Replica 1, Replica 2, and Replica 3) in complex with JAK2 (PDB ID: 6VGL).

Compound **2-8** in complex with JAK2 (1)

Compound **2-8** in complex with JAK2 (2)

Compound **2-8** in complex with JAK2 (3)

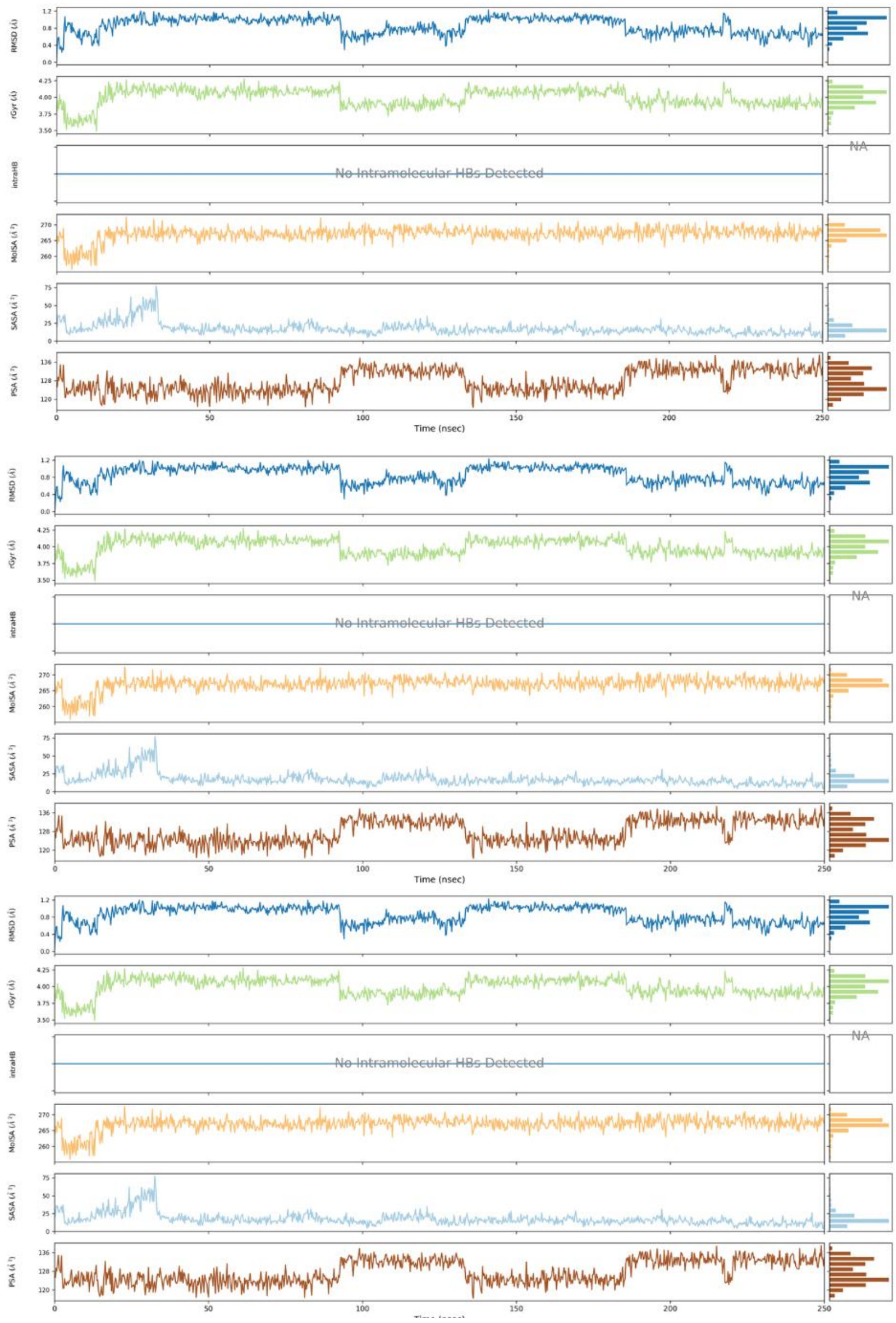

Figure S60. Time-dependent physicochemical properties of compound **2-8** with the JAK2 protein captured across independent triplicate production runs (Replica 1, Replica 2, and Replica 3) in complex with JAK2 (PDB ID: 6VGL).

11. NMR spectra and certificate of analysis for 42 compounds tested *in vitro*

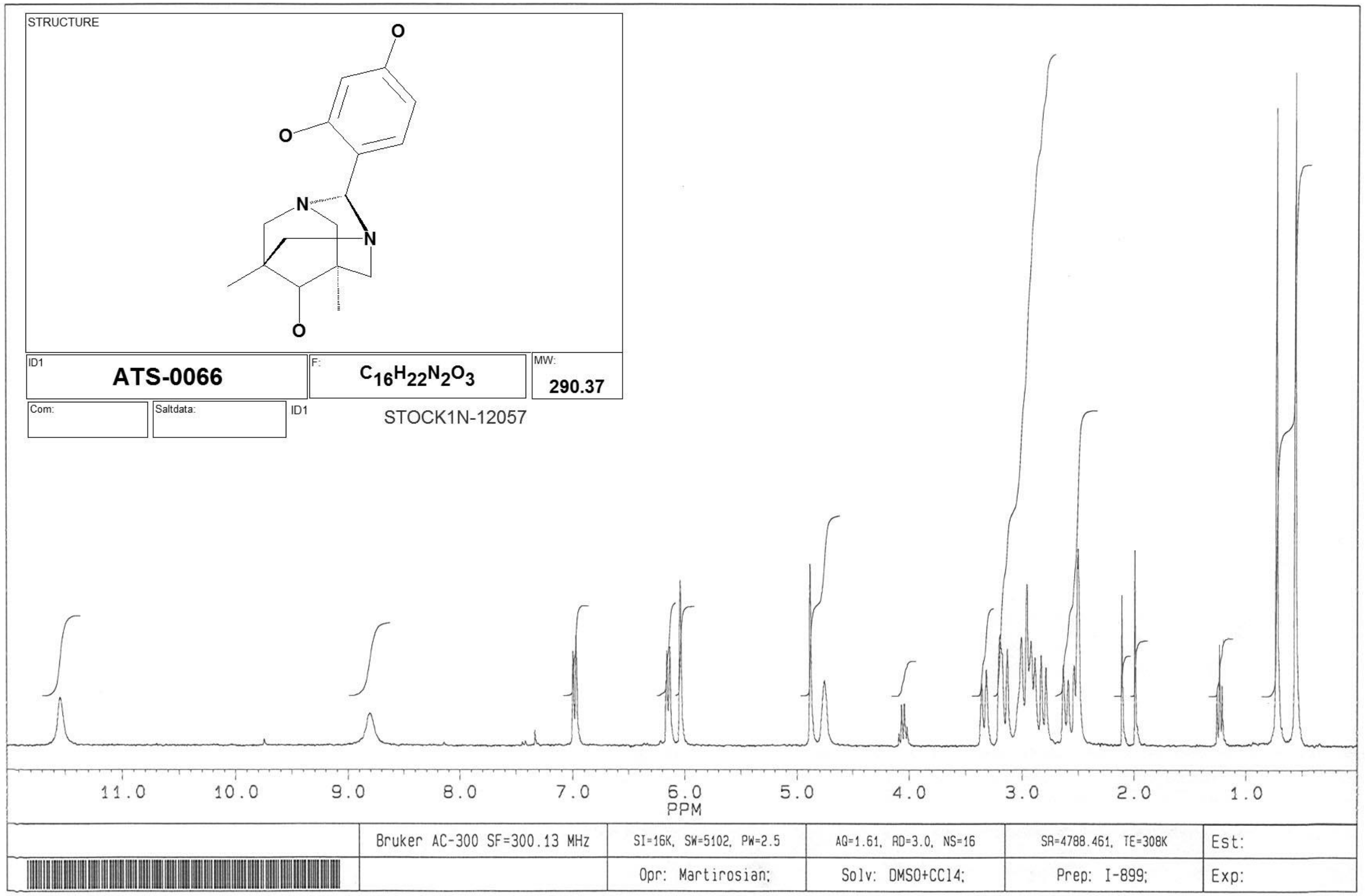

Figure S61. 1H-NMR spectrum of STOCK1N-12057

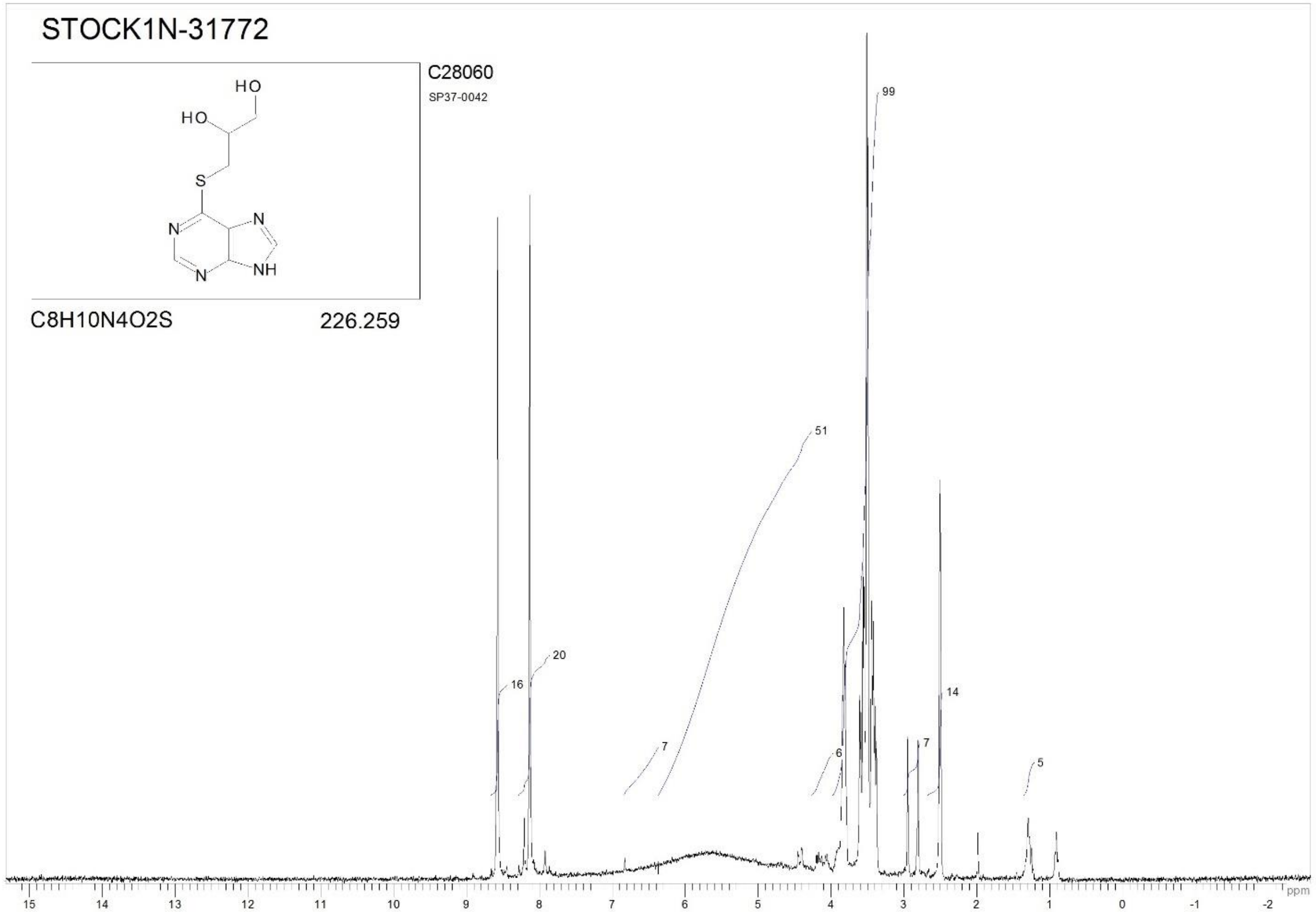

Figure S62. 1H-NMR spectrum of STOCK1N-31772

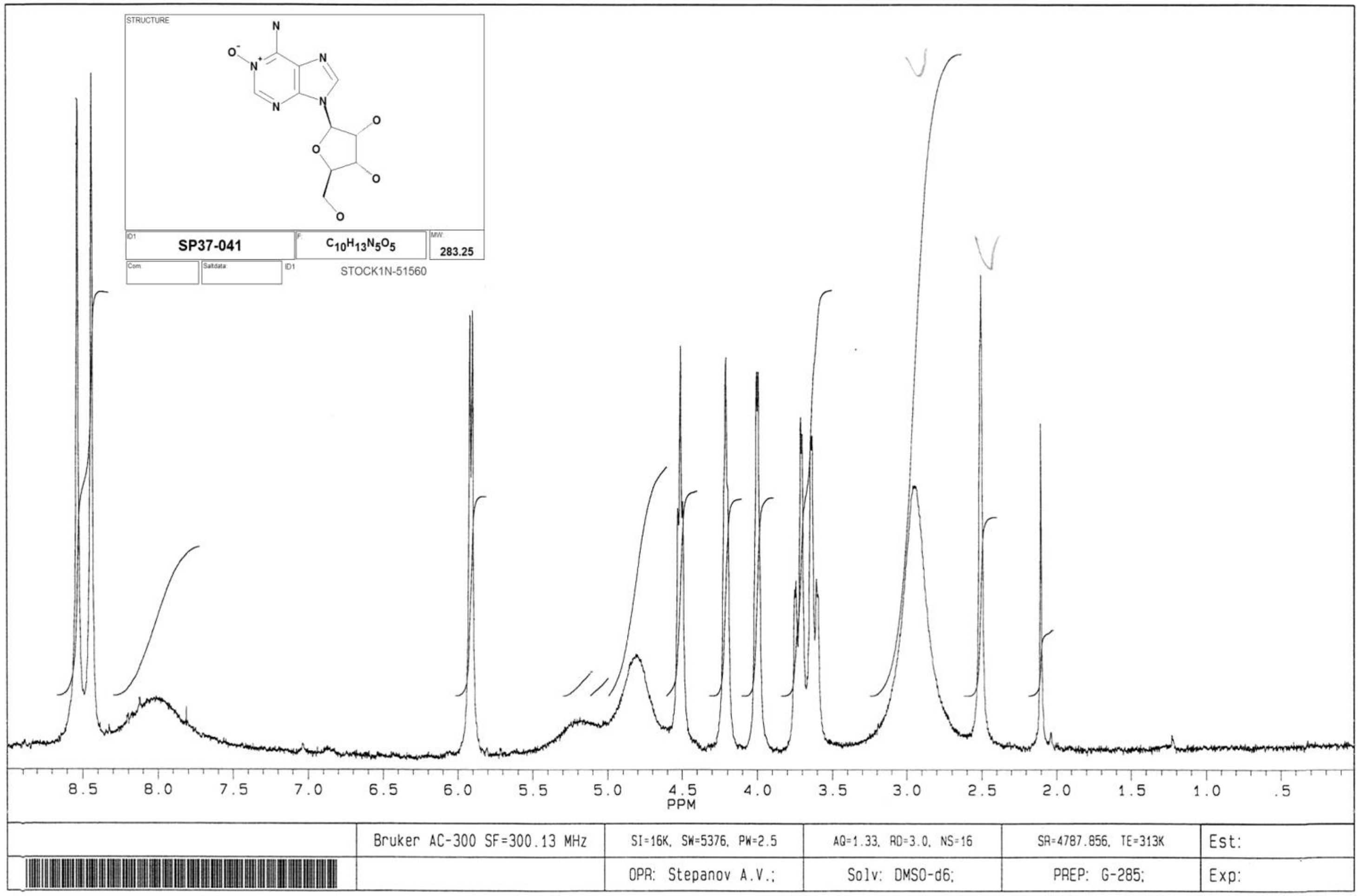

Figure S63. 1H-NMR spectrum of STOCK1N-51560

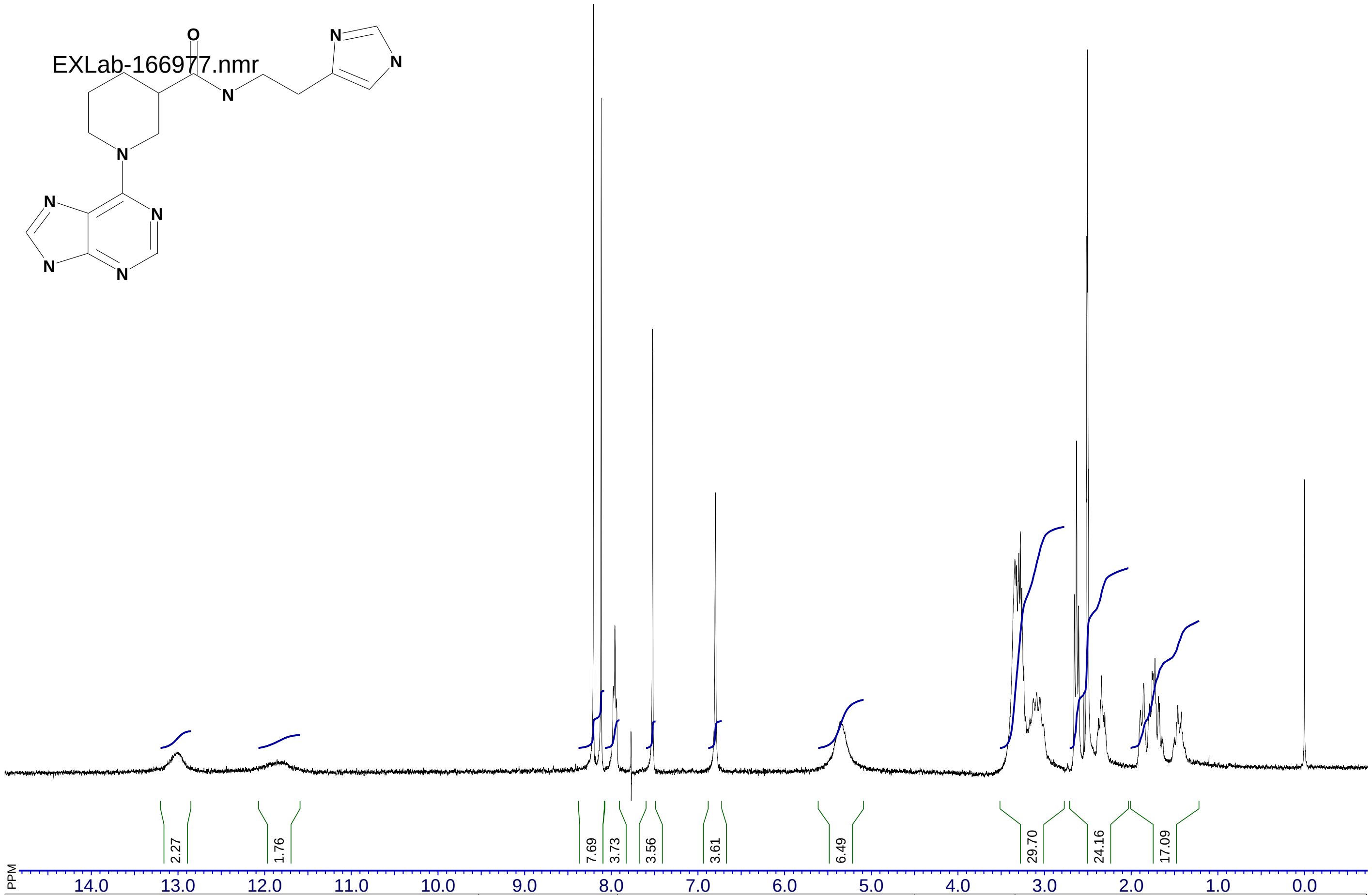

**File name: EXLab-166977.nmr**	Owner:	SF: 299.9450 MHz	NS: -1	SI: 16384, TD: 32768
Date:	Solvent:	SW: 5099	TE: 0	SPE in DMSO-D6

Figure S64. 1H-NMR spectrum of STOCK1N-69616

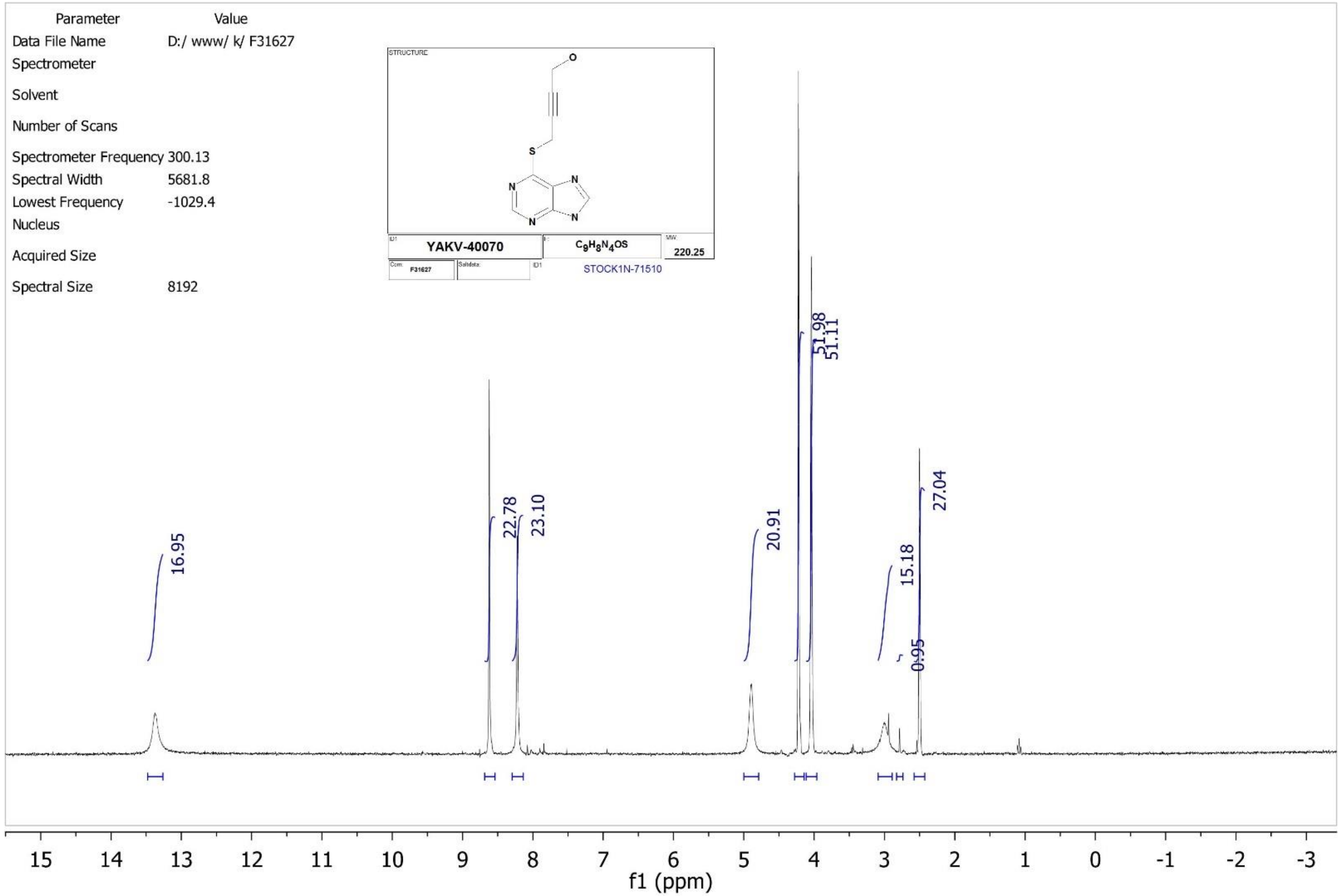

Figure S65. 1H-NMR spectrum of STOCK1N-71510

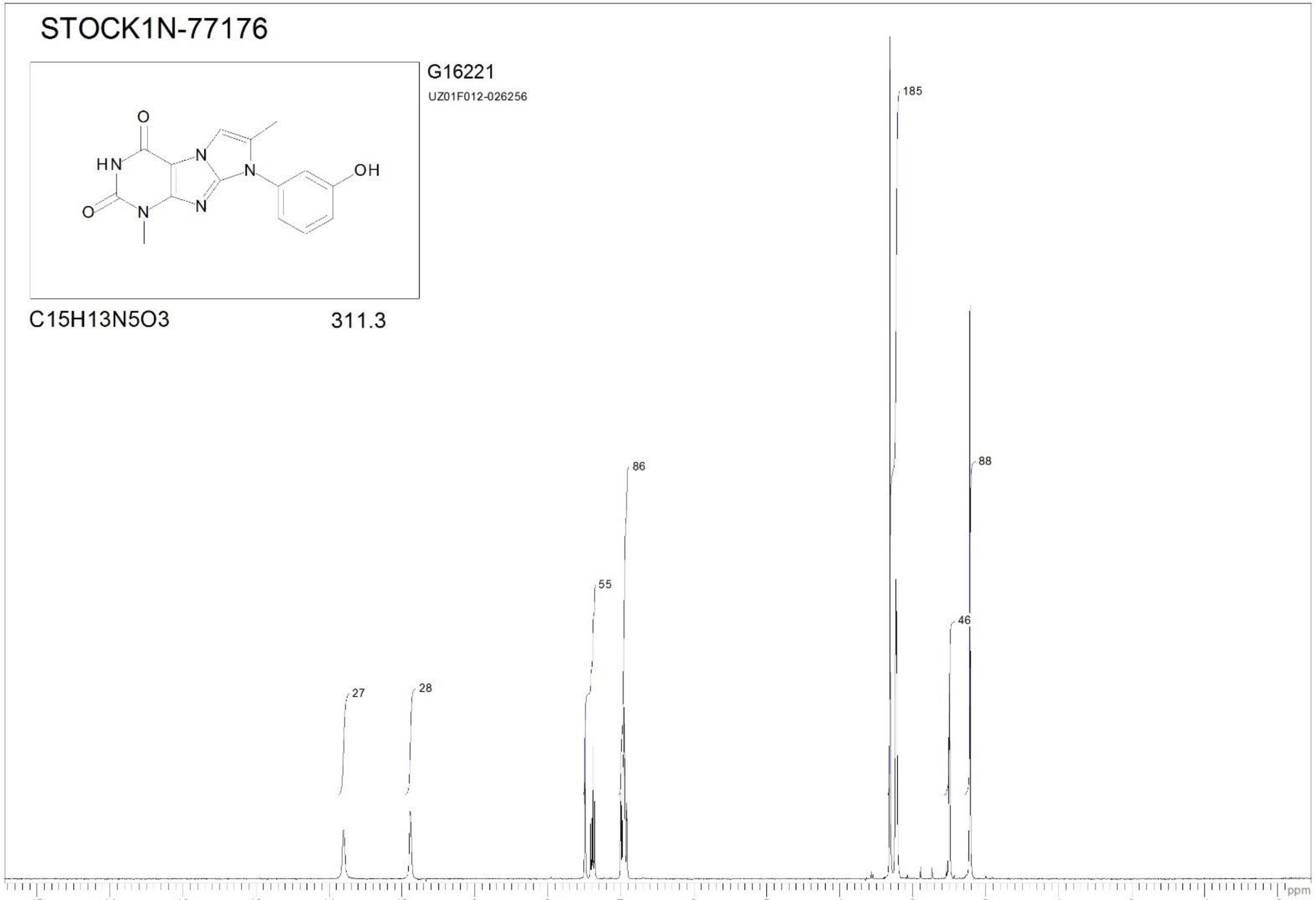

Figure S66. 1H-NMR spectrum of STOCK1N-77176

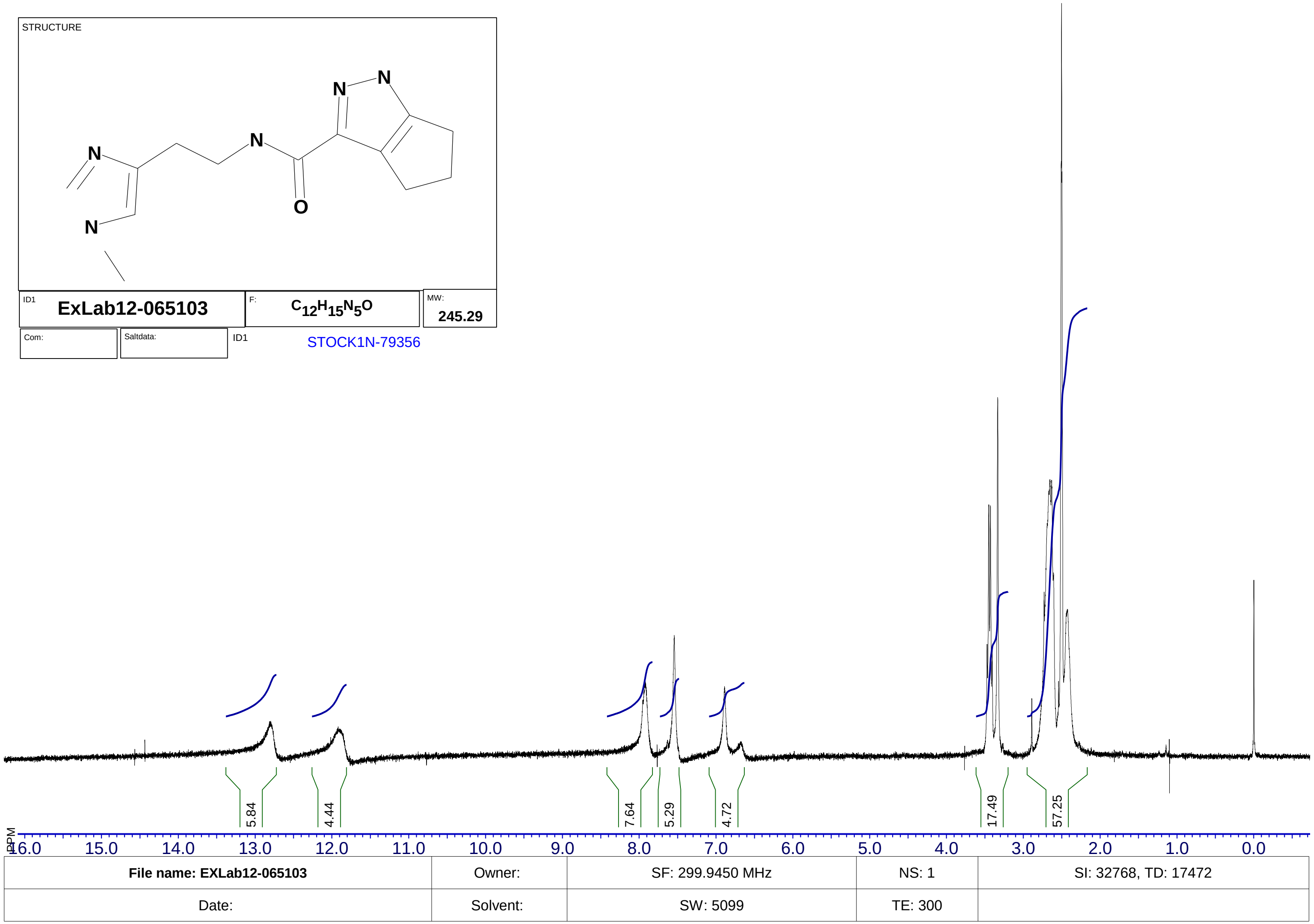

Figure S67. 1H-NMR spectrum of STOCK1N-79356

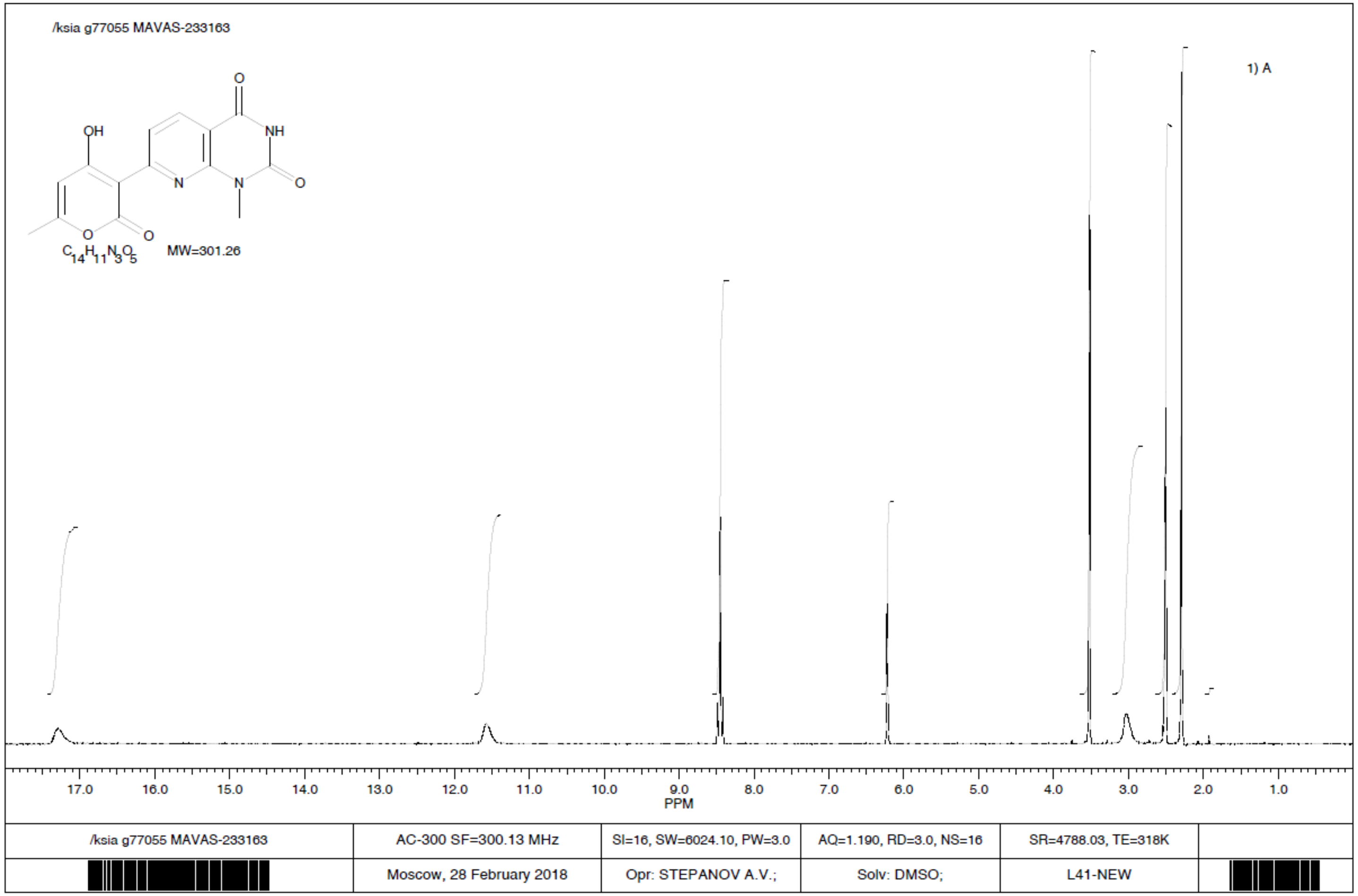

Figure S68. 1H-NMR spectrum of STOCK1N-97980

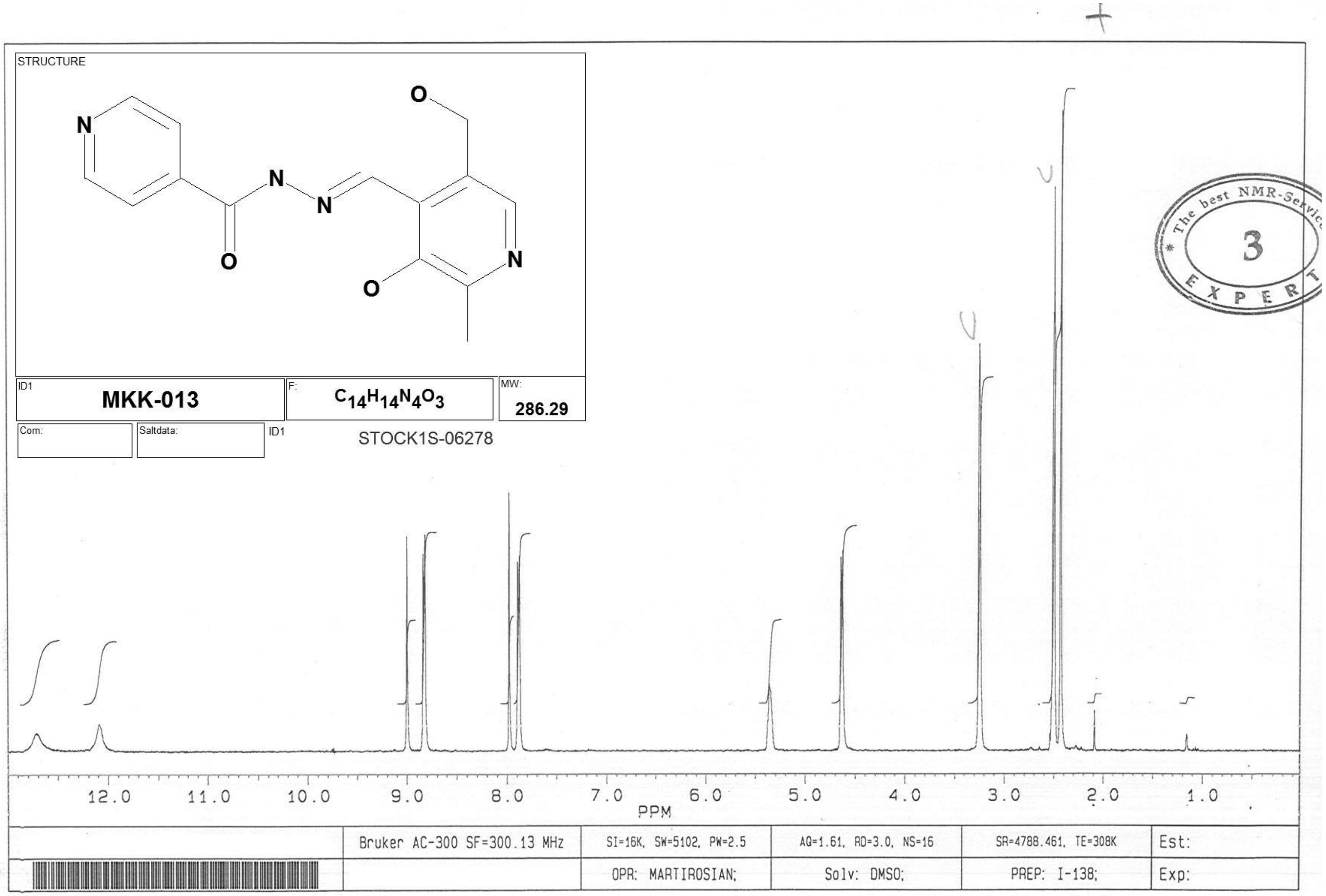

Figure S69. 1H-NMR spectrum of STOCK1S-06278

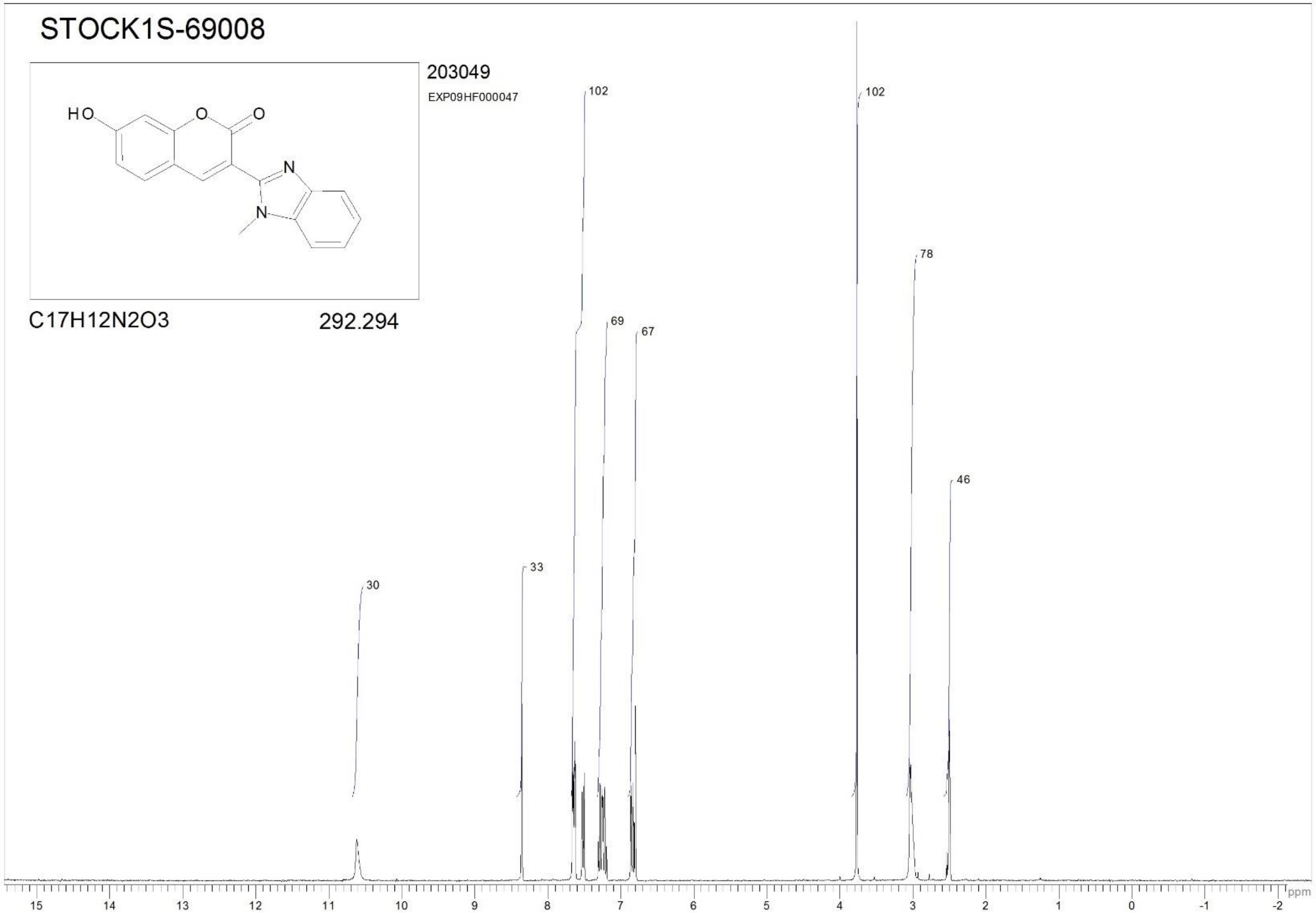

Figure S70. 1H-NMR spectrum of STOCK1S-69008

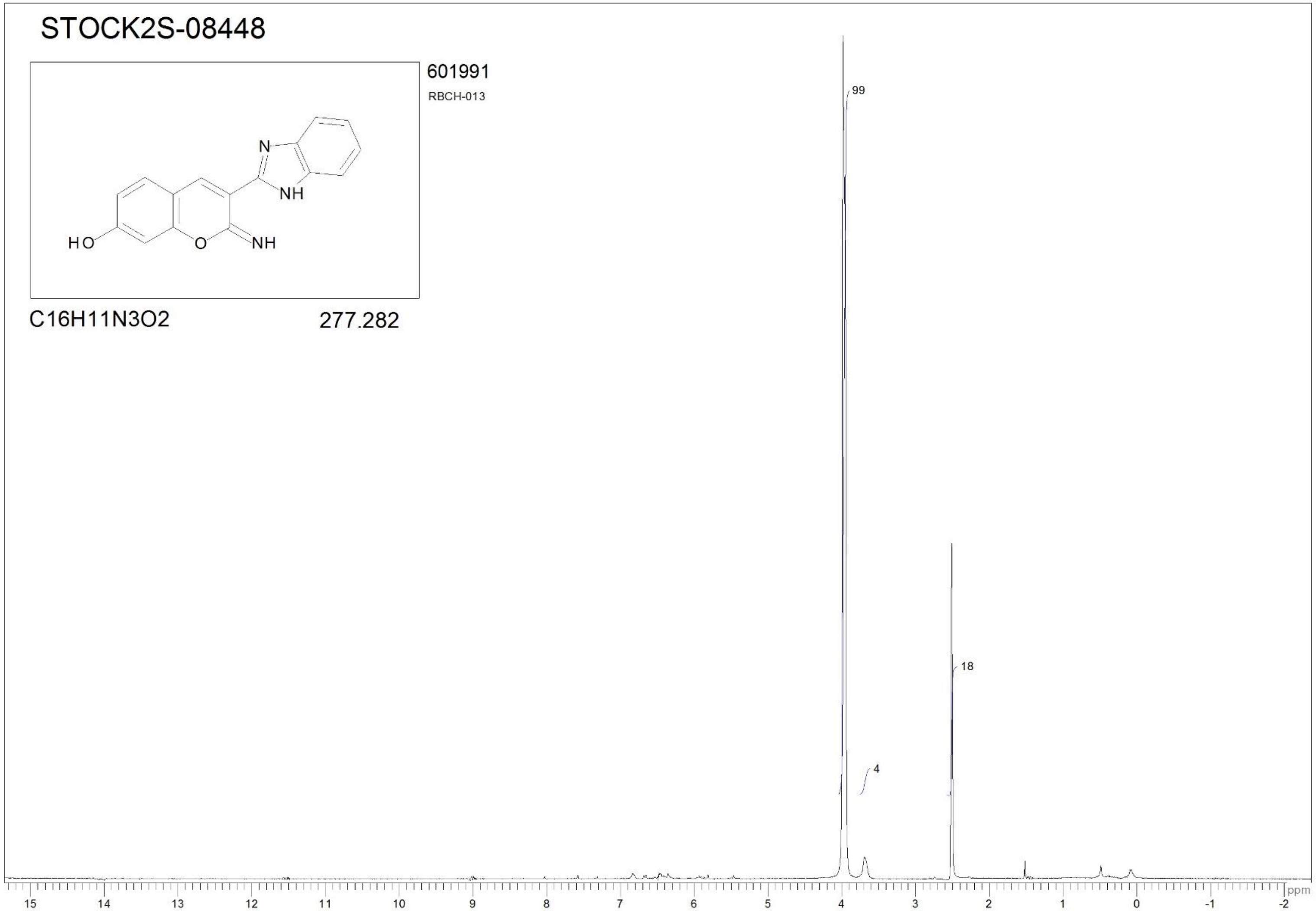

Figure S71. 1H-NMR spectrum of STOCK2S-08448

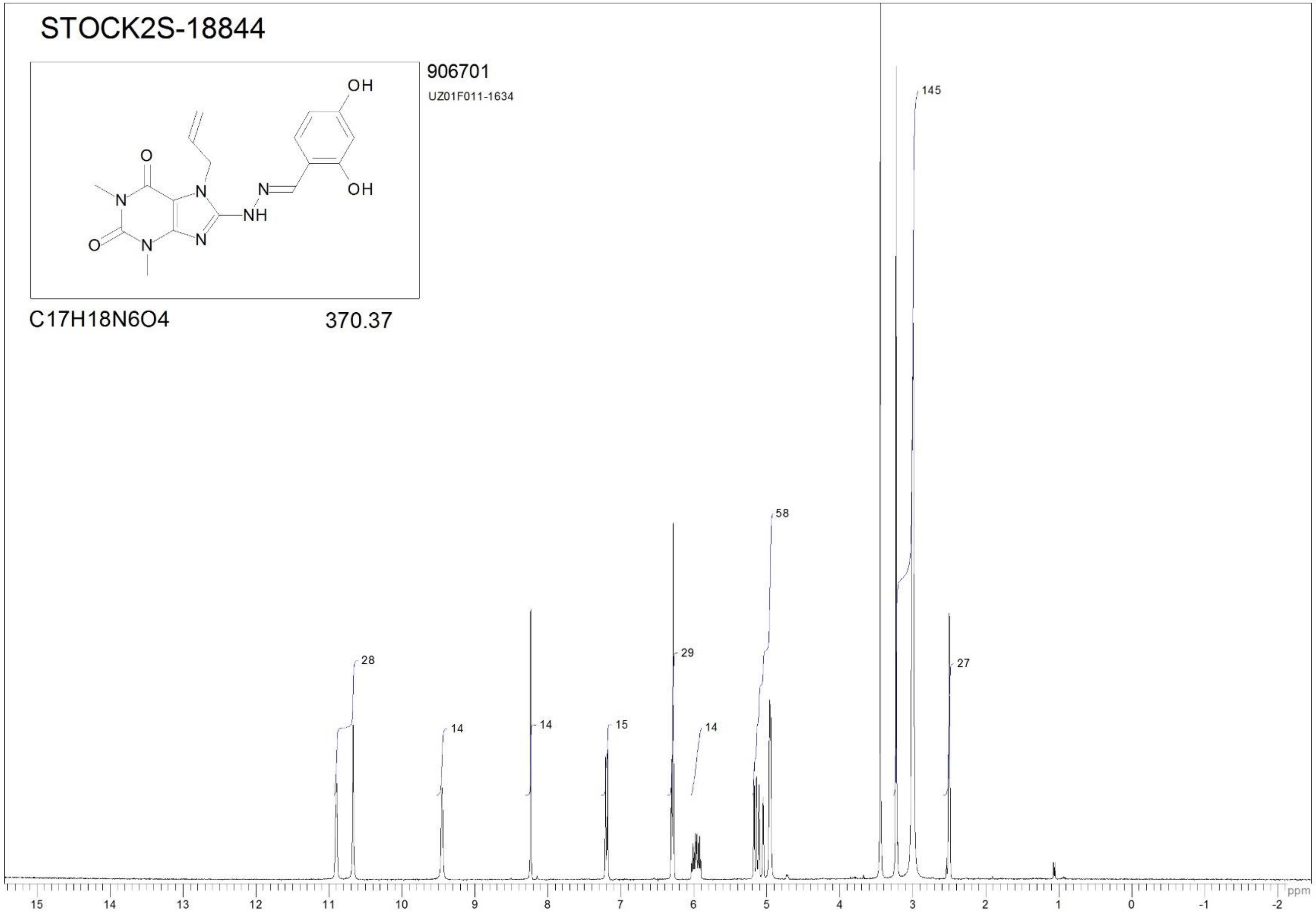

Figure S72. 1H-NMR spectrum of STOCK2S-18844

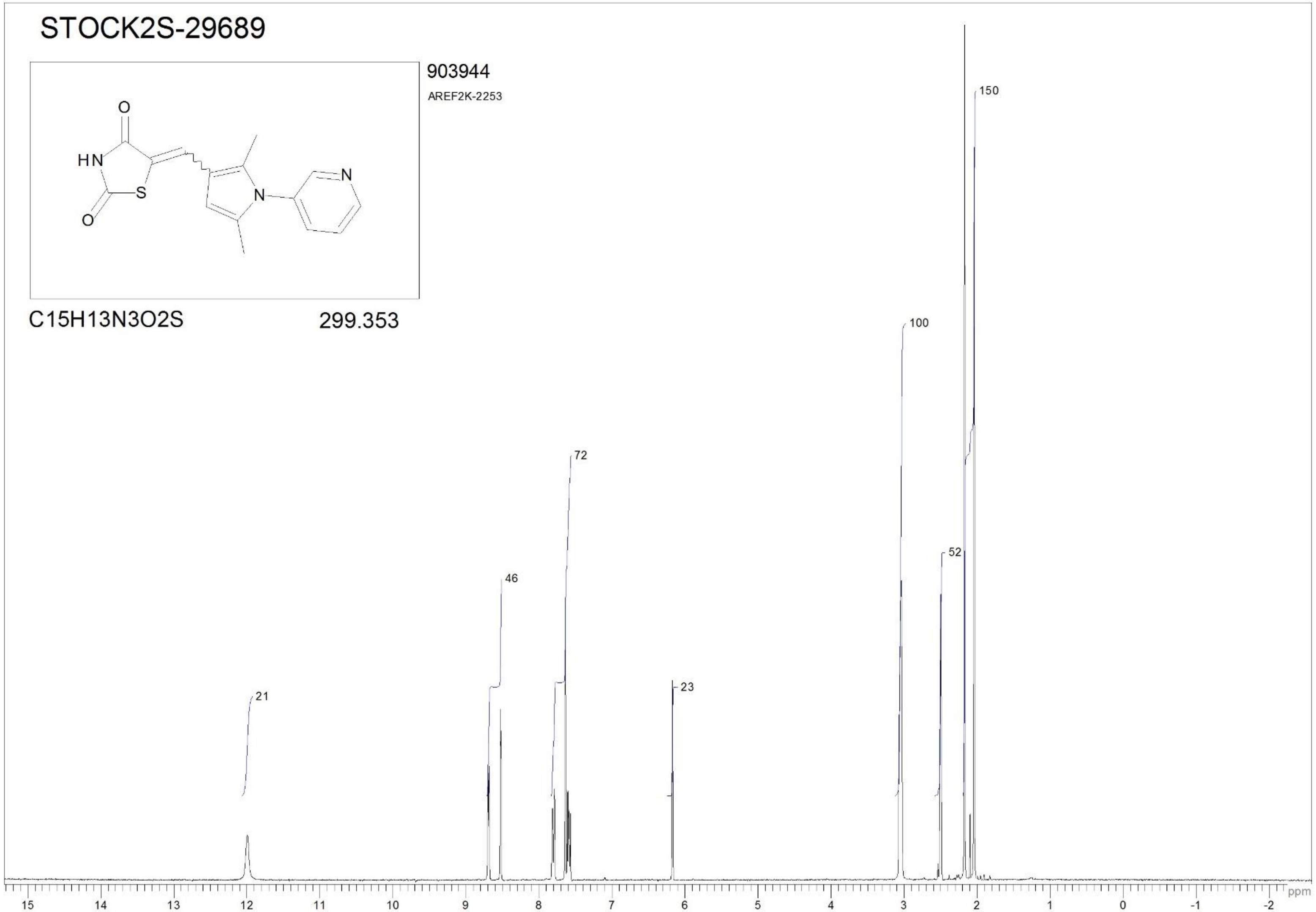

Figure S73. 1H-NMR spectrum of STOCK2S-29689

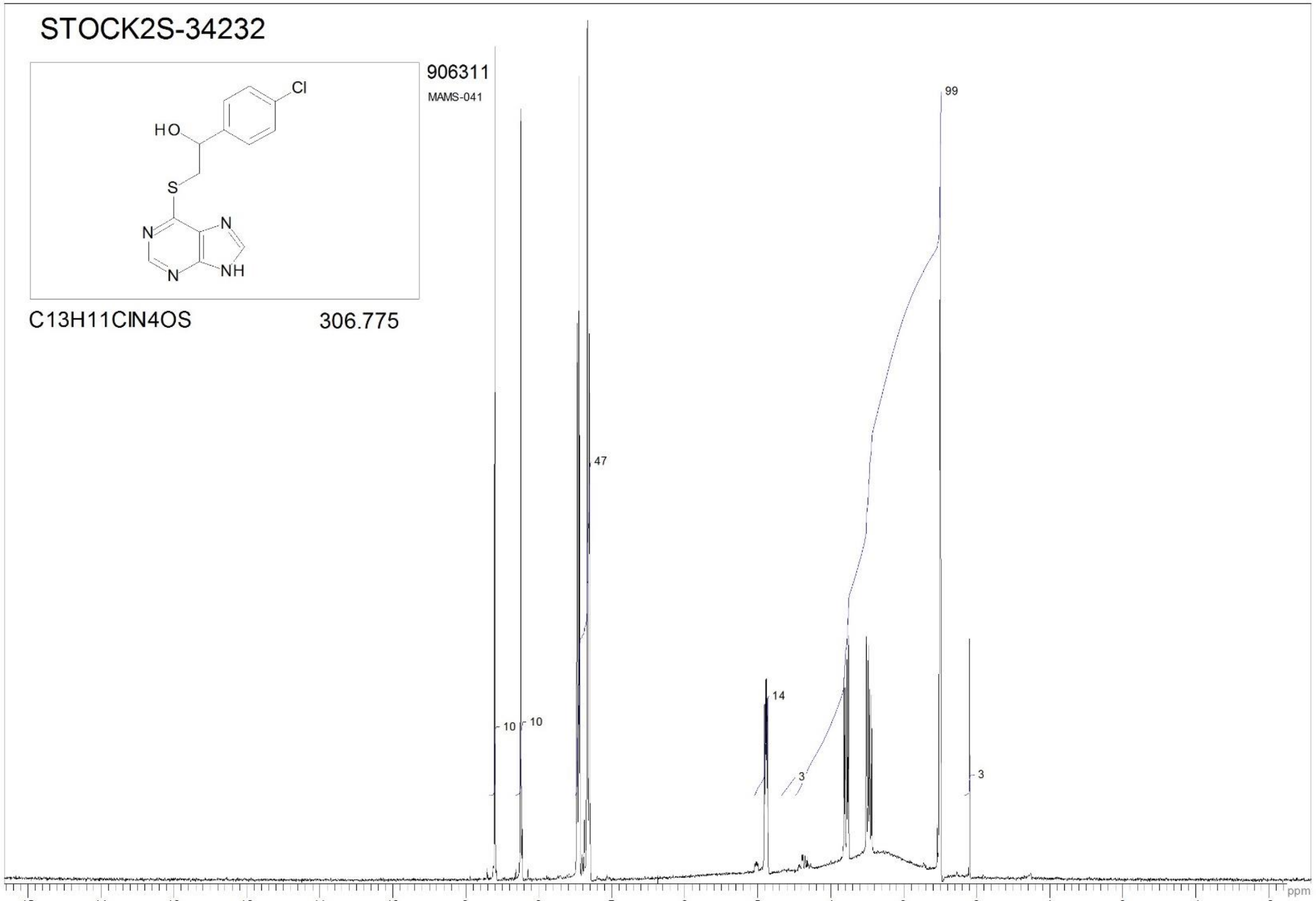

Figure S74. 1H-NMR spectrum of STOCK2S-34232

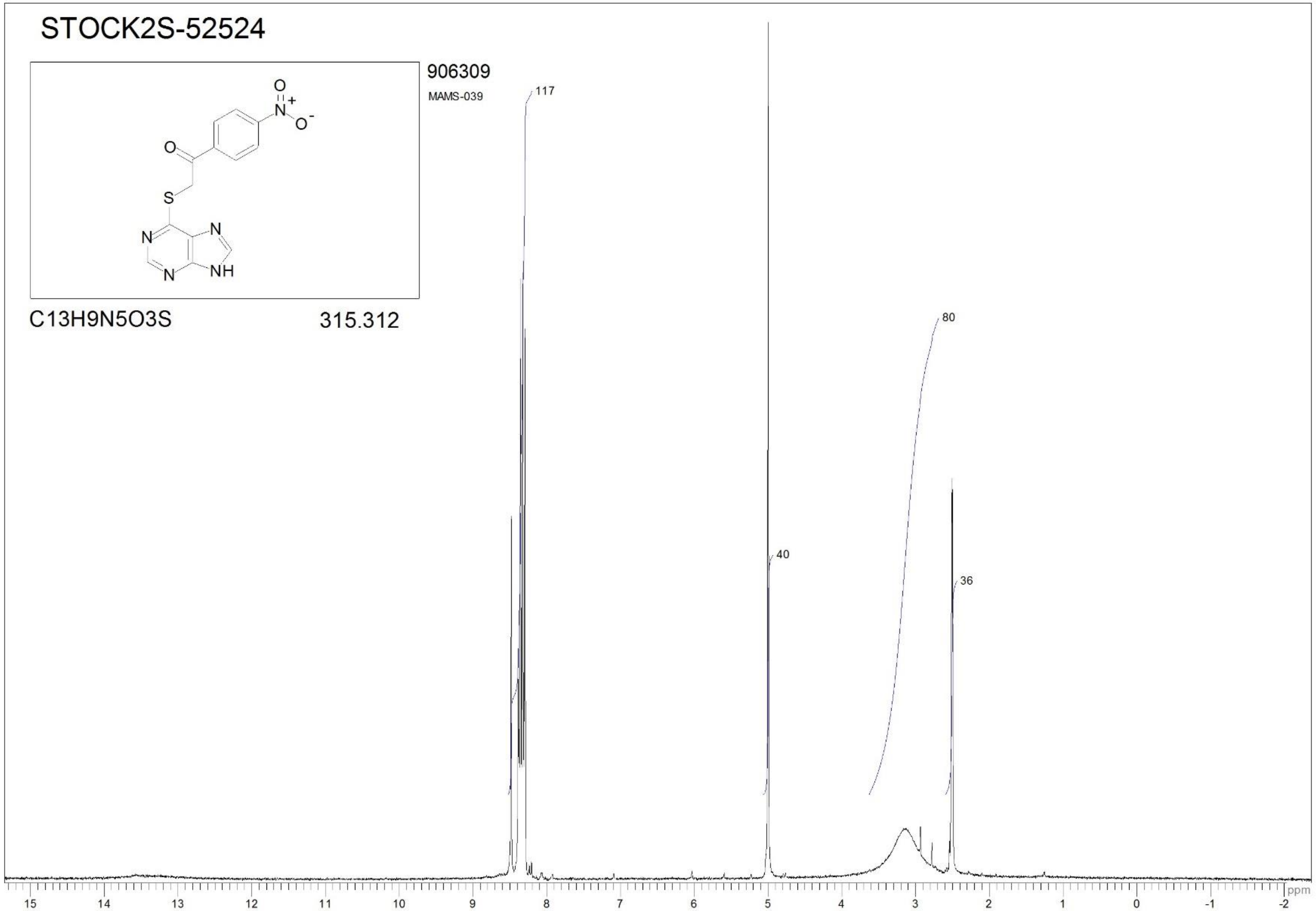

Figure S75. 1H-NMR spectrum of STOCK2S-52524

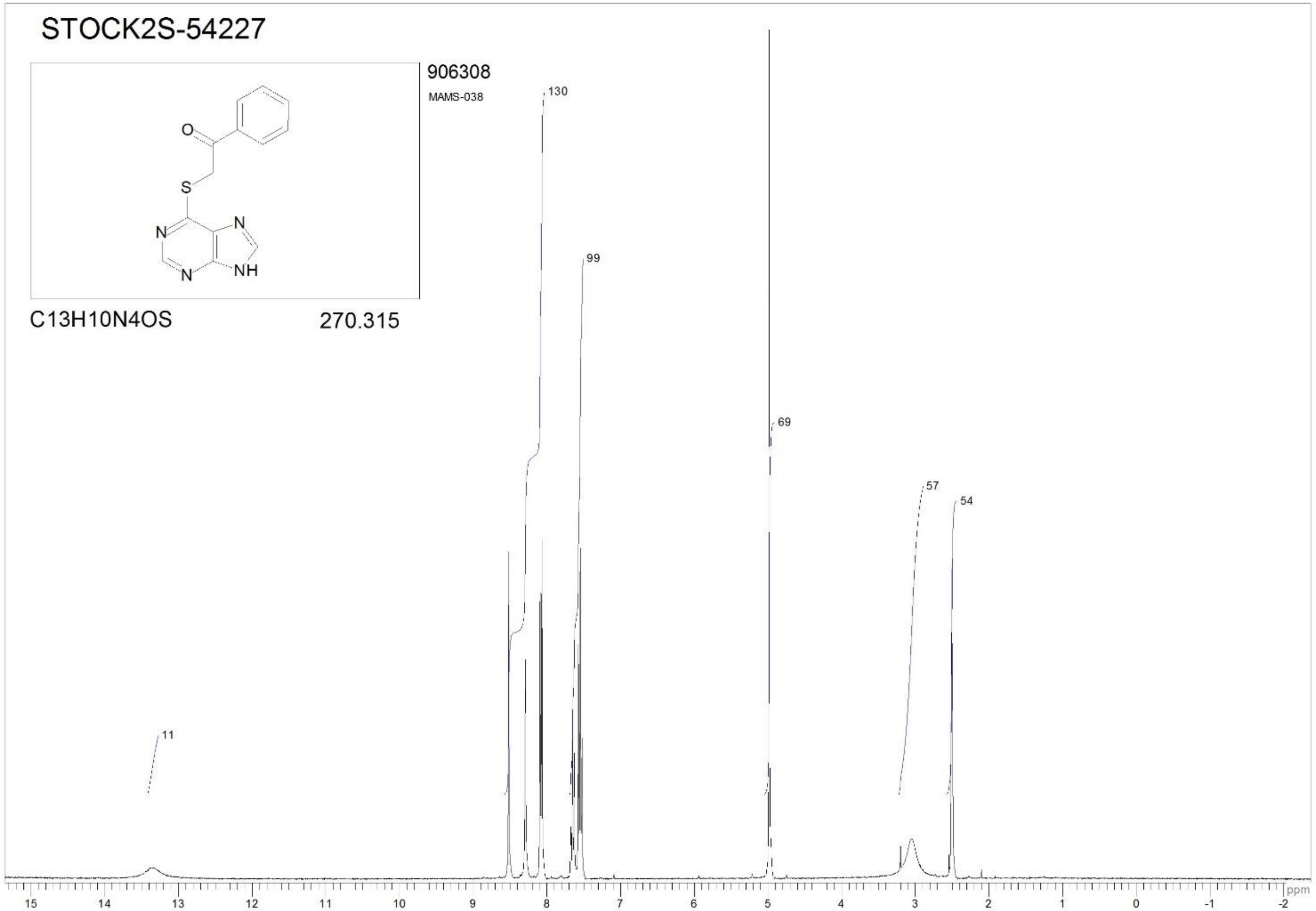

Figure S76. 1H-NMR spectrum of STOCK2S-54227

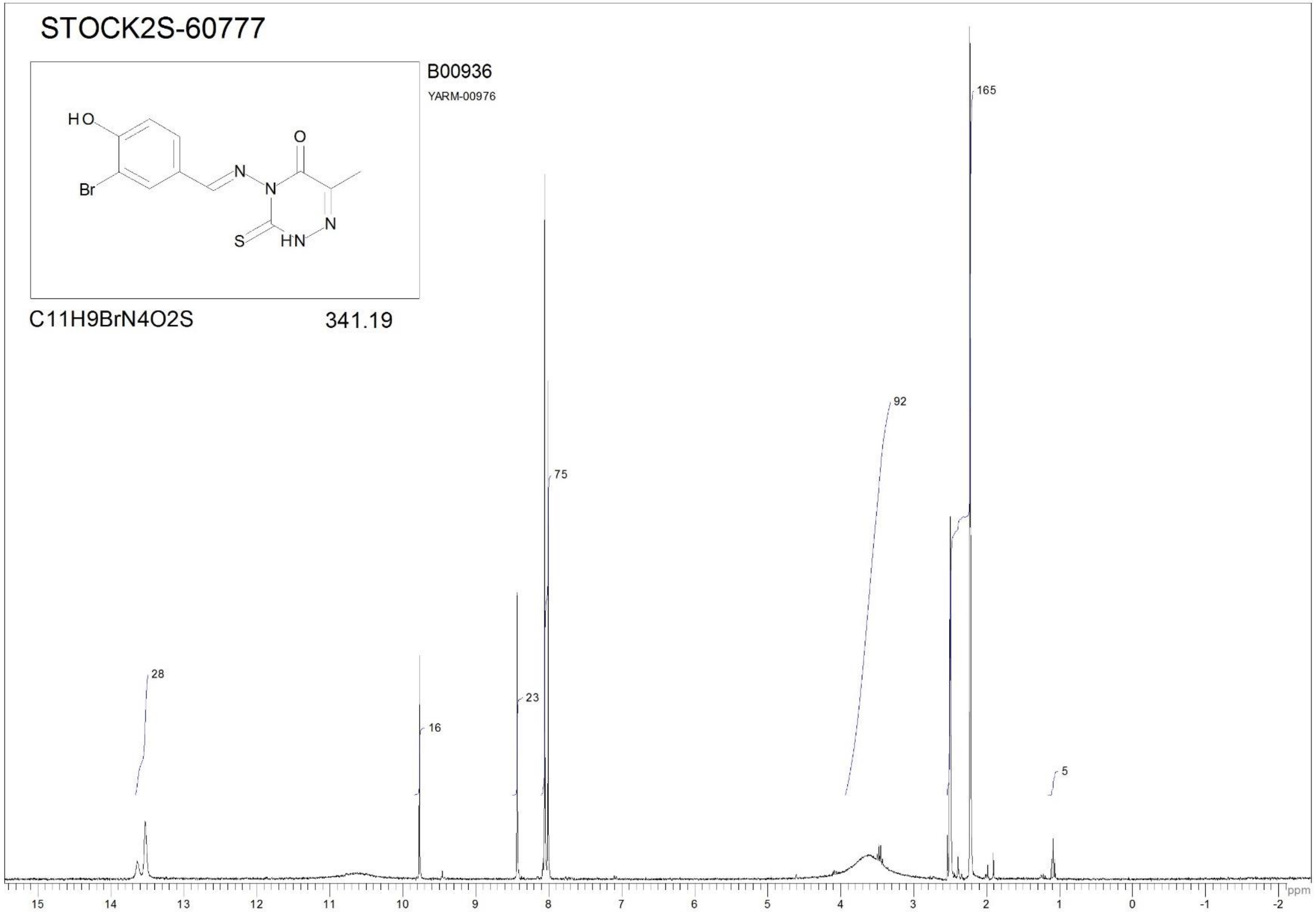

Figure S77. 1H-NMR spectrum of STOCK2S-60777

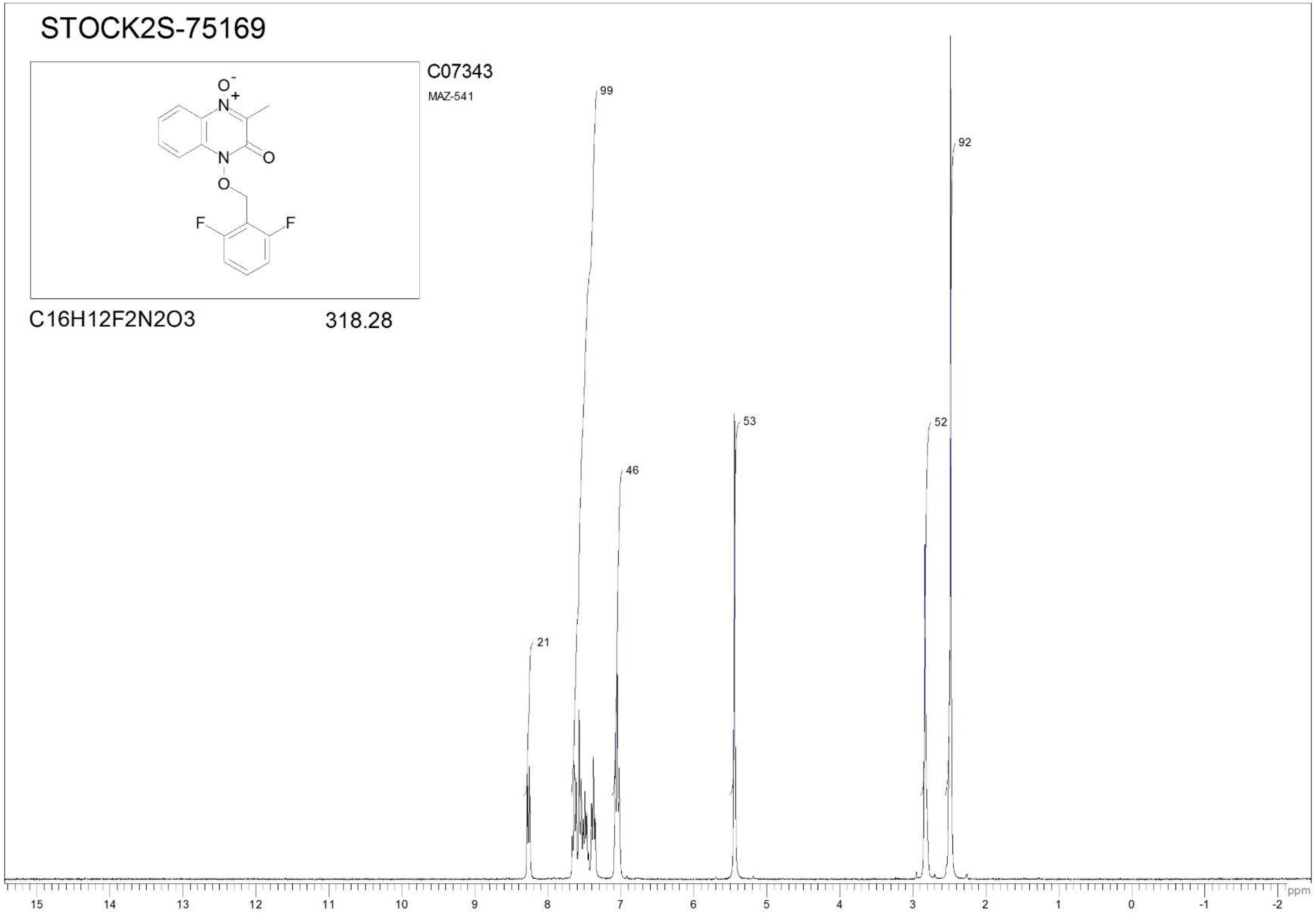

Figure S78. 1H-NMR spectrum of STOCK2S-75169

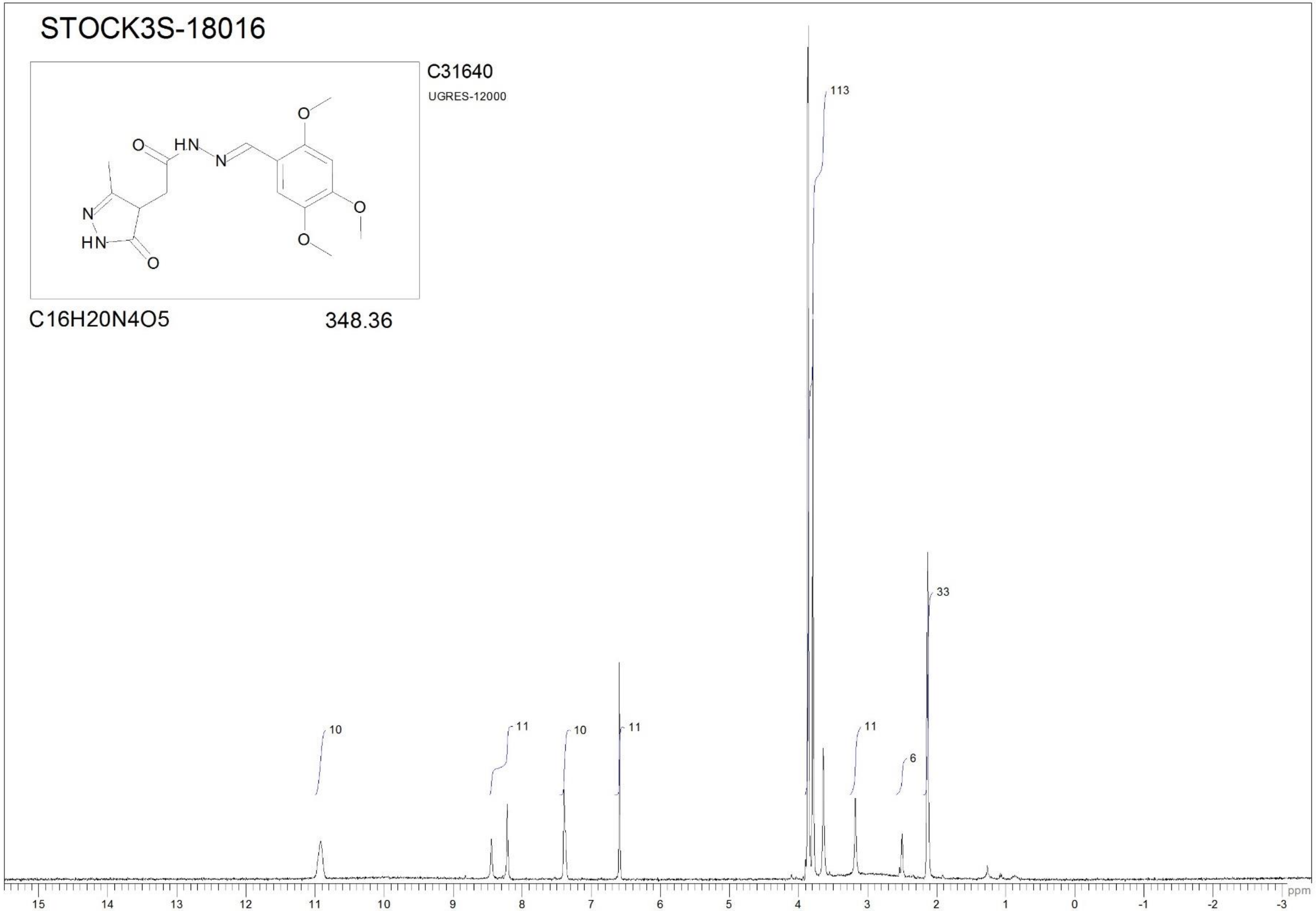

Figure S79. 1H-NMR spectrum of STOCK3S-18016

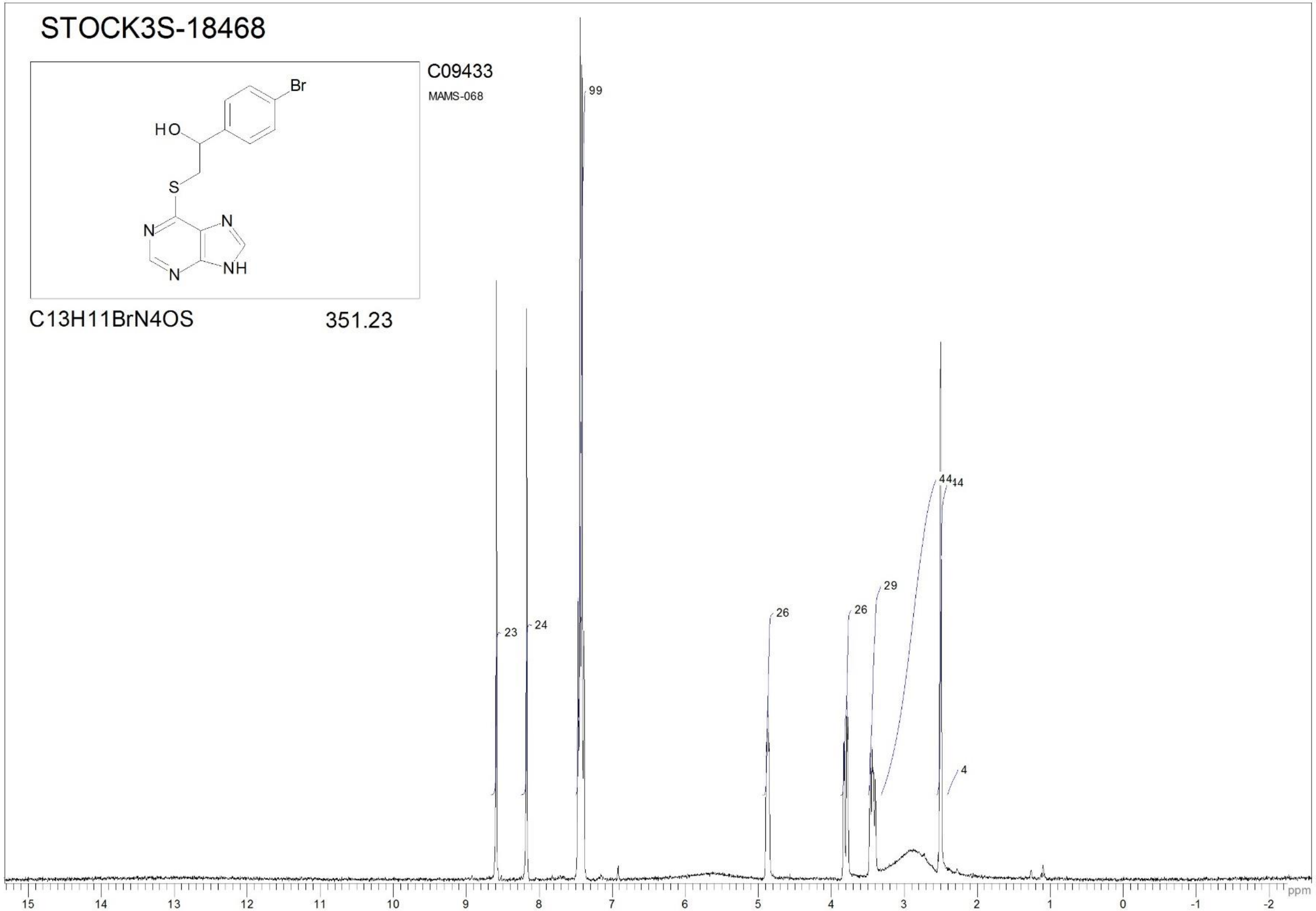

Figure S80. 1H-NMR spectrum of STOCK3S-18468

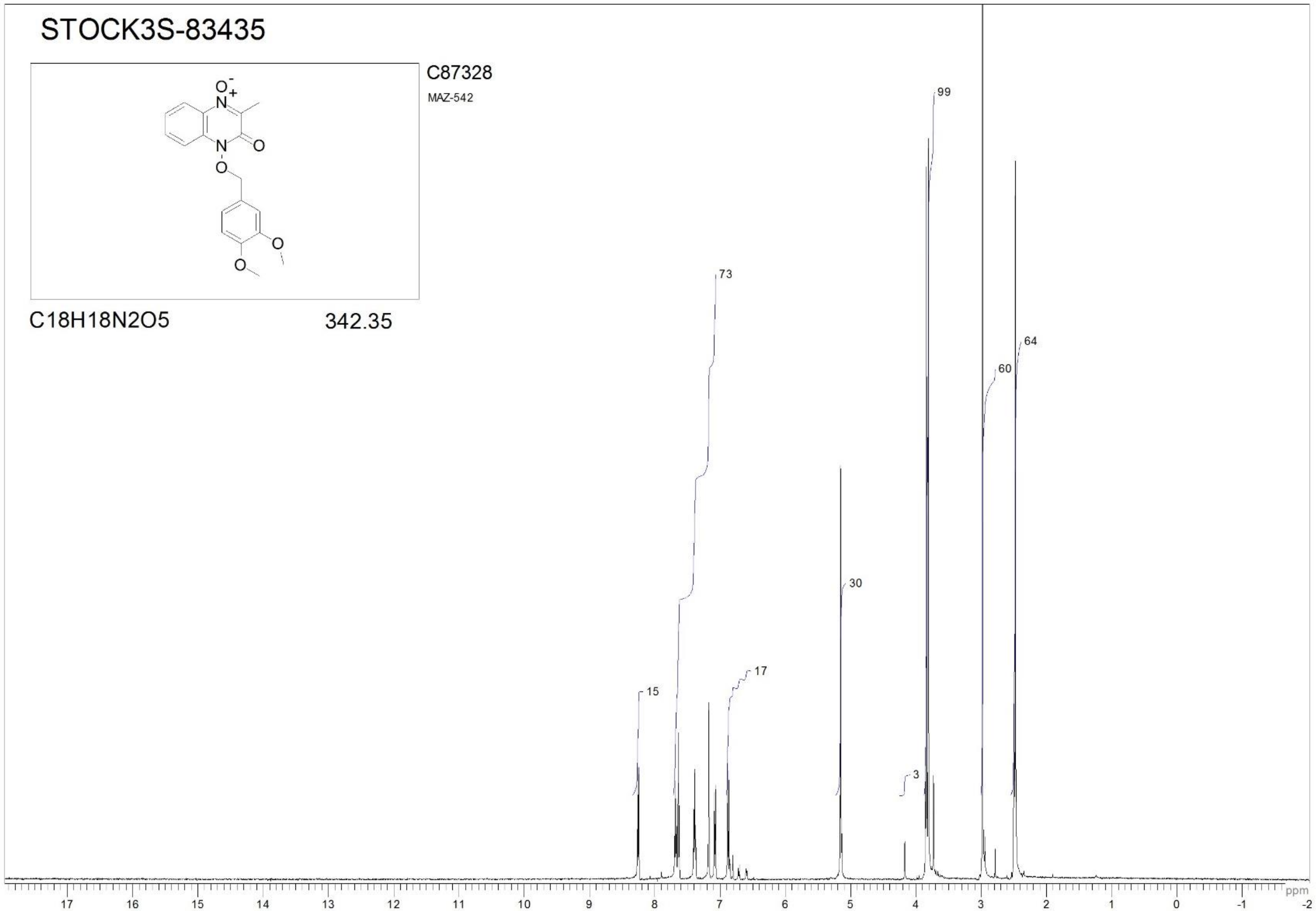

Figure S81. 1H-NMR spectrum of STOCK3S-83435

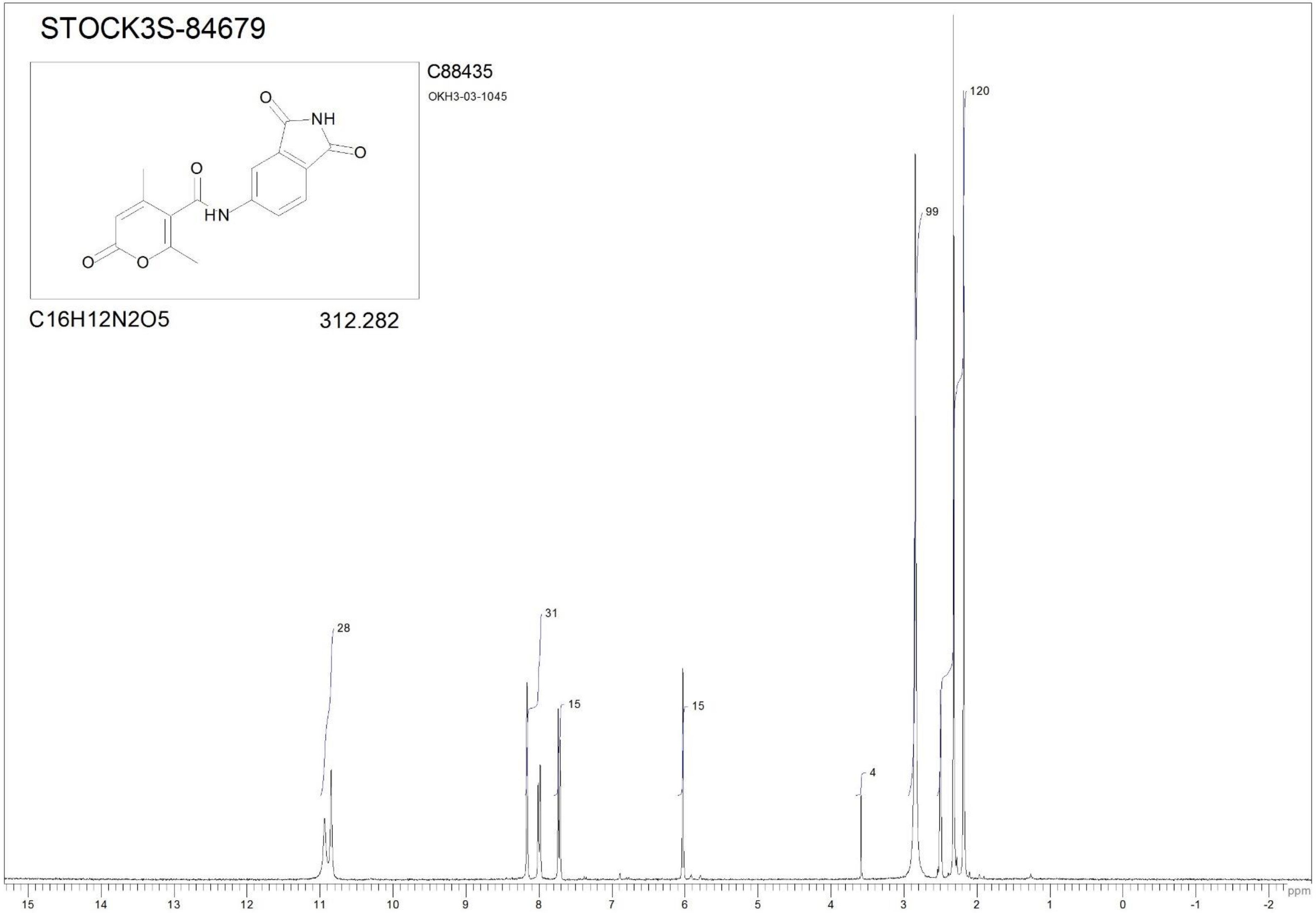

Figure S82. 1H-NMR spectrum of STOCK3S-84679

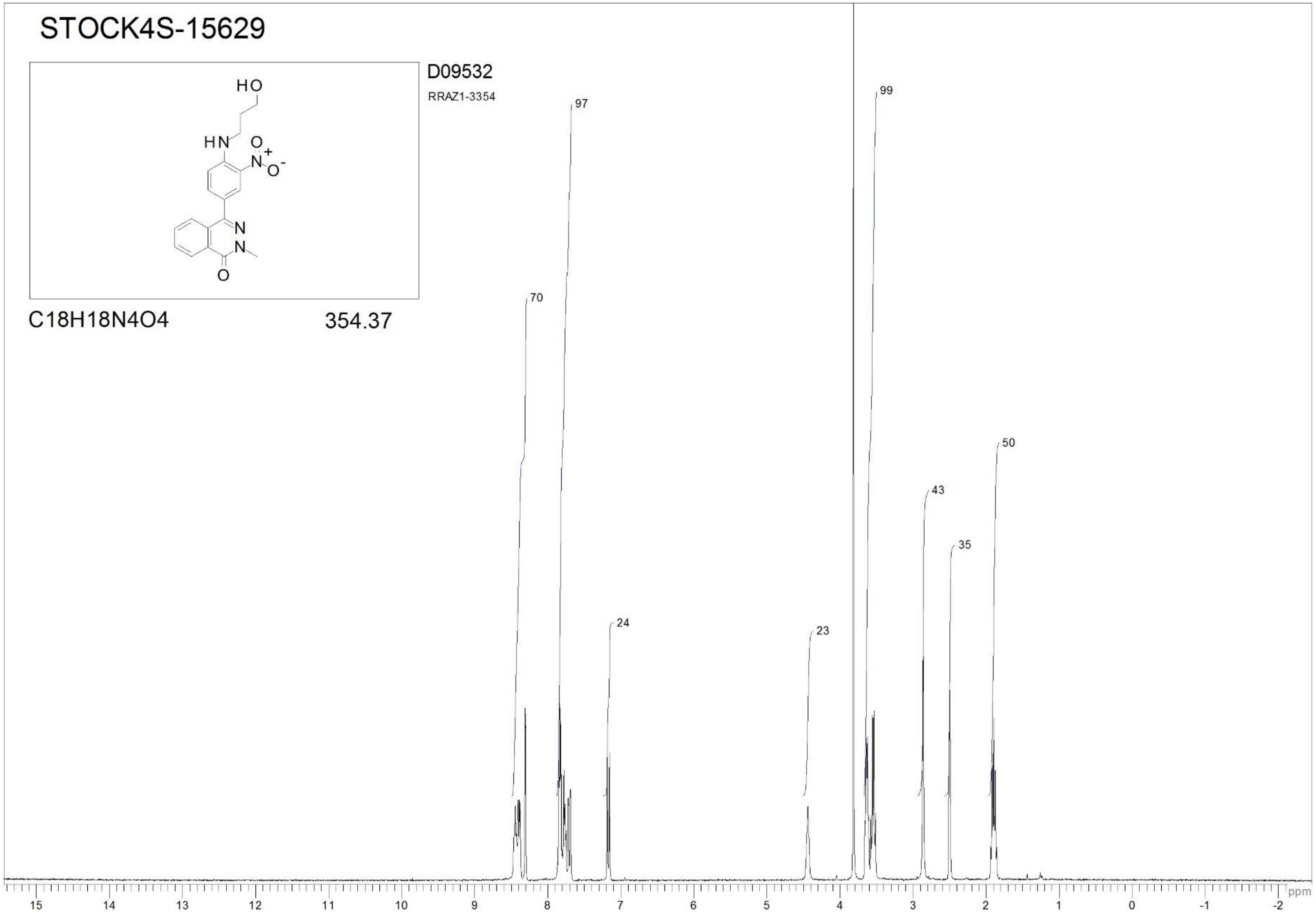

Figure S83. 1H-NMR spectrum of STOCK4S-15629

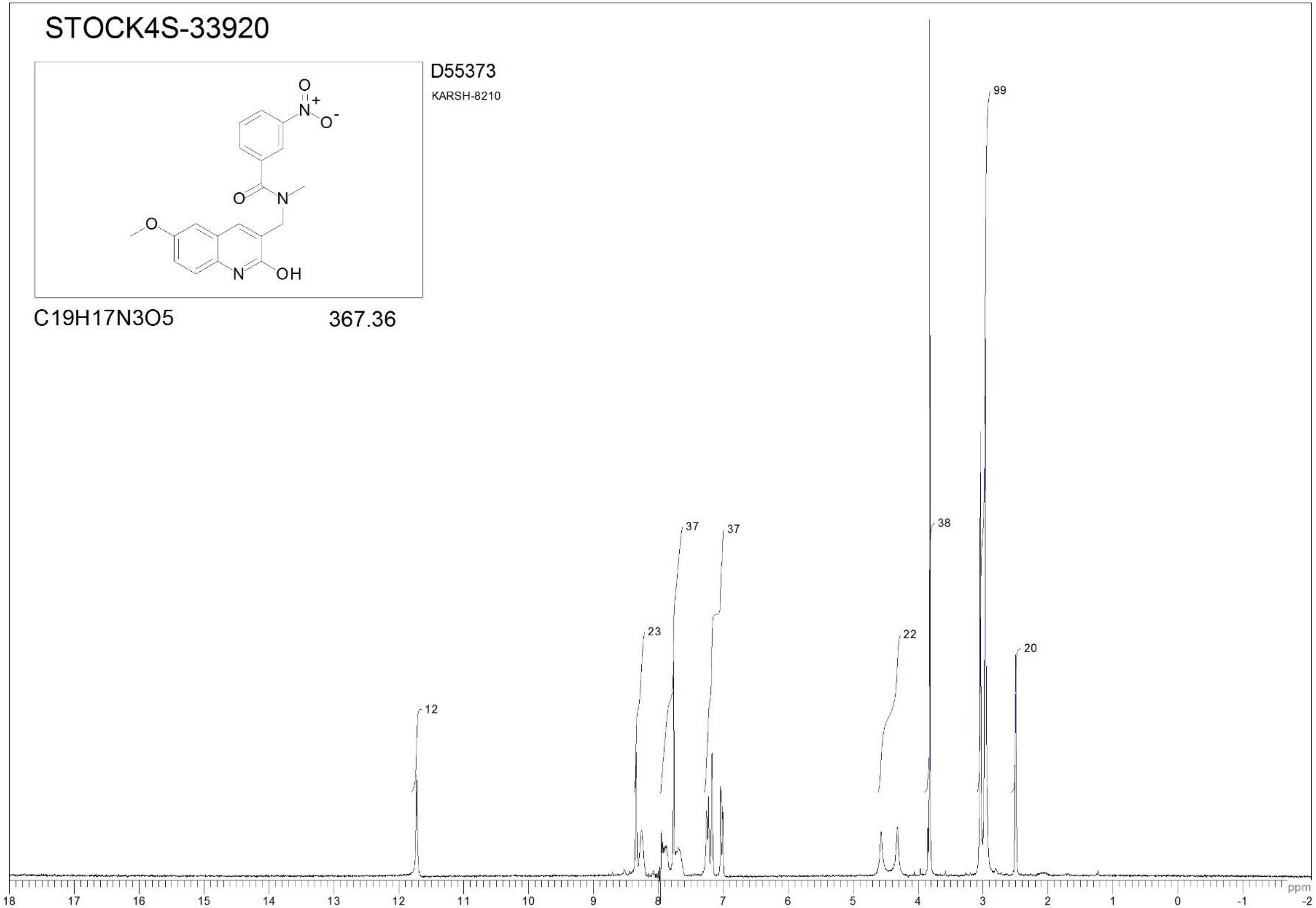

Figure S84. 1H-NMR spectrum of STOCK4S-33920

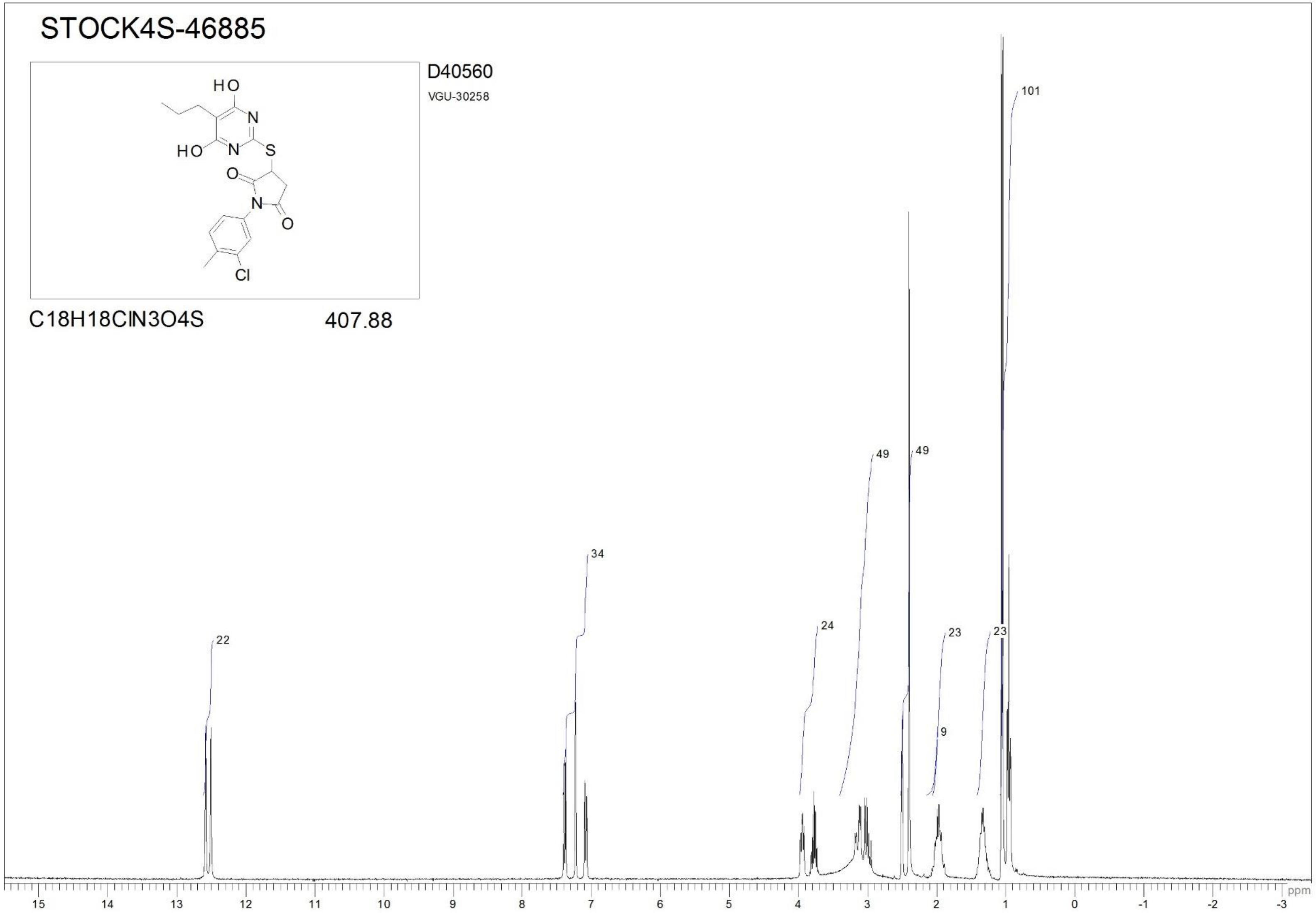

Figure S85. 1H-NMR spectrum of STOCK4S-46885

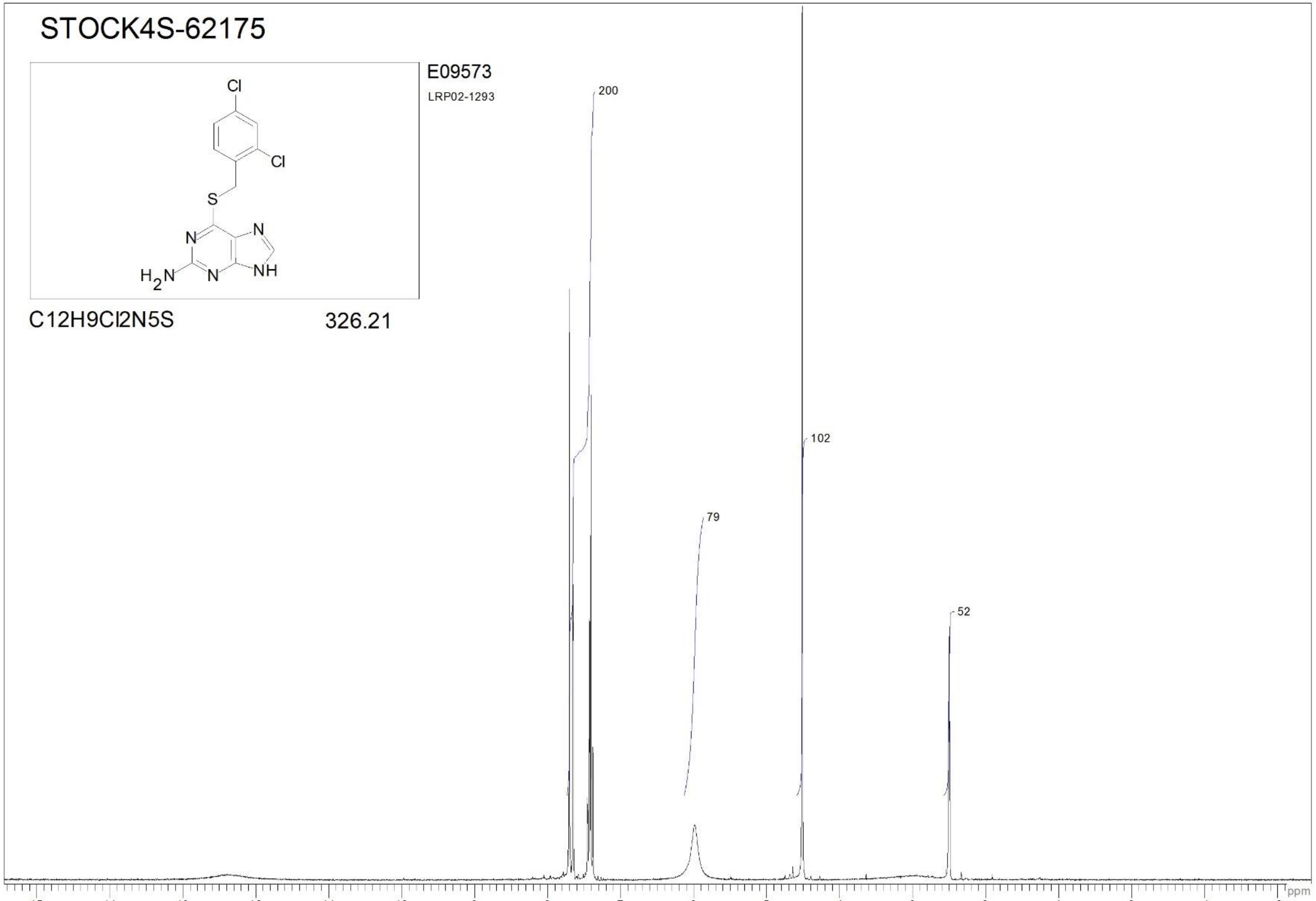

Figure S86. 1H-NMR spectrum of STOCK4S-62175

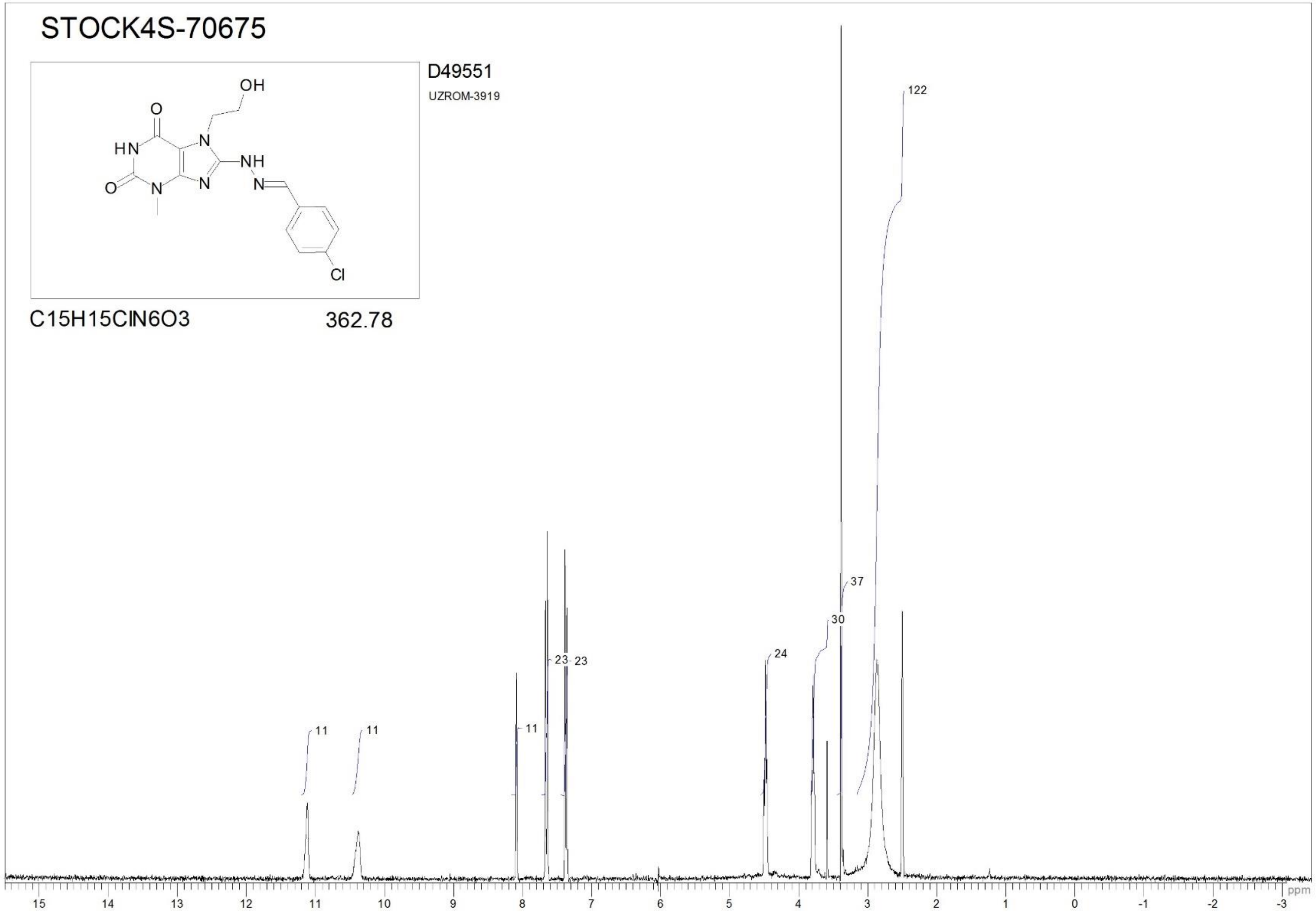

Figure S87. 1H-NMR spectrum of STOCK4S-70675

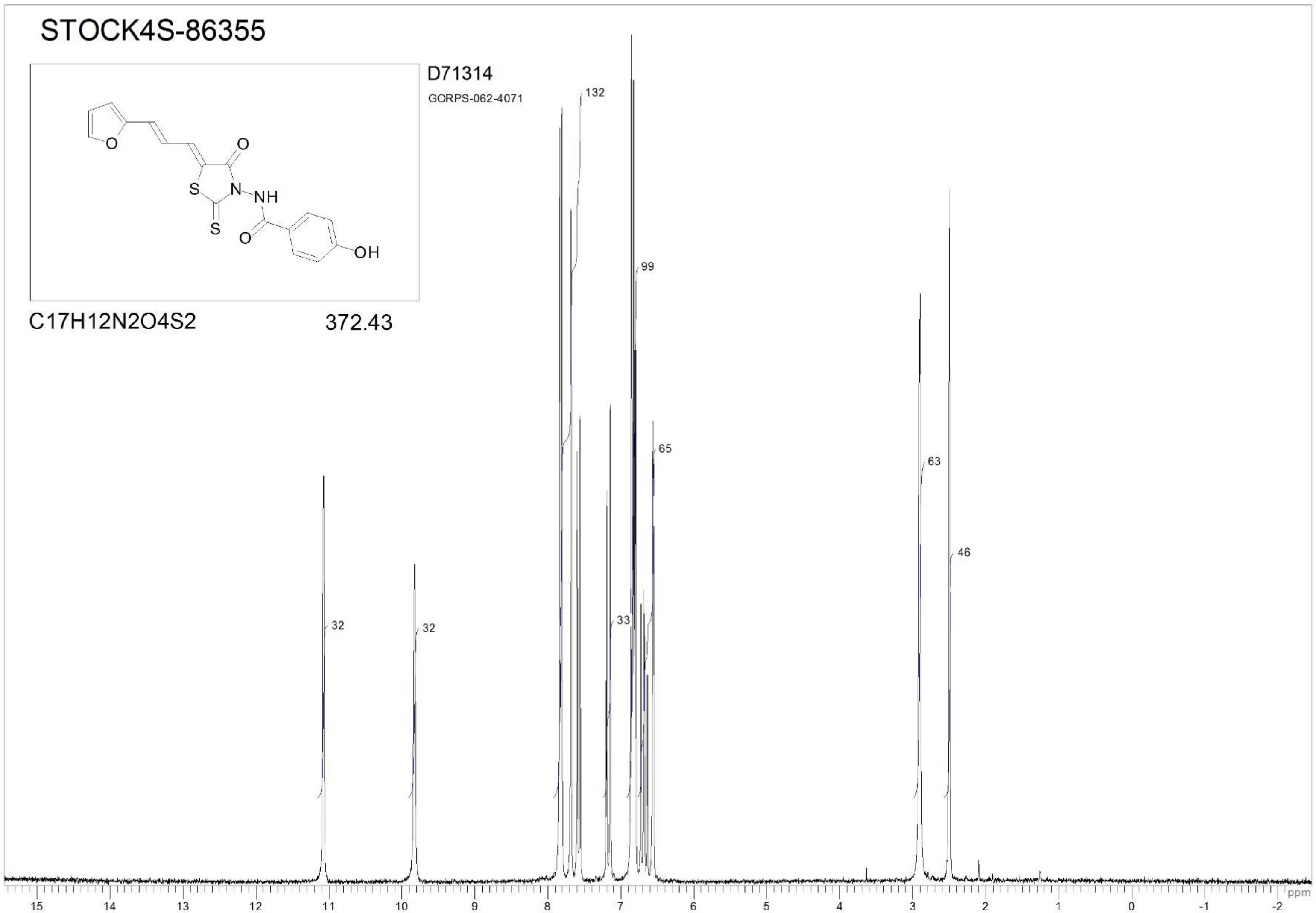

Figure S88. 1H-NMR spectrum of STOCK4S-86355

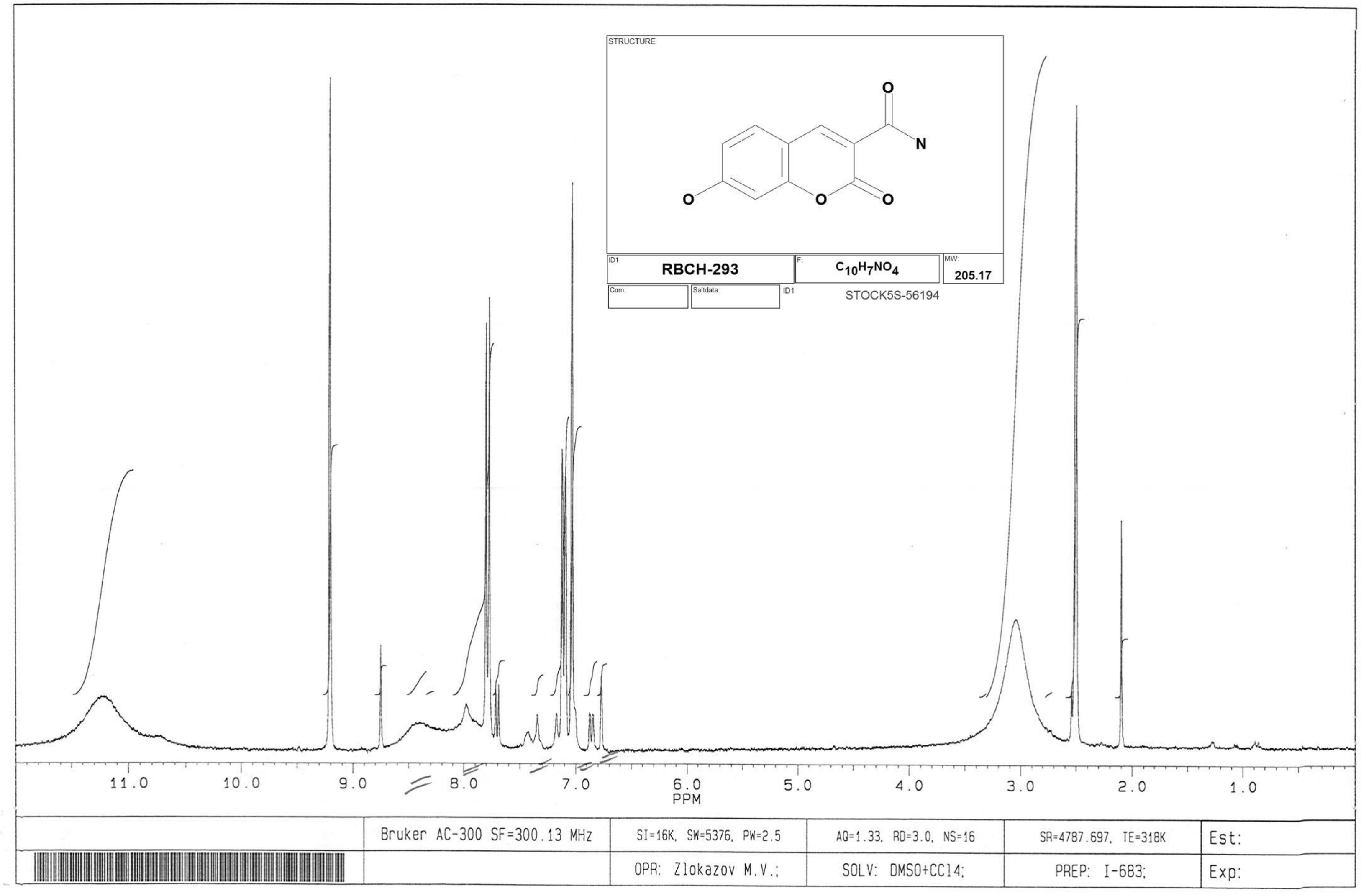

Figure S89. 1H-NMR spectrum of STOCK5S-56194

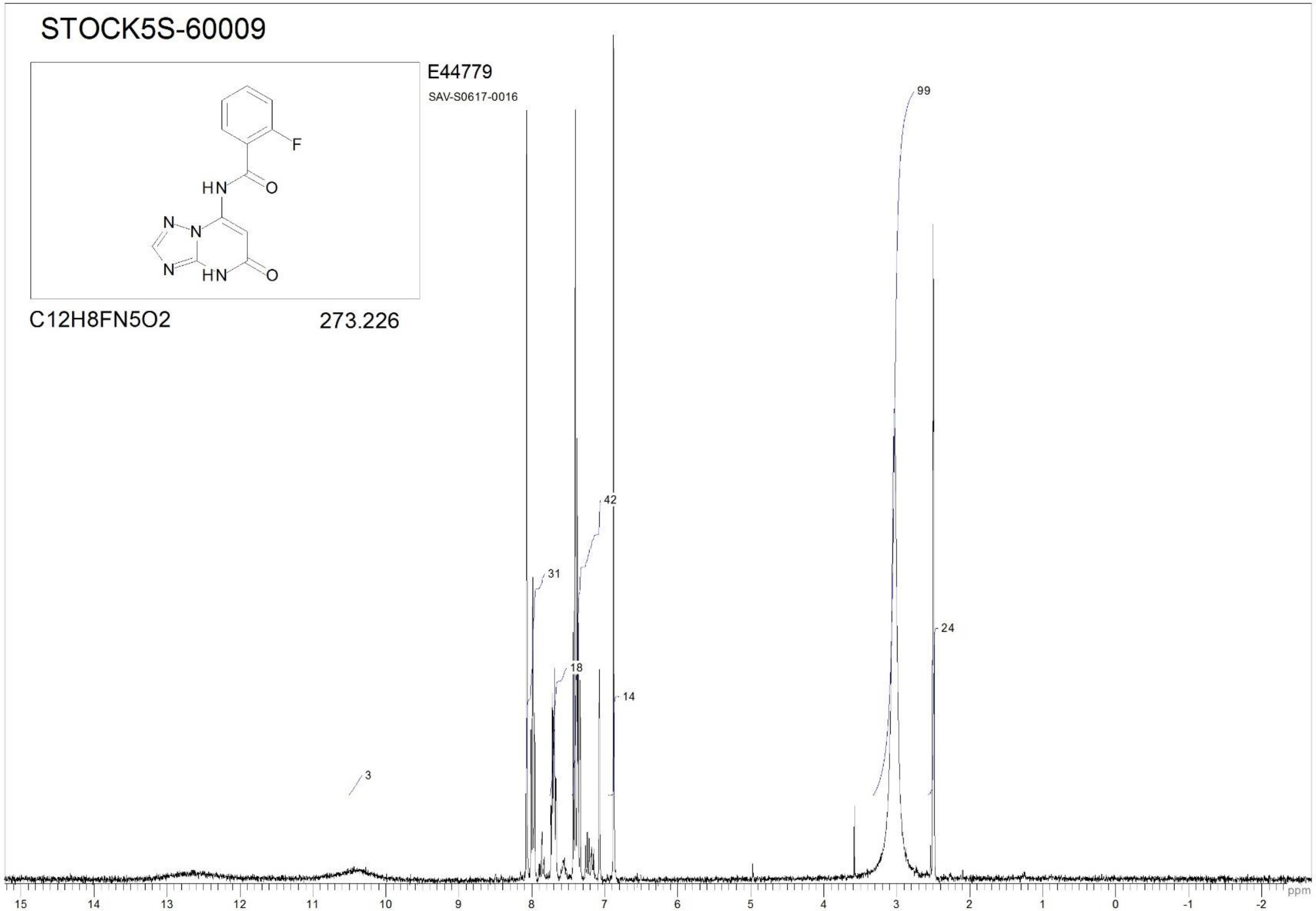

Figure S90. 1H-NMR spectrum of STOCK5S-60009

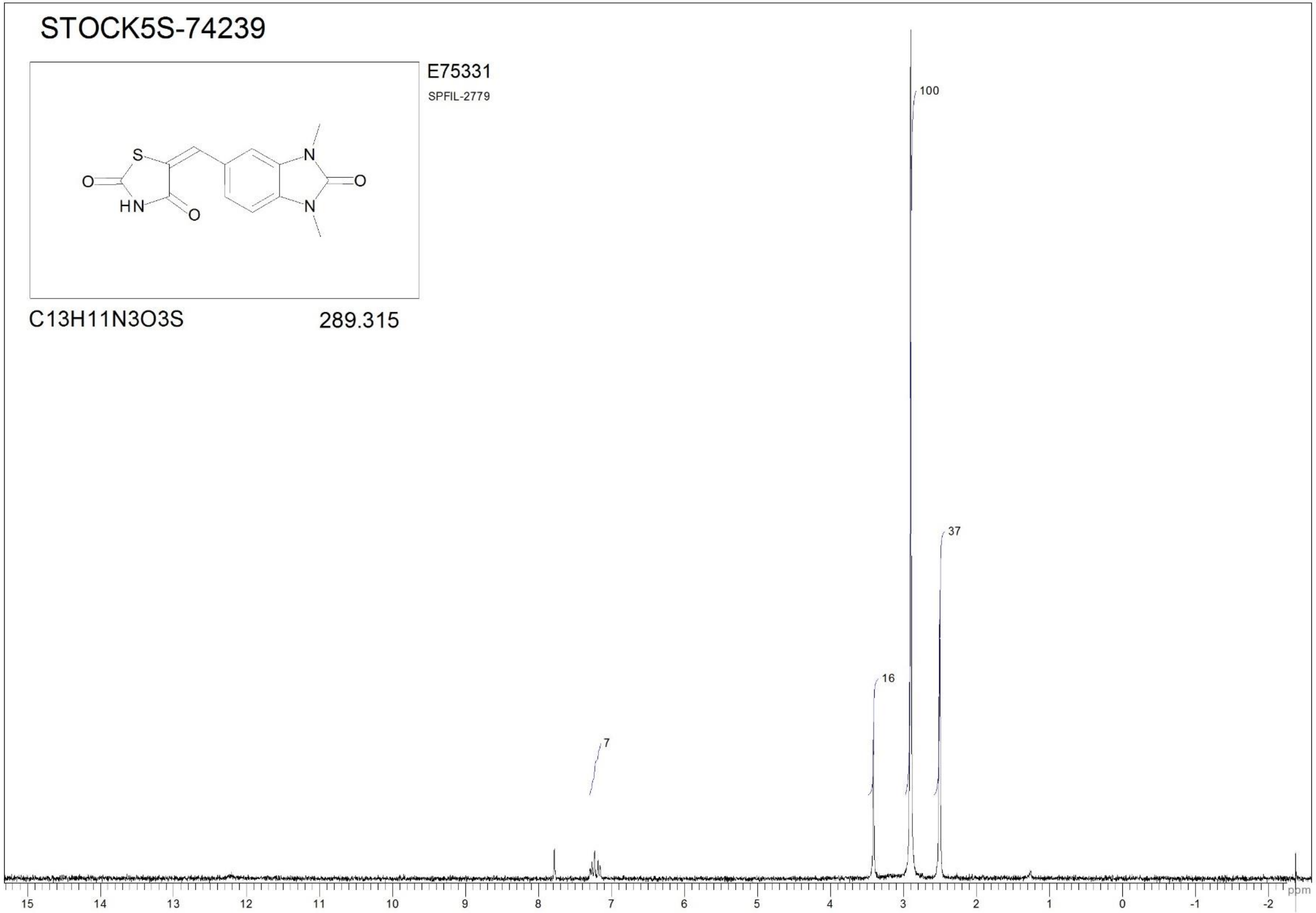

Figure S91. 1H-NMR spectrum of STOCK5S-74239

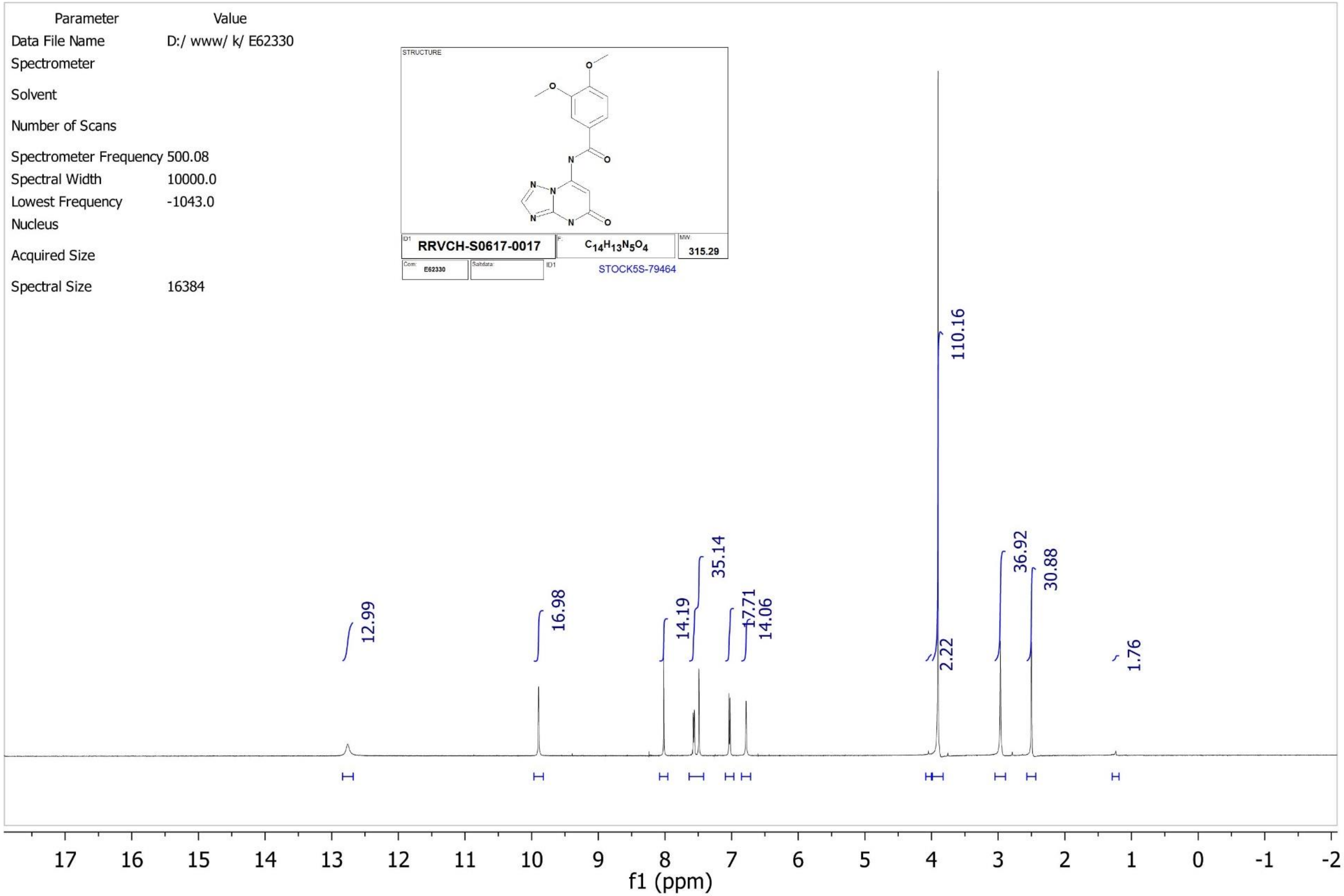

Figure S92. 1H-NMR spectrum of STOCK5S-79464

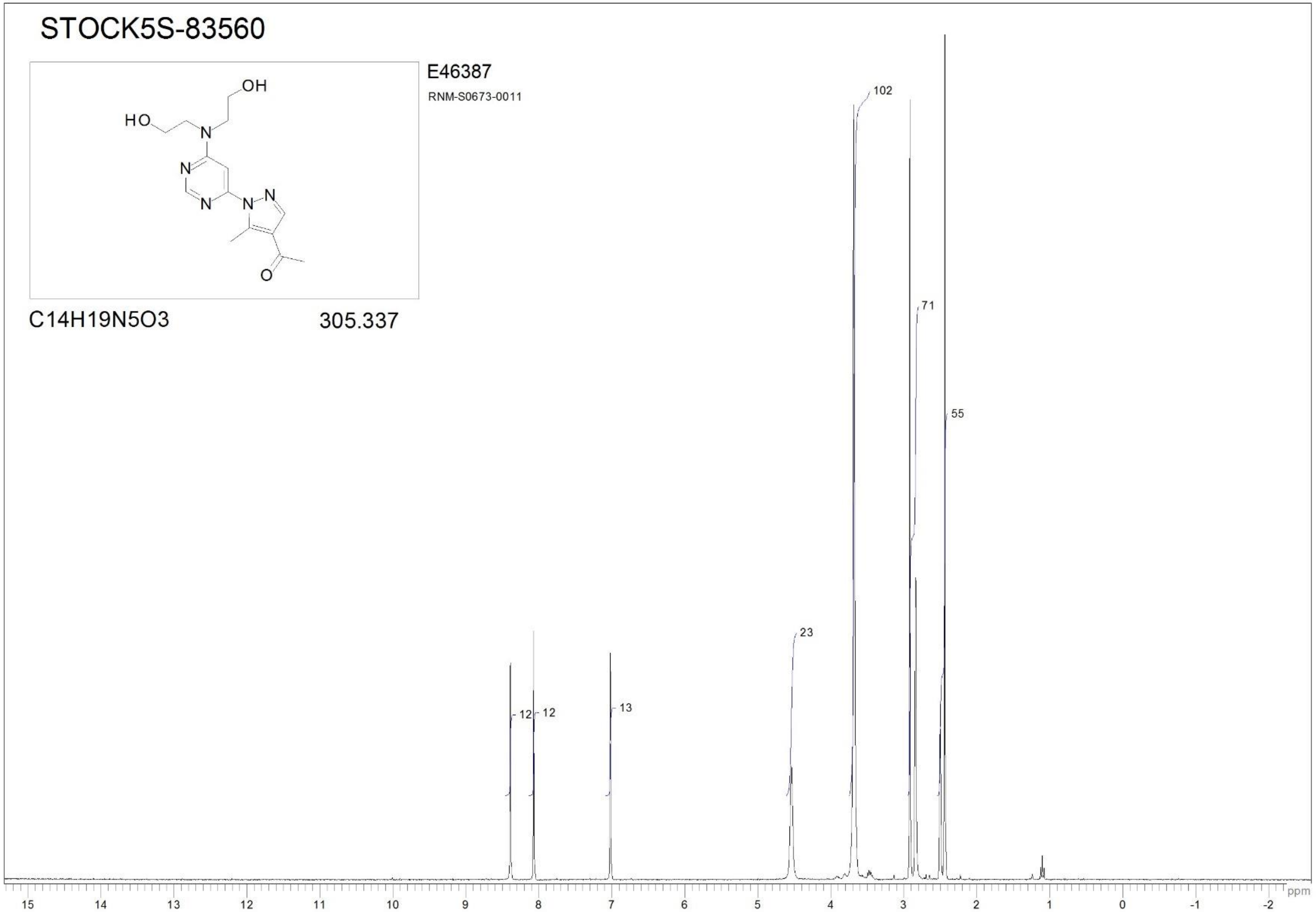

Figure S93. 1H-NMR spectrum of STOCK5S-83560

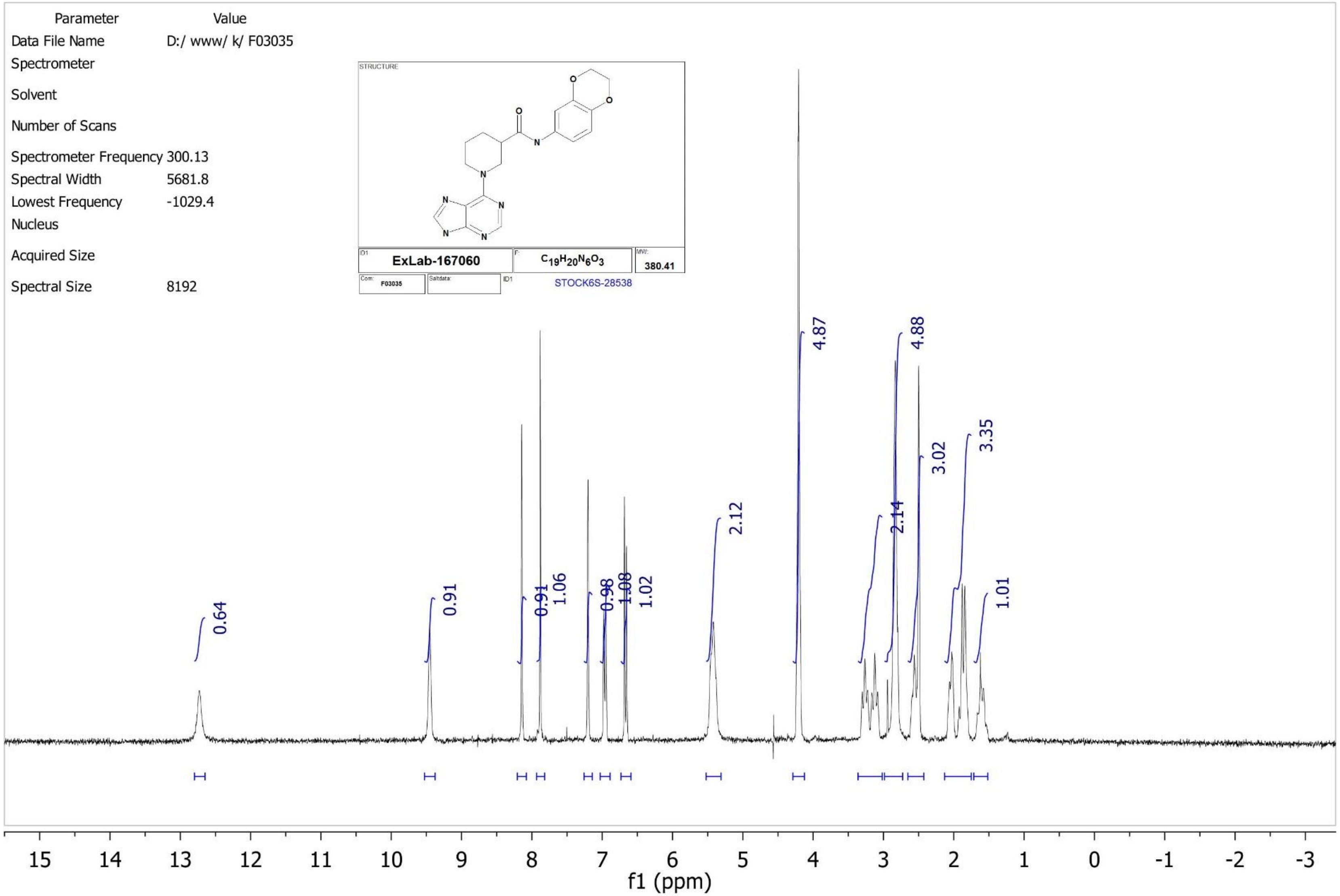

Figure S94. 1H-NMR spectrum of STOCK6S-28538

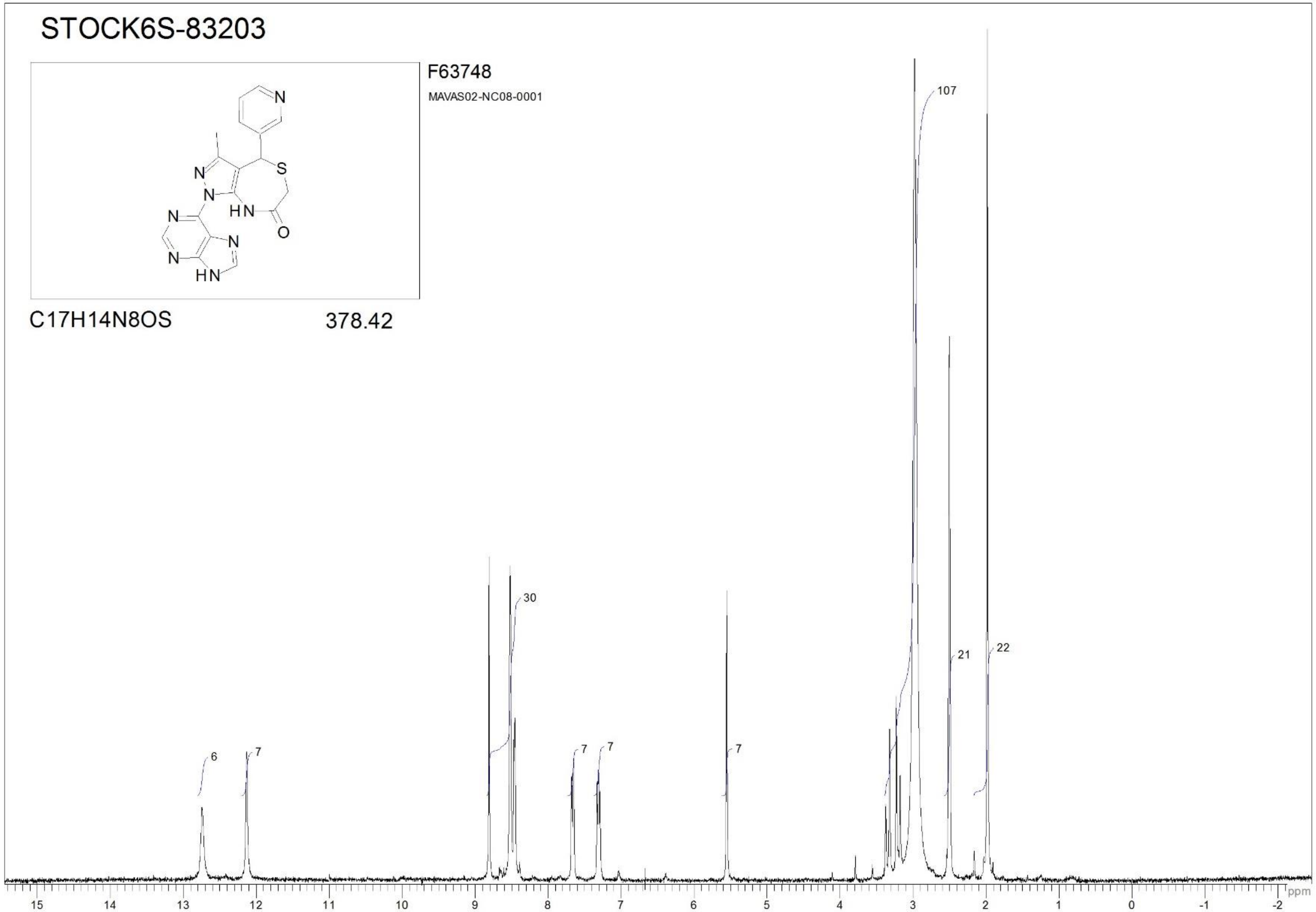

Figure S95. 1H-NMR spectrum of STOCK6S-83203

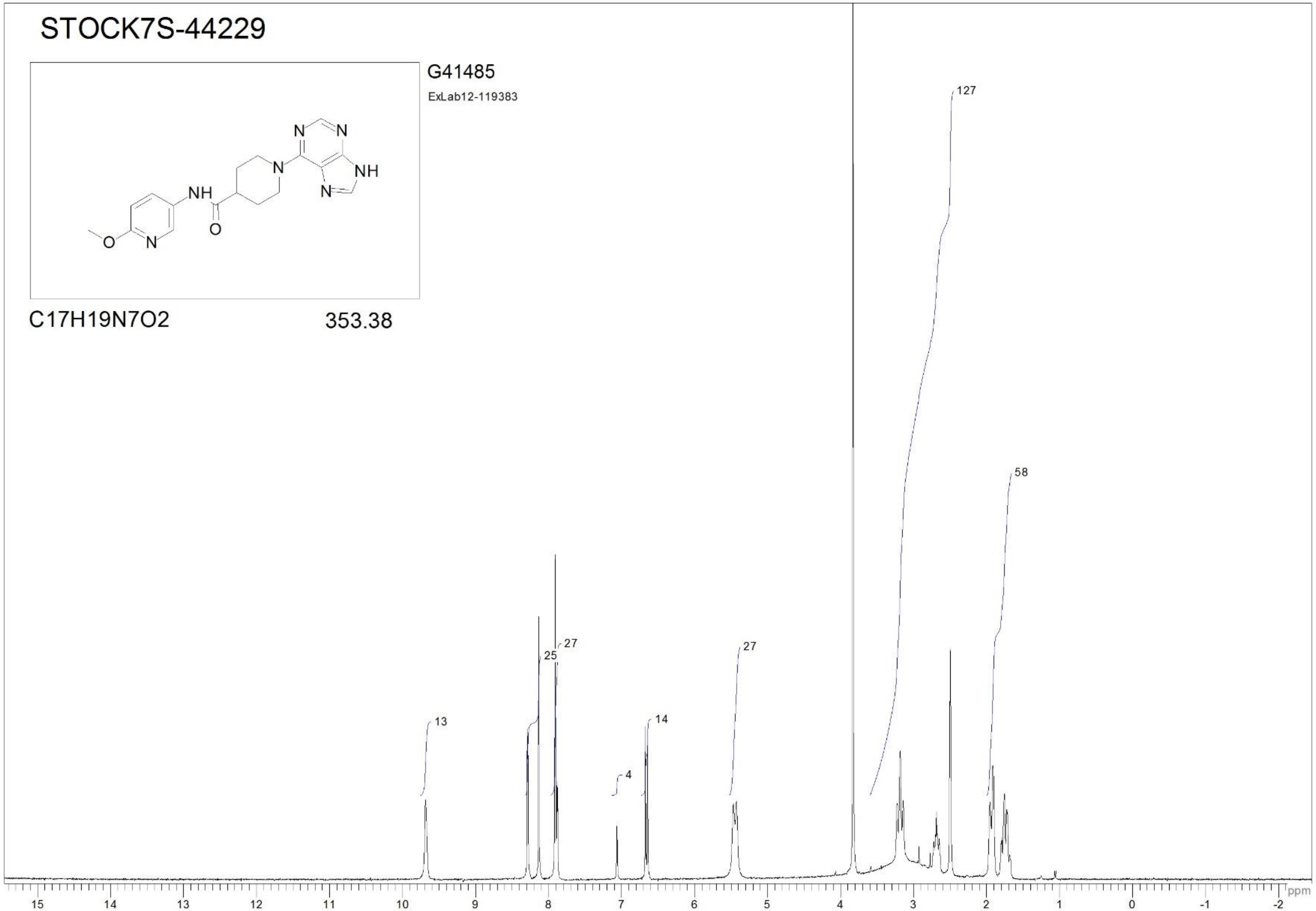

Figure S96. 1H-NMR spectrum of STOCK7S-44229

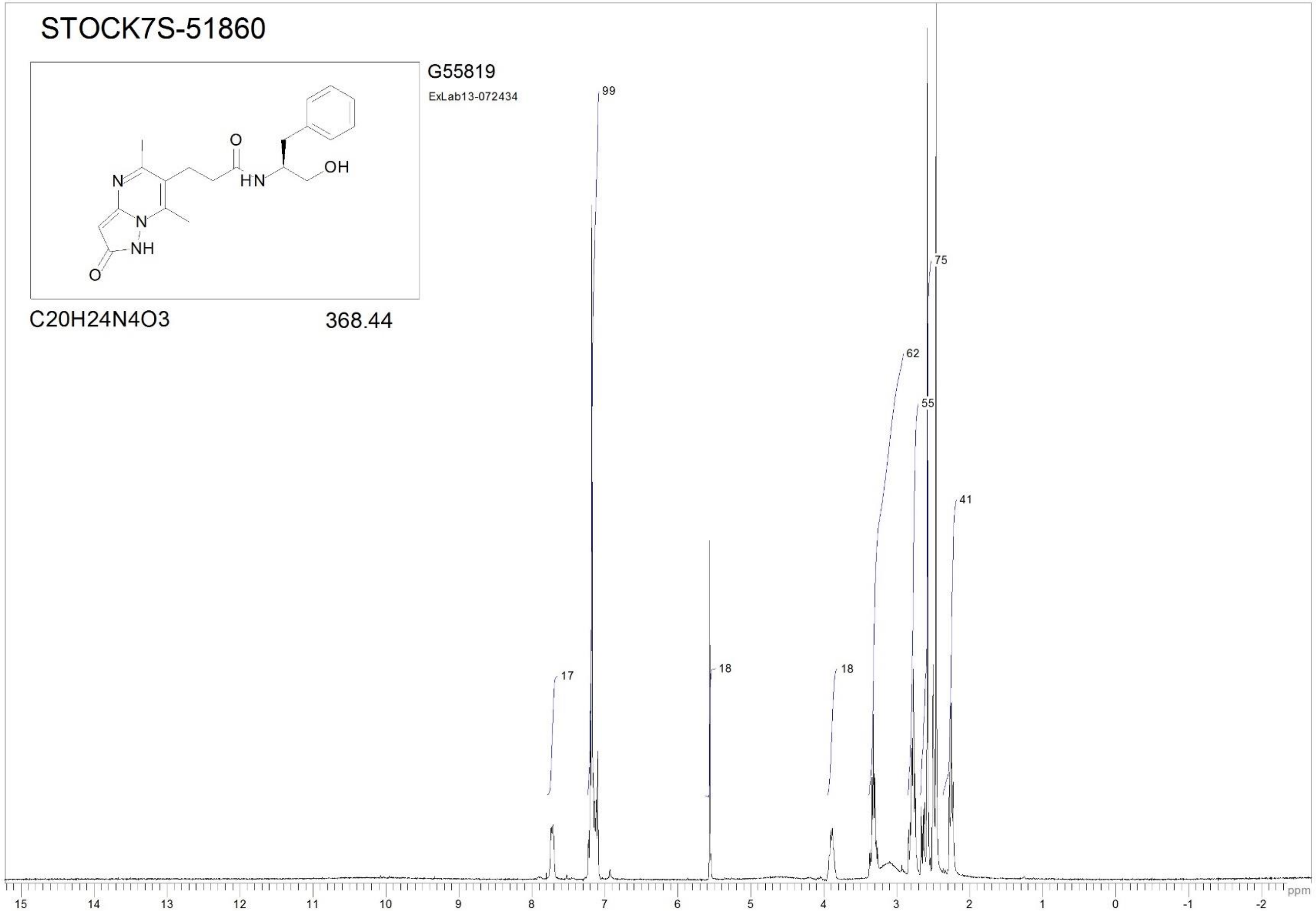

Figure S97. 1H-NMR spectrum of STOCK7S-51860

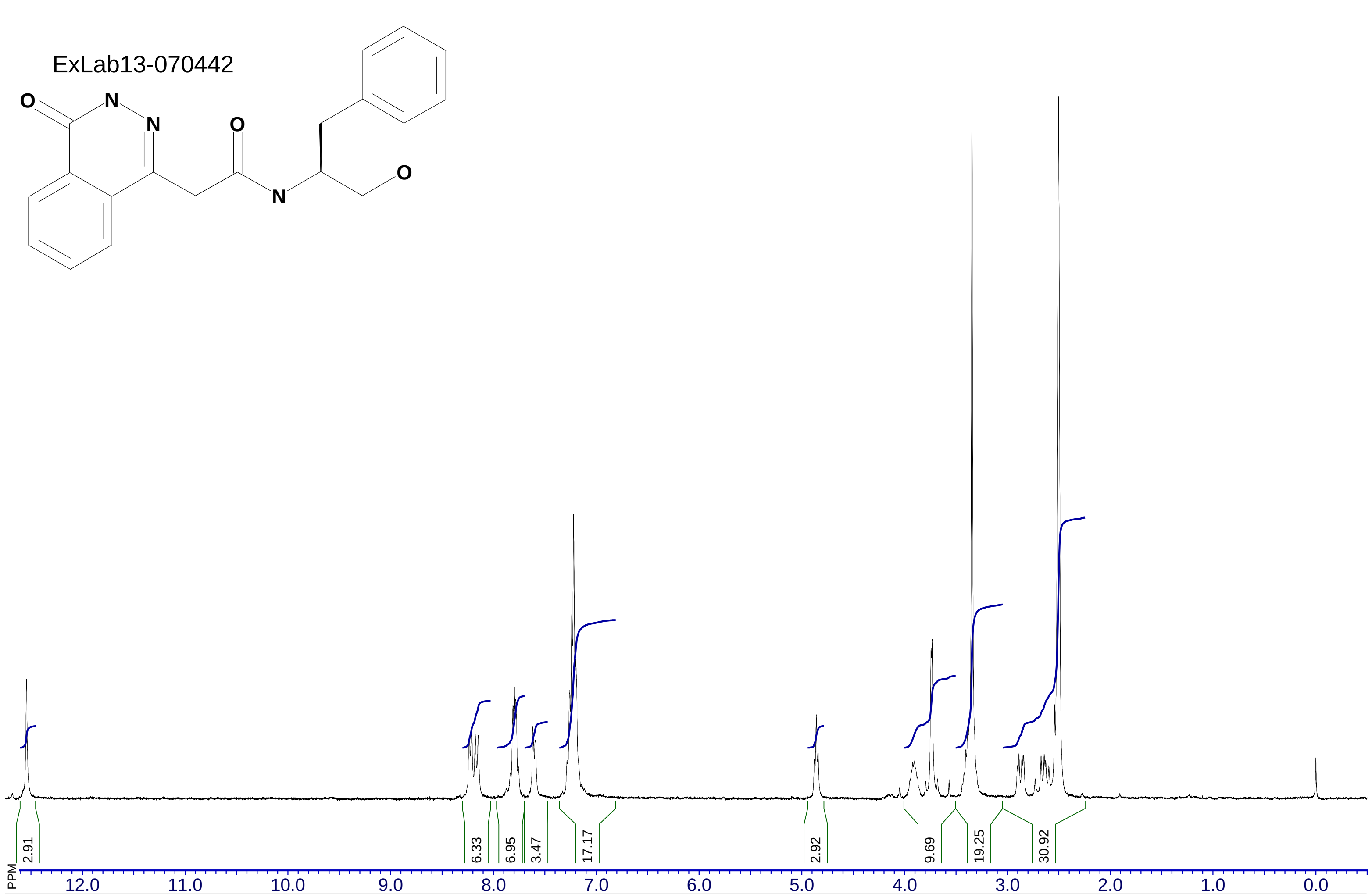

**File name: ExLab13-070442**	Owner:	SF: 299.9450 MHz	NS: 2	SI: 32768, TD: 17472
Date:	Solvent:	SW: 5099	TE: 300	

Figure S98. 1H-NMR spectrum of STOCK7S-53671

April 2025

CERTIFICATE OF ANALYSIS

	ID	mol weight	fmla STRUCTURE	Batch	Purity (quality control on a Bruker-400 MHz NMR spectrometer): not less	iupac name
1	STOCK1N-12057	290,365	C16H22N2O3	B9-2146	>=92%	4-((1r,3r,5r,7r)-6-hydroxy-5,7-dimethyl-1,3-diazaadamantan-2-yl)benzene-1,3-diol
2	STOCK1N-31772	226,259	C8H10N4O2S	C2-8060	>=92%	3-((9H-purin-6-yl)thio)propane-1,2-diol
3	STOCK1N-51560	283,246	C10H13N5O5	B6-2671	>=95%	6-amino-9-((2R,3R,4S,5R)-3,4-dihydroxy-5-(hydroxymethyl)tetrahydrofuran-2-yl)-9H-purine 1-oxide
4	STOCK1N-69616	340,391	C16H20N8O	F0-5347	>=92%	N-(2-(1H-imidazol-4-yl)ethyl)-1-(9H-purin-6-yl)piperidine-3-carboxamide
5	STOCK1N-71510	220,254	C9H8N4OS	F3-1627	>=95%	4-((9H-purin-6-yl)thio)but-2-yn-1-ol
6	STOCK1N-77176	311,303	C15H13N5O3	G1-6221	>=95%	8-(3-hydroxyphenyl)-1,7-dimethyl-1H-imidazo[2,1-f]purine-2,4(3H,8H)-dione
7	STOCK1N-79356	245,286	C12H15N5O	G3-6861	>=92%	N-(2-(1H-imidazol-4-yl)ethyl)-1,4,5,6-tetrahydrocyclopenta[c]pyrazole-3-carboxamide
8	STOCK1N-97980	301,261	C14H11N3O5	G7-7055	>=95%	7-(4-hydroxy-6-methyl-2-oxo-2H-pyran-3-yl)-1-methylpyrido[2,3-d]pyrimidine-2,4(1H,3H)-dione
9	STOCK1S-00944	294,318	C15H14N6O	B7-8791	>=95%	6-(4-(2-aminophenoxy)phenyl)-1,3,5-triazine-2,4-diamine
10	STOCK1S-06278	286,293	C14H14N4O3	B8-6123	>=95%	(E)-N'-((3-hydroxy-5-(hydroxymethyl)-2-methylpyridin-4-yl)methylene)isonicotinohydrazide
11	STOCK1S-10380	310,318	C15H14N6O2	B6-9004	>=95%	7,9-dimethyl-3-phenyl-7,9-dihydro-[1,2,4]triazino[3,4-f]purine-6,8(1H,4H)-dione
12	STOCK1S-69008	292,297	C17H12N2O3	B3-3583	>=95%	7-hydroxy-3-(1-methyl-1H-benzo[d]imidazol-2-yl)-2H-chromen-2-one

13	STOCK1S-78591	309,327	C17H15N3O3	B1-5531	>=95%	N-(3-hydroxyphenyl)-2-(4-methyl-1-oxophthalazin-2(1H)-yl)acetamide
14	STOCK2S-08448	277,285	C16H11N3O2	B5-1371	>=95%	3-(1H-benzo[d]imidazol-2-yl)-2-imino-2H-chromen-7-ol
15	STOCK2S-18844	370,371	C17H18N6O4	C3-9737	>=95%	(E)-7-allyl-8-(2-(2,4-dihydroxybenzylidene)hydrazinyl)-1,3-dimethyl-1H-purine-2,6(3H,7H)-dione
16	STOCK2S-29689	299,354	C15H13N3O2S	A1-4010	>=95%	5-((2,5-dimethyl-1-(pyridin-3-yl)-1H-pyrrol-3-yl)methylene)thiazolidine-2,4-dione
17	STOCK2S-34232	306,776	C13H11ClN4OS	B8-9275	>=95%	2-((9H-purin-6-yl)thio)-1-(4-chlorophenyl)ethanol
18	STOCK2S-52524	315,312	C13H9N5O3S	B8-9273	>=95%	2-((9H-purin-6-yl)thio)-1-(4-nitrophenyl)ethanone
19	STOCK2S-54227	270,315	C13H10N4OS	B8-9272	>=95%	2-((9H-purin-6-yl)thio)-1-phenylethanone
20	STOCK2S-60777	341,188	C11H9BrN4O2S	B0-0936	>=92%	(E)-4-((3-bromo-4-hydroxybenzylidene)amino)-6-methyl-3-thioxo-3,4-dihydro-1,2,4-triazin-5(2H)-one
21	STOCK2S-75169	318,282	C16H12F2N2O3	C0-7343	>=95%	4-((2,6-difluorobenzyl)oxy)-2-methyl-3-oxo-3,4-dihydroquinoxaline 1-oxide
22	STOCK3S-18016	348,362	C16H20N4O5	C3-1640	>=92%	(E)-2-(3-methyl-5-oxo-4,5-dihydro-1H-pyrazol-4-yl)-N'-(2,4,5-trimethoxybenzylidene)acetohydrazide
23	STOCK3S-18468	351,227	C13H11BrN4OS	C0-9433	>=95%	2-((9H-purin-6-yl)thio)-1-(4-bromophenyl)ethanol
24	STOCK3S-83435	342,355	C18H18N2O5	C8-7328	>=92%	4-((3,4-dimethoxybenzyl)oxy)-2-methyl-3-oxo-3,4-dihydroquinoxaline 1-oxide
25	STOCK3S-84679	312,284	C16H12N2O5	C8-8435	>=95%	N-(1,3-dioxoisoindolin-5-yl)-4,6-dimethyl-2-oxo-2H-pyran-5-carboxamide
26	STOCK4S-15629	354,369	C18H18N4O4	D0-9532	>=95%	4-(4-((3-hydroxypropyl)amino)-3-nitrophenyl)-2-methylphthalazin-1(2H)-one
27	STOCK4S-33920	367,364	C19H17N3O5	D5-5373	>=92%	N-((2-hydroxy-6-methoxyquinolin-3-yl)methyl)-N-methyl-3-nitrobenzamide
28	STOCK4S-46885	407,879	C18H18ClN3O4S	D6-0744	>=95%	1-(3-chloro-4-methylphenyl)-3-((4,6-dihydroxy-5-propylpyrimidin-2-yl)thio)pyrrolidine-2,5-dione

29	STOCK4S-62175	326,209	C12H9Cl2N5S	E0-9573	>=95%	6-((2,4-dichlorobenzyl)thio)-9H-purin-2-amine
30	STOCK4S-70675	362,778	C15H15ClN6O3	D4-9551	>=95%	(E)-8-(2-(4-chlorobenzylidene)hydrazinyl)-7-(2-hydroxyethyl)-3-methyl-1H-purine-2,6(3H,7H)-dione
31	STOCK4S-86355	372,424	C17H12N2O4S2	D7-1314	>=95%	N-((Z)-5-((E)-3-(furan-2-yl)allylidene)-4-oxo-2-thioxothiazolidin-3-yl)-4-hydroxybenzamide
32	STOCK5S-56194	205,172	C10H7NO4	B5-1608	>=92%	7-hydroxy-2-oxo-2H-chromene-3-carboxamide
33	STOCK5S-60009	273,228	C12H8FN5O2	E4-4779	>=92%	2-fluoro-N-(5-oxo-4,5-dihydro-[1,2,4]triazolo[1,5-a]pyrimidin-7-yl)benzamide
34	STOCK5S-74239	289,315	C13H11N3O3S	E7-5331	>=95%	(E)-5-((1,3-dimethyl-2-oxo-2,3-dihydro-1H-benzo[d]imidazol-5-yl)methylene)thiazolidine-2,4-dione
35	STOCK5S-79464	315,291	C14H13N5O4	E6-2330	>=95%	3,4-dimethoxy-N-(5-oxo-4,5-dihydro-[1,2,4]triazolo[1,5-a]pyrimidin-7-yl)benzamide
36	STOCK5S-83560	305,339	C14H19N5O3	E4-6387	>=95%	1-(1-(6-(bis(2-hydroxyethyl)amino)pyrimidin-4-yl)-5-methyl-1H-pyrazol-4-yl)ethanone
37	STOCK6S-28538	380,41	C19H20N6O3	F0-3035	>=95%	N-(2,3-dihydrobenzo[b][1,4]dioxin-6-yl)-1-(9H-purin-6-yl)piperidine-3-carboxamide
38	STOCK6S-72898	286,293	C14H14N4O3	F5-9017	>=95%	2-(4-(6-oxo-4,5,6,7-tetrahydro-2H-pyrazolo[3,4-b]pyridin-4-yl)phenoxy)acetamide
39	STOCK6S-83203	378,418	C17H14N8OS	F6-3748	>=95%	3-methyl-1-(9H-purin-6-yl)-4-(pyridin-3-yl)-6,8-dihydro-1H-pyrazolo[3,4-e][1,4]thiazepin-7(4H)-one
40	STOCK7S-44229	353,387	C17H19N7O2	G4-1485	>=95%	N-(6-methoxypyridin-3-yl)-1-(9H-purin-6-yl)piperidine-4-carboxamide
41	STOCK7S-51860	368,439	C20H24N4O3	G5-5819	>=95%	(S)-3-(5,7-dimethyl-2-oxo-1,2-dihydropyrazolo[1,5-a]pyrimidin-6-yl)-N-(1-hydroxy-3-phenylpropan-2-yl)propanamide
42	STOCK7S-53671	337,382	C19H19N3O3	G5-8695	>=92%	(S)-N-(1-hydroxy-3-phenylpropan-2-yl)-2-(4-oxo-3,4-dihydrophthalazin-1-yl)acetamide
